# Genome-Wide Identification and Characterization of Long Non-Coding RNA in Wheat Roots in Response to Ca^2+^ Channel Blocker

**DOI:** 10.3389/fpls.2018.00244

**Published:** 2018-03-06

**Authors:** Keshi Ma, Wenshuo Shi, Mengyue Xu, Jiaxi Liu, Feixiong Zhang

**Affiliations:** ^1^College of Life Sciences, Capital Normal University, Beijing, China; ^2^College of Life Science and Agronomy, Zhoukou Normal University, Zhoukou, China

**Keywords:** lncRNA, wheat, Ca^2+^-channel block, RNA-seq, transcription factor, cell cycle

## Abstract

It remains unclear whether plant lncRNAs are responsive to Ca^2+^-channel blocking. When using the Ca^2+^-channel blocker, LaCl_3_, to treat germinated wheat seeds for 24 h, we found that both root length and mitosis were inhibited in the LaCl_3_-treated groups. The effect of the Ca^2+^-channel blocker was verified in three ways: a [Ca^2+^]_cyt_ decrease detected using Fluo-3/AM staining, a decrease in the Ca content measured using inductively coupled plasma mass spectrometry, and an inhibition of Ca^2+^ influx detected using Non-invasive Micro-test Technology. Genome-wide high throughput RNA-seq and bioinformatical methods were used to identify lncRNAs, and found 177 differentially expressed lncRNAs that might be in responsive to Ca^2+^-channel blocking. Among these, 108 were up-regulated and 69 were down-regulated. The validity of identified lncRNAs data from RNA-seq was verified using qPCR. GO and KEGG analysis indicated that a number of lncRNAs might be involved in diverse biological processes upon Ca^2+^-channel blocking. Further GO analysis showed that 23 lncRNAs might play roles as transcription factor (TF); Moreover, eight lncRNAs might participate in cell cycle regulation, and their relative expressions were detected using qPCR. This study also provides diverse data on wheat lncRNAs that can deepen our understanding of the function and regulatory mechanism of Ca^2+^-channel blocking in plants.

## Introduction

Long non-coding RNAs (lncRNAs) are non-protein coding transcripts longer than 200 nucleotides and can be divided into at least five categories based on their structural characteristics, including intergenic lncRNAs, intronic lncRNAs, natural antisense transcripts, pseudogenes, and retrotransposons (Kitagawa et al., 2013). They have even been known as “transcriptional noise” under low expression (Ponjavic et al., 2007; Ponting et al., 2009). However, emerging studies have shown that lncRNAs could play a role in diverse biological processes via a number of complex mechanisms (Chekanova, 2015): they can serve as decoys, scaffolds, and guides (Rinn and Chang, 2012) to regulate gene expression in either *cis* or *trans* acting (Kang and Liu, 2015; Li et al., 2015), or they can serve as competing endogenous RNA (ceRNA) (Salmena et al., 2011) to compete with microRNA (miRNA) or interfere with the miRNA-mediated regulation of their mRNA targets (Rubio-Somoza et al., 2011; Fan et al., 2015).

The function of plant lncRNAs has mainly been reported in *Arabidopsis* and rice (Liu et al., 2015). They can function in *cis* and/or in *trans* by sequence complementarity or homology with DNA or RNAs, forming molecular frames and scaffolds for assembly of macromolecular complexes (Chekanova, 2015). Previous studies indicated that plant lncRNAs can play key roles in flowering time (Berry and Dean, 2015), gene silencing (Swiezewski et al., 2009; Bardou et al., 2014), root organogenesis (Matzke and Mosher, 2014), seedling photomorphogenesis (Wang Y. et al., 2014), and reproduction (Zhang et al., 2014).

It is promising that more plant lncRNAs have been identified in several other species, such as maize (Lv et al., 2016), cotton (Lu et al., 2016), *Populus trichocarpa* (Shuai et al., 2014), *Medicago truncatula* (Wang et al., 2015), and wheat (Xin et al., 2011; Shumayla et al., 2017) in response to a series of stresses such as cold, heat, drought, salt, and nitrogen. However, only a few biological functions of lncRNAs have been investigated in *Arabidopsis* and rice (Chekanova, 2015; Liu et al., 2015), such as, the lncRNA COLDAIR can regulate vernalization-mediated epigenetic silencing in responsive to cold stress (Heo and Sung, 2011), and cold induced lncRNA COOLAIR plays role in the early phase of vernalization (Swiezewski et al., 2009), a lncRNA LDMAR can regulate photoperiod-sensitive male sterility in hybrid rice (Ding et al., 2012); lncRNA DRIR can play roles as positive regulator in *Arabidopsis* response to drought and salt stress (Qin et al., 2017). So, more biological functions of lncRNAs involved in plant growth need to be investigated.

As a secondary messenger, Ca^2+^ is vital in plant growth and development (Hepler, 2005). The uptake of Ca^2+^ into cells is mediated by Ca^2+^-channels (White, 2000; Miedema et al., 2001; Demidchik and Tester, 2002). Previous studies implied that Ca^2+^-channels are involved in the regulation of cytosolic Ca^2+^ (Chinnusamy et al., 2004). The sustained blockage of the Ca^2+^-channels would decrease cytoplasmic Ca^2+^ concentrations, lead to calcium decrease, and affect many physiological, biochemical and metabolic processes in plants (Simon, 1978; Liu et al., 2013). LaCl_3_ is a widely used Ca^2+^-channel blocker (Lettvin et al., 1964; Takata et al., 1966; Choi et al., 2014). It has been shown that La^3+^ can inhibit plant growth (Hu et al., 2002; Diatloff et al., 2008). Unfortunately, the underling regulation mechanisms are unknown.

In this study, germinated wheat seeds were treated with different concentrations of LaCl_3_. It was shown that the growth of the roots was suppressed, and the mitotic index was also decreased. Detection using Fluo-3M staining, ICP-MS and NMT indicated that both the [Ca^2+^]_cyt_ and Ca content decreased significantly, and the Ca^2+^ influx was obviously inhibited in the LaCl_3_-treated group. Analysis using high throughput RNA-seq and bioinformatics revealed that eight lncRNAs might regulate the cell cycle by acting on their target genes. To the best of our knowledge, this is the first study on the molecular mechanisms of lncRNAs involved in cell cycle regulation in plant response to Ca^2+^-channel blocking.

## Materials and methods

### Plant growth and LaCl_3_ treatment

Seeds of the wheat cultivar “CB017-A” (Beijing Academy of Agriculture and Forestry Science, Beijing, China) were pre-treated with distilled water for 1 h and then placed in 10-cm Petri dishes on moistened filter paper for germination at 22°C under dark conditions. When the radicles emerged from the seed coats, the germinated seeds were grown in modified Hoagland + 0 (Control), 0.5, 1.0, 1.5, or 2.0 mM LaCl_3_ solution at 22°C under dark conditions for 24 h.

### Statistical analysis of the root length and mitotic index

The wheat primary root of each group was harvested and fixed in 3:1 absolute alcohol: acetic acid at 4°C for 24 h and then washed with distilled water three times. Following all treatments, the root tips were disassociated, macerated, stained, and squashed, and the mitotic index was calculated as described by Zhang et al. (2016).

### Detection of intracellular free Ca^2+^ distribution using Fluo-3/AM staining

To detect the distribution of intracellular free Ca^2+^ ([Ca^2+^]_cyt_) in the cells, the control and 1.5 mM LaCl_3_-treated roots were probed with Fluo-3/AM (Beyotime, Shanghai, China, #S1056) according to the protocol described by Zhang et al. (2016), and the control roots that were not treated by Fluo-3/AM served as the negative control. The levels of intracellular free Ca^2+^ in roots were visualized using a Zeiss LSM 5 live with an excitation wavelength of 488 nm and an emission wavelength of 525 nm (Wang et al., 2011), and the Ca^2+^ fluorescence intensity was quantified using Zeiss LSM Image Browser software (4.2).

### Measurement of Ca content using inductively coupled plasma mass spectrometry (ICP-MS)

To determine the Ca content in the wheat root-tip meristematic region, 200 μg of the control and 1.5 mM LaCl_3_-treated roots were completely dried and then digested in 5 mL concentrated nitric acid + 1 mL H_2_O_2_. Digested samples were diluted with ultra-pure water to 50 g. The Ca content in each group was measured using an Agilent 7500ce ICP-MS (Agilent Technologies, Santa Clara, USA).

### Measurement of Ca^2+^ flux using a non-invasive microtest technique (NMT)

To measure the Ca^2+^ flux in the meristematic region of the control and 1.5 mM LaCl_3_-treated wheat roots, non-invasive micro-test technology (NMT) (Xuyue Sci. & Tech. Co. Ltd. Beijing, China) was used. Briefly, Ca^2+^ ion-selective microelectrodes with an external tip diameter of 0.3 μm were manufactured, and only electrodes with Nernstian slopes >56 mV per decade were used. The samples were measured in the testing solution at 5 mM CaCl_2_ after the electrodes were calibrated at two Ca^2+^ levels (solution I, 0.1 mM CaCl_2_ and solution II, 10 mM CaCl_2_). The detailed methodology was presented by Tan et al. (2015).

All of the treatments, detections and analyses above were performed in at least three biological triplicates.

### RNA extraction and sequencing

Approximately 0.2 g of the control and 1.5 mM LaCl_3_-treated roots were ground to a fine powder in liquid nitrogen following the TRIzol (Invitrogen, Carlsbad, CA, USA, #15596-026) method for three independent replicates. The purified RNA was reverse transcribed using the RevertAid First Strand cDNA Synthesis Kit (Thermo, USA, K1066). RNA quality and integrity were assessed using an Agilent 2100 Bioanalyzer (Agilent Technologies, Santa Clara, CA). The Ribo-Zero rRNA Removal Kit (EpiCentre, Biotechnologies, USA) and the NEBNext® Ultra™RNA Library Prep Kit for Illumina (New England Biolabs, Beijing, China) were used to construct RNA-seq libraries according to the manufacturer's instructions. The resulting libraries were sequenced using an Illumina HiSeq™2000 (Illumina, USA) based on the paired-end method. The experiment of RNA sequencing was performed in three biological replicates in control and treatment, respectively.

### Identification of the putative lncRNAs in wheat roots

**Figure 1:**
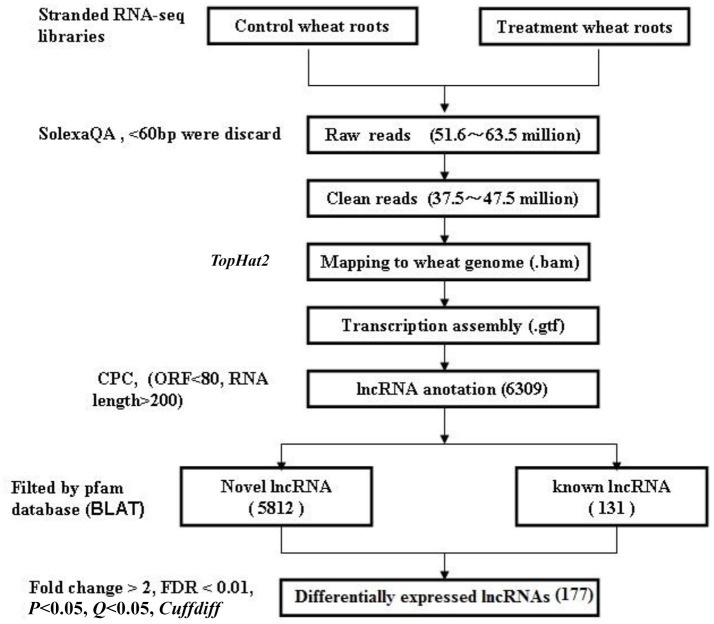
The flowchart for identifying lncRNAs in wheat roots responsive to Ca^2+^-channel blocker.

The flowchart of lncRNA identification is shown in Figure 1. Briefly, the high-throughput sequencing reads from all of the three biological replicates were pre-processed, and Cutadapt was used to remove adapters (Martin, 2011). The raw reads were filtered into clean reads using SolexaQA (those with ≤60 bp were discarded) (Cox et al., 2010). Then, using the TopHat 2.0 program (Trapnell et al., 2012), the assembled reads were mapped to the wheat genome: (ftp://ftp.ensemblgenomes.org/pub/release-27/plants/fasta/triticum_aestivum/dna/Triticum_aestivum.IWGSC1.0+popseq.27.dna.genome.fa.gz); genome alignment data (bam.) were acquired, and the RNA-seq saturation was measured using RSeQC (Wang et al., 2012). The alignment data were mapped to wheat lncRNA data: (ftp://ftp.ensemblgenomes.org/pub/plants/release-27/gtf/triticum_aestivum/Triticum_aestivum.IWGSC1.0+popseq.27.gtf.gz). The annotated transcriptomes were identified as conserved lncRNAs (known lncRNAs), the new transcriptomes were screened according to transcript length >200 nt and open reading frames (ORFs) <80 bp. CPC (coding potential calculator) was used to predict putative lncRNA, and BLAT (BLAST-Like Alignment Tool) was used to filter these lncRNAs by searching against the pfam database (*E* < 0.001) (Finn et al., 2014). CPC can search the sequences using a known protein sequence database to clarify the coding and non-coding transcripts mainly by assessing the extent and quality of the ORFs in the transcripts (Kong et al., 2007), and BLAT can identify sequence similarity in closely related genomes (Bhagwat et al., 2012). The lncRNA sequence reads were normalized to FPKM (fragments per kilobase of transcript per million mapped reads) values for each sample (Ashburner et al., 2000; Trapnell et al., 2010). FPKM and Cufflinks were used to analyze gene expression enrichment, and Cuffdiff was used to screen differentially expressed lncRNAs based on the following criteria: fold change>2 and *Q* < 0.05.

### Co-expression analysis of lncRNAs-mRNA-miRNA

The functional annotation of identified lncRNAs was performed using co-expression analysis (Mattick and Rinn, 2015). Based on the FPKM values of mRNAs and lncRNAs, Pearson's correlation coefficients between mRNAs and lncRNAs were calculated, and the putative target mRNA had to have a value >0.99 or < −0.99. In addition, the TargetFinder (Lavorgna et al., 1999) was used to predict the target mRNA and target lncRNA of the miRNA. Based on the correlations between lncRNAs, mRNAs and miRNAs, a lncRNA-mRNA-miRNA network was constructed using Cytoscape (Cline et al., 2007) software (Version3.0.2).

### Gene Ontology (GO) and Kyoto Encyclopedia of Genes and Genomes (KEGG) enrichment analysis

The predicted target genes were submitted to http://www.uniprot.org/downloads, and the GO ID and KO ID of each target gene were extracted. GO ID was submitted to Gene Ontology (http://geneontology.org/) using the tool of AmiGO 2, and the GO term of each target gene was annotated. The GO analysis was performed using GO Enrichment Analysis tool, and the Gene Functional Classification tool of DAVID (Database for Annotation, Visualization and Integrated Discovery) (http://david.abcc.ncifcrf.gov/) (Dennis et al., 2003; Huang et al., 2009) was used to search other functionally related genes from genome. Furthermore, the KO ID of each target gene was submitted to the Kyoto Encyclopedia of Genes and Genomes (KEGG) database (http://www.genome.ad.jp/KEGG/) to analyze the potential functions of these target genes in the pathways (Han et al., 2012; Li et al., 2013). Hyper-geometric distribution was employed to detect significant GO terms and KEGG pathways based on a significance level of 0.05.

### Quantitative real time polymerase chain reaction (qRT-PCR) analysis

Total RNAs (1 μg) from control and treatment were used to make cDNA using M-MLV Reverse Transcriptase (Takara, Japan) according to the supplier's protocol, respectively. After treatment with DNase I (Sigma, Germany), the cDNA was used as a template for qRT-PCR to quantify selected lncRNAs and mRNAs using the lncRNA-specific primers and target mRNA-specific primers. *GAPDH* and *Actin2* were used as the controls, and all experiments were conducted with at least three technical replications.

SYBR Green PCR was performed following the manufacturer's instructions (Takara, Japan). Briefly, 1 μl of cDNA template was added to 10 μl of 2 × SYBR Green PCR master mix (Takara, Dalian China), 1 μM of each primer, and ddH_2_O to a final volume of 20 μl. The reactions were amplified for 30 s at 95°C, followed by 36 cycles of 95°C for 10 s, 58°C for 30 s, and 72°C 10 s. All reactions were performed in triplicate. The relative expression level was analyzed using the 2^−ΔΔ*Ct*^ method.

### Statistical analysis

All experiments were repeated at least three times. The resulting data are presented as the mean ± standard error of the mean (SEM). Statistical comparisons between the control and treatment groups were conducted using Student's *t*-test or ANOVA, as well as Tukey's multiple comparisons test; *p* < 0.05 was considered significant, and *p* < 0.01 was considered highly significant. GraphPad Prism 5 (Graphpad Software, San Diego, CA, USA) was used for data and graphing analysis, and figures were appropriately processed using Photoshop CS5 (Adobe Systems, San Jose, CA, USA).

## Results

### Effects of the Ca^2+^-channel blocker LaCl_3_ on wheat root growth

**Figure 2:**
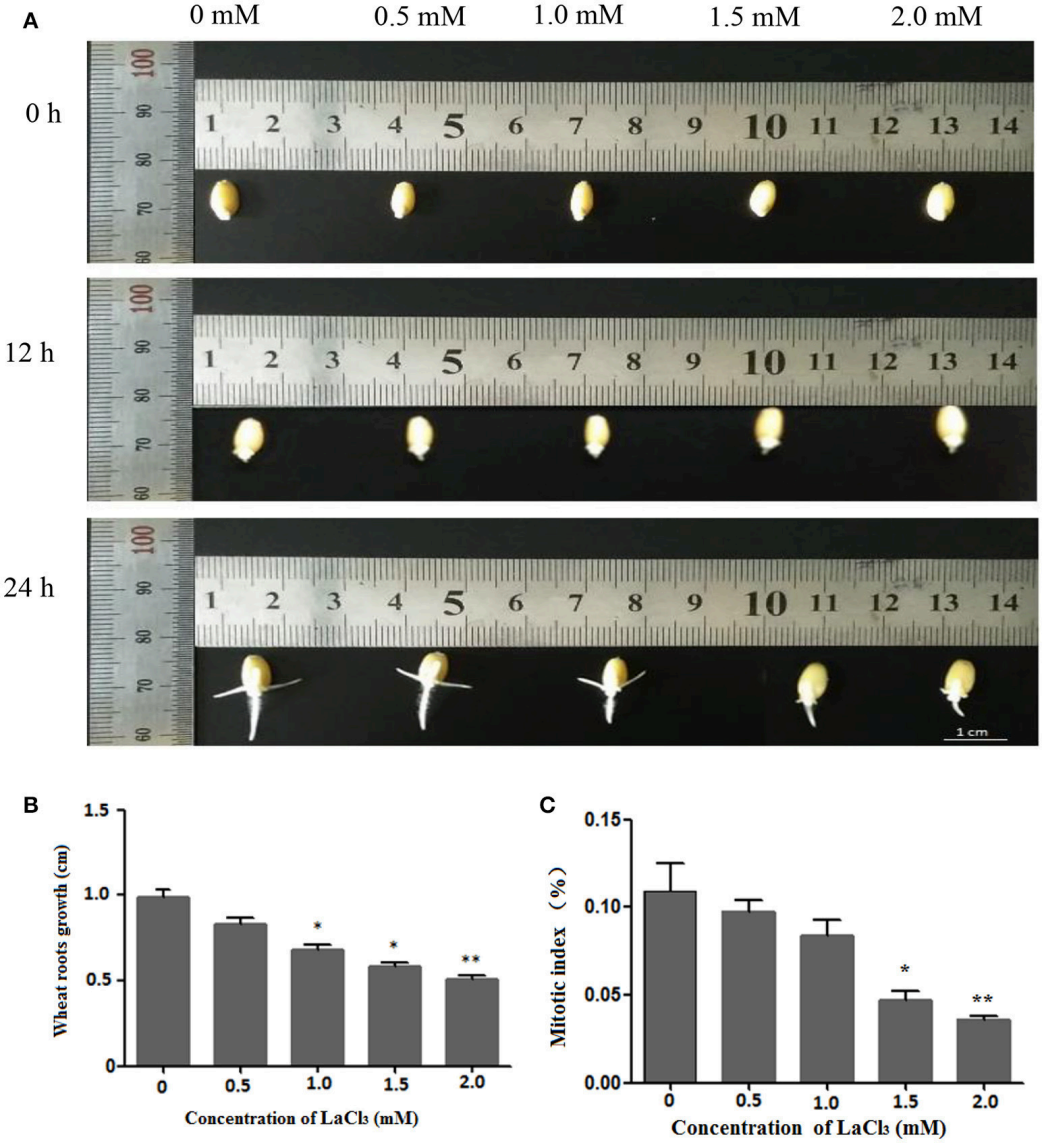
Wheat root length and mitotic index under different concentrations of LaCl_3_ (0 mM: New Hoagland solution; 0.5 mM: New Hoagland solution +0.5 mM LaCl_3_; 1.0 mM: New Hoagland solution +1.0 mM LaCl_3_; 1.5 mM: New Hoagland solution +1.5 mM LaCl_3_; 2.0 mM: New Hoagland solution +2.0 mM LaCl_3_). The experiments and statistical analyses were performed in three biological replicates. ^*^Significant difference (*p* < 0.05); ^**^Highly significant difference (*p* < 0.01). **(A)** Micrograph showing grown roots of germinated seeds in different groups at 0, 12, 24 h. **(B)** A bar chart showing the statistical results of the root length at 24 h. **(C)** A chart showing the statistical results of the mitotic index of wheat roots at 24 h. Scale bar = 1.0 cm.

Figure 2A depicts the growth status of the germinated wheat seeds treated with different concentrations of LaCl_3_ (0, 0.5, 1.0, 1.5, and 2 mM) for 0, 12, and 24 h. It is clear that the root lengths decreased with increasing concentrations of the drug. In addition, the inhibitory effects on root growth became more evident over time. Statistical analysis of the 24-h treatment indicated that the average length decreased by 83.82%, 68.55%, 58.65%, and 51.47% compared with the control group (Figure 2B), and the mitotic index decreased from 10.89% (control) to 9.75% and 8.38%, 4.70%, and 3.60%, respectively (Figure 2C). Both the wheat root length and mitotic index were significantly affected at a concentration of 1.5 mM.

These results showed that LaCl_3_ treatment can inhibit wheat root growth and decrease the mitotic index, and the effect of inhibition is positively correlated to the concentration of LaCl_3_.

### Effects of the Ca^2+^-channel blocker LaCl_3_ on [Ca^2+^]_cyt_ distribution, Ca content and Ca^2+^ flux in wheat roots

The distribution of intracellular free Ca^2+^, the Ca content and the Ca^2+^ flux in the meristematic regions were analyzed based on a comparison of 1.5 mM LaCl_3_-treated wheat roots relative to the control.

**Figure 3:**
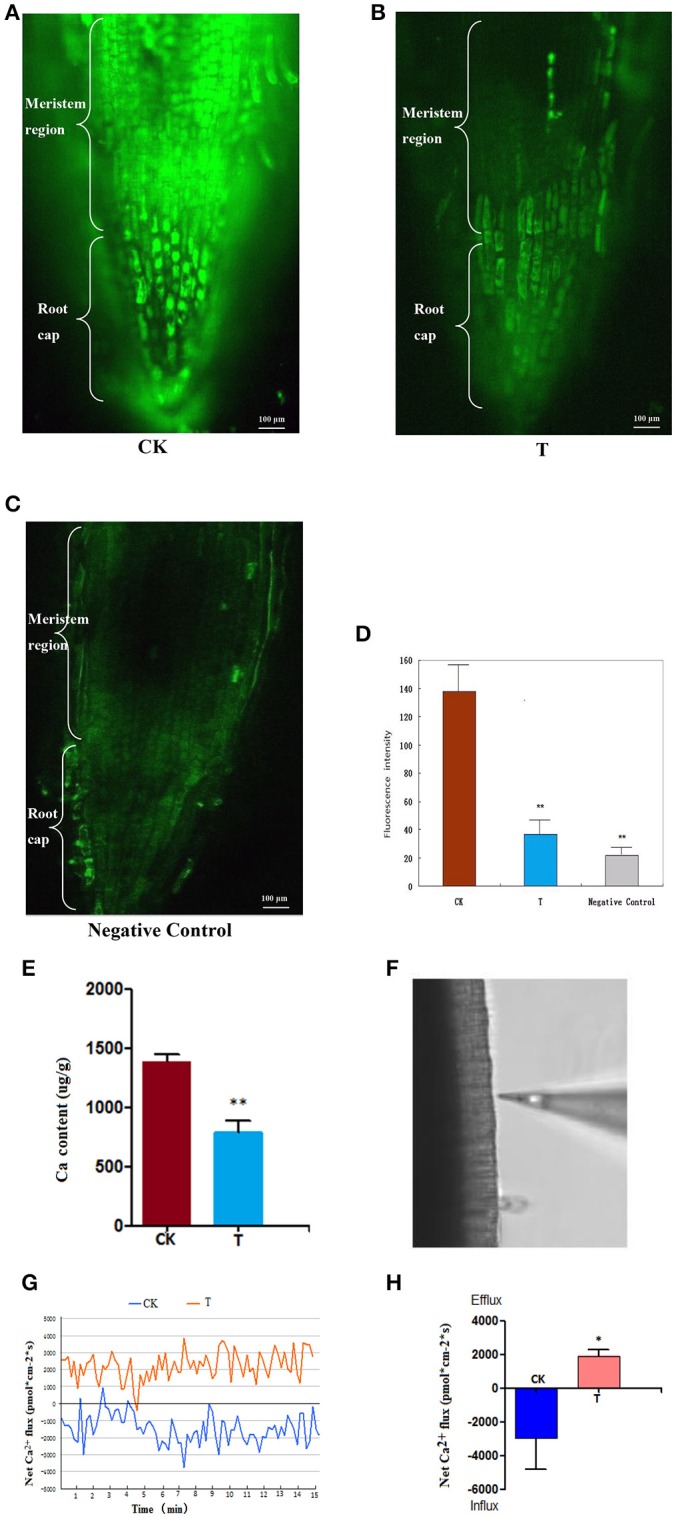
Ca^2+^ detected using Fluo-3/AM Staining, ICP-MS and NMT. All of the detections and the statistical analyses were performed in three biological replicates. ^*^Significant difference (*p* < 0.05); ^**^Highly significant difference (*p* < 0.01). **(A)** Confocal image of [Ca^2+^]_cyt_ in CK: the control wheat roots treated with Fluo-3/AM. Scale bar = 100 μm. **(B)** Confocal image of [Ca^2+^]_cyt_ in T: the 1.5 LaCl_3_- treated wheat roots treated with Fluo-3/AM. **(C)** Confocal image of [Ca^2+^]_cyt_ in the negative control: the control wheat roots not treated with Fluo-3/AM. **(D)** The chart showing the statistical results of the fluorescence intensity in meristematic regions. **(E)** Ca content in wheat measured using ICP-MS. **(F)** The image shows the measuring position using the Ca^2+^-selective microelectrode of NMT. **(G)** The image shows the kinetics of Ca^2+^ flux in wheat roots of CK and T. **(H)** A chart showing the statistical results of the net Ca^2+^ flux in meristematic regions at 24 h.

After the wheat roots were loaded with 20 μmol/L of Fluo-3/AM, a specific Ca^2+^ indicator (Li et al., 2012), it was observed under the confocal microscope that in the control (0 mM LaCl_3_), the fluorescence signals were strong and mainly distributed within the meristematic regions (Figure 3A). By contrast, in the LaCl_3_-treated roots, fluorescence labeling was much weaker and was only distributed in small areas of the meristematic regions (Figure 3B), and the fluorescence signals in the negative control were weaker than in the LaCl_3_ treatment (Figure 3C). The statistical analysis showed that the fluorescence intensity in the control was nearly three times greater than in the LaCl_3_ treatment, and there was a highly significant difference between the two groups (*p* < 0.01) (Figure 3D).

ICP-MS detection indicated that the average Ca content decreased from 1,385 (μg/g) in the control to 784 (μg/g) in the 1.5 mM-treated wheat roots. There was a highly significant difference between the control and treatment (*p* < 0.01) (Figure 3E).

The Ca^2+^ flux was detected at the surface of root meristem regions (Figure 3F), and the real-time kinetics of the Ca^2+^ flux in the root meristem regions (Figures 3G) recorded using NMT revealed that the average net Ca^2+^ flux was −2,955 pmol^*^cm^−2^^*^s in the control (influx), and 1,900 pmol^*^cm^−2^^*^s in the 1.5 mM LaCl_3_-treated samples (efflux) (Figure 3H). It is clear that the net Ca^2+^ flux was blocked after LaCl_3_ treatment.

It can be inferred from these results that LaCl_3_ treatment can block Ca^2+^ flux, such that extracellular Ca^2+^ cannot enter cells, causing Ca^2+^ deprivation.

### Profile of differentially expressed lncRNAs in wheat roots responsive to Ca^2+^-channel blocking

In order to understand the molecular mechanisms of wheat lncRNAs responsive to Ca^2+^-channel blocking, in this study, the RNAs from six wheat root samples (three control and three Ca^2+^-channel blocked treatment samples) were extracted and sequenced. We analyzed the RNA-Seq data from the triplicates, in which 51.6–63.5 million raw reads and 37.5–47.5 million clear reads per sample were obtained, and the raw reads were submitted to NCBI (SRA: SRP111314). The assembled clean reads were mapped to wheat genome using TopHat2, and the results indicated that the average alignment coverage was nearly 58% in all samples; uniquely mapped genes were about 46% (Table S1). The saturation of lncRNA from RNA-seq was measured by RSeQC and the result is shown in Figure S1, and the cluster or PCA results were shown in Figure S2. These results indicate that the quality of RNA-seq is good and reliable.

CPC was used to predict protein coding genes and lncRNAs. The results showed that 7,056 differentially expressed genes were identified (Supplementary Data 1), and a total of 6,309 putative lncRNAs were acquired, conserved lncRNAs were identified according to wheat lncRNA dataset (ftp://ftp.ensemblgenomes.org/pub/plants/release-27/gtf/triticum_aestivum/Triticum_aestivum.IWGSC1.0+popseq.27.gtf.gz), BLAT was used to submit to pfam database to remove potential coding transcripts, and 5,943 lncRNAs were identified. Among the 5,943 lncRNAs, 131 were known lncRNAs and 5,812 were novel lncRNAs (Figure 1). A heat map (Figure S3) and the Volcano matrix (Figure S4) indicated that a number of transcripts were differentially expressed in the control and treatment groups; further analysis showed that 177 lncRNAs were differently expressed in wheat roots responsive to Ca^2+^-channel blocking; their general information was shown in Table S2, and their sequence is shown in Supplementary Data 2. These 177 lncRNAs were classified into five classes: 90 sense lncRNAs, one antisense lncRNA, 75 intergenic lncRNAs, one intronic lncRNA, and 10 pseudogenes (Table 1). Among them, 108 lncRNAs containing 49 sense, one antisense, 54 intergenic, and one intronic lncRNAs and three pseudogenes were up-regulated, and another 69 lncRNAs containing 41 sense, 21 intergenic lncRNAs and seven pseudogenes were down-regulated (Table 1).

**Table 1:** Identification of differentially expressed lncRNAs in wheat roots responsive to Ca^2+^-channel blocker.

	**Sense**	**Antisense**	**Intergenic**	**Intronic**	**Pseudogene**	**Total**
Up-regulation	49	1	54	1	3	108
Down-regulation	41	0	21	0	7	69
Total	90	1	75	1	10	177

**Figure 4:**
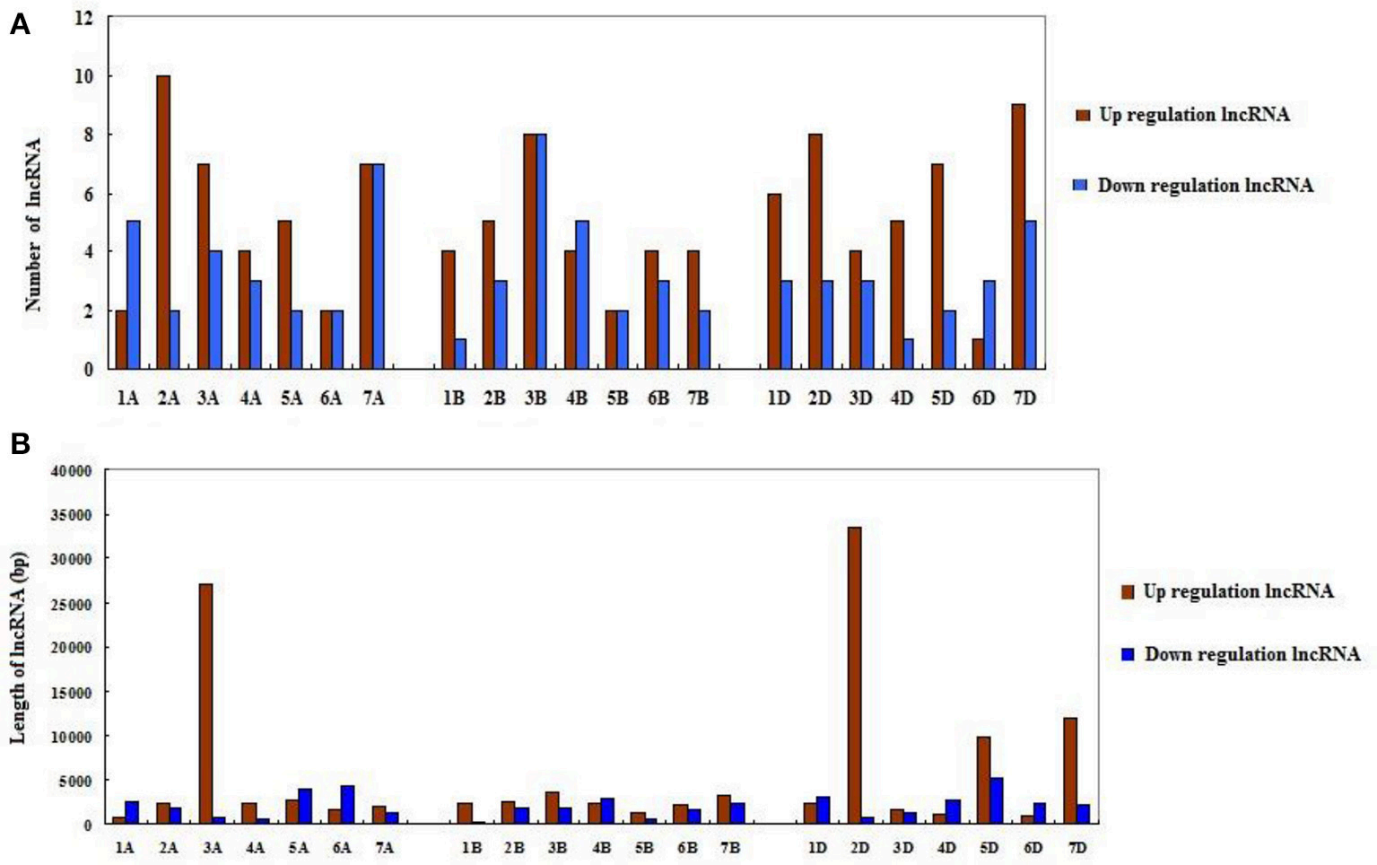
The distribution of differentially expressed lncRNAs in the wheat genome. **(A)** The number of differentially expressed lncRNAs on different chromosomes. **(B)** The length of differentially expressed lncRNAs on different chromosomes.

Since wheat is an allohexaploid with three distinct subgenomes, A, B, and D, we analyzed the situation of all differentially expressed lncRNAs in the three subgenomes (Table 2). In subgenome A, there were 62 differentially expressed lncRNAs with 37 up-expressed and 25 down-expressed; 32 were sense, 29 intergenetic and one intonic lncRNAs; the average length of up-expressed lncRNAs was 5,547 bp and that of down-expressed lncRNAs was 2,198 bp. In subgenome B, there were 55 lncRNAs with 31 up-expressed and 24 down-expressed; 23 were sense, 22 were intergenetic and 10 were pseudogenes lncRNAs; the average length of up-expressed lncRNAs was 2,499 bp and that of down-expressed lncRNAs was 1,655 bp. In subgenome D, there were 60 lncRNAs with 40 up-expressed and 20 down-expressed; 35 were sense, one was antisense and 24 were intergenetic lncRNAs; the average length of up-expressed lncRNAs was 8,748 bp and that of down-expressed lncRNAs was 2,498 bp. Furthermore, the numbers of lncRNAs distributed among the different chromosomes were shown in Figure 4A. The average length of differentially expressed lncRNAs distributed in subgenomes, A, B, and D was analyzed and is shown in Figure 4B: the average length ranged from 277 to 33,466 bp; the shortest lncRNA was on 1B and the longest lncRNA was on 2D. Notably, 47 of the total 177 differentially expressed lncRNAs were scaffolds, i.e., ~27%. Therefore, it was difficult to identify their exact location within the genome.

**Table 2:** The situation of differentially expressed lncRNAs among A, B, and D subgenome in wheat responsive to Ca^2+^-channel blocker.

**Subgenome**	**Number**			**Class**					**Average length**	
	**Total lncRNAs**	**Up-expressed lncRNA**	**Down-expressed lncRNA**	**Sense**	**Antisense**	**Intergenetic**	**Intonic**	**Pseudogene**	**Up-expressed lncRNA**	**Down-expressed lncRNA**
A	62	37	25	32	0	29	1	0	5,547	2,198
B	55	31	24	23	0	22	0	10	2,499	1,655
D	60	40	20	35	1	24	0	0	8,748	2,498

To confirm the differential expression of lncRNAs identified with genome-wide RNA-seq, qRT-PCR was performed on eight randomly selected lncRNAs (Table S3) using specific primers (Table S4). The results showed that four lncRNAs (lncRNA_024812, lncRNA_046989, lncRNA_032897, and lncRNA_042235) were down-regulated (Figure S5a), and four lncRNAs (lncRNA_013190, lncRNA_039803, lncRNA_053211, and lncRNA_014373) were up-regulated (Figure S5b). Their relative expressions were consistent with the RNA-seq data, this result indicated that the identification of lncRNAs was applicable and reliable.

### Function analysis of differentially expressed lncRNAs in wheat roots responsive to Ca^2+^-channel blocking

Until now, the functions of most wheat lncRNAs have not been annotated. The functional prediction of lncRNAs is based on the annotations of co-expressed mRNAs (Li et al., 2017). Therefore, we calculated and constructed an lncRNA-mRNA co-expressed correlation based on their expression enrichment (FPKM); this result is presented in Table S5. Moreover, the lncRNA-miRNA co-expression correlation was also calculated and is shown in Table S6, and the miRNA-mRNA co-expression correlation is shown in Table S7. Based on the co-expression correlations of lncRNA-mRNA and lncRNA-miRNA, we found that 32 lncRNAs were related to 63 miRNAs, of which 21 lncRNAs were positively correlated with 58 miRNAs, and 12 lncRNAs were negatively correlated with 16 miRNAs (Table 3). In addition, 165 lncRNAs were related to 1,626 mRNAs, of which 157 lncRNAs were positively correlated with 1,253 mRNAs, and 82 were negatively correlated with 738 mRNAs (Table 3). Furthermore, an miRNA-lncRNA-mRNA network was constructed (Figure S6) based on the co-expression correlation of lncRNA-mRNA, lncRNA-miRNA, and miRNA-mRNA. In the network, we found a common feature, i.e., that a number of miRNAs, lncRNAs and mRNAs were correlated, and one lncRNA might be related to more than one miRNA and/or mRNAs.

**Table 3:** The correlation of co-expressed lncRNA-miRNA and lncRNA-mRNA.

**Correlation**	**lncRNA-miRNA**	**lncRNA-mRNA**
Positive	24–58	157–1,253
Negative	12–16	82–738
Total	32–63	165–1,626

To further study the function of these lncRNAs, GO enrichment analysis was performed using the GO website (http://geneontology.org/). The result showed that these differentially expressed lncRNAs can play roles in many biological processes, cellular components and molecular functions. Among all target genes, 324 were up-regulated (Figure S7a), and 161 were down-regulated (Figure S7b). The significant down-regulated GO terms and the significant up-regulated GO terms were also analyzed. We found that “water transport,” “water channel activity,” “hydrogen peroxide transmembrane transport,” “chloroplast thylakoid membrane,” “plastoglobule,” “response to abscisic acid,” “mitochondrial envelope,” “Golgi membrane,” “ubiquitin protein ligase binding,” “primary cell wall biogenesis” and “response to absence of light” were down-regulated, and “vacuolar membrane,” “ATP binding,” “vacuole,” “cytosol” were up-regulated (Figures S7c,d).

KEGG pathway analysis was performed using the website: http://www.genome.jp/kegg. It was observed that “phenylalanine metabolism,” “phenylpropanoid biosynthesis,” “biosynthesis of secondary metabolites,” “metabolic pathways,” “plant-pathogen interaction,” “peroxisome,” “PI3K-Akt signaling pathway” and “cell cycle pathway” were down-regulated (Figure S7e), and “endocytosis,” “ascorbate and aldarate metabolism” and “glutathione metabolism” were up-regulated (Figure S7f). The down-regulated pathways had higher significance levels than those of the up-regulated pathways.

### Identification of lncRNAs related to transcription factor and cell cycle regulation in wheat roots stressed by Ca^2+^-channel blocking

To further study the function of lncRNAs in the growth of wheat roots in response to Ca^2+^-channel blocking, the GO terms of differentially expressed lncRNAs were annotated based on http://geneontology.org/ and http://www.uniprot.org/uniprot/. The results showed that 23 lncRNAs might be involved in the regulation of gene transcription, because their target genes have transcription factor activity (Table 4), of them eight were for down-regulation and 15 were for up-regulation; Five were negative to their target genes and 18 were positive to their target genes. Furthermore, homologous genes were identified using Blast tool kit (version 31) (Camacho et al., 2009), the result showed that 14 target genes were homologous in rice genome (Table S8).

**Table 4:** The profile of 23 differentially expressed lncRNAs related to transcription factor.

**LncRNA name**	**Log_2_ (T/CK)**	**Target_gene**	**Correlation**	***P*-value**	**GO term accession**	**Function description**
lncRNA_000823	−0.980413	Traes_1AL_88D49649D	0.991908859	9.79E-05	GO:0003700	transcription factor activity, sequence-specific DNA binding
lncRNA_020477	−1.21932	Traes_4AS_F04DD4409	0.990987147	0.000121481	GO:0003700	transcription factor activity, sequence-specific DNA binding
lncRNA_029088	−1.30935	Traes_3DL_8FD0F859B	0.993705177	5.93E-05	GO:0003700	transcription factor activity, sequence-specific DNA binding
lncRNA_029384	−1.09603	Traes_5BL_F5D379AFC	0.992006664	9.56E-05	GO:0003700	transcription factor activity, sequence-specific DNA binding
**lncRNA_053766**	−1.49756	Traes_7AL_25850F96F	−**0.994553129**	4.44E-05	GO:0003700	transcription factor activity, sequence-specific DNA binding
lncRNA_072935	−1.04047	Traes_5DL_91AE6CA271	0.993558546	6.21E-05	GO:0003700	transcription factor activity, sequence-specific DNA binding
lncRNA_083996	−1.43547	TRAES3BF051200110CFD_g	0.992314697	8.84E-05	GO:0003700	transcription factor activity, sequence-specific DNA binding
XLOC_001557	−0.684295	TRAES3BF091100240CFD_g	0.991173887	0.000116507	GO:0043433	negative regulation of sequence-specific DNA binding transcription factor activity
**lncRNA_033754**	0.932921	Traes_4DL_2527CA8BF	−**0.995906832**	2.51E-05	GO:0003700	transcription factor activity, sequence-specific DNA binding
lncRNA_006270	2.08992	Traes_2BL_FC0F8A3DC	0.993479972	6.36E-05	GO:0003700	transcription factor activity, sequence-specific DNA binding
lncRNA_008977	1.06478	Traes_2DL_04535D371	0.994148858	5.13E-05	GO:0003700	transcription factor activity, sequence-specific DNA binding
lncRNA_014639	1.39391	Traes_3AS_965E2F790	0.992930736	7.48E-05	GO:0003700	transcription factor activity, sequence-specific DNA binding
**lncRNA_018111**	1.26842	Traes_6AL_0C0899C15	−**0.994888702**	3.91E-05	GO:0003700	transcription factor activity, sequence-specific DNA binding
lncRNA_021433	2.45667	Traes_6DL_6DC75B590	0.996733717	1.60E-05	GO:0003700	transcription factor activity, sequence-specific DNA binding
lncRNA_043877	2.48297	Traes_7BL_625F55A12	0.992872581	7.60E-05	GO:0003700	transcription factor activity, sequence-specific DNA binding
**lncRNA_051318**	2.31774	Traes_4BL_9BCD28A4E	−**0.992200754**	9.10E-05	GO:0003700	transcription factor activity, sequence-specific DNA binding
lncRNA_057390	1.23621	Traes_1BL_BDF0801D01	0.991344416	0.000112054	GO:0003700	transcription factor activity, sequence-specific DNA binding
lncRNA_063547	1.65342	Traes_7DL_310E46F15	0.993894918	5.58E-05	GO:0003700	transcription factor activity, sequence-specific DNA binding
lncRNA_064639	1.58444	TRAES3BF063000030CFD_g	0.997884348	6.71E-06	GO:0003700	transcription factor activity, sequence-specific DNA binding
**lncRNA_068995**	1.44673	TRAES3BF025700030CFD_g	−**0.990586196**	0.000132512	GO:0003700	transcription factor activity, sequence-specific DNA binding
lncRNA_072748	1.21533	Traes_4AS_02B607421	0.994010715	5.37E-05	GO:0003700	transcription factor activity, sequence-specific DNA binding
lncRNA_074658	1.65571	TRAES3BF066400010CFD_g	0.991399622	0.000110632	GO:0001076	transcription factor activity, RNA polymerase II transcription factor binding
lncRNA_078349	1.97463	Traes_7DL_A9EF00572	0.992798512	7.76E-05	GO:0003700	transcription factor activity, sequence-specific DNA binding

*The bold values mean the lncRNAs were negative correlation to target genes*.

Interestingly, we found eight lncRNAs might be involved in cell cycle regulation (Table 5). These eight lncRNAs and their target genes were all located on different chromosomes, and their features and functions are shown in Table 5. Among these, lncRNA_082364 was intergenic lncRNA and was up-regulated; its target gene, Traes_2BL_E5A7188DB (code RCC1 family protein, regulator of chromosome condensation), was annotated to cell division (GO:0051301) and the mitotic cell cycle (GO:0000278); lncRNA_047461 was sense lncRNA and was up-regulated; its target gene, Traes_7BS_E11EC3E6E (GCR1-cAMP receptor), was annotated to the mitotic cell cycle (GO:0000278); lncRNA_074658 was intergenic lncRNA and was up-regulated, and its target genes, Traes_5DS_7722ED6BA (code Protein AAA, ATPase family) and TRAES3BF066400010CFD_g (code B3 family protein, DNA bonding, regulation of transcription), were annotated to cell division (GO:0051301); lncRNA_008977 was intergenic lncRNA and was up-regulated; its target gene, Traes_6BS_01CD46D81 (code A20-like zinc finger family), was annotated to mitotic nuclear division (GO:0007067); lncRNA _061738 was intergenic lncRNA and was up-regulated; its target gene, Traes_6AS_0A0B33CEF (Uncharacterized), was annotated to regulation of G2/M transition of the mitotic cell cycle (GO:0010389); lncRNA_018111 was intergenic lncRNA and was up-regulated; its target gene, Traes_5DL_128F9DE77 (TPR-like super family), was annotated to regulation of the cell cycle (GO:0051726) and DNA replication (GO:0006275); and lncRNA_000823 was intergenic lncRNA and was down-regulated; its target gene, Traes_5BL_922358DB7 (code ATP banding protein, serine/threonine kinase activity), was annotated to chromosome segregation (GO:0007059) and the mitotic cell cycle (GO:0000278). Furthermore, we found one lncRNA, lncRNA_058136 (sense, down-regulated), and its target genes, TRAES3BF024700350CFD_g and Traes_3AS_8A727B48F, coded the 14-3-3 family protein, an important regulation protein in the cell cycle (Ferl et al., 2002; Sato et al., 2002; Jin et al., 2006). KEGG analysis also showed that the “14-3-3” hub in both the “PI3K-Akt signaling pathway” and “Cell cycle pathway” was down regulated (Figure S8).

**Table 5:** Putative lncRNAs involved in the cell cycle in wheat roots responsive to Ca^2+^-channel blocking.

**LncRNA (class)**	**Locus**	**Expression**	**Correlation**	**Target-genes**	**Function of the Target-gene (http://www.uniprot.org)**	**GO term**
lncRNA_082364 (intergenetic)	IWGSC_CSS_7BL_scaff_6744266:2212-3128	Up	0.99	Traes_2BL_E5A7188DB	RCC1 family:Regulator of chromosome condensation	GO (0051301): cell division; GO:0000278: mitotic cell cycle
lncRNA_047461 (sense)	6D:171673293-171673674	Up	0.99	Traes_7BS_E11EC3E6E	GCR1-cAMP receptor	GO (0000278): mitotic cell cycle
lncRNA_074658 (intergenetic)	IWGSC_CSS_5BL_scaff_10892062:1651-3092	Up	0.99	Traes_5DS_7722ED6BA	Cell Division Protein AAA ATPase family	GO (0051301): cell division
				TRAES3BF066400010CFD_g	B3 families (DNA bonding, regulation of transcription)	
lncRNA_008977 (intergenetic)	2A:43623104-43626665	Up	−0.99	Traes_6BS_01CD46D81	A20-like zinc finger family	GO (0007067): mitotic nuclear division
lncRNA_061738 (intergenetic)	IWGSC_CSS_2BL_scaff_8062645:3991-5001	Up	−0.99	Traes_6AS_0A0B33CEF	Uncharacterized	GO (0010389): regulation of G2/M transition of mitotic cell cycle
lncRNA_018111 (intergenetic)	3A:179057334-179222831	Up	−0.99	Traes_5DL_128F9DE77	TPR-like super family (Tetratricopeptide repeat)	GO (0051726): regulation of cell cycle; GO (0006275: regulation of DNA replication
lncRNA_000823 (intergenetic)	1A:224565164-224566779	Down	0.99	Traes_5BL_922358DB7	ATP banding, protein serine/threonine kinase activity	GO (0007059): chromosome segregation; GO (0000278): mitotic cell cycle
lncRNA_058136 (sense)	IWGSC_CSS_1DL_scaff_1677028:995-5235	Down	0.99	TRAES3BF024700350CFD_g	14-3-3 family	GO (0019904): protein domain specific binding
				Traes_3AS_8A727B48F		

**Figure 5:**
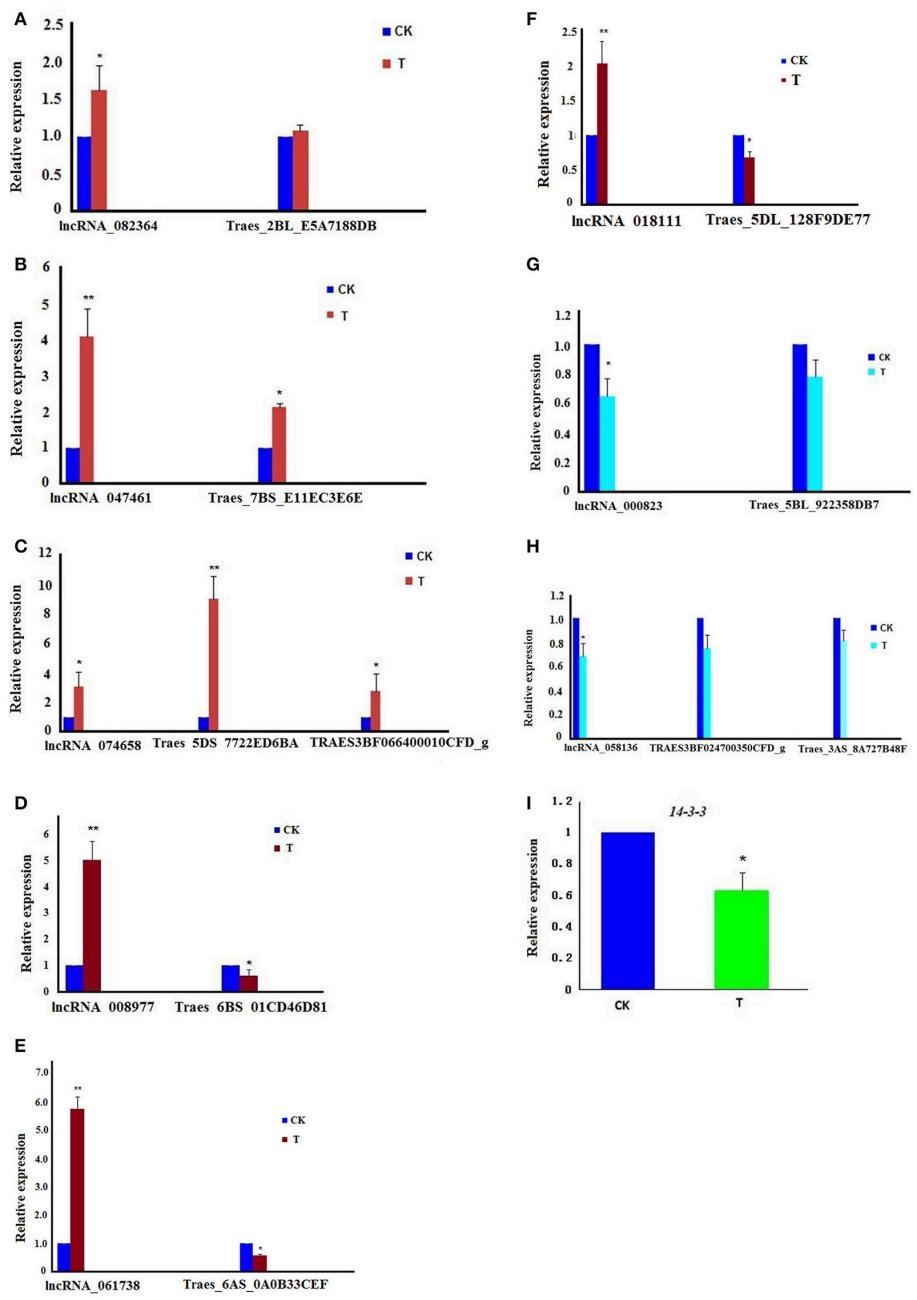
The relative expression of lncRNAs involved in the cell cycle and their target genes; the experiments of q-PCR and the data analyses were performed in three biological replicates. ^*^Significant difference (*p* < 0.05); ^**^Highly significant difference (*p* < 0.01). **(A)** The chart shows that both lncRNA_082364 and its target gene, Traes_2BL_E5A7188DB, were up-regulated. **(B)** The chart shows that both lncRNA_047461 and its target gene, Traes_7BS_E11EC3E6E, were up-regulated. **(C)** The chart shows that lncRNA_074658 and its target genes, Traes_5DS_7722ED6BA and TRAES3BF066400010CFD_g, were up-regulated. **(D)** The chart shows that lncRNA_008977 was up-regulated, but its target gene, Traes_6BS_01CD46D81, was down-regulated. **(E)** The chart shows that lncRNA_061738 was up-regulated, but its target gene, Traes_6AS_0A0B33CEF was down-regulated. **(F)** The chart shows that lncRNA_018111 was up-regulated, but its target gene, Traes_5DL_128F9DE77 was down-regulated. **(G)** The chart shows that both lncRNA_000823 and its target gene, Traes_5BL_922358DB7, were down-regulated; **(H)** The chart shows that lncRNA_058136 was down-regulated, and its target genes, TRAES3BF024700350CFD_g and Traes_3AS_8A727B48F, were down-regulated. **(I)** The chart shows that the *14-3-3* gene was down-regulated.

Using specific primers for these lncRNAs and their target genes (Table S9), real-time q-PCR was performed. The result showed that all eight lncRNAs were consistent with the results of RNA-seq data (Figures 5A–H). Moreover, the relative expression of *14-3-3* was down-regulated in the treatment group (Figure 5I).

### Analysis of target mirnas of lncRNAs involved in the cell cycle

Micro RNAs (miRNA) can play important role in plants responsive to abiotic stress (Sunkar et al., 2007), and one of the important functions of lncRNAs was serving as primary miRNAs (primiRNA) (Diederichs, 2015). In this study, we analyzed the potential primiRNAs from all identified lncRNAs using TargetFinder (Lavorgna et al., 1999), the result showed that eight lncRNAs might be precursor of four miRNAs (Table S10). Among them, four sites of lncRNA_051551 were matched with bdi-miR1127_R18-4L21; one site of lncRNA_054399 was matched with mtr-miR7701-5p_R18-4L21; two sites of lncRNA_042235 were matched with ssp-miR444b.2_R15-1L21; one site of lncRNA_033754 was matched with ssp-miR444b.2_R15-1L21; two site of lncRNA_034367 were matched with ssp-miR444b.2_R15-1L21; two site of lncRNA_054399 were matched with ssp-miR444b.2_R15-1L21; one site of lncRNA_033754 was matched with tae-miR1121_R3-21L22 and one site of lncRNA_072440 was also matched with tae-miR1121_R3-21L22. However, they were not identified as differentially expressed lncRNAs.

**Figure 6:**
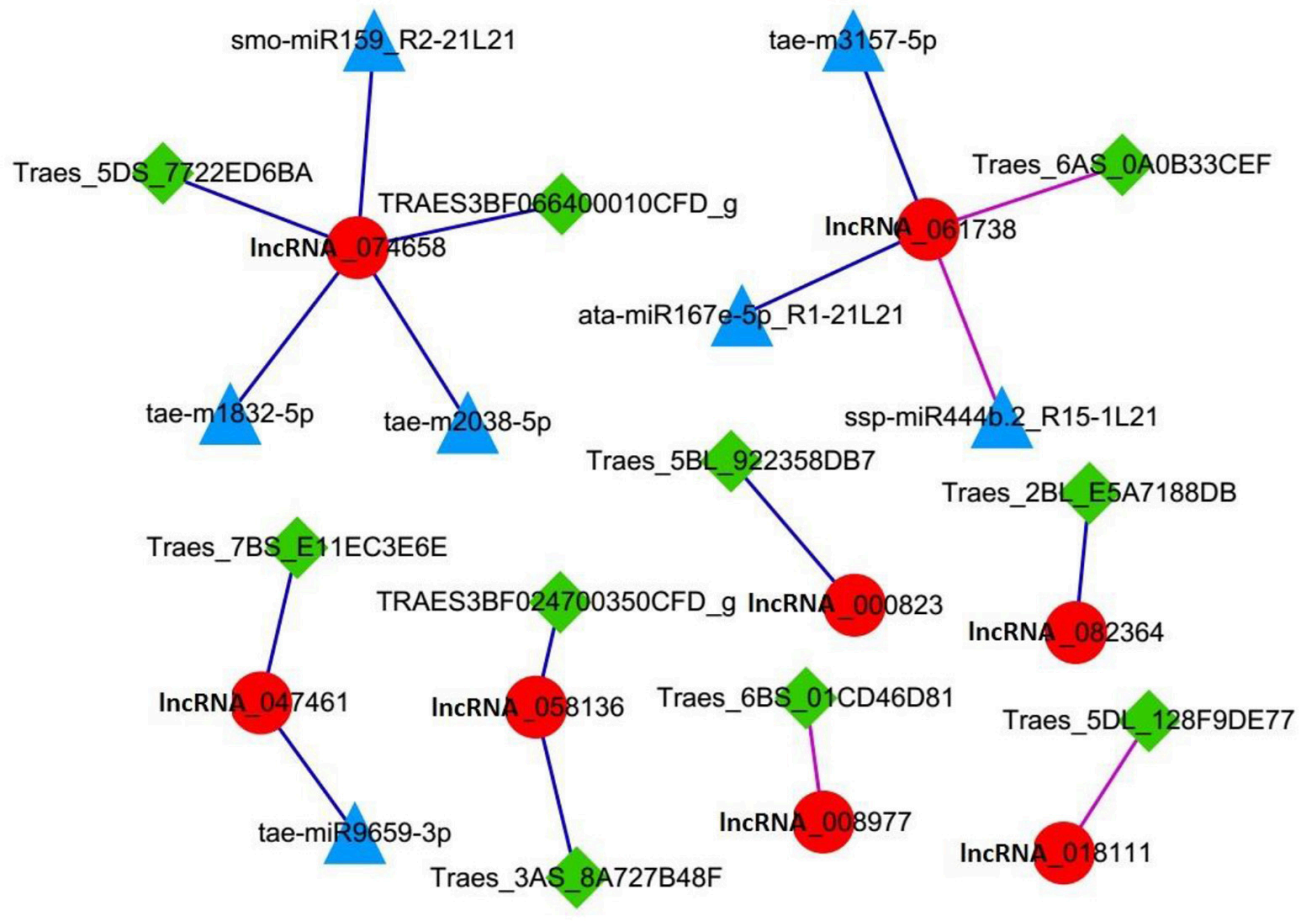
Network of lncRNAs involved in the cell cycle. Red circle node: lncRNA. Square green nodes: mRNA. Triangular blue nodes: miRNA.

Further analysis of lncRNAs involved in cell cycle regulation was performed based on the co-expression correlation of lncRNA-miRNA (Table S6). We found three lncRNAs, lncRNA_047461, lncRNA_074658 and lncRNA_061738 were correlated with seven miRNAs (Table 6). Among these, lncRNA_047461 had a positive co-expression relationship with tae-miR9659-3p (>0.99), lncRNA_074658 was positively co-expressed with tae-m1832-5p, tae-m2038-5p and smo-miR159_R2-21L21, and lncRNA_061738 was positively co-expressed with ata-miR167e-5p_R1-21L21 and tae-m3157-5p and negatively with ssp-miR444b.2_R15-1L21. The network (Figure 6) was constructed based on the correlation between mRNA-lncRNA and lncRNA-miRNA. The result showed that lncRNA_047461, lncRNA_074658 and lncRNA_061738 might not only interact with target genes but also interact with miRNAs. Interestingly, we found that miR59 might be involved in cell cycle regulation because it was reported to affect the expression of TCP (teosinte branched cycloidea PCF) (Palatnik et al., 2007). TCP is necessary for PCNA in cell proliferation (Li et al., 2015), and TCP plays a role in the development of diverse organs via the cell cycle (Danisman, 2016).

**Table 6:** Putative lncRNAs involved in the cell cycle and target miRNAs.

**lncRNA**	**miRNA**	**Correlation**	**Functional description of miRNA**
lncRNA_047461	tae-miR9659-3p	>0.99	Unknown
lncRNA_074658	smo-miR159_R2-21L21	>0.99	Target to TCP, TCP is necessary for PCNA, cell proliferation
lncRNA_074658	tae-m1832-5p	>0.99	Unknown
lncRNA_074658	tae-m2038-5p	>0.99	Unknown
lncRNA_061738	ata-miR167e-5p_R1-21L21	>0.99	Unknown
lncRNA_061738	tae-m3157-5p	>0.99	Unknown
lncRNA_061738	ssp-miR444b.2_R15-1L21	<-0.99	Unknown

This result indicated there were three lncRNAs in the cell cycle regulation that were positively correlated with seven miRNAs, and they might interact with their target miRNAs and target mRNAs simultaneously.

## Discussion

Wheat (*Triticum aestivum* L., AABBDD, 2*n* = 6*x* = 42) was the first domesticated crop plant and has become one of the most important crops grown for our daily life. Wheat occupies ~17% of all cultivated land and provides >20% of carbohydrates (Gill et al., 2004; Xin et al., 2011). As a typical polyploidy plant, the genome of common wheat is both large and complex (Brenchley et al., 2012; Mayer et al., 2014), providing it greater physiological and ecological plasticity (Dubcovsky and Dvorak, 2007; Feldman et al., 2012; Yang et al., 2014). Thus, it is necessary to explore the regulation mechanisms in the wheat growth process.

Plant growth relies on cell cycle progression (Sablowski and Carnier Dornelas, 2013), which is regulated by numerous internal and external factors. Ca^2+^ is one of the key factors. It has been demonstrated that low levels of Ca^2+^ affect the plant cell cycle (Hepler, 2005); however, the underlying functional properties are unclear. Some researchers have described the functions of lncRNAs in cell cycle regulation in animals (Kitagawa et al., 2013; Li et al., 2016). However, to date, no lncRNAs have been found to be involved in cell cycle regulation in plants responsive to Ca^2+^ deprivation.

In this study, when the germinated wheat seeds were treated with the specific Ca^2+^-channel blocker, LaCl_3_, we found that wheat root growth was inhibited and the mitotic index decreased. We comprehensively investigated the Ca^2+^ change in the plants using Fluo-3M staining, ICP-MS and NMT, and demonstrated that Ca^2+^ deprivation in LaCl_3_-treated wheat roots indeed resulted from Ca^2+^-channel blockage.

To understand the molecular mechanisms of the growth of wheat roots responsive to the Ca^2+^-channel blocker, high throughput RNA-seq was performed. A total of 5,943 transcripts were identified as putative lncRNAs, and 177 were differentially expressed lncRNAs responsive to the Ca^2+^-channel blocker. Xin et al. identified 125 wheat lncRNAs responsive to powdery mildew infection and heat stress (Xin et al., 2011) that are not conserved among plant species. These 177 lncRNAs (not including the 10 pseudogenes) had no homologs or significant matches to known plant lncRNAs, were novel and wheat specific; this result is consistent with a previous study on *Arabidopsis* (MacIntosh et al., 2001). GO and KEGG analysis indicated that these lncRNAs might play a role in many biological processes in wheat root growth, such as biological regulation, cell proliferation and metabolic processes.

The most-known function of lncRNAs is regulation of gene transcription, and they can directly regulate the Pol II transcription machinery (Chekanova, 2015). Previous study indicated that animal lncRNAs can promote the phosphorylation of transcription factors and regulate their DNA-binding activity (Wang P. et al., 2014). In *Arabidopsis, trans-acting* lncRNA HID1 associates with the chromatin of the TF gene PIF3 and can repress its transcription (Wang Y. et al., 2014). The lncRNA APOLO can participate in the spatial association and interaction between APOLO and the distant PID genomic regions via formation of a dynamic chromatin loop that determines PID expression (Ariel et al., 2014). In this study, we found that 23 lncRNAs might play roles as regulators of transcription, their target genes were annotated to transcription factor activity (Table 4), and 14 of them were homologous in rice genome (Table S8). Among them, lncRNA_074658 regulates polymerase II transcription binding factor, and another 22 lncRNAs annotated to transcription factor activity, can regulate sequence-specific DNA binding. Since five of them were negatively correlated with their target genes, and the others were positively correlated with their target genes, they may play roles via *cis-acting* or *trans-acting*. However, their exact mechanisms need to be investigated.

Interestingly, we found that seven lncRNAs, lncRNA_082364, lncRNA_047461, lncRNA_074658, lncRNA_008977, lncRNA_061738, lncRNA_018111, and lncRNA_000823, might play a role in cell cycle regulation because their target genes are involved in DNA replication, chromosome condensation, and G2/M transition. Furthermore, lncRNA_058136 might also regulate the cell cycle, because its target gene can code the 14-3-3 family protein. KEGG pathway analysis indicated that *14-3-3* is involved in the “PI3K-Akt signaling pathway” and “cell cycle pathway,” and the “14-3-3” hub was down-regulated in both pathways (Figures S8a,b). The “PI3K-Akt signaling pathway” is a well-known pathway that regulates the cell cycle progress (Okkenhaug and Vanhaesebroeck, 2003; Engelman et al., 2006; Duronio, 2008) and plays an essential role in cell survival and cell growth via direct or indirect regulation of apoptotic factors and cell cycle regulators (Nicholson and Anderson, 2002; Liang and Slingerland, 2003; Manning and Cantley, 2007; Zhang et al., 2011). A previous study indicated that some lncRNAs are involved in cell cycle regulation in animals (Kitagawa et al., 2013), but no lncRNAs have been found to be involved in cell cycle regulation in plants. Therefore, our finding might be the starting point for investigating the function of plant lncRNAs in cell cycle regulation.

In order to reveal the regulatory mechanism of the lncRNAs involved in the cell cycle, the relative expression of the eight lncRNAs and their target genes were detected (Figure 5): lncRNA_082364, lncRNA_047461, and lncRNA_074658 were up-regulated and positively related to their target genes; lncRNA_008977, lncRNA_061738, and lncRNA_018111 were up-regulated but negatively related to their target genes; and lncRNA_000823 and lncRNA_058136 were down-regulated and positively related to their target genes. Based on target gene expression, various regulatory strategies have been proposed for lncRNAs (Quinodoz and Guttman, 2014), including the activation (Zhang et al., 2011) and repression (Rinn et al., 2007; Huarte and Rinn, 2010) of genes in *cis-acting* and/or in *trans-acting* factors (Rinn et al., 2007; Zhang et al., 2011). In this study, we found that the eight lncRNAs might play different roles in cell cycle regulation, i.e., lncRNA_082364, lncRNA_047461, lncRNA_074658, lncRNA_000823, and lncRNA_058136 might be involved in activation, whereas lncRNA_008977, lncRNA_061738 and lncRNA_018111 might be involved in repression. Since these lncRNAs and their target genes are located on different chromosomes, we speculated that they may all play a role as *trans*-acting factors. However, the exact regulatory mechanism requires considerable investigation, and more studies on the over-expression of lncRNA genes or knock-out genes in wheat will shed further light on the regulatory mechanisms.

The competing endogenous RNAs (ceRNA) hypothesis (Rubio-Somoza et al., 2011; Salmena et al., 2011) states that ceRNAs including mRNA, lncRNAs, pseudogenes, and other miRNA sponges, share common miRNA binding sites and can act as molecular sponges to compete for given miRNAs (Rubio-Somoza et al., 2011; Xu et al., 2016). The ceRNA phenomenon has been found in maize and rice (Fan et al., 2015; Xu et al., 2016). However, to date it has not been reported in wheat. In this study, we found seven miRNAs had co-expression correlation with three lncRNAs: lncRNA_047461, lncRNA_074658, and lncRNA_061738 (Table 5). Among these, smo-miR159_R2-21L21 belong to the miRNA159 family, and miRNA159 might play a role in cell proliferation by affecting TCP (Palatnik et al., 2007), which is essential for PCNA (Ferl et al., 2002). PCNA, as a cell-cycle marker, is an evolutionarily conserved protein in all eukaryotic species (Strzalka and Ziemienowicz, 2011). Although the function of other miRNAs is unclear, according to the lncRNA network involved in the cell cycle (Figure 6) and the differentially expressed lncRNA-mRNA-miRNA network (Figure S6), we speculated that lncRNA_047461, lncRNA_074658, and lncRNA_061738 might act as decoys or sponges to compete for miRNAs, regulate gene expression, and play a role in wheat growth and cell cycle regulation. To the best of our knowledge, only a few regulatory mechanisms of lncRNAs involved in the cell cycle have been reported in animals (Kitagawa et al., 2013). This study suggests that lncRNAs might participate in cell regulation in plants.

In summary, in this study, we found that wheat root growth was inhibited after treatment with the Ca^2+^-channel blocker, LaCl_3_; Ca^2+^decreased in the wheat roots, and [Ca^2+^]_cyt_ was reduced. In order to investigate the molecular mechanism of wheat responsive to the Ca^2+^-channel blocker, genome-wide identification of lncRNAs was performed using high throughput RNA-Seq and bioinformatics analysis. We found 177 putative wheat lncRNAs were responsive to the Ca^2+^-channel blocker; further function analysis indicated that a number of lncRNAs might be involved in wheat growth. Among them, 23 lncRNAs were predicated as transcription factors, and 14 of them were homologous in rice. Interestingly, eight lncRNAs might be involved in cell cycle regulation, and the functional analysis showed that these lncRNAs might regulate target genes through *trans-acting* or act as ceRNAs to compete for certain miRNAs. Furthermore, this study will benefit an in-depth understanding of the function and regulatory mechanisms in plants responsive to Ca^2+^-channel blocking.

## Author contributions

KM, JL, and FZ: designed the experiment; KM, WS, MX, and FZ: collected and analyzed the data; KM and FZ: wrote the manuscript. All authors discussed the results and contributed to the manuscript.

## Additional information

Supplementary information accompanies this paper at http://journal.frontiersin.org/journal/plant-science.

### Conflict of interest statement

The authors declare that the research was conducted in the absence of any commercial or financial relationships that could be construed as a potential conflict of interest.

## References

[B1] Ariel F. Jegu T. Latrasse D. Romero-Barrios N. Christ A. Benhamed M.. (2014). Noncoding transcription by alternative RNA polymerases dynamically regulates an auxin-driven chromatin loop. Mol. Cell 55, 383–396. 10.1016/j.molcel.2014.06.011 25018019

[B2] Ashburner M. Ball C. A. Blake J. A. Botstein D. Butler H. Cherry J. M.. (2000). Gene ontology: tool for the unification of biology. The gene ontology consortium. Nat. Genet. 25, 25–29. 10.1038/75556 10802651 PMC3037419

[B3] Bardou F. Ariel F. Simpson C. G. Romero-Barrios N. Laporte P. Balzergue S.. (2014). Long noncoding RNA modulates alternative splicing regulators in *Arabidopsis*. Dev. Cell. 30, 166–176. 10.1016/j.devcel.2014.06.017 25073154

[B4] Berry S. Dean C. (2015). Environmental perception and epigenetic memory: mechanistic insight through FLC. Plant J. 83, 133–148. 10.1111/tpj.12869 25929799 PMC4691321

[B5] Bhagwat M. Young L. Robison R. R. (2012). Using BLAT to find sequence similarity in closely related genomes. Curr. Protoc. Bioinformatics. 37, 1–24. 10.1002/0471250953.bi1008s37 PMC4101998 22389010

[B6] Brenchley R. Spannagl M. Pfeifer M. Barker G. L. A. D'Amore R. Allen A. M.. (2012). Analysis of the bread wheat genome using whole-genome shotgun sequencing. Nature 491, 705–710. 10.1038/nature11650 23192148 PMC3510651

[B7] Camacho C. Coulouris G. Avagyan V. Ma N. Papadopoulos J. Bealer K.. (2009). Blast+: architecture and applications. BMC Bioinformatics 10:421. 10.1186/1471-2105-10-421 20003500 PMC2803857

[B8] Chekanova J. A. (2015). Long non-coding RNAs and their functions in plants. Curr. Opin. Plant Biol. 27, 207–216. 10.1016/j.pbi.2015.08.003 26342908

[B9] Chinnusamy V. Schumaker K. Zhu J. K. (2004). Molecular genetic perspectives on cross-talk and specificity in abiotic stress signalling in plants. J. Exp. Bot. 55, 225–236. 10.1093/jxb/erh005 14673035

[B10] Choi W. G. Toyota M. Kim S. H. Hilleary R. Gilroy S. (2014). Salt stress-induced Ca^2+^ waves are associated with rapid, long-distance root-to-shoot signaling in plants. Proc. Natl. Acad. Sci. U.S.A. 111, 6497–6502. 10.1073/pnas.1319955111 24706854 PMC4035928

[B11] Cline M. S. Smoot M. Cerami E. Kuchinsky A. Landys N. Workman C.. (2007). Integration of biological networks and gene expression data using Cytoscape. Nat. Protoc. 2, 2366–2382. 10.1038/nprot.2007.324 17947979 PMC3685583

[B12] Cox M. P. Peterson D. A. Biggs P. J. (2010). SolexaQA: at-a-glance quality assessment of Illumina second-generation sequencing data. BMC Bioinformatics. 11:485. 10.1186/1471-2105-11-485 20875133 PMC2956736

[B13] Danisman S. (2016). TCP transcription factors at the interface between environmental challenges and the plant's growth responses. Front. Plant Sci. 7:1930. 10.3389/fpls.2016.01930 28066483 PMC5174091

[B14] Demidchik V. Tester M. (2002). Sodium fluxes through nonselective cation channels in the plasma membrane of protoplasts from Arabidopsis roots. Plant Physiol. 128, 379–387. 10.1104/pp.010524 11842142 PMC148901

[B15] Dennis G. J. Sherman B. T. Hosack D. A. Yang J. Gao W. Lane H. C.. (2003). DAVID: database for annotation, visualization, and integrated discovery. Genome Biol. 4:R60. 10.1186/gb-2003-4-9-r60 12734009 PMC193660

[B16] Diatloff E. Smith F. W. Asher C. J. (2008). Effects of lanthanum and cerium on the growth and mineral nutrition of corn and mungbean. Ann. Bot. 101, 971–982. 10.1093/aob/mcn021 18292604 PMC2710236

[B17] Diederichs S. (2015). Micro-terminator: ‘Hasta la vista, lncRNA!’. Nat. Struct. Mol. Biol. 22, 279–281. 10.1038/nsmb.3001 25837873

[B18] Ding J. Lu Q. Ouyang Y. Mao H. Zhang P. Yao J.. (2012). A long noncoding RNA regulates photoperiod-sensitive male sterility, an essential component of hybrid rice. Proc. Natl. Acad. Sci. U.S.A. 109, 2654–2659. 10.1073/pnas.1121374109 22308482 PMC3289353

[B19] Dubcovsky J. Dvorak J. (2007). Genome plasticity a key factor in the success of polyploidy wheat under domestication. Science 316, 1862–1866. 10.1126/science.1143986 17600208 PMC4737438

[B20] Duronio V. (2008). The life of a cell: apoptosis regulation by the PI3K/PKB pathway. Biochem. J. 415, 333–344. 10.1042/BJ20081056 18842113

[B21] Engelman J. A. Luo J. Cantley L. C. (2006). The evolution of phosphatidylinositol 3-kinases as regulators of growth and metabolism. Nat. Rev. Genet. 7, 606–619. 10.1038/nrg1879 16847462

[B22] Fan C. Hao Z. Yan J. Li G. (2015). Genome-wide identification and function analysis of lincRNAs acting as miRNA targets or decoys in maize. BMC Genomics 16:793. 10.1186/s12864-015-2024-0 26470872 PMC4608266

[B23] Feldman M. Levy A. A. Fahima T. Korol A. (2012). Genomic asymmetry in allopolyploid plants: wheat as a model. J. Exp. Bot. 63, 5045–5059. 10.1093/jxb/ers192 22859676

[B24] Ferl R. J. Manak M. S. Reyes M. F. (2002). The 14-3-3s. Genome Biol. 3, 1–7. 10.1186/gb-2002-3-7-reviews3010 12184815 PMC139387

[B25] Finn R. D. Bateman A. Clements J. Coggill P. Eberhardt R. Y. Eddy S. R.. (2014). Pfam: the protein families database. Nucleic Acids Res. 42, D222–D230. 10.1093/nar/gkt1223 24288371 PMC3965110

[B26] Gill B. S. Appels R. Botha-Oberholster A. M. Buell C. R. Bennetzen J. L. Chalhoub B.. (2004). A workshop report on wheat genome sequencing: international genome research on wheat consortium. Genetics 168, 1087–1096. 10.1534/genetics.104.034769 15514080 PMC1448818

[B27] Han Z. D. Zhang Y. Q. He H. C. Dai Q. S. Qin G. Q. Chen J. H.. (2012). Identification of novel serological tumor markers for human prostate cancer using integrative transcriptome and proteome analysis. Med. Oncol. 29, 2877–2888. 10.1007/s12032-011-0149-9 22215415

[B28] Heo J. B. Sung S. (2011). Vernalization-mediated epigenetic silencing by a long intronic noncoding RNA. Science 331, 76–79. 10.1126/science.1197349 21127216

[B29] Hepler P. K. (2005). Calcium: a central regulator of plant growth and development. Plant Cell 17, 2142–2155. 10.1105/tpc.105.032508 16061961 PMC1182479

[B30] Hu X. Ding Z. Wang X. Chen Y. Dai L. (2002). Effects of lanthanum and cerium on the vegetable growth of wheat (*Triticum aestivum* L.) seedlings. Bull. Environ. Contam. Toxicol. 69, 727–733. 10.1007/s00128-002-0121-7 12375123

[B31] Huang D. W. Sherman B. T. Lempicki R. A. (2009). Systematic and integrative analysis of large gene lists using DAVID bioinformatics resources. Nat. Protoc. 4, 44–57. 10.1038/nprot.2008.211 19131956

[B32] Huarte M. Rinn J. H. (2010). Large non-coding RNAs: missing links in cancer? Hum. Mol. Genet. 19, 152–161. 10.1093/hmg/ddq353 20729297 PMC2953740

[B33] Jin Y. Dai M. S. Lu S. Z. Xu Y. Luo Z. Zhao Y.. (2006). 14-3-3γ binds to MDMX that is phosphorylated by UV-activated Chk1, resulting in p53 activation. EMBO J. 25, 1207–1218. 10.1038/sj.emboj.7601010 16511572 PMC1422168

[B34] Kang C. Liu Z. (2015). Global identification and analysis of long non-coding RNAs in diploid strawberry *Fragaria vesca* during flower and fruit development. BMC Genomics 16:815. 10.1186/s12864-015-2014-2 26481460 PMC4617481

[B35] Kitagawa M. Kitagawa K. Kotake Y. Niida H. Ohhata T. (2013). Cell cycle regulation by long non-coding RNAs. Cell Mol. Life Sci. 70, 4785–4794. 10.1007/s00018-013-1423-0 23880895 PMC3830198

[B36] Kong L. Zhang Y. Ye Z. Q. Liu X. Q. Zhao S. Q. Wei L.. (2007). CPC: assess the protein-coding potential of transcripts using sequence features and support vector machine. Nucleic Acids Res. 35, 345–349. 10.1093/nar/gkm391 17631615 PMC1933232

[B37] Lavorgna G. Guffanti A. Borsani G. Ballabio A. Boncinelli E. (1999). TargetFinder: searching annotated sequence databases for target genes of transcription factors. Bioinformatics 15, 172–173. 10.1093/bioinformatics/15.2.172 10089203

[B38] Lettvin J. Y. Pickard W. F. McCulloth W. S. Pitts W. (1964). A theory of passive ion flux through axon membrane. Nature 202, 1338–1339. 10.1038/2021338a0 14210975

[B39] Li C. Qiao Z. Qi W. Wang Q. Yuan Y. Yang X.. (2015). Genome-wide characterization of cis-acting DNA targets reveals the transcriptional regulatory framework of opaque2 in maize. Plant Cell 27, 532–545. 10.1105/tpc.114.134858 25691733 PMC4558662

[B40] Li J. Li C. Wang J. Song G. Zhao Z. Wang H.. (2017). Genome-wide analysis of differentially expressed lncRNAs and mRNAs in primary gonadotrophin adenomas by RNA-seq. Oncotarget 8, 4585–4606. 10.18632/oncotarget.13948 27992366 PMC5354857

[B41] Li J. Tian H. Jie Y. Gong Z. (2016). Long noncoding RNA regulate cell growth, proliferation, and apoptosis. DNA Cell Biol. 35, 459–470. 10.1089/dna.2015.3187 27213978

[B42] Li S. P. Xie W. L. Cai H. H. Cai J. Y. Yang P. H. (2012). Hydroxyl radical scavenging mechanism of human erythrocytes by quercetin-germanium (IV) complex. Eur. J. Pharm. Sci. 47, 28–34. 10.1016/j.ejps.2012.04.019 22579957

[B43] Li X. Luo J. Yan T. Xiang L. Jin F. Qin D.. (2013). Deep sequencing-based analysis of the *Cymbidium ensifolium* floral transcriptome. PLoS ONE 8:e85480. 10.1371/journal.pone.0085480 24392013 PMC3877369

[B44] Liang J. Slingerland J. M. (2003). Multiple roles of the PI3K/PKB (Akt) pathway in cell cycle progression. Cell Cycle 2, 339–345. 10.4161/cc.2.4.433 12851486

[B45] Liu D. Wang X. Zhang X. Gao Z. (2013). Effects of lanthanum on growth and accumulation in roots of rice seedlings. Plant Soil Environ. 59, 196–200. 10.17221/760/2012-PSE

[B46] Liu J. Wang H. Chua N. H. (2015). Long noncoding RNA transcriptome of plants. Plant Biotechnol. J. 13, 319–328. 10.1111/pbi.12336 25615265

[B47] Lu X. Chen X. Mu M. Wang J. Wang X. Wang D.. (2016). Genome-wide analysis of long noncoding RNAs and their responses to drought stress in cotton (*Gossypium hirsutum* L.). PLoS ONE 11:e0156723. 10.1371/journal.pone.0156723 27294517 PMC4905672

[B48] Lv Y. Liang Z. Ge M. Qi W. Zhang T. Lin F.. (2016). Genome-wide identification and functional prediction of nitrogen-responsive intergenic and intronic long non-coding RNAs in maize (*Zea mays* L.). BMC Genomics 17:350. 10.1186/s12864-016-2650-1 27169379 PMC4865003

[B49] MacIntosh G. C. Wilkerson C. Green P. J. (2001). Identification and analysis of Arabidopsis expressed sequence tags characteristic of non-coding RNAs. Plant Physiol. 127, 765–776. 10.1104/pp.010501 11706161 PMC129250

[B50] Manning B. D. Cantley L. C. (2007). AKT/PKB signaling: navigating downstream. Cell 129, 1261–1274. 10.1016/j.cell.2007.06.009 17604717 PMC2756685

[B51] Martin M. (2011). Cutadapt removes adapter sequences from high-throughput sequencing reads. EMBnet J. 17, 10–12. 10.14806/ej.17.1.200

[B52] Mattick J. S. Rinn J. L. (2015). Discovery and annotation of long noncoding RNAs. Nat. Struct. Mol. Biol. 22, 5–7. 10.1038/nsmb.2942 25565026

[B53] Matzke M. A. Mosher R. A. (2014). RNA-directed DNA methylation: an epigenetic pathway of increasing complexity. Nat. Rev. Genet. 15, 394–408. 10.1038/nrg3683 24805120

[B54] Mayer K. F. X. Rogers J. DoleŽel J. Pozniak C. Eversole K. Feuillet C.. (2014). A chromosome-based draft sequence of the hexaploid bread wheat (*Triticum aestivum* L.) genome. Science 345, 1251788. 10.1126/science.1251788 25035500

[B55] Miedema H. Bothwell J. H. F. Brownlee C. Davies J. M. (2001). Calcium uptake by plant cells–channels and pumps acting in concert. Trends Plant Sci. 6, 514–519. 10.1016/S1360-1385(01)02124-0 11701379

[B56] Nicholson K. M. Anderson N. G. (2002). The protein kinase B/Akt signalling pathway in human malignancy. Cell Signal. 14, 381–395. 10.1016/S0898-6568(01)00271-6 11882383

[B57] Okkenhaug K. Vanhaesebroeck B. (2003). PI3K in lymphocyte development, differentiation and activation. Nat. Rev. Immunol. 3, 317–330. 10.1038/nri1056 12669022

[B58] Palatnik J. F. Wollmann H. Schommer C. Schwab R. Boisbouvier J. Rodriguez R.. (2007). Sequence and expression differences underlie functional specialization of *Arabidopsis*, microRNAs miR159 and miR319. Dev. Cell 13, 115–125. 10.1016/j.devcel.2007.04.012 17609114

[B59] Ponjavic J. Ponting C. P. Lunter G. (2007). Functionality or transcriptional noise? Evidence for selection within long noncoding RNAs. Genome Res. 17, 556–565. 10.1101/gr.6036807 17387145 PMC1855172

[B60] Ponting C. P. Oliver P. L. Reik W. (2009). Evolution and functions of long noncoding RNAs. Cell 136, 629–641. 10.1016/j.cell.2009.02.006 19239885

[B61] Qin T. Zhao H. Cui P. Albesher N. Xiong L. (2017). A nucleus-localized long non-coding RNA enhances drought and salt stress tolerance. Plant Physiol. 175, 1321–1336. 10.1104/pp.17.00574 28887353 PMC5664461

[B62] Quinodoz S. Guttman M. (2014). Long noncoding RNAs: an emerging link between gene regulation and nuclear organization. Trends Cell Biol. 24, 651–663. 10.1016/j.tcb.2014.08.009 25441720 PMC4254690

[B63] Rinn J. L. Chang H. Y. (2012). Genome regulation by long noncoding RNAs. Annu. Rev. Biochem. 81, 145–166. 10.1146/annurev-biochem-051410-092902 22663078 PMC3858397

[B64] Rinn J. L. Kertesz M. Wang J. K. Squazzo S. L. Xu X. Brugmann S. A.. (2007). Functional demarcation of active and silent chromatin domains in human *HOX* loci by non-coding RNAs. Cell 129, 1311–1323. 10.1016/j.cell.2007.05.022 17604720 PMC2084369

[B65] Rubio-Somoza I. Weigel D. Franco-Zorilla J. M. García J. A. Paz-Ares J. (2011). CeRNAs: miRNA target mimic mimics. Cell 147, 1431–1432. 10.1016/j.cell.2011.12.003 22196719

[B66] Sablowski R. Carnier Dornelas D. M. (2013). Interplay between cell growth and cell cycle in plants. J. Exp. Bot. 65, 2703–2714. 10.1093/jxb/ert354 24218325

[B67] Salmena L. Poliseno L. Tay Y. Kats L. Pandolfi P. P. (2011). A ceRNA hypothesis: the Rosetta Stone of a hidden RNA language? Cell 146, 353–358. 10.1016/j.cell.2011.07.014 21802130 PMC3235919

[B68] Sato M. Watanabe Y. Akiyoshi Y. Yamamoto M. (2002). 14-3-3 protein interferes with the binding of RNA to the phosphorylated form of fission yeast meiotic regulator Mei2p. Curr. Biol. 12, 141–145. 10.1016/S0960-9822(01)00654-6 11818066

[B69] Shuai P. Liang D. Tang S. Zhang Z. Ye C. Y. Su Y.. (2014). Genome-wide identification and functional prediction of novel and drought-responsive lincRNAs in *Populus trichocarpa*. J. Exp. Bot. 65, 4975–4983. 10.1093/jxb/eru256 24948679 PMC4144774

[B70] Shumayla Sharma, S. Taneja M. Tyagi S. Singh K. Upadhyay S. K. (2017). Survey of high throughput RNA-seq data reveals potential roles for lncRNAs during development and stress response in bread wheat. Front. Plant Sci. 8:1019. 10.3389/fpls.2017.01019 28649263 PMC5465302

[B71] Simon E. W. (1978). The symptoms of calcium deficiency in plants. New Phytol. 80, 1–15. 10.1111/j.1469-8137.1978.tb02259.x

[B72] Strzalka W. Ziemienowicz A. (2011). Proliferating cell nuclear antigen (PCNA): a key factor in DNA replication and cell cycle regulation. Ann. Bot. 107, 1127–1140. 10.1093/aob/mcq243 21169293 PMC3091797

[B73] Sunkar R. Chinnusamy V. Zhu J. Zhu J. K. (2007). Small RNAs as big players in plant abiotic stress responses and nutrient deprivation. Trends Plant Sci. 12, 301–309. 10.1016/j.tplants.2007.05.001 17573231

[B74] Swiezewski S. Liu F. Magusin A. Dean C. (2009). Cold-induced silencing by long antisense transcripts of an *Arabidopsis* Polycomb target. Nature 462, 799–802. 10.1038/nature08618 20010688

[B75] Takata M. Pickard W. F. Lettvin J. Y. Moore J. W. (1966). Ionic conductance changes in lobster axon membrane when lanthanum is substituted for calcium. J. Gen. Physiol. 50, 461–471. 10.1085/jgp.50.2.461 11526840 PMC2225656

[B76] Tan S. Han R. Li P. Yang G. Li S. Zhang P.. (2015). Over-expression of the *MxIRT1* gene increases iron and zinc content in rice seeds. Transgenic Res. 24, 109–122. 10.1007/s11248-014-9822-z 25099285

[B77] Trapnell C. Roberts A. Goff L. Pertea G. Kim D. Kelley D. R.. (2012). Differential gene and transcript expression analysis of RNA-seq experiments with TopHat and Cufflinks. Nat. Protoc. 7, 562–578. 10.1038/nprot.2012.016 22383036 PMC3334321

[B78] Trapnell C. Williams B. A. Pertea G. Mortazavi A. Kwan G. van Baren M. J.. (2010). Transcript assembly and quantification by RNA-seq reveals unannotated transcripts and isoform switching during cell differentiation. Nat. Biotechnol. 28, 511–515. 10.1038/nbt.1621 20436464 PMC3146043

[B79] Wang L. G, Wang, S. Q. Li W. (2012). RSeQC: quality control of RNA-seq experiments. Bioinformatics 28, 2184–2185. 10.1093/bioinformatics/bts356 22743226

[B80] Wang M. Ruan Y. Chen Q. Li S. Wang Q. Cai Y. (2011). Curcumin induced HepG2 cell apoptosis-associated mitochondrial membrane potential and intracellular free Ca^2+^ concentration. Eur. J. Pharm. Sci. 650, 41–47. 10.1016/j.ejphar.2010.09.049 20883687

[B81] Wang P. Xue Y. Han Y. Lin L. Wu C. Xu S.. (2014). The STAT3-binding long noncoding RNA Lnc-DC controls human dendritic cell differentiation. Science 344, 310–313. 10.1126/science.1251456 24744378

[B82] Wang T. Z. Liu M. Zhao M. G. Chen R. Zhang W. H. (2015). Identification and characterization of long non-coding RNAs involved in osmotic and salt stress in *Medicago truncatula* using genome-wide high-throughput sequencing. BMC Plant Biol. 15:131. 10.1186/s12870-015-0530-5 26048392 PMC4457090

[B83] Wang Y. Fan X. Lin F. He G. Terzaghi W. Zhu D.. (2014). *Arabidopsis* noncoding RNA mediates control of photomorphogenesis by red light. Proc. Natl. Acad. Sci. U.S.A. 111, 10359–10364. 10.1073/pnas.1409457111 24982146 PMC4104870

[B84] White P. J. (2000). Calcium channels in higher plants. Biochim. Biophys. Acta 1465, 171–189. 10.1016/S0005-2736(00)00137-1 10748253

[B85] Xin M. Wang Y. Yao Y. Song N. Hu Z. Qin D.. (2011). Identification and characterization of wheat long non-protein coding RNAs responsive to powdery mildew infection and heat stress by using microarray analysis and SBS sequencing. BMC Plant Biol. 11:61. 10.1186/1471-2229-11-61 21473757 PMC3079642

[B86] Xu X. W. Zhou X. H. Wang R. R. Peng W. L. An Y. Chen L. L. (2016). Functional analysis of long intergenic non-coding RNAs in phosphate-starved rice using competing endogenous RNA network. Sci. Rep. 6:20715. 10.1038/srep20715 26860696 PMC4748279

[B87] Yang C. Zhao L. Zhang H. Yang Z. Wang H. Wen S.. (2014). Evolution of physiological responses to salt stress in hexaploid wheat. Proc. Natl. Acad. Sci. U.S.A. 111, 11882–11887. 10.1073/pnas.1412839111 25074914 PMC4136619

[B88] Zhang C. Y. Shi W. S. Ma K. S. Li H. J. Zhang F. X. (2016). EGTA, a calcium chelator, affects cell cycle and increases DNA methylation in root tips of *Triticum aestivum* L. Acta Soc. Bot. Pol. 85:3502 10.5586/asbp.3502

[B89] Zhang X. Tang N. Hadden T. J. Rishi A. K. (2011). Akt, FoxO and regulation of apoptosis. Biochim. Biophys. Acta 1813, 1978–1986. 10.1016/j.bbamcr.2011.03.010 21440011

[B90] Zhang Y. C. Liao J. Y. Li Z. Y. Yu Y. Zhang J. P. Li Q. F.. (2014). Genome-wide screening and functional analysis identify a large number of long noncoding RNAs involved in the sexual reproduction of rice. Genome Biol. 15:512. 10.1186/s13059-014-0512-1 25517485 PMC4253996

