# Supplementary material for: Genome-Wide Identification and Characterization of Long Non-Coding RNA in Wheat Roots in Response to Ca2+ Channel Blocker
Source: Front Plant Sci. 2018 Mar 6;9:244. doi: 10.3389/fpls.2018.00244 (PMC5845709; doi:10.3389/fpls.2018.00244)
Supplement: Supplementary file 1 [file Table1.docx]

**Supplementary information 2: Tables**

**Genome-wide identification and characterization of long non-coding RNA in wheat roots in response to Ca^2+^ channel blocker**

Keshi Ma^1, 2^, Wenshuo Shi^1^, Mengyue Xu^1^, Jiaxi Liu^1^ & Feixiong Zhang^1^

^1^College of Life Sciences, Capital Normal University, Beijing 100048, China. ^2^College of Life Science and Agronomy, Zhoukou Normal University, Zhoukou 466001, China. Correspondence and requests for materials should be addressed to F.Z. (email: fxzhang@cnu.edu.cn) or J.L. (email: liu-jiaxi@263.net)

Table S1. The mapping statistics of the RNA-seq data

| Sample | Total Reads | Total Mapped | Multiple Mapped | Uniquely Mapped |
| --- | --- | --- | --- | --- |
| CK1 | 47481026 | 27419139(57.75%) | 5129422(10.80%) | 22289717(46.94%) |
| CK2 | 37700260 | 21726303(57.63%) | 4156840(11.03%) | 17569463(46.60%) |
| CK3 | 42003366 | 21894255(52.12%) | 3962780(9.43%) 1 | 7931475(42.69%) |
| T1 | 46305586 | 27160924(58.66%) | 6190701(13.37%) | 20970223(45.29%) |
| T2 | 37500424 | 22909638(61.09%) | 4965355(13.24%) | 17944283(47.85%) |
| T3 | 41555156 | 25301450(60.89%) | 6979362(16.80%) | 18322088(44.09%) |

Table S2. The general information of 177 differentially expressed lncRNA

| **lncRNA name** | **class** | **locus** | **sample 1** | **sample 2** | **status** | **value_1** | **value_2** | **log2(T/CK)** | **p_value** | **q_value** | **significant** |
| --- | --- | --- | --- | --- | --- | --- | --- | --- | --- | --- | --- |
| *lncRNA_083139* | sense | IWGSC_CSS_7DL_scaff_3366267:812-1253 | CK | T | OK | 0 | 0.893175 | infinity | 5.00E-05 | 0.001906 | yes |
| *lncRNA_073681* | sense | IWGSC_CSS_5AS_scaff_1541139:6872-8634 | CK | T | OK | 0 | 0.949795 | infinity | 5.00E-05 | 0.001906 | yes |
| *lncRNA_054824* | sense | 7D:118905103-118905864 | CK | T | OK | 0 | 1.95181 | infinity | 5.00E-05 | 0.001906 | yes |
| *lncRNA_048320* | sense | 7A:17019460-17020208 | CK | T | OK | 0 | 5.52606 | infinity | 5.00E-05 | 0.001906 | yes |
| *lncRNA_039837* | sense | 5D:15899549-15902979 | CK | T | OK | 0 | 3.42195 | infinity | 0.00015 | 0.004877 | yes |
| *lncRNA_039748* | sense | 5D:27678-29921 | CK | T | OK | 0 | 0.536789 | infinity | 5.00E-05 | 0.001906 | yes |
| *lncRNA_031968* | sense | 4D:39767708-39768123 | CK | T | OK | 0 | 0.891849 | infinity | 5.00E-05 | 0.001906 | yes |
| *lncRNA_020939* | sense | 3B:750694247-750694813 | CK | T | OK | 0 | 0.615159 | infinity | 5.00E-05 | 0.001906 | yes |
| *lncRNA_019748* | sense | 3B:127500589-127500999 | CK | T | OK | 0 | 0.593182 | infinity | 0.0002 | 0.006157 | yes |
| *lncRNA_016209* | sense | 2D:98062076-98063318 | CK | T | OK | 0 | 1.37134 | infinity | 5.00E-05 | 0.001906 | yes |
| *lncRNA_008510* | sense | 2A:230227467-230227680 | CK | T | OK | 0 | 6.21655 | infinity | 0.0003 | 0.008542 | yes |
| *lncRNA_003448* | sense | 1B:261600851-261601175 | CK | T | OK | 0 | 2.25307 | infinity | 5.00E-05 | 0.001906 | yes |
| *lncRNA_039803* | sense | 5D:7337814-7386098 | CK | T | OK | 124.35 | 2952.21 | 4.56931 | 5.00E-05 | 0.001906 | yes |
| *lncRNA_044556* | sense | 6B:36403548-36403923 | CK | T | OK | 260.668 | 3340.85 | 3.69303 | 0.00025 | 0.007391 | yes |
| *lncRNA_082069* | sense | IWGSC_CSS_7BL_scaff_6687438:4041-10337 | CK | T | OK | 0.129596 | 1.51658 | 3.54873 | 0.0012 | 0.026009 | yes |
| *lncRNA_056853* | sense | IWGSC_CSS_1AL_scaff_3975510:2332-2639 | CK | T | OK | 4.393 | 34.1511 | 2.95865 | 5.00E-05 | 0.001906 | yes |
| *lncRNA_054317* | sense | 7D:15966752-15968695 | CK | T | OK | 3.67911 | 25.3767 | 2.78607 | 5.00E-05 | 0.001906 | yes |
| *lncRNA_014501* | sense | 2D:25140392-25384921 | CK | T | OK | 519.366 | 3549.79 | 2.77291 | 5.00E-05 | 0.001906 | yes |
| *lncRNA_011366* | sense | 2B:211869121-211869368 | CK | T | OK | 8.5634 | 45.6072 | 2.41301 | 5.00E-05 | 0.001906 | yes |
| *lncRNA_009127* | sense | 2A:79314392-79315062 | CK | T | OK | 1.11699 | 2.72993 | 2.26291 | 0.0028 | 0.048378 | yes |
| *lncRNA_047461* | sense | 6D:171673293-171673674 | CK | T | OK | 5.01354 | 23.9427 | 2.25569 | 5.00E-05 | 0.001906 | yes |
| *lncRNA_026968* | sense | 4A:165276644-165278506 | CK | T | OK | 0.598522 | 2.42534 | 2.01871 | 0.00025 | 0.007391 | yes |
| *lncRNA_032429* | sense | 4D:12690-12932 | CK | T | OK | 7.20055 | 28.9074 | 2.00526 | 0.0001 | 0.003498 | yes |
| *lncRNA_007700* | sense | 2A:35548507-35553868 | CK | T | OK | 0.520316 | 2.0711 | 1.99294 | 5.00E-05 | 0.001906 | yes |
| *lncRNA_063009* | sense | IWGSC_CSS_2DL_scaff_9764161:1626-1944 | CK | T | OK | 9.51951 | 34.1289 | 1.84203 | 5.00E-05 | 0.001906 | yes |
| *lncRNA_059554* | sense | IWGSC_CSS_2AL_scaff_3760763:3211-3648 | CK | T | OK | 1.74008 | 6.09196 | 1.80775 | 0.0023 | 0.042071 | yes |
| *lncRNA_012242* | sense | 2B:60006613-60009871 | CK | T | OK | 7.65444 | 25.8249 | 1.75439 | 5.00E-05 | 0.001906 | yes |
| *lncRNA_063547* | sense | IWGSC_CSS_2DL_scaff_9904759:145-3847 | CK | T | OK | 1.74274 | 5.4823 | 1.65342 | 5.00E-05 | 0.001906 | yes |
| *lncRNA_027360* | sense | 4A:25099116-25103001 | CK | T | OK | 0.632026 | 1.78832 | 1.50055 | 0.00065 | 0.015955 | yes |
| *lncRNA_020959* | sense | 3B:756898382-756902503 | CK | T | OK | 4.83969 | 13.2227 | 1.45003 | 5.00E-05 | 0.001906 | yes |
| *lncRNA_014639* | sense | 2D:42595401-42609621 | CK | T | OK | 3.25294 | 8.54841 | 1.39391 | 0.0013 | 0.027596 | yes |
| *lncRNA_014504* | sense | 2D:25440917-25442781 | CK | T | OK | 5.00749 | 12.448 | 1.31375 | 5.00E-05 | 0.001906 | yes |
| *lncRNA_054804* | sense | 7D:113002697-113010188 | CK | T | OK | 0.631279 | 1.53237 | 1.27941 | 0.0013 | 0.027596 | yes |
| *lncRNA_053891* | sense | 7D:188338486-188346339 | CK | T | OK | 1.17281 | 2.82425 | 1.2679 | 5.00E-05 | 0.001906 | yes |
| *lncRNA_071124* | sense | IWGSC_CSS_4DL_scaff_14448424:5331-6158 | CK | T | OK | 6.40979 | 14.6311 | 1.19069 | 5.00E-05 | 0.001906 | yes |
| *lncRNA_039325* | sense | 5D:119596961-119600165 | CK | T | OK | 3.86431 | 8.75638 | 1.18013 | 5.00E-05 | 0.001906 | yes |
| *lncRNA_007944* | sense | 2A:102641063-102642297 | CK | T | OK | 6.85307 | 9.26496 | 1.10549 | 0.0009 | 0.02085 | yes |
| *lncRNA_029528* | sense | 4B:281409580-281412468 | CK | T | OK | 0.693597 | 1.4856 | 1.09887 | 0.00175 | 0.03428 | yes |
| *lncRNA_080683* | sense | IWGSC_CSS_7AL_scaff_4550052:2438-5075 | CK | T | OK | 1.7502 | 3.659 | 1.06393 | 0.00275 | 0.047678 | yes |
| *lncRNA_017751* | sense | 3A:105831736-105834047 | CK | T | OK | 3.82882 | 7.8727 | 1.03996 | 0.0001 | 0.003498 | yes |
| *lncRNA_025391* | sense | 3D:78129114-78131289 | CK | T | OK | 3.29123 | 6.71372 | 1.02849 | 0.0002 | 0.006157 | yes |
| *lncRNA_039319* | sense | 5D:118563091-118566918 | CK | T | OK | 5.46441 | 10.8802 | 0.993562 | 5.00E-05 | 0.001906 | yes |
| *lncRNA_077026* | sense | IWGSC_CSS_6AL_scaff_5711760:0-254 | CK | T | OK | 27.4102 | 52.7464 | 0.944359 | 0.00245 | 0.043711 | yes |
| *lncRNA_044308* | sense | 6B:180653824-180656273 | CK | T | OK | 3.62761 | 6.76342 | 0.898735 | 0.00095 | 0.021764 | yes |
| *lncRNA_017776* | sense | 3A:115181720-115187076 | CK | T | OK | 0.702611 | 1.28924 | 0.875726 | 0.0018 | 0.035038 | yes |
| *lncRNA_084019* | sense | IWGSC_CSS_7DS_scaff_568735:0-4688 | CK | T | OK | 1.93347 | 3.42376 | 0.824391 | 0.0008 | 0.018909 | yes |
| *lncRNA_018919* | sense | 3A:182696641-182708211 | CK | T | OK | 1.8297 | 3.15174 | 0.784544 | 0.00235 | 0.042388 | yes |
| *lncRNA_048256* | sense | 7A:6706811-6710329 | CK | T | OK | 23.2523 | 39.3252 | 0.758081 | 0.0001 | 0.003498 | yes |
| *lncRNA_001839* | sense | 1A:217768607-217769629 | CK | T | OK | 9.38869 | 15.5145 | 0.724622 | 0.0006 | 0.014923 | yes |
| *TRAES3BF053100050CFD_g* | Pseudogene | 3B:402491635-402493288 | CK | T | OK | 0.158445 | 4.43931 | 4.80829 | 0.0001 | 0.003498 | yes |
| *TRAES3BF052700300CFD_g* | Pseudogene | 3B:393258638-393259404 | CK | T | OK | 3.78803 | 9.67182 | 1.35234 | 5.00E-05 | 0.001906 | yes |
| *Traes_1BS_B58657408* | Pseudogene | 1B:62930307-62935485 | CK | T | OK | 1.74641 | 3.03971 | 0.799544 | 0.0006 | 0.014923 | yes |
| *lncRNA_009362* | intonic | 2A:152624900-152629047 | CK | T | OK | 1.11386 | 2.59839 | 1.22204 | 0.0026 | 0.045698 | yes |
| *lncRNA_030381* | intergenetic | 4B:213988585-213988995 | CK | T | OK | 0 | 2.44433 | inf | 0.0004 | 0.010804 | yes |
| *lncRNA_027203* | intergenetic | 4A:207888297-207888780 | CK | T | OK | 0 | 8.69369 | inf | 5.00E-05 | 0.001906 | yes |
| *lncRNA_025607* | intergenetic | 3D:107172585-107172920 | CK | T | OK | 0 | 6.42494 | inf | 5.00E-05 | 0.001906 | yes |
| *lncRNA_014373* | intergenetic | 2D:10786544-10787014 | CK | T | OK | 2.20675 | 136.588 | 5.95177 | 5.00E-05 | 0.001906 | yes |
| *lncRNA_013190* | intergenetic | 2B:301669555-301672559 | CK | T | OK | 0.175172 | 2.38161 | 3.7651 | 5.00E-05 | 0.001906 | yes |
| *lncRNA_009698* | intergenetic | 2A:232020002-232024585 | CK | T | OK | 0.139336 | 1.83758 | 3.72116 | 5.00E-05 | 0.001906 | yes |
| *lncRNA_004117* | intergenetic | 1B:155808412-155808867 | CK | T | OK | 4.72606 | 43.6779 | 3.20819 | 5.00E-05 | 0.001906 | yes |
| *lncRNA_061738* | intergenetic | IWGSC_CSS_2BL_scaff_8062645:3991-5001 | CK | T | OK | 0.684622 | 6.0723 | 3.14886 | 5.00E-05 | 0.001906 | yes |
| *lncRNA_013231* | intergenetic | 2B:310631387-310633730 | CK | T | OK | 0.271495 | 1.92864 | 2.82859 | 5.00E-05 | 0.001906 | yes |
| *lncRNA_015487* | intergenetic | 2D:4602726-4604106 | CK | T | OK | 0.885078 | 5.21604 | 2.55908 | 0.00035 | 0.009688 | yes |
| *lncRNA_039289* | intergenetic | 5D:115948161-115952401 | CK | T | OK | 0.215785 | 1.22524 | 2.50539 | 5.00E-05 | 0.001906 | yes |
| *lncRNA_043877* | intergenetic | 6B:37593673-37597082 | CK | T | OK | 0.50354 | 2.81503 | 2.48297 | 5.00E-05 | 0.001906 | yes |
| *lncRNA_021433* | intergenetic | 3B:177328428-177342450 | CK | T | OK | 1.20619 | 6.62135 | 2.45667 | 0.00155 | 0.031411 | yes |
| *lncRNA_024807* | intergenetic | 3D:118354059-118355040 | CK | T | OK | 9.90947 | 53.9938 | 2.44591 | 5.00E-05 | 0.001906 | yes |
| *lncRNA_051318* | intergenetic | 7B:8501731-8504766 | CK | T | OK | 0.755308 | 3.76559 | 2.31774 | 5.00E-05 | 0.001906 | yes |
| *lncRNA_034367* | intergenetic | 5A:74492005-74495535 | CK | T | OK | 0.588153 | 2.63651 | 2.16437 | 5.00E-05 | 0.001906 | yes |
| *lncRNA_006270* | intergenetic | 1D:7553053-7554025 | CK | T | OK | 0.740784 | 3.1537 | 2.08992 | 0.0001 | 0.003498 | yes |
| *lncRNA_082364* | intergenetic | IWGSC_CSS_7BL_scaff_6744266:2212-3128 | CK | T | OK | 2.44682 | 10.4156 | 2.08977 | 5.00E-05 | 0.001906 | yes |
| *lncRNA_018036* | intergenetic | 3A:163132945-163134615 | CK | T | OK | 0.576003 | 2.43072 | 2.07724 | 0.00025 | 0.007391 | yes |
| *lncRNA_078349* | intergenetic | IWGSC_CSS_6BL_scaff_4351052:3373-5532 | CK | T | OK | 0.928199 | 3.64808 | 1.97463 | 0.00045 | 0.011883 | yes |
| *lncRNA_049461* | intergenetic | 7A:83320324-83323121 | CK | T | OK | 0.43518 | 1.67829 | 1.94731 | 0.00125 | 0.026775 | yes |
| *lncRNA_080648* | intergenetic | IWGSC_CSS_7AL_scaff_4535622:4207-5296 | CK | T | OK | 9.92722 | 37.4285 | 1.91467 | 5.00E-05 | 0.001906 | yes |
| *lncRNA_059976* | intergenetic | IWGSC_CSS_2AL_scaff_6361721:3416-7004 | CK | T | OK | 0.553252 | 2.04069 | 1.88305 | 0.00265 | 0.046425 | yes |
| *lncRNA_022554* | intergenetic | 3B:755949099-755952684 | CK | T | OK | 0.308073 | 1.1296 | 1.87447 | 5.00E-05 | 0.001906 | yes |
| *lncRNA_032287* | intergenetic | 4D:104893081-104893524 | CK | T | OK | 2.45287 | 8.21171 | 1.74321 | 0.00025 | 0.007391 | yes |
| *lncRNA_074658* | intergenetic | IWGSC_CSS_5BL_scaff_10892062:1651-3092 | CK | T | OK | 1.88668 | 5.94455 | 1.65571 | 5.00E-05 | 0.001906 | yes |
| *lncRNA_005462* | intergenetic | 1D:1504055-1508494 | CK | T | OK | 0.454704 | 1.42991 | 1.65292 | 0.0006 | 0.014923 | yes |
| *lncRNA_072167* | intergenetic | IWGSC_CSS_5AL_scaff_2035398:0-541 | CK | T | OK | 1.85465 | 5.58623 | 1.59073 | 0.00215 | 0.040029 | yes |
| *lncRNA_064639* | intergenetic | IWGSC_CSS_3AL_scaff_4246747:2-1945 | CK | T | OK | 2.31509 | 6.94277 | 1.58444 | 5.00E-05 | 0.001906 | yes |
| *lncRNA_081520* | intergenetic | IWGSC_CSS_7AS_scaff_4250944:9058-11223 | CK | T | OK | 0.508791 | 1.49166 | 1.55178 | 0.0028 | 0.048378 | yes |
| *lncRNA_048266* | intergenetic | 7A:7476084-7479290 | CK | T | OK | 1.5784 | 4.61256 | 1.5471 | 5.00E-05 | 0.001906 | yes |
| *lncRNA_043148* | intergenetic | 6A:196379207-196382890 | CK | T | OK | 0.602973 | 1.75633 | 1.5424 | 0.0023 | 0.042071 | yes |
| *lncRNA_008784* | intergenetic | 2A:4798734-4800271 | CK | T | OK | 0.753026 | 2.17556 | 1.53062 | 0.00045 | 0.011883 | yes |
| *lncRNA_018078* | intergenetic | 3A:171496246-171497172 | CK | T | OK | 57.0173 | 150.657 | 1.40179 | 5.00E-05 | 0.001906 | yes |
| *lncRNA_006361* | intergenetic | 1D:31337837-31340685 | CK | T | OK | 0.404207 | 1.02578 | 1.34356 | 0.00235 | 0.042388 | yes |
| *lncRNA_051888* | intergenetic | 7B:190775722-190778512 | CK | T | OK | 1.15313 | 2.82035 | 1.29033 | 0.00015 | 0.004877 | yes |
| *lncRNA_029013* | intergenetic | 4B:110411992-110415527 | CK | T | OK | 1.07202 | 2.61493 | 1.28644 | 5.00E-05 | 0.001906 | yes |
| *lncRNA_018111* | intergenetic | 3A:179057334-179222831 | CK | T | OK | 13.6554 | 32.8955 | 1.26842 | 5.00E-05 | 0.001906 | yes |
| *lncRNA_057390* | intergenetic | IWGSC_CSS_1BL_scaff_3805710:3577-7441 | CK | T | OK | 0.421066 | 0.99194 | 1.23621 | 0.0001 | 0.003498 | yes |
| *lncRNA_072748* | intergenetic | IWGSC_CSS_5AL_scaff_2776037:715-3705 | CK | T | OK | 1.60629 | 3.72971 | 1.21533 | 0.00035 | 0.009688 | yes |
| *lncRNA_071008* | intergenetic | IWGSC_CSS_4DL_scaff_14424661:5722-8879 | CK | T | OK | 2.0992 | 4.86432 | 1.2124 | 0.0002 | 0.006157 | yes |
| *lncRNA_019725* | intergenetic | 3B:117474302-117477801 | CK | T | OK | 1.35803 | 3.11515 | 1.19778 | 5.00E-05 | 0.001906 | yes |
| *lncRNA_006085* | intergenetic | 1D:121237822-121238249 | CK | T | OK | 33.7961 | 75.4468 | 1.1586 | 5.00E-05 | 0.001906 | yes |
| *lncRNA_026943* | intergenetic | 4A:160955740-160959223 | CK | T | OK | 1.60759 | 3.5254 | 1.13288 | 0.0002 | 0.006157 | yes |
| *lncRNA_083353* | intergenetic | IWGSC_CSS_7DS_scaff_158263:0-2431 | CK | T | OK | 17.9788 | 38.3194 | 1.09178 | 0.00275 | 0.047678 | yes |
| *lncRNA_059323* | intergenetic | IWGSC_CSS_1DS_scaff_1911306:3165-8109 | CK | T | OK | 1.72933 | 3.64613 | 1.07615 | 0.00105 | 0.023512 | yes |
| *lncRNA_008977* | intergenetic | 2A:43623104-43626665 | CK | T | OK | 0.498576 | 1.04294 | 1.06478 | 0.00135 | 0.02827 | yes |
| *lncRNA_069396* | intergenetic | IWGSC_CSS_4BL_scaff_6961717:547-3118 | CK | T | OK | 3.5769 | 7.41613 | 1.05196 | 5.00E-05 | 0.001906 | yes |
| *lncRNA_033754* | intergenetic | 5A:75206485-75211160 | CK | T | OK | 0.458358 | 0.875069 | 0.932921 | 0.0027 | 0.047075 | yes |
| *lncRNA_037690* | intergenetic | 5B:244269851-244271437 | CK | T | OK | 2.69671 | 5.00718 | 0.8928 | 0.00065 | 0.015955 | yes |
| *lncRNA_039420* | intergenetic | 5D:128456270-128460191 | CK | T | OK | 1.21387 | 2.2041 | 0.860573 | 0.00285 | 0.049073 | yes |
| *lncRNA_059207* | intergenetic | IWGSC_CSS_1DS_scaff_1896996:4753-5429 | CK | T | OK | 11.549 | 20.5328 | 0.830168 | 0.0002 | 0.006157 | yes |
| *lncRNA_024152* | intergenetic | 3D:10528450-10532782 | CK | T | OK | 7.4798 | 12.5544 | 0.747116 | 0.00165 | 0.032906 | yes |
| *lncRNA_054399* | intergenetic | 7D:27107466-27113170 | CK | T | OK | 7.64388 | 11.5037 | 0.589727 | 0.0026 | 0.045698 | yes |
| *lncRNA_053211* | antisense | 7D:23763160-23865432 | CK | T | OK | 618.675 | 23239.2 | 5.23124 | 5.00E-05 | 0.001906 | yes |
| *lncRNA_001557* | sense | 1A:136823958-136824640 | CK | T | OK | 55.8005 | 34.7252 | -0.684295 | 0.0004 | 0.010804 | yes |
| *lncRNA_005569* | sense | 1D:17735992-17739668 | CK | T | OK | 14.2956 | 8.70258 | -0.716053 | 0.00075 | 0.017885 | yes |
| *lncRNA_027894* | sense | 4A:158240677-158241260 | CK | T | OK | 9.71921 | 5.48496 | -0.825359 | 0.00045 | 0.011883 | yes |
| *lncRNA_051551* | sense | 7B:82863027-82867469 | CK | T | OK | 4.0599 | 2.2143 | -0.874595 | 0.00045 | 0.011883 | yes |
| *lncRNA_010675* | sense | 2B:31027703-31028088 | CK | T | OK | 19.0678 | 10.3753 | -0.877986 | 5.00E-05 | 0.001906 | yes |
| *lncRNA_039943* | sense | 5D:38416237-38425948 | CK | T | OK | 11.5179 | 6.18292 | -0.897517 | 5.00E-05 | 0.001906 | yes |
| *lncRNA_062306* | sense | IWGSC_CSS_2BS_scaff_5215259:393-4625 | CK | T | OK | 4.38768 | 2.32229 | -0.91791 | 0.00135 | 0.02827 | yes |
| *lncRNA_017777* | sense | 3A:116544301-116546074 | CK | T | OK | 5.25634 | 2.76448 | -0.927052 | 0.0003 | 0.008542 | yes |
| *lncRNA_047317* | sense | 6D:152638294-152639857 | CK | T | OK | 4.06111 | 2.05319 | -0.984003 | 0.00125 | 0.026775 | yes |
| *lncRNA_000833* | sense | 1A:226502148-226502741 | CK | T | OK | 13.1062 | 6.61984 | -0.985382 | 0.00075 | 0.017885 | yes |
| *lncRNA_076127* | sense | IWGSC_CSS_5DL_scaff_4560347:5997-6641 | CK | T | OK | 13.8519 | 6.99263 | -0.986181 | 0.00045 | 0.011883 | yes |
| *lncRNA_044581* | sense | 6B:43157423-43159346 | CK | T | OK | 17.39 | 8.77299 | -0.987115 | 0.0002 | 0.006157 | yes |
| *lncRNA_072935* | sense | IWGSC_CSS_5AL_scaff_2799565:71-1901 | CK | T | OK | 5.11728 | 2.48786 | -1.04047 | 0.0001 | 0.003498 | yes |
| *lncRNA_042235* | sense | 6A:192110639-192115368 | CK | T | OK | 17.9214 | 8.68102 | -1.04574 | 5.00E-05 | 0.001906 | yes |
| *lncRNA_029384* | sense | 4B:248139069-248146445 | CK | T | OK | 1.82238 | 0.852514 | -1.09603 | 0.00085 | 0.019834 | yes |
| *lncRNA_070075* | sense | IWGSC_CSS_4BS_scaff_4912151:1673-1969 | CK | T | OK | 109.461 | 73.2471 | -1.1158 | 0.0013 | 0.027596 | yes |
| *lncRNA_030379* | sense | 4B:213898957-213902415 | CK | T | OK | 7.32611 | 3.35014 | -1.12882 | 0.0001 | 0.003498 | yes |
| *lncRNA_048825* | sense | 7A:139844329-139844597 | CK | T | OK | 298.022 | 133.511 | -1.15846 | 5.00E-05 | 0.001906 | yes |
| *lncRNA_048419* | sense | 7A:32442322-32443163 | CK | T | OK | 6.10225 | 2.42778 | -1.3297 | 0.00015 | 0.004877 | yes |
| *lncRNA_083996* | sense | IWGSC_CSS_7DS_scaff_3967532:1636-5441 | CK | T | OK | 2.6595 | 0.983292 | -1.43547 | 5.00E-05 | 0.001906 | yes |
| *lncRNA_074434* | sense | IWGSC_CSS_5BL_scaff_10847269:4239-4672 | CK | T | OK | 117.519 | 58.168 | -1.48578 | 5.00E-05 | 0.001906 | yes |
| *lncRNA_066190* | sense | IWGSC_CSS_3AS_scaff_639014:0-461 | CK | T | OK | 8.0809 | 2.78848 | -1.53504 | 0.0003 | 0.008542 | yes |
| *lncRNA_049669* | sense | 7A:153133956-153138231 | CK | T | OK | 14.395 | 4.93668 | -1.54395 | 0.0002 | 0.006157 | yes |
| *lncRNA_058136* | sense | IWGSC_CSS_1DL_scaff_1677028:995-5235 | CK | T | OK | 4.33789 | 1.48589 | -1.54567 | 5.00E-05 | 0.001906 | yes |
| *lncRNA_043943* | sense | 6B:61941486-61942237 | CK | T | OK | 4.62364 | 1.49375 | -1.63009 | 0.00095 | 0.021764 | yes |
| *lncRNA_049300* | sense | 7A:44239980-44240233 | CK | T | OK | 45.3588 | 14.3656 | -1.65876 | 0.0001 | 0.003498 | yes |
| *lncRNA_006859* | sense | 1D:132187037-132190175 | CK | T | OK | 2.44066 | 0.763086 | -1.67735 | 0.0002 | 0.006157 | yes |
| *lncRNA_068342* | sense | IWGSC_CSS_4AL_scaff_7139663:1565-1719 | CK | T | OK | 4.70412 | 1.46282 | -1.68517 | 0.0003 | 0.008542 | yes |
| *lncRNA_054623* | sense | 7D:74526095-74526485 | CK | T | OK | 38.1687 | 8.81926 | -2.11366 | 5.00E-05 | 0.001906 | yes |
| *lncRNA_046989* | sense | 6D:88236434-88241624 | CK | T | OK | 4.99687 | 0.880098 | -2.50529 | 5.00E-05 | 0.001906 | yes |
| *lncRNA_066381* | sense | IWGSC_CSS_3DL_scaff_5877113:1-2888 | CK | T | OK | 19.7579 | 2.11945 | -3.22067 | 0.0007 | 0.016924 | yes |
| *lncRNA_078106* | sense | IWGSC_CSS_6BL_scaff_4275922:1099-3399 | CK | T | OK | 0.873431 | 0 | infinitesimal | 5.00E-05 | 0.001906 | yes |
| *lncRNA_077407* | sense | IWGSC_CSS_6AL_scaff_5833929:526-4390 | CK | T | OK | 0.725653 | 0 | infinitesimal | 5.00E-05 | 0.001906 | yes |
| *lncRNA_061348* | sense | IWGSC_CSS_2BL_scaff_7967169:794-995 | CK | T | OK | 5.57867 | 0 | infinitesimal | 0.0005 | 0.012886 | yes |
| *lncRNA_057614* | sense | IWGSC_CSS_1BL_scaff_3865931:311-588 | CK | T | OK | 1.20703 | 0 | infinitesimal | 0.0016 | 0.032156 | yes |
| *lncRNA_054394* | sense | 7D:26104225-26104491 | CK | T | OK | 3.05443 | 0 | infinitesimal | 0.0005 | 0.012886 | yes |
| *lncRNA_053655* | sense | 7D:130918133-130922685 | CK | T | OK | 3.69712 | 0 | infinitesimal | 0.00015 | 0.004877 | yes |
| *lncRNA_051428* | sense | 7B:40588469-40588751 | CK | T | OK | 4.03491 | 0 | infinitesimal | 5.00E-05 | 0.001906 | yes |
| *lncRNA_045725* | sense | 6D:222566-222870 | CK | T | OK | 1.1809 | 0 | infinitesimal | 0.0016 | 0.032156 | yes |
| *lncRNA_008951* | sense | 2A:35564088-35564764 | CK | T | OK | 1.73992 | 0 | infinitesimal | 5.00E-05 | 0.001906 | yes |
| *lncRNA_001072* | sense | 1A:1859565-1859822 | CK | T | OK | 3.01309 | 0 | infinitesimal | 0.0005 | 0.012886 | yes |
| *TRAES3BF024700270CFD_g* | Pseudogene | 3B:452827978-452829274 | CK | T | OK | 7.01401 | 3.89609 | -0.848214 | 0.0022 | 0.040733 | yes |
| *TRAES3BF177500010CFD_g* | Pseudogene | 3B:736047623-736048721 | CK | T | OK | 6.44934 | 3.37779 | -0.933071 | 0.0012 | 0.026009 | yes |
| *TRAES3BF117100150CFD_g* | Pseudogene | 3B:623494381-623495710 | CK | T | OK | 7.88747 | 3.95495 | -0.995904 | 5.00E-05 | 0.001906 | yes |
| *TRAES3BF026700070CFD_g* | Pseudogene | 3B:622494034-622499317 | CK | T | OK | 6.17614 | 2.67164 | -1.20898 | 5.00E-05 | 0.001906 | yes |
| *TRAES3BF051200090CFD_g* | Pseudogene | 3B:608416091-608417593 | CK | T | OK | 1.81379 | 0.607175 | -1.57883 | 0.0009 | 0.02085 | yes |
| *TRAES3BF060500060CFD_g* | Pseudogene | 3B:448322124-448323729 | CK | T | OK | 1.02732 | 0.145357 | -2.82121 | 0.0015 | 0.030629 | yes |
| *TRAES3BF021800100CFD_g* | Pseudogene | 3B:592758528-592760234 | CK | T | OK | 0.513997 | 0 | infinitesimal | 5.00E-05 | 0.001906 | yes |
| *lncRNA_015322* | intergenetic | 2D:141526371-141527153 | CK | T | OK | 23.9508 | 15.1291 | -0.662754 | 0.00225 | 0.041383 | yes |
| *lncRNA_009293* | intergenetic | 2A:131210867-131213989 | CK | T | OK | 5.93497 | 3.48768 | -0.766972 | 0.0018 | 0.035038 | yes |
| *lncRNA_072440* | intergenetic | IWGSC_CSS_5AL_scaff_2731537:45-6231 | CK | T | OK | 1.59058 | 0.886764 | -0.842932 | 0.00175 | 0.03428 | yes |
| *lncRNA_063127* | intergenetic | IWGSC_CSS_2DL_scaff_9822084:1841-2717 | CK | T | OK | 8.51929 | 4.62805 | -0.880327 | 0.00165 | 0.032906 | yes |
| *lncRNA_029500* | intergenetic | 4B:274192038-274192918 | CK | T | OK | 6.2168 | 3.18547 | -0.964664 | 0.0017 | 0.033595 | yes |
| *lncRNA_000823* | intergenetic | 1A:224565164-224566779 | CK | T | OK | 80.1985 | 40.6474 | -0.980413 | 5.00E-05 | 0.001906 | yes |
| *lncRNA_032048* | intergenetic | 4D:54309610-54311395 | CK | T | OK | 5.59125 | 2.75556 | -1.02083 | 0.00075 | 0.017885 | yes |
| *lncRNA_048368* | intergenetic | 7A:20841666-20842872 | CK | T | OK | 5.36689 | 2.62791 | -1.03017 | 0.00035 | 0.009688 | yes |
| *lncRNA_020477* | intergenetic | 3B:547158398-547159302 | CK | T | OK | 21.8893 | 9.40113 | -1.21932 | 5.00E-05 | 0.001906 | yes |
| *lncRNA_014538* | intergenetic | 2D:29481118-29481718 | CK | T | OK | 12.009 | 5.02659 | -1.25646 | 0.0002 | 0.006157 | yes |
| *lncRNA_029088* | intergenetic | 4B:137338984-137341745 | CK | T | OK | 2.49231 | 1.00565 | -1.30935 | 0.00215 | 0.040029 | yes |
| *lncRNA_074837* | intergenetic | IWGSC_CSS_5BL_scaff_10919017:3074-3805 | CK | T | OK | 7.38926 | 2.74725 | -1.42744 | 5.00E-05 | 0.001906 | yes |
| *lncRNA_068995* | intergenetic | IWGSC_CSS_4AS_scaff_5991233:6097-7040 | CK | T | OK | 2.70258 | 0.99145 | -1.44673 | 0.0023 | 0.042071 | yes |
| *lncRNA_053766* | intergenetic | 7D:157181499-157182998 | CK | T | OK | 54.3006 | 19.2307 | -1.49756 | 5.00E-05 | 0.001906 | yes |
| *lncRNA_048668* | intergenetic | 7A:99974280-99976193 | CK | T | OK | 7.88282 | 2.54724 | -1.62978 | 5.00E-05 | 0.001906 | yes |
| *lncRNA_000928* | intergenetic | 1A:234748718-234758069 | CK | T | OK | 5.58912 | 1.46361 | -1.93309 | 0.0006 | 0.014923 | yes |
| *lncRNA_024812* | intergenetic | 3D:119356588-119357483 | CK | T | OK | 5.96374 | 1.03576 | -2.52553 | 5.00E-05 | 0.001906 | yes |
| *lncRNA_065747* | intergenetic | IWGSC_CSS_3AS_scaff_3320891:119-598 | CK | T | OK | 0.854122 | 0 | infinitesimal | 0.0016 | 0.032156 | yes |
| *lncRNA_065442* | intergenetic | IWGSC_CSS_3AS_scaff_1387517:7716-8231 | CK | T | OK | 1.07772 | 0 | infinitesimal | 0.00035 | 0.009688 | yes |
| *lncRNA_049366* | intergenetic | 7A:55248376-55248851 | CK | T | OK | 3.83453 | 0 | infinitesimal | 5.00E-05 | 0.001906 | yes |
| *lncRNA_024464* | intergenetic | 3D:61960534-61960881 | CK | T | OK | 2.15948 | 0 | infinitesimal | 0.00015 | 0.004877 | yes |

**Table S3.** Random selected lncRNA for q-PCR

| lncRNA | value_CK | value_T | log2(fold_change) | test_stat | up/down |
| --- | --- | --- | --- | --- | --- |
| lncRNA_013190 | 0.175172 | 2.38161 | 3.7651 | 3.60442 | up |
| lncRNA_039803 | 124.35 | 2952.21 | 4.56931 | 4.16702 | up |
| lncRNA_053211 | 618.675 | 23239.2 | 5.23124 | 3.43199 | up |
| lncRNA _014373 | 2.20675 | 136.588 | 5.95177 | 7.77176 | up |
| lncRNA _024812 | 5.96374 | 1.03576 | -2.52553 | -3.61029 | down |
| lncRNA _046989 | 4.99687 | 0.880098 | -2.50529 | -3.78964 | down |
| lncRNA _032897 | 2.34771 | 0.413971 | -2.50365 | -2.98809 | down |
| lncRNA _042235 | 17.9214 | 8.68102 | -1.04574 | -2.76862 | down |

**Table S4.** Primers of random selected lncRNA

| lncRNA | Seqence of primer |
| --- | --- |
| lncRNA _024812 f | AGGCACCGGTTTCAGCTT |
| lncRNA _024812 r | CCGTGATATGGCTACAACCGA |
| lncRNA _046989 f | CCTCATGGTGGATGACGGTAA |
| lncRNA _046989 r | TCGGGTTCTCCTTTCCTCGTA |
| lncRNA _032897 f | CGTGCTGGAACCCGTAGAG |
| lncRNA _032897 r | GAGGAGATGCGGAGCAAGG |
| lncRNA _042235 f | CCGTCAGTAGTTGGTACTCGG |
| lncRNA _042235 r | CAGAGTAGCAGGGTGTAGCAG |
| lncRNA _013190 f | TGCTCCCTATTTCTCTCCTTCATT |
| lncRNA _013190 r | TTCAACAGATGCACATCACCAC |
| lncRNA _039803 f | GCTGACGCAATGTGATTTCTGG |
| lncRNA _039803 r | CCGTTCCCTTGACTGTGGTT |
| lncRNA _053211f | AGGGCACCACCAGACATAGC |
| lncRNA _053211r | AGACAAATCGCTCCACCAACT |
| lncRNA _014373 f | CGCAAGGCTGAAACTTAACAGG |
| lncRNA _014373 r | CAGACAAATCGCTCCACCAA |

**Table S5.** The correlation of lncRNA-mRNA co-expression

| Source | Target | Correlation | P.value |
| --- | --- | --- | --- |
| lncRNA_059323 | TRAES3BF003100010CFD_g | 0.9969631 | 1.38E-05 |
| lncRNA_081387 | TRAES3BF007400070CFD_g | 0.9909719 | 0.0001219 |
| lncRNA_083996 | TRAES3BF009500080CFD_g | 0.9967073 | 1.62E-05 |
| lncRNA_001839 | TRAES3BF010700030CFD_g | -0.990999 | 0.0001212 |
| lncRNA_029088 | TRAES3BF012100010CFD_g | 0.9928495 | 7.65E-05 |
| TRAES3BF177500010CFD_g | TRAES3BF013000050CFD_g | 0.9904497 | 0.0001364 |
| lncRNA_001839 | TRAES3BF018400060CFD_g | -0.995567 | 2.94E-05 |
| lncRNA_048256 | TRAES3BF018900010CFD_g | 0.9914643 | 0.000109 |
| lncRNA_051318 | TRAES3BF018900010CFD_g | 0.993409 | 6.50E-05 |
| lncRNA_083996 | TRAES3BF019300210CFD_g | 0.994051 | 5.30E-05 |
| lncRNA_048668 | TRAES3BF020300040CFD_g | 0.9966815 | 1.65E-05 |
| lncRNA_007700 | TRAES3BF021100050CFD_g | 0.9926149 | 8.16E-05 |
| lncRNA_018078 | TRAES3BF021100050CFD_g | 0.9909411 | 0.0001227 |
| lncRNA_029013 | TRAES3BF021100050CFD_g | 0.9913219 | 0.0001126 |
| lncRNA_063127 | TRAES3BF021100050CFD_g | -0.991598 | 0.0001056 |
| lncRNA_063547 | TRAES3BF021100050CFD_g | 0.9909072 | 0.0001236 |
| lncRNA_009698 | TRAES3BF021600130CFD_g | 0.9906514 | 0.0001307 |
| lncRNA_034367 | TRAES3BF021600130CFD_g | 0.9981309 | 5.24E-06 |
| lncRNA_019725 | TRAES3BF021700010CFD_g | -0.991765 | 0.0001014 |
| lncRNA_012610 | TRAES3BF022100060CFD_g | -0.990169 | 0.0001445 |
| lncRNA_018111 | TRAES3BF022100060CFD_g | -0.99093 | 0.000123 |
| lncRNA_044308 | TRAES3BF022700030CFD_g | 0.9995931 | 2.48E-07 |
| lncRNA_008977 | TRAES3BF024300050CFD_g | 0.9903635 | 0.0001388 |
| lncRNA_058136 | TRAES3BF024700350CFD_g | 0.9919343 | 9.73E-05 |
| lncRNA_007700 | TRAES3BF025400010CFD_g | -0.99087 | 0.0001247 |
| lncRNA_059554 | TRAES3BF025400010CFD_g | -0.997412 | 1.00E-05 |
| lncRNA_015487 | TRAES3BF025700030CFD_g | 0.9909441 | 0.0001226 |
| lncRNA_068995 | TRAES3BF025700030CFD_g | -0.990586 | 0.0001325 |
| TRAES3BF052700300CFD_g | TRAES3BF026100050CFD_g | 0.9911453 | 0.0001173 |
| lncRNA_014501 | TRAES3BF026100050CFD_g | 0.998172 | 5.01E-06 |
| lncRNA_061738 | TRAES3BF026100050CFD_g | 0.9956599 | 2.82E-05 |
| lncRNA_009293 | TRAES3BF026200060CFD_g | 0.9924657 | 8.49E-05 |
| lncRNA_029013 | TRAES3BF026500060CFD_g | 0.9981166 | 5.32E-06 |
| lncRNA_063127 | TRAES3BF026500060CFD_g | -0.992855 | 7.64E-05 |
| lncRNA_063547 | TRAES3BF026500060CFD_g | 0.9907698 | 0.0001274 |
| lncRNA_039943 | TRAES3BF026700020CFD_g | 0.9933557 | 6.61E-05 |
| lncRNA_053766 | TRAES3BF026700020CFD_g | 0.9928195 | 7.72E-05 |
| TRAES3BF021800100CFD_g | TRAES3BF026700050CFD_g | 0.9903399 | 0.0001395 |
| lncRNA_045725 | TRAES3BF026700050CFD_g | 1 | 0 |
| lncRNA_051428 | TRAES3BF026700050CFD_g | 1 | 0 |
| lncRNA_057614 | TRAES3BF026700050CFD_g | 1 | 0 |
| lncRNA_065747 | TRAES3BF026700050CFD_g | 1 | 0 |
| Traes_1BS_B58657408 | TRAES3BF026900020CFD_g | -0.990797 | 0.0001267 |
| lncRNA_046989 | TRAES3BF027700220CFD_g | 0.9953415 | 3.25E-05 |
| lncRNA_039420 | TRAES3BF029600050CFD_g | -0.994135 | 5.15E-05 |
| lncRNA_074837 | TRAES3BF029600050CFD_g | 0.9950648 | 3.65E-05 |
| TRAES3BF052700300CFD_g | TRAES3BF031600050CFD_g | 0.9935169 | 6.29E-05 |
| lncRNA_014501 | TRAES3BF031600050CFD_g | 0.9911016 | 0.0001184 |
| lncRNA_047461 | TRAES3BF031600050CFD_g | 0.9954096 | 3.16E-05 |
| lncRNA_054317 | TRAES3BF032200010CFD_g | -0.992165 | 9.18E-05 |
| lncRNA_068995 | TRAES3BF033900100CFD_g | 0.9991867 | 9.92E-07 |
| lncRNA_039325 | TRAES3BF034900010CFD_g | 0.9939967 | 5.40E-05 |
| lncRNA_049366 | TRAES3BF036400010CFD_g | 0.9911324 | 0.0001176 |
| lncRNA_009698 | TRAES3BF038000030CFD_g | -0.995359 | 3.23E-05 |
| lncRNA_013190 | TRAES3BF038000030CFD_g | -0.995093 | 3.61E-05 |
| lncRNA_048256 | TRAES3BF038000030CFD_g | -0.997351 | 1.05E-05 |
| lncRNA_020959 | TRAES3BF042900030CFD_g | 0.9906381 | 0.0001311 |
| lncRNA_024807 | TRAES3BF042900030CFD_g | 0.9928741 | 7.60E-05 |
| lncRNA_061738 | TRAES3BF042900030CFD_g | 0.9910638 | 0.0001194 |
| lncRNA_019725 | TRAES3BF043700170CFD_g | -0.991087 | 0.0001188 |
| lncRNA_068995 | TRAES3BF044000030CFD_g | 0.998968 | 1.60E-06 |
| lncRNA_007700 | TRAES3BF045500260CFD_g | 0.9945911 | 4.38E-05 |
| lncRNA_018078 | TRAES3BF045500260CFD_g | 0.9946727 | 4.25E-05 |
| lncRNA_029013 | TRAES3BF045500260CFD_g | 0.9928201 | 7.71E-05 |
| lncRNA_063127 | TRAES3BF045500260CFD_g | -0.994217 | 5.01E-05 |
| lncRNA_054623 | TRAES3BF045900020CFD_g | 0.992975 | 7.39E-05 |
| lncRNA_006361 | TRAES3BF046300070CFD_g | 0.9919825 | 9.62E-05 |
| lncRNA_015487 | TRAES3BF046300070CFD_g | 0.9955375 | 2.98E-05 |
| lncRNA_051318 | TRAES3BF046300070CFD_g | 0.993387 | 6.55E-05 |
| lncRNA_029528 | TRAES3BF048900230CFD_g | -0.990579 | 0.0001327 |
| lncRNA_033754 | TRAES3BF049800030CFD_g | -0.992857 | 7.64E-05 |
| lncRNA_024152 | TRAES3BF049800160CFD_g | -0.991335 | 0.0001123 |
| lncRNA_054317 | TRAES3BF049800160CFD_g | -0.992433 | 8.57E-05 |
| lncRNA_083996 | TRAES3BF051200110CFD_g | 0.9923147 | 8.84E-05 |
| lncRNA_012610 | TRAES3BF051600120CFD_g | 0.9938183 | 5.72E-05 |
| lncRNA_015487 | TRAES3BF051600120CFD_g | 0.9920356 | 9.49E-05 |
| lncRNA_018111 | TRAES3BF051600120CFD_g | 0.9959577 | 2.45E-05 |
| lncRNA_051318 | TRAES3BF051600120CFD_g | 0.9912187 | 0.0001153 |
| lncRNA_024812 | TRAES3BF052300070CFD_g | 0.996074 | 2.31E-05 |
| Traes_1BS_B58657408 | TRAES3BF056900080CFD_g | -0.996853 | 1.48E-05 |
| TRAES3BF117100150CFD_g | TRAES3BF058500020CFD_g | -0.999647 | 1.87E-07 |
| lncRNA_048256 | TRAES3BF059200050CFD_g | 0.9923826 | 8.68E-05 |
| lncRNA_010675 | TRAES3BF060500310CFD_g | 0.9944505 | 4.61E-05 |
| lncRNA_063127 | TRAES3BF061700060CFD_g | -0.995341 | 3.25E-05 |
| lncRNA_064639 | TRAES3BF063000030CFD_g | 0.9978843 | 6.71E-06 |
| lncRNA_013053 | TRAES3BF064800030CFD_g | 0.993039 | 7.25E-05 |
| lncRNA_006361 | TRAES3BF065400180CFD_g | -0.99581 | 2.63E-05 |
| lncRNA_015487 | TRAES3BF065400180CFD_g | -0.991113 | 0.0001181 |
| lncRNA_018111 | TRAES3BF065400180CFD_g | -0.99215 | 9.22E-05 |
| lncRNA_014504 | TRAES3BF066100080CFD_g | 0.9954976 | 3.04E-05 |
| lncRNA_019725 | TRAES3BF066100080CFD_g | 0.9908436 | 0.0001254 |
| lncRNA_051318 | TRAES3BF066100080CFD_g | 0.9901913 | 0.0001438 |
| lncRNA_058136 | TRAES3BF066100080CFD_g | -0.990852 | 0.0001251 |
| lncRNA_063127 | TRAES3BF066100080CFD_g | -0.994153 | 5.12E-05 |
| lncRNA_013053 | TRAES3BF066100090CFD_g | 0.9904068 | 0.0001376 |
| lncRNA_051551 | TRAES3BF066100090CFD_g | 0.9901764 | 0.0001443 |
| lncRNA_044556 | TRAES3BF066400010CFD_g | 0.9954453 | 3.11E-05 |
| lncRNA_074658 | TRAES3BF066400010CFD_g | 0.9913996 | 0.0001106 |
| lncRNA_008500 | TRAES3BF067800230CFD_g | 0.9930787 | 7.17E-05 |
| TRAES3BF021800100CFD_g | TRAES3BF068400030CFD_g | 0.9946257 | 4.32E-05 |
| lncRNA_045725 | TRAES3BF068400030CFD_g | 0.9993739 | 5.88E-07 |
| lncRNA_051428 | TRAES3BF068400030CFD_g | 0.9993739 | 5.88E-07 |
| lncRNA_057614 | TRAES3BF068400030CFD_g | 0.9993739 | 5.88E-07 |
| lncRNA_065747 | TRAES3BF068400030CFD_g | 0.9993739 | 5.88E-07 |
| lncRNA_032048 | TRAES3BF069700040CFD_g | 0.9923204 | 8.82E-05 |
| TRAES3BF053100050CFD_g | TRAES3BF072400290CFD_g | 0.9971968 | 1.18E-05 |
| lncRNA_080648 | TRAES3BF072400290CFD_g | 0.9927228 | 7.92E-05 |
| lncRNA_015322 | TRAES3BF073300100CFD_g | -0.991218 | 0.0001153 |
| lncRNA_014501 | TRAES3BF073700180CFD_g | -0.994902 | 3.89E-05 |
| lncRNA_061738 | TRAES3BF073700180CFD_g | -0.992321 | 8.82E-05 |
| lncRNA_009293 | TRAES3BF074400080CFD_g | 0.9961119 | 2.26E-05 |
| lncRNA_006270 | TRAES3BF075200040CFD_g | 0.9905126 | 0.0001346 |
| lncRNA_044556 | TRAES3BF075200040CFD_g | 0.9915315 | 0.0001073 |
| lncRNA_013231 | TRAES3BF075200060CFD_g | -0.995917 | 2.50E-05 |
| lncRNA_064639 | TRAES3BF076900070CFD_g | -0.998299 | 4.34E-06 |
| lncRNA_037690 | TRAES3BF081300060CFD_g | 0.9944105 | 4.68E-05 |
| lncRNA_000928 | TRAES3BF082600160CFD_g | -0.991479 | 0.0001086 |
| lncRNA_009698 | TRAES3BF082600160CFD_g | 0.9977919 | 7.31E-06 |
| lncRNA_012610 | TRAES3BF082600160CFD_g | 0.9942822 | 4.89E-05 |
| lncRNA_013190 | TRAES3BF082600160CFD_g | 0.9922801 | 8.92E-05 |
| lncRNA_019725 | TRAES3BF082600160CFD_g | 0.9903531 | 0.0001391 |
| lncRNA_012608 | TRAES3BF085700020CFD_g | 0.9901816 | 0.0001441 |
| TRAES3BF024700270CFD_g | TRAES3BF086500030CFD_g | 0.9961299 | 2.24E-05 |
| lncRNA_012608 | TRAES3BF086500030CFD_g | 0.9919183 | 9.77E-05 |
| lncRNA_049300 | TRAES3BF086500030CFD_g | 0.9914824 | 0.0001085 |
| lncRNA_066381 | TRAES3BF088300010CFD_g | 0.9901765 | 0.0001443 |
| lncRNA_001557 | TRAES3BF091100240CFD_g | 0.9911739 | 0.0001165 |
| lncRNA_051318 | TRAES3BF091200050CFD_g | -0.99597 | 2.43E-05 |
| lncRNA_032897 | TRAES3BF092200020CFD_g | 0.9945284 | 4.48E-05 |
| lncRNA_051551 | TRAES3BF092200020CFD_g | 0.9952189 | 3.42E-05 |
| lncRNA_029500 | TRAES3BF092300040CFD_g | 0.9926976 | 7.98E-05 |
| TRAES3BF052700300CFD_g | TRAES3BF093000020CFD_g | -0.998512 | 3.32E-06 |
| lncRNA_054623 | TRAES3BF093600150CFD_g | 0.995982 | 2.42E-05 |
| lncRNA_066190 | TRAES3BF093600150CFD_g | 0.9939294 | 5.52E-05 |
| lncRNA_048256 | TRAES3BF096800120CFD_g | -0.996174 | 2.19E-05 |
| lncRNA_076127 | TRAES3BF098400070CFD_g | 0.9954919 | 3.04E-05 |
| lncRNA_048266 | TRAES3BF104600010CFD_g | -0.994708 | 4.19E-05 |
| lncRNA_001839 | TRAES3BF107900020CFD_g | 0.9920796 | 9.39E-05 |
| lncRNA_020477 | TRAES3BF108800070CFD_g | 0.9937231 | 5.90E-05 |
| lncRNA_047461 | TRAES3BF110000010CFD_g | -0.993344 | 6.63E-05 |
| Traes_1BS_B58657408 | TRAES3BF111700080CFD_g | 0.9974392 | 9.83E-06 |
| lncRNA_026968 | TRAES3BF112400020CFD_g | 0.9927813 | 7.80E-05 |
| lncRNA_029013 | TRAES3BF112400020CFD_g | 0.9914332 | 0.0001098 |
| lncRNA_063547 | TRAES3BF112400020CFD_g | 0.9968018 | 1.53E-05 |
| lncRNA_078349 | TRAES3BF112400020CFD_g | 0.9967503 | 1.58E-05 |
| Traes_1BS_B58657408 | TRAES3BF114000090CFD_g | -0.990499 | 0.000135 |
| lncRNA_029088 | TRAES3BF114300110CFD_g | 0.9918489 | 9.94E-05 |
| lncRNA_074837 | TRAES3BF116200010CFD_g | 0.9961166 | 2.26E-05 |
| lncRNA_029088 | TRAES3BF117100120CFD_g | 0.9957356 | 2.72E-05 |
| lncRNA_013053 | TRAES3BF133600020CFD_g | 0.9979949 | 6.03E-06 |
| lncRNA_014501 | TRAES3BF136200010CFD_g | -0.994607 | 4.36E-05 |
| lncRNA_047461 | TRAES3BF136200010CFD_g | -0.995863 | 2.56E-05 |
| lncRNA_068995 | TRAES3BF136200030CFD_g | 0.9907586 | 0.0001277 |
| lncRNA_074434 | TRAES3BF140800020CFD_g | 0.992394 | 8.66E-05 |
| lncRNA_020959 | TRAES3BF146000040CFD_g | -0.996072 | 2.31E-05 |
| lncRNA_024807 | TRAES3BF146000040CFD_g | -0.993326 | 6.67E-05 |
| lncRNA_044308 | TRAES3BF146000040CFD_g | -0.991267 | 0.0001141 |
| lncRNA_006361 | TRAES3BF151500010CFD_g | -0.995482 | 3.06E-05 |
| lncRNA_020959 | TRAES3BF154700110CFD_g | -0.993679 | 5.98E-05 |
| lncRNA_044308 | TRAES3BF154700110CFD_g | -0.990265 | 0.0001417 |
| lncRNA_014501 | TRAES3BF159100010CFD_g | 0.9961793 | 2.19E-05 |
| lncRNA_020959 | TRAES3BF159100010CFD_g | 0.990724 | 0.0001287 |
| lncRNA_024807 | TRAES3BF159100010CFD_g | 0.9963831 | 1.96E-05 |
| lncRNA_061738 | TRAES3BF159100010CFD_g | 0.9980064 | 5.96E-06 |
| TRAES3BF052700300CFD_g | TRAES3BF168400280CFD_g | 0.9908385 | 0.0001255 |
| lncRNA_014501 | TRAES3BF175100090CFD_g | -0.991971 | 9.64E-05 |
| lncRNA_024807 | TRAES3BF175100090CFD_g | -0.990279 | 0.0001413 |
| lncRNA_061738 | TRAES3BF175100090CFD_g | -0.992979 | 7.38E-05 |
| TRAES3BF053100050CFD_g | TRAES3BF245700010CFD_g | 0.9972805 | 1.11E-05 |
| lncRNA_018078 | TRAES3BF245700010CFD_g | 0.9949999 | 3.74E-05 |
| lncRNA_043877 | TRAES3BF245700010CFD_g | 0.999058 | 1.33E-06 |
| lncRNA_080648 | TRAES3BF245700010CFD_g | 0.9952234 | 3.42E-05 |
| lncRNA_044308 | TRAES3BF268900100CFD_g | 0.9924232 | 8.59E-05 |
| lncRNA_047461 | TRAES3BF271100040CFD_g | 0.9913561 | 0.0001118 |
| lncRNA_007700 | TRAES3BF273100010CFD_g | -0.994432 | 4.64E-05 |
| lncRNA_015487 | TRAES3BF276600020CFD_g | -0.994192 | 5.05E-05 |
| lncRNA_054317 | TRAES3BF276600020CFD_g | -0.996479 | 1.86E-05 |
| lncRNA_063009 | TRAES3BF276600020CFD_g | -0.996093 | 2.29E-05 |
| lncRNA_022554 | Traes_1AL_0458C8353 | -0.990545 | 0.0001337 |
| lncRNA_049300 | Traes_1AL_058AFDE7F | 0.9984868 | 3.43E-06 |
| lncRNA_063127 | Traes_1AL_0913A6035 | 0.9943697 | 4.75E-05 |
| lncRNA_048256 | Traes_1AL_0A80F5807 | -0.995524 | 3.00E-05 |
| lncRNA_056853 | Traes_1AL_0BDE975B6 | 0.9918611 | 9.91E-05 |
| lncRNA_006085 | Traes_1AL_101B391E1 | -0.99079 | 0.0001268 |
| lncRNA_054317 | Traes_1AL_14BFFD9EA | -0.994294 | 4.87E-05 |
| lncRNA_063009 | Traes_1AL_14BFFD9EA | -0.990289 | 0.000141 |
| lncRNA_018111 | Traes_1AL_151BF9143 | 0.9904207 | 0.0001372 |
| lncRNA_047461 | Traes_1AL_151BF9143 | 0.9957895 | 2.66E-05 |
| lncRNA_063009 | Traes_1AL_151BF9143 | 0.9915364 | 0.0001071 |
| lncRNA_020477 | Traes_1AL_16EAFFACD | 0.9905275 | 0.0001342 |
| lncRNA_048668 | Traes_1AL_16EAFFACD | 0.9904817 | 0.0001355 |
| lncRNA_048668 | Traes_1AL_1D02EEFB9 | 0.9909286 | 0.0001231 |
| lncRNA_011366 | Traes_1AL_1D8244B27 | -0.995663 | 2.82E-05 |
| lncRNA_020959 | Traes_1AL_1EBD4E9F5 | 0.9935366 | 6.25E-05 |
| lncRNA_024807 | Traes_1AL_1EBD4E9F5 | 0.9944491 | 4.61E-05 |
| lncRNA_061738 | Traes_1AL_1EBD4E9F5 | 0.9901894 | 0.0001439 |
| lncRNA_014501 | Traes_1AL_1F3A0CD1F | -0.992633 | 8.12E-05 |
| lncRNA_047461 | Traes_1AL_1F3A0CD1F | -0.994808 | 4.04E-05 |
| lncRNA_027894 | Traes_1AL_22BFD3F69 | 0.9902902 | 0.000141 |
| lncRNA_025391 | Traes_1AL_27293A71B | -0.996862 | 1.48E-05 |
| lncRNA_046989 | Traes_1AL_2EC583AC9 | 0.9979725 | 6.16E-06 |
| lncRNA_047461 | Traes_1AL_2F2E5017B | 0.9902034 | 0.0001435 |
| lncRNA_048256 | Traes_1AL_2F2E5017B | 0.9917188 | 0.0001026 |
| lncRNA_018111 | Traes_1AL_34404D5D8 | 0.9912304 | 0.000115 |
| lncRNA_047461 | Traes_1AL_34404D5D8 | 0.9922856 | 8.90E-05 |
| lncRNA_048256 | Traes_1AL_34404D5D8 | 0.990811 | 0.0001263 |
| lncRNA_063009 | Traes_1AL_34404D5D8 | 0.9934063 | 6.51E-05 |
| lncRNA_013190 | Traes_1AL_36750809D | 0.9919149 | 9.78E-05 |
| lncRNA_018078 | Traes_1AL_36750809D | 0.9938432 | 5.67E-05 |
| lncRNA_048256 | Traes_1AL_36750809D | 0.9913461 | 0.000112 |
| lncRNA_051318 | Traes_1AL_36750809D | 0.9955468 | 2.97E-05 |
| lncRNA_022554 | Traes_1AL_36B201285 | -0.994351 | 4.78E-05 |
| lncRNA_048256 | Traes_1AL_36B201285 | -0.992552 | 8.30E-05 |
| lncRNA_063009 | Traes_1AL_36B201285 | -0.993387 | 6.54E-05 |
| lncRNA_000823 | Traes_1AL_3791A0A3A | 0.9943617 | 4.76E-05 |
| TRAES3BF053100050CFD_g | Traes_1AL_3996E29ED | -0.991206 | 0.0001157 |
| lncRNA_059554 | Traes_1AL_3996E29ED | -0.991101 | 0.0001184 |
| lncRNA_039420 | Traes_1AL_3B82F56A2 | -0.995691 | 2.78E-05 |
| lncRNA_020477 | Traes_1AL_3C825BAE8 | 0.9950273 | 3.70E-05 |
| lncRNA_047461 | Traes_1AL_3C825BAE8 | -0.992262 | 8.96E-05 |
| lncRNA_048668 | Traes_1AL_3C825BAE8 | 0.9900947 | 0.0001467 |
| lncRNA_012242 | Traes_1AL_3EC96C539 | -0.991813 | 0.0001003 |
| lncRNA_054317 | Traes_1AL_3EC96C539 | -0.99088 | 0.0001244 |
| TRAES3BF052700300CFD_g | Traes_1AL_45A9AD1EA | -0.991778 | 0.0001011 |
| lncRNA_029528 | Traes_1AL_4B3591E96 | 0.9939668 | 5.45E-05 |
| lncRNA_029013 | Traes_1AL_4F0745006 | 0.9949954 | 3.75E-05 |
| lncRNA_008500 | Traes_1AL_53F3380AF | -0.992204 | 9.09E-05 |
| lncRNA_006361 | Traes_1AL_55440EF31 | 0.9948417 | 3.98E-05 |
| lncRNA_012610 | Traes_1AL_55440EF31 | 0.9902042 | 0.0001435 |
| lncRNA_015487 | Traes_1AL_55440EF31 | 0.9905683 | 0.000133 |
| lncRNA_018111 | Traes_1AL_55440EF31 | 0.990244 | 0.0001423 |
| lncRNA_051318 | Traes_1AL_55440EF31 | 0.9955977 | 2.90E-05 |
| lncRNA_032287 | Traes_1AL_5D3EECC71 | -0.992921 | 7.50E-05 |
| lncRNA_008977 | Traes_1AL_5D90CAB50 | -0.995325 | 3.27E-05 |
| lncRNA_014504 | Traes_1AL_5E4EECFBC | 0.9970925 | 1.27E-05 |
| lncRNA_010675 | Traes_1AL_5EEF86979 | -0.990736 | 0.0001283 |
| lncRNA_014501 | Traes_1AL_5EEF86979 | 0.9955875 | 2.92E-05 |
| lncRNA_046989 | Traes_1AL_638691536 | 0.9914166 | 0.0001102 |
| lncRNA_009293 | Traes_1AL_6C5C28AA8 | 0.9956312 | 2.86E-05 |
| lncRNA_009698 | Traes_1AL_6CDAB2400 | 0.9989156 | 1.76E-06 |
| lncRNA_012610 | Traes_1AL_6CDAB2400 | 0.9925169 | 8.38E-05 |
| lncRNA_013190 | Traes_1AL_6CDAB2400 | 0.9914048 | 0.0001105 |
| lncRNA_048256 | Traes_1AL_6CDAB2400 | 0.9909657 | 0.0001221 |
| lncRNA_051551 | Traes_1AL_734F4BCAF | 0.9979114 | 6.54E-06 |
| lncRNA_048825 | Traes_1AL_7402A5761 | 0.9973829 | 1.03E-05 |
| lncRNA_005569 | Traes_1AL_7FEC8D7C5 | 0.9926965 | 7.98E-05 |
| lncRNA_000823 | Traes_1AL_88D49649D | 0.9919089 | 9.79E-05 |
| lncRNA_015487 | Traes_1AL_8EF2B803D | -0.991255 | 0.0001144 |
| Traes_1BS_B58657408 | Traes_1AL_8F917ED50 | -0.990747 | 0.000128 |
| lncRNA_039420 | Traes_1AL_8F917ED50 | -0.991904 | 9.80E-05 |
| lncRNA_074837 | Traes_1AL_8F917ED50 | 0.9942822 | 4.89E-05 |
| lncRNA_076127 | Traes_1AL_9022E66BC | 0.9903534 | 0.0001391 |
| TRAES3BF053100050CFD_g | Traes_1AL_95235FDBD | 0.9948164 | 4.02E-05 |
| lncRNA_013190 | Traes_1AL_95235FDBD | 0.9955736 | 2.93E-05 |
| lncRNA_018078 | Traes_1AL_95235FDBD | 0.9973851 | 1.02E-05 |
| lncRNA_051318 | Traes_1AL_95235FDBD | 0.9958944 | 2.52E-05 |
| lncRNA_063127 | Traes_1AL_95235FDBD | -0.992068 | 9.41E-05 |
| lncRNA_059323 | Traes_1AL_954898895 | 0.9905834 | 0.0001326 |
| lncRNA_039420 | Traes_1AL_990E4507E | -0.994098 | 5.21E-05 |
| lncRNA_051551 | Traes_1AL_9B45E596F | 0.9981577 | 5.09E-06 |
| lncRNA_078349 | Traes_1AL_9CF44F05F | -0.991041 | 0.00012 |
| lncRNA_021433 | Traes_1AL_A77FBFDDB | 0.9967835 | 1.55E-05 |
| lncRNA_012608 | Traes_1AL_A8190CDDF | 0.9903786 | 0.0001384 |
| lncRNA_014538 | Traes_1AL_A8190CDDF | 0.9909319 | 0.000123 |
| lncRNA_008500 | Traes_1AL_A9FB6BF52 | 0.991278 | 0.0001138 |
| lncRNA_039420 | Traes_1AL_B23CA03E6 | -0.991119 | 0.000118 |
| lncRNA_021433 | Traes_1AL_B3737F042 | -0.995092 | 3.61E-05 |
| lncRNA_009362 | Traes_1AL_B427A0153 | -0.994472 | 4.58E-05 |
| lncRNA_012610 | Traes_1AL_B85068B07 | -0.990826 | 0.0001259 |
| lncRNA_018111 | Traes_1AL_B85068B07 | -0.991346 | 0.000112 |
| lncRNA_051318 | Traes_1AL_B85068B07 | -0.994167 | 5.09E-05 |
| lncRNA_013231 | Traes_1AL_B8B0A092C | 0.9914578 | 0.0001091 |
| lncRNA_020959 | Traes_1AL_B8B0A092C | 0.9921697 | 9.17E-05 |
| TRAES3BF021800100CFD_g | Traes_1AL_BB3D071F8 | 0.9903399 | 0.0001395 |
| lncRNA_045725 | Traes_1AL_BB3D071F8 | 1 | 0 |
| lncRNA_051428 | Traes_1AL_BB3D071F8 | 1 | 0 |
| lncRNA_057614 | Traes_1AL_BB3D071F8 | 1 | 0 |
| lncRNA_065747 | Traes_1AL_BB3D071F8 | 1 | 0 |
| lncRNA_008784 | Traes_1AL_BF0FB9CCC | -0.991728 | 0.0001023 |
| lncRNA_047461 | Traes_1AL_C1EF04361 | 0.997483 | 9.49E-06 |
| lncRNA_058136 | Traes_1AL_C53A9A6B2 | 0.9966675 | 1.66E-05 |
| lncRNA_015487 | Traes_1AL_C73885878 | 0.9945011 | 4.53E-05 |
| lncRNA_018111 | Traes_1AL_C73885878 | 0.9906419 | 0.000131 |
| lncRNA_054317 | Traes_1AL_C73885878 | 0.9946392 | 4.30E-05 |
| lncRNA_063009 | Traes_1AL_C73885878 | 0.9951623 | 3.50E-05 |
| lncRNA_012610 | Traes_1AL_CC3EB56C4 | -0.990692 | 0.0001296 |
| lncRNA_013190 | Traes_1AL_CC3EB56C4 | -0.99286 | 7.63E-05 |
| lncRNA_048256 | Traes_1AL_CC3EB56C4 | -0.998589 | 2.99E-06 |
| lncRNA_063009 | Traes_1AL_CC3EB56C4 | -0.993824 | 5.71E-05 |
| lncRNA_007700 | Traes_1AL_CD83B0FA4 | 0.995379 | 3.20E-05 |
| lncRNA_078349 | Traes_1AL_CD83B0FA4 | 0.990387 | 0.0001382 |
| lncRNA_024812 | Traes_1AL_D2BCFD121 | 0.9950042 | 3.74E-05 |
| lncRNA_024807 | Traes_1AL_D7BDD58BD | 0.9913197 | 0.0001127 |
| lncRNA_061738 | Traes_1AL_D7BDD58BD | 0.9907467 | 0.000128 |
| TRAES3BF053100050CFD_g | Traes_1AL_DD4604320 | 0.9960615 | 2.32E-05 |
| lncRNA_007700 | Traes_1AL_DD4604320 | 0.9961165 | 2.26E-05 |
| lncRNA_013190 | Traes_1AL_DD4604320 | 0.9941653 | 5.10E-05 |
| lncRNA_018078 | Traes_1AL_DD4604320 | 0.9989889 | 1.53E-06 |
| lncRNA_043877 | Traes_1AL_DD4604320 | 0.9944712 | 4.58E-05 |
| lncRNA_032287 | Traes_1AL_E12BF1698 | -0.992686 | 8.00E-05 |
| lncRNA_008977 | Traes_1AL_E25202CC2 | 0.9925455 | 8.31E-05 |
| lncRNA_048256 | Traes_1AL_E25202CC2 | 0.992195 | 9.11E-05 |
| lncRNA_015487 | Traes_1AL_EF6194976 | -0.990711 | 0.000129 |
| TRAES3BF052700300CFD_g | Traes_1AL_F245CC03B | -0.991955 | 9.68E-05 |
| lncRNA_001839 | Traes_1AL_F245CC03B | -0.990619 | 0.0001316 |
| lncRNA_007700 | Traes_1AS_06988AF2C | -0.994904 | 3.89E-05 |
| lncRNA_048256 | Traes_1AS_06988AF2C | -0.99382 | 5.72E-05 |
| lncRNA_048256 | Traes_1AS_08A0A8A0E | -0.995592 | 2.91E-05 |
| lncRNA_020959 | Traes_1AS_1234FA28F | 0.9907225 | 0.0001287 |
| lncRNA_010675 | Traes_1AS_12E5ECBE2 | 0.9964633 | 1.87E-05 |
| lncRNA_016209 | Traes_1AS_24511D656 | 0.995336 | 3.26E-05 |
| lncRNA_054824 | Traes_1AS_24511D656 | 0.9988991 | 1.82E-06 |
| lncRNA_073681 | Traes_1AS_24511D656 | 0.9956242 | 2.87E-05 |
| lncRNA_004117 | Traes_1AS_34DBDBA7D | 0.9908791 | 0.0001244 |
| lncRNA_013231 | Traes_1AS_34DBDBA7D | 0.992949 | 7.44E-05 |
| lncRNA_081387 | Traes_1AS_36865F81C | -0.991426 | 0.00011 |
| lncRNA_018078 | Traes_1AS_3FDE06628 | 0.9931315 | 7.06E-05 |
| lncRNA_029013 | Traes_1AS_3FDE06628 | 0.9925495 | 8.31E-05 |
| lncRNA_043877 | Traes_1AS_3FDE06628 | 0.9906774 | 0.00013 |
| lncRNA_063127 | Traes_1AS_3FDE06628 | -0.998437 | 3.66E-06 |
| lncRNA_080648 | Traes_1AS_3FDE06628 | 0.9953301 | 3.27E-05 |
| lncRNA_014504 | Traes_1AS_400EF6C081 | 0.9942243 | 4.99E-05 |
| lncRNA_020959 | Traes_1AS_400EF6C081 | 0.9905213 | 0.0001343 |
| lncRNA_029013 | Traes_1AS_400EF6C081 | 0.9900632 | 0.0001476 |
| lncRNA_063127 | Traes_1AS_400EF6C081 | -0.991103 | 0.0001184 |
| Traes_1BS_B58657408 | Traes_1AS_4AD2F6772 | -0.994957 | 3.81E-05 |
| lncRNA_029013 | Traes_1AS_536E18ACD | 0.9915655 | 0.0001064 |
| lncRNA_056853 | Traes_1AS_651A2A839 | -0.993408 | 6.50E-05 |
| lncRNA_018111 | Traes_1AS_77FA29CF3 | 0.9906779 | 0.0001299 |
| lncRNA_047461 | Traes_1AS_77FA29CF3 | 0.9912181 | 0.0001153 |
| lncRNA_014504 | Traes_1AS_82CA6D2F3 | 0.992261 | 8.96E-05 |
| lncRNA_015322 | Traes_1AS_82CA6D2F3 | -0.992482 | 8.46E-05 |
| lncRNA_006361 | Traes_1AS_863D41595 | -0.997208 | 1.17E-05 |
| lncRNA_015487 | Traes_1AS_863D41595 | -0.993121 | 7.08E-05 |
| TRAES3BF053100050CFD_g | Traes_1AS_8A319EC97 | 0.9934312 | 6.46E-05 |
| lncRNA_007700 | Traes_1AS_8A319EC97 | 0.9918503 | 9.94E-05 |
| lncRNA_013190 | Traes_1AS_8A319EC97 | 0.9966988 | 1.63E-05 |
| lncRNA_018078 | Traes_1AS_8A319EC97 | 0.9985187 | 3.29E-06 |
| lncRNA_048256 | Traes_1AS_8A319EC97 | 0.9909109 | 0.0001235 |
| lncRNA_051318 | Traes_1AS_8A319EC97 | 0.995425 | 3.13E-05 |
| lncRNA_063127 | Traes_1AS_8A319EC97 | -0.990364 | 0.0001388 |
| lncRNA_076127 | Traes_1AS_A85EF2464 | 0.9909791 | 0.0001217 |
| lncRNA_022554 | Traes_1AS_B26B7D59F | -0.997599 | 8.64E-06 |
| lncRNA_017751 | Traes_1AS_C261D2216 | -0.996211 | 2.15E-05 |
| lncRNA_025391 | Traes_1AS_C261D2216 | -0.995615 | 2.88E-05 |
| lncRNA_024812 | Traes_1AS_C3ACFA4D8 | 0.9950755 | 3.63E-05 |
| lncRNA_048825 | Traes_1AS_EE98F3ED0 | 0.9922304 | 9.03E-05 |
| lncRNA_006361 | Traes_1BL_0302D9F63 | 0.9930943 | 7.14E-05 |
| lncRNA_012610 | Traes_1BL_0302D9F63 | 0.9957342 | 2.73E-05 |
| lncRNA_013190 | Traes_1BL_0302D9F63 | 0.9943069 | 4.85E-05 |
| lncRNA_015487 | Traes_1BL_0302D9F63 | 0.9943395 | 4.80E-05 |
| lncRNA_018111 | Traes_1BL_0302D9F63 | 0.9928108 | 7.73E-05 |
| lncRNA_051318 | Traes_1BL_0302D9F63 | 0.9970426 | 1.31E-05 |
| Traes_1BS_B58657408 | Traes_1BL_05865F944 | -0.991492 | 0.0001083 |
| lncRNA_039420 | Traes_1BL_063023D50 | -0.990929 | 0.0001231 |
| lncRNA_009698 | Traes_1BL_07898F3B2 | 0.9925584 | 8.29E-05 |
| lncRNA_012610 | Traes_1BL_07898F3B2 | 0.9927861 | 7.79E-05 |
| lncRNA_013190 | Traes_1BL_07898F3B2 | 0.9996973 | 1.37E-07 |
| lncRNA_018078 | Traes_1BL_07898F3B2 | 0.9919866 | 9.61E-05 |
| lncRNA_048256 | Traes_1BL_07898F3B2 | 0.995591 | 2.91E-05 |
| lncRNA_051318 | Traes_1BL_07898F3B2 | 0.9936237 | 6.09E-05 |
| lncRNA_014501 | Traes_1BL_07B8BFA42 | -0.991558 | 0.0001066 |
| lncRNA_024807 | Traes_1BL_07B8BFA42 | -0.993238 | 6.84E-05 |
| lncRNA_061738 | Traes_1BL_07B8BFA42 | -0.992516 | 8.38E-05 |
| lncRNA_029013 | Traes_1BL_0A3CDBCFC | 0.994346 | 4.79E-05 |
| lncRNA_063127 | Traes_1BL_0A3CDBCFC | -0.994181 | 5.07E-05 |
| lncRNA_063009 | Traes_1BL_0A996D4A2 | -0.990642 | 0.000131 |
| lncRNA_068995 | Traes_1BL_0F1D3BB9E | 0.9900909 | 0.0001468 |
| lncRNA_083996 | Traes_1BL_13E2A6068 | 0.9919452 | 9.71E-05 |
| lncRNA_012242 | Traes_1BL_16964487D | 0.9929644 | 7.41E-05 |
| lncRNA_068995 | Traes_1BL_19E3557DD | 0.9933549 | 6.61E-05 |
| lncRNA_039943 | Traes_1BL_1CCE08A29 | 0.994774 | 4.09E-05 |
| lncRNA_048668 | Traes_1BL_1CCE08A29 | 0.9916206 | 0.000105 |
| lncRNA_051888 | Traes_1BL_1CCE08A29 | -0.993653 | 6.03E-05 |
| lncRNA_039420 | Traes_1BL_22B15A022 | -0.993854 | 5.65E-05 |
| lncRNA_074837 | Traes_1BL_22B15A022 | 0.990978 | 0.0001217 |
| TRAES3BF052700300CFD_g | Traes_1BL_28DB0E01C | -0.992107 | 9.32E-05 |
| lncRNA_047461 | Traes_1BL_28DB0E01C | -0.993856 | 5.65E-05 |
| lncRNA_048668 | Traes_1BL_2F2FCE5CD | 0.9977539 | 7.56E-06 |
| lncRNA_051888 | Traes_1BL_2F2FCE5CD | -0.992259 | 8.96E-05 |
| lncRNA_053766 | Traes_1BL_2F2FCE5CD | 0.9918669 | 9.90E-05 |
| lncRNA_008977 | Traes_1BL_301F6C93C | 0.9901998 | 0.0001436 |
| lncRNA_076127 | Traes_1BL_36B22EF51 | 0.9901149 | 0.0001461 |
| lncRNA_074658 | Traes_1BL_38CAFCA56 | -0.995728 | 2.73E-05 |
| lncRNA_074658 | Traes_1BL_38CAFCA561 | -0.995822 | 2.61E-05 |
| lncRNA_014504 | Traes_1BL_46DCF8472 | 0.9920093 | 9.55E-05 |
| lncRNA_020959 | Traes_1BL_46DCF8472 | 0.990297 | 0.0001408 |
| lncRNA_068995 | Traes_1BL_4AB17FFF4 | 0.9918456 | 9.95E-05 |
| lncRNA_081387 | Traes_1BL_5049DC7EA | -0.99284 | 7.67E-05 |
| lncRNA_029013 | Traes_1BL_51774DCD7 | 0.9924478 | 8.53E-05 |
| lncRNA_037690 | Traes_1BL_51774DCD7 | 0.9944756 | 4.57E-05 |
| lncRNA_017751 | Traes_1BL_5390D3FF8 | -0.990412 | 0.0001374 |
| lncRNA_025391 | Traes_1BL_5390D3FF8 | -0.995569 | 2.94E-05 |
| lncRNA_063547 | Traes_1BL_553D7A793 | 0.99006 | 0.0001477 |
| lncRNA_048256 | Traes_1BL_55F0D425F | -0.99516 | 3.51E-05 |
| lncRNA_017751 | Traes_1BL_56BBB4B2F | -0.990622 | 0.0001315 |
| lncRNA_074837 | Traes_1BL_5838C5EE4 | 0.9933058 | 6.71E-05 |
| lncRNA_009362 | Traes_1BL_59A688795 | -0.990434 | 0.0001368 |
| lncRNA_051318 | Traes_1BL_5CD8FB94C | 0.9921689 | 9.17E-05 |
| lncRNA_039420 | Traes_1BL_5ED9A20B31 | -0.997821 | 7.12E-06 |
| lncRNA_039420 | Traes_1BL_5F8449E10 | 0.9926691 | 8.04E-05 |
| lncRNA_024812 | Traes_1BL_6215D102F | 0.9927215 | 7.93E-05 |
| lncRNA_000928 | Traes_1BL_63874F598 | 0.9915074 | 0.0001079 |
| lncRNA_013053 | Traes_1BL_63874F598 | 0.9936651 | 6.01E-05 |
| lncRNA_039943 | Traes_1BL_63874F598 | 0.9922193 | 9.06E-05 |
| lncRNA_051888 | Traes_1BL_63874F598 | -0.99254 | 8.33E-05 |
| lncRNA_058136 | Traes_1BL_63874F598 | 0.9956861 | 2.79E-05 |
| lncRNA_008977 | Traes_1BL_65789BC98 | -0.994796 | 4.05E-05 |
| lncRNA_048668 | Traes_1BL_681629423 | 0.9967283 | 1.60E-05 |
| lncRNA_051888 | Traes_1BL_681629423 | -0.992436 | 8.56E-05 |
| lncRNA_046989 | Traes_1BL_68E4D6F70 | -0.993567 | 6.19E-05 |
| lncRNA_024152 | Traes_1BL_6F04DAB5B | 0.9964122 | 1.93E-05 |
| lncRNA_056853 | Traes_1BL_6F04DAB5B | 0.9900986 | 0.0001466 |
| lncRNA_014501 | Traes_1BL_71EF97B701 | 0.9955372 | 2.98E-05 |
| lncRNA_024807 | Traes_1BL_71EF97B701 | 0.9911223 | 0.0001179 |
| lncRNA_061738 | Traes_1BL_71EF97B701 | 0.9944711 | 4.58E-05 |
| lncRNA_032287 | Traes_1BL_72EC293D2 | -0.991359 | 0.0001117 |
| lncRNA_033754 | Traes_1BL_72EC293D2 | -0.990671 | 0.0001301 |
| lncRNA_006011 | Traes_1BL_751E80A9A | 0.9921068 | 9.32E-05 |
| lncRNA_063127 | Traes_1BL_78443376F | -0.995284 | 3.33E-05 |
| lncRNA_080648 | Traes_1BL_78443376F | 0.9925014 | 8.41E-05 |
| TRAES3BF021800100CFD_g | Traes_1BL_78D0F4408 | 0.9903399 | 0.0001395 |
| lncRNA_045725 | Traes_1BL_78D0F4408 | 1 | 0 |
| lncRNA_051428 | Traes_1BL_78D0F4408 | 1 | 0 |
| lncRNA_057614 | Traes_1BL_78D0F4408 | 1 | 0 |
| lncRNA_065747 | Traes_1BL_78D0F4408 | 1 | 0 |
| TRAES3BF053100050CFD_g | Traes_1BL_82507217C | 0.9989633 | 1.61E-06 |
| lncRNA_013190 | Traes_1BL_82507217C | 0.991336 | 0.0001123 |
| lncRNA_018078 | Traes_1BL_82507217C | 0.9950618 | 3.65E-05 |
| lncRNA_043877 | Traes_1BL_82507217C | 0.9976817 | 8.06E-06 |
| lncRNA_080648 | Traes_1BL_82507217C | 0.9945269 | 4.48E-05 |
| lncRNA_039943 | Traes_1BL_8F261F085 | 0.9934338 | 6.45E-05 |
| lncRNA_051888 | Traes_1BL_8F261F085 | -0.993364 | 6.59E-05 |
| lncRNA_058136 | Traes_1BL_8F261F085 | 0.9909332 | 0.0001229 |
| lncRNA_076127 | Traes_1BL_91A98E876 | 0.995409 | 3.16E-05 |
| TRAES3BF052700300CFD_g | Traes_1BL_930E75040 | -0.992561 | 8.28E-05 |
| lncRNA_022554 | Traes_1BL_930E75040 | -0.993084 | 7.16E-05 |
| lncRNA_024812 | Traes_1BL_987C54D65 | 0.9927878 | 7.78E-05 |
| lncRNA_082364 | Traes_1BL_9B3C88F90 | 0.9960575 | 2.33E-05 |
| lncRNA_009698 | Traes_1BL_9C0934A77 | -0.991953 | 9.69E-05 |
| lncRNA_013190 | Traes_1BL_9C0934A77 | -0.992878 | 7.59E-05 |
| lncRNA_019725 | Traes_1BL_9C0934A77 | -0.991658 | 0.0001041 |
| lncRNA_048256 | Traes_1BL_9C0934A77 | -0.994353 | 4.77E-05 |
| lncRNA_051318 | Traes_1BL_9C0934A77 | -0.991067 | 0.0001193 |
| lncRNA_033754 | Traes_1BL_A7A180B22 | -0.9908 | 0.0001266 |
| lncRNA_064639 | Traes_1BL_A7A180B22 | -0.992893 | 7.56E-05 |
| lncRNA_008977 | Traes_1BL_AE6A21360 | -0.996838 | 1.50E-05 |
| lncRNA_047461 | Traes_1BL_AE6A21360 | -0.990898 | 0.0001239 |
| lncRNA_063009 | Traes_1BL_AE6A21360 | -0.990703 | 0.0001292 |
| lncRNA_018078 | Traes_1BL_B4D15E522 | -0.990845 | 0.0001253 |
| lncRNA_051318 | Traes_1BL_B4D15E522 | -0.991602 | 0.0001055 |
| lncRNA_063127 | Traes_1BL_B4D15E522 | 0.9979868 | 6.08E-06 |
| lncRNA_048668 | Traes_1BL_B7318A659 | 0.9947621 | 4.11E-05 |
| lncRNA_008977 | Traes_1BL_B7C41EBAD | -0.991061 | 0.0001195 |
| lncRNA_053766 | Traes_1BL_B7F04D24D | -0.990222 | 0.000143 |
| lncRNA_008500 | Traes_1BL_BDF0801D01 | 0.993165 | 6.99E-05 |
| lncRNA_057390 | Traes_1BL_BDF0801D01 | 0.9913444 | 0.0001121 |
| lncRNA_068995 | Traes_1BL_C40D1B0CC | -0.990518 | 0.0001344 |
| lncRNA_008977 | Traes_1BL_C574F2491 | 0.9903497 | 0.0001392 |
| lncRNA_012242 | Traes_1BL_C574F2491 | 0.9925869 | 8.22E-05 |
| lncRNA_054317 | Traes_1BL_C574F2491 | 0.991779 | 0.0001011 |
| lncRNA_063009 | Traes_1BL_C574F2491 | 0.9941385 | 5.14E-05 |
| lncRNA_027894 | Traes_1BL_C77593483 | 0.9924418 | 8.55E-05 |
| TRAES3BF053100050CFD_g | Traes_1BL_C8C5419FE | -0.99278 | 7.80E-05 |
| lncRNA_007700 | Traes_1BL_C8C5419FE | -0.993293 | 6.73E-05 |
| lncRNA_018078 | Traes_1BL_C8C5419FE | -0.994978 | 3.78E-05 |
| lncRNA_063127 | Traes_1BL_C8C5419FE | 0.9909934 | 0.0001213 |
| lncRNA_009293 | Traes_1BL_C8C7930AD | 0.9911143 | 0.0001181 |
| lncRNA_013190 | Traes_1BL_CC64CA41E | 0.9910769 | 0.0001191 |
| lncRNA_012242 | Traes_1BL_CCA6E16B1 | -0.990749 | 0.000128 |
| lncRNA_054317 | Traes_1BL_CCA6E16B1 | -0.994197 | 5.04E-05 |
| lncRNA_032048 | Traes_1BL_D6EDAAEAC | 0.99055 | 0.0001335 |
| lncRNA_072935 | Traes_1BL_D7214C937 | -0.991893 | 9.83E-05 |
| lncRNA_020959 | Traes_1BL_D90F27EE3 | -0.998945 | 1.67E-06 |
| lncRNA_024807 | Traes_1BL_D90F27EE3 | -0.997381 | 1.03E-05 |
| lncRNA_044308 | Traes_1BL_D90F27EE3 | -0.990071 | 0.0001474 |
| lncRNA_006361 | Traes_1BL_DA6486931 | -0.994937 | 3.84E-05 |
| lncRNA_015487 | Traes_1BL_DA6486931 | -0.996683 | 1.65E-05 |
| lncRNA_018111 | Traes_1BL_DA6486931 | -0.991797 | 0.0001007 |
| lncRNA_012242 | Traes_1BL_E595222FC | -0.997413 | 1.00E-05 |
| lncRNA_054317 | Traes_1BL_E595222FC | -0.998964 | 1.61E-06 |
| lncRNA_063009 | Traes_1BL_E595222FC | -0.991335 | 0.0001123 |
| lncRNA_027894 | Traes_1BL_E6A53E6FA | 0.9951679 | 3.50E-05 |
| lncRNA_014504 | Traes_1BL_E9549FBDC | 0.9951617 | 3.51E-05 |
| lncRNA_019725 | Traes_1BL_E9549FBDC | 0.9915018 | 0.000108 |
| lncRNA_051888 | Traes_1BL_E9549FBDC | 0.9917849 | 0.000101 |
| lncRNA_058136 | Traes_1BL_E9549FBDC | -0.991732 | 0.0001023 |
| lncRNA_018111 | Traes_1BL_EB95F4919 | 0.9929421 | 7.45E-05 |
| lncRNA_051318 | Traes_1BL_EB95F4919 | 0.9941826 | 5.07E-05 |
| lncRNA_022554 | Traes_1BL_EDDD35B93 | 0.9904603 | 0.0001361 |
| lncRNA_006270 | Traes_1BL_F026049BC | 0.9928043 | 7.75E-05 |
| TRAES3BF052700300CFD_g | Traes_1BL_F143786F5 | -0.993247 | 6.82E-05 |
| TRAES3BF052700300CFD_g | Traes_1BL_F32B62E49 | 0.9955231 | 3.00E-05 |
| lncRNA_012242 | Traes_1BL_F4D20AE54 | -0.990032 | 0.0001485 |
| lncRNA_064639 | Traes_1BL_F4D20AE54 | -0.994083 | 5.24E-05 |
| lncRNA_039289 | Traes_1BL_F9D93E1F8 | 0.9974407 | 9.82E-06 |
| lncRNA_020959 | Traes_1BL_FA20AEA33 | -0.992121 | 9.29E-05 |
| lncRNA_024807 | Traes_1BL_FA20AEA33 | -0.990785 | 0.000127 |
| lncRNA_044308 | Traes_1BL_FA20AEA33 | -0.99764 | 8.35E-06 |
| lncRNA_048668 | Traes_1BL_FD97D9FAF | 0.9931802 | 6.96E-05 |
| lncRNA_051888 | Traes_1BL_FD97D9FAF | -0.990628 | 0.0001313 |
| lncRNA_001072 | Traes_1BS_01E501596 | 0.9961731 | 2.19E-05 |
| lncRNA_024464 | Traes_1BS_01E501596 | 0.9961731 | 2.19E-05 |
| lncRNA_054394 | Traes_1BS_01E501596 | 0.9961731 | 2.19E-05 |
| lncRNA_061348 | Traes_1BS_01E501596 | 0.9961731 | 2.19E-05 |
| lncRNA_065442 | Traes_1BS_01E501596 | 0.9996631 | 1.70E-07 |
| lncRNA_044556 | Traes_1BS_1425AAD7C | 0.9965918 | 1.74E-05 |
| lncRNA_053655 | Traes_1BS_14FF4B034 | 0.9908137 | 0.0001262 |
| lncRNA_000928 | Traes_1BS_18474C493 | 0.9963764 | 1.97E-05 |
| lncRNA_039943 | Traes_1BS_18474C493 | 0.9932267 | 6.87E-05 |
| lncRNA_001072 | Traes_1BS_188DDFB56 | 0.9921705 | 9.17E-05 |
| lncRNA_024464 | Traes_1BS_188DDFB56 | 0.9921705 | 9.17E-05 |
| lncRNA_054394 | Traes_1BS_188DDFB56 | 0.9921705 | 9.17E-05 |
| lncRNA_061348 | Traes_1BS_188DDFB56 | 0.9921705 | 9.17E-05 |
| lncRNA_065442 | Traes_1BS_188DDFB56 | 0.9983215 | 4.22E-06 |
| lncRNA_077407 | Traes_1BS_188DDFB56 | 0.9927074 | 7.96E-05 |
| lncRNA_047461 | Traes_1BS_1E67F5AE2 | -0.996761 | 1.57E-05 |
| lncRNA_024812 | Traes_1BS_2E766829E | 0.9926393 | 8.11E-05 |
| lncRNA_076127 | Traes_1BS_30DE427D5 | 0.9903149 | 0.0001402 |
| TRAES3BF177500010CFD_g | Traes_1BS_44CE2D44E | 0.9930049 | 7.32E-05 |
| lncRNA_013190 | Traes_1BS_59F91C2FE | 0.9906832 | 0.0001298 |
| lncRNA_048668 | Traes_1BS_64E9CC6E0 | 0.9953641 | 3.22E-05 |
| lncRNA_048256 | Traes_1BS_672D28589 | 0.9918932 | 9.83E-05 |
| lncRNA_000823 | Traes_1BS_6FE6E86C3 | 0.9944467 | 4.62E-05 |
| lncRNA_048256 | Traes_1BS_8182F75AD | -0.994206 | 5.03E-05 |
| lncRNA_063009 | Traes_1BS_8182F75AD | -0.993146 | 7.03E-05 |
| lncRNA_072167 | Traes_1BS_8A19C460B | 0.9910703 | 0.0001193 |
| lncRNA_024152 | Traes_1BS_9C956DD02 | 0.9945892 | 4.38E-05 |
| lncRNA_039325 | Traes_1BS_A5AFE568B | -0.994981 | 3.77E-05 |
| lncRNA_026968 | Traes_1BS_CEB5DA163 | 0.9918254 | 1.00E-04 |
| lncRNA_063547 | Traes_1BS_CEB5DA163 | 0.9978242 | 7.10E-06 |
| lncRNA_078349 | Traes_1BS_CEB5DA163 | 0.9940965 | 5.22E-05 |
| lncRNA_048668 | Traes_1BS_D0D706E9B | 0.9908366 | 0.0001256 |
| lncRNA_053766 | Traes_1BS_D0D706E9B | 0.9914048 | 0.0001105 |
| lncRNA_000823 | Traes_1BS_DD55B7D8F | 0.9942576 | 4.94E-05 |
| lncRNA_064639 | Traes_1BS_E05D6C92A1 | 0.9913995 | 0.0001106 |
| TRAES3BF051200090CFD_g | Traes_1BS_F9B6B9C50 | 0.997863 | 6.85E-06 |
| lncRNA_005569 | Traes_1BS_F9B6B9C50 | 0.9940532 | 5.29E-05 |
| lncRNA_039289 | Traes_1BS_F9B6B9C50 | -0.990632 | 0.0001312 |
| lncRNA_013231 | Traes_1DL_03D1DC620 | 0.9990683 | 1.30E-06 |
| lncRNA_044556 | Traes_1DL_03D1DC620 | 0.990723 | 0.0001287 |
| TRAES3BF053100050CFD_g | Traes_1DL_03FCF0D83 | 0.9940413 | 5.32E-05 |
| lncRNA_013190 | Traes_1DL_03FCF0D83 | 0.9900352 | 0.0001485 |
| lncRNA_018078 | Traes_1DL_03FCF0D83 | 0.9945163 | 4.50E-05 |
| lncRNA_051318 | Traes_1DL_03FCF0D83 | 0.9943456 | 4.79E-05 |
| lncRNA_063127 | Traes_1DL_03FCF0D83 | -0.995171 | 3.49E-05 |
| lncRNA_080648 | Traes_1DL_03FCF0D83 | 0.9942372 | 4.97E-05 |
| lncRNA_046989 | Traes_1DL_0715CD9A6 | 0.9905792 | 0.0001327 |
| lncRNA_020959 | Traes_1DL_0D7B55EF1 | 0.9941037 | 5.20E-05 |
| lncRNA_024807 | Traes_1DL_0D7B55EF1 | 0.9952934 | 3.32E-05 |
| lncRNA_061738 | Traes_1DL_0D7B55EF1 | 0.9912292 | 0.0001151 |
| lncRNA_046989 | Traes_1DL_0DEDB7543 | 0.9954114 | 3.15E-05 |
| lncRNA_014501 | Traes_1DL_122F16FB2 | 0.9941327 | 5.15E-05 |
| lncRNA_047461 | Traes_1DL_122F16FB2 | 0.9953773 | 3.20E-05 |
| lncRNA_059323 | Traes_1DL_260008870 | 0.9930201 | 7.29E-05 |
| lncRNA_081387 | Traes_1DL_260008870 | 0.9912966 | 0.0001133 |
| lncRNA_048668 | Traes_1DL_274235AB5 | 0.9923811 | 8.69E-05 |
| lncRNA_047461 | Traes_1DL_291C515F3 | 0.9992698 | 8.00E-07 |
| lncRNA_009698 | Traes_1DL_2A2451D6E | 0.9949924 | 3.76E-05 |
| lncRNA_012610 | Traes_1DL_2A2451D6E | 0.9953501 | 3.24E-05 |
| lncRNA_013190 | Traes_1DL_2A2451D6E | 0.9959494 | 2.46E-05 |
| lncRNA_048256 | Traes_1DL_2A2451D6E | 0.9908935 | 0.000124 |
| lncRNA_056853 | Traes_1DL_2B11C55B9 | -0.994335 | 4.81E-05 |
| lncRNA_012610 | Traes_1DL_2B473C495 | -0.994242 | 4.96E-05 |
| lncRNA_018111 | Traes_1DL_2B473C495 | -0.99296 | 7.42E-05 |
| lncRNA_033754 | Traes_1DL_2B473C495 | -0.994303 | 4.86E-05 |
| TRAES3BF053100050CFD_g | Traes_1DL_37A17C1AC | 0.9979164 | 6.51E-06 |
| lncRNA_013190 | Traes_1DL_37A17C1AC | 0.9962271 | 2.13E-05 |
| lncRNA_018078 | Traes_1DL_37A17C1AC | 0.9971188 | 1.24E-05 |
| lncRNA_043877 | Traes_1DL_37A17C1AC | 0.9915521 | 0.0001067 |
| lncRNA_051318 | Traes_1DL_37A17C1AC | 0.9934843 | 6.35E-05 |
| lncRNA_063127 | Traes_1DL_37A17C1AC | -0.990291 | 0.0001409 |
| lncRNA_080648 | Traes_1DL_37A17C1AC | 0.9933036 | 6.71E-05 |
| lncRNA_006011 | Traes_1DL_47D4F60E6 | 0.9925486 | 8.31E-05 |
| lncRNA_072440 | Traes_1DL_4A4BCC71B | 0.993416 | 6.49E-05 |
| lncRNA_022554 | Traes_1DL_4ACF54D50 | -0.992353 | 8.75E-05 |
| lncRNA_063009 | Traes_1DL_4ACF54D50 | -0.992537 | 8.33E-05 |
| lncRNA_013190 | Traes_1DL_4D7C10235 | 0.9925072 | 8.40E-05 |
| lncRNA_018078 | Traes_1DL_4D7C10235 | 0.9944312 | 4.64E-05 |
| lncRNA_048256 | Traes_1DL_4D7C10235 | 0.9931649 | 6.99E-05 |
| lncRNA_051318 | Traes_1DL_4D7C10235 | 0.9947333 | 4.15E-05 |
| lncRNA_000928 | Traes_1DL_53DDD6D20 | 0.9941605 | 5.11E-05 |
| lncRNA_029384 | Traes_1DL_53DDD6D20 | 0.9949373 | 3.84E-05 |
| lncRNA_000823 | Traes_1DL_54BFB7BDC | 0.9946633 | 4.26E-05 |
| lncRNA_020477 | Traes_1DL_580C04A3D | 0.9908021 | 0.0001265 |
| lncRNA_048320 | Traes_1DL_5DC4E9455 | 0.9936903 | 5.96E-05 |
| lncRNA_054824 | Traes_1DL_5DC4E9455 | 0.9941328 | 5.15E-05 |
| lncRNA_007700 | Traes_1DL_5F13FD194 | -0.993343 | 6.63E-05 |
| lncRNA_018078 | Traes_1DL_5F13FD194 | -0.993143 | 7.04E-05 |
| lncRNA_048256 | Traes_1DL_5F13FD194 | -0.99239 | 8.66E-05 |
| lncRNA_051318 | Traes_1DL_607C1A6E6 | -0.990555 | 0.0001334 |
| lncRNA_058136 | Traes_1DL_607C1A6E6 | 0.9978644 | 6.84E-06 |
| lncRNA_059554 | Traes_1DL_636878C41 | 0.991644 | 0.0001044 |
| lncRNA_054317 | Traes_1DL_645A2ECC0 | -0.995847 | 2.58E-05 |
| lncRNA_063009 | Traes_1DL_645A2ECC0 | -0.9923 | 8.87E-05 |
| lncRNA_019725 | Traes_1DL_6655FDC74 | -0.990754 | 0.0001278 |
| lncRNA_068995 | Traes_1DL_6B3479A5F | 0.9907882 | 0.0001269 |
| lncRNA_029088 | Traes_1DL_7277B4C11 | 0.9941179 | 5.18E-05 |
| lncRNA_006270 | Traes_1DL_80768AB26 | 0.993318 | 6.68E-05 |
| lncRNA_021433 | Traes_1DL_80768AB26 | 0.9934314 | 6.46E-05 |
| lncRNA_009698 | Traes_1DL_831559883 | 0.9928671 | 7.61E-05 |
| lncRNA_022554 | Traes_1DL_83E665871 | 0.9927197 | 7.93E-05 |
| lncRNA_049300 | Traes_1DL_847254606 | 0.9900596 | 0.0001477 |
| lncRNA_008977 | Traes_1DL_88DD1E468 | 0.9956586 | 2.82E-05 |
| lncRNA_047461 | Traes_1DL_88DD1E468 | 0.9902036 | 0.0001435 |
| lncRNA_063009 | Traes_1DL_88DD1E468 | 0.9956893 | 2.78E-05 |
| lncRNA_006859 | Traes_1DL_8C6B737E9 | 0.993604 | 6.12E-05 |
| lncRNA_014538 | Traes_1DL_8C6B737E9 | 0.9924802 | 8.46E-05 |
| lncRNA_059323 | Traes_1DL_8D89E0813 | -0.990494 | 0.0001351 |
| lncRNA_074658 | Traes_1DL_8D89E0813 | -0.991829 | 9.99E-05 |
| lncRNA_012242 | Traes_1DL_9926BBAE1 | -0.990042 | 0.0001483 |
| lncRNA_064639 | Traes_1DL_9926BBAE1 | -0.993296 | 6.73E-05 |
| TRAES3BF053100050CFD_g | Traes_1DL_9C07DF37D | 0.9902439 | 0.0001423 |
| lncRNA_034367 | Traes_1DL_A0EE6CD39 | 0.9955171 | 3.01E-05 |
| lncRNA_068995 | Traes_1DL_A6553EC96 | -0.992287 | 8.90E-05 |
| TRAES3BF052700300CFD_g | Traes_1DL_A9FBE73D6 | 0.9976801 | 8.07E-06 |
| lncRNA_014501 | Traes_1DL_A9FBE73D6 | 0.9938327 | 5.69E-05 |
| lncRNA_061738 | Traes_1DL_A9FBE73D6 | 0.9935039 | 6.32E-05 |
| lncRNA_022554 | Traes_1DL_ADAD51121 | 0.9912702 | 0.000114 |
| lncRNA_008977 | Traes_1DL_B25915381 | 0.9941527 | 5.12E-05 |
| lncRNA_013190 | Traes_1DL_CA3D844E5 | 0.9957369 | 2.72E-05 |
| lncRNA_018078 | Traes_1DL_CA3D844E5 | 0.9922793 | 8.92E-05 |
| lncRNA_022554 | Traes_1DL_CA3D844E5 | 0.9943707 | 4.74E-05 |
| lncRNA_048256 | Traes_1DL_CA3D844E5 | 0.994459 | 4.60E-05 |
| lncRNA_051318 | Traes_1DL_CA3D844E5 | 0.9917924 | 0.0001008 |
| lncRNA_013053 | Traes_1DL_CAA9298F0 | 0.9934715 | 6.38E-05 |
| lncRNA_048256 | Traes_1DL_D3AEF0EB6 | -0.99299 | 7.35E-05 |
| Traes_1BS_B58657408 | Traes_1DL_D5512C9E1 | 0.9956053 | 2.89E-05 |
| lncRNA_019725 | Traes_1DL_DD8D1FAF1 | -0.990004 | 0.0001494 |
| lncRNA_048668 | Traes_1DL_DD8D1FAF1 | 0.9918597 | 9.91E-05 |
| lncRNA_051888 | Traes_1DL_DD8D1FAF1 | -0.992013 | 9.54E-05 |
| lncRNA_009698 | Traes_1DL_E1AFC0004 | -0.994073 | 5.26E-05 |
| lncRNA_013190 | Traes_1DL_E1AFC0004 | -0.992219 | 9.06E-05 |
| lncRNA_018078 | Traes_1DL_E1AFC0004 | -0.990007 | 0.0001493 |
| lncRNA_019725 | Traes_1DL_E1AFC0004 | -0.996538 | 1.80E-05 |
| lncRNA_051318 | Traes_1DL_E1AFC0004 | -0.991056 | 0.0001196 |
| lncRNA_051551 | Traes_1DL_E217977A6 | 0.9922715 | 8.94E-05 |
| lncRNA_032048 | Traes_1DL_E219BD119 | -0.99171 | 0.0001028 |
| lncRNA_032048 | Traes_1DL_EC9A14EB3 | 0.9918461 | 9.95E-05 |
| lncRNA_081387 | Traes_1DL_ED54F5B27 | -0.994975 | 3.78E-05 |
| lncRNA_048668 | Traes_1DL_F0FE02681 | 0.9911355 | 0.0001175 |
| lncRNA_020959 | Traes_1DL_F3EBE6998 | 0.9975971 | 8.65E-06 |
| lncRNA_024807 | Traes_1DL_F3EBE6998 | 0.9955656 | 2.95E-05 |
| lncRNA_081387 | Traes_1DL_F6ABE8653 | -0.993086 | 7.15E-05 |
| lncRNA_029088 | Traes_1DL_FC0973C52 | 0.9902757 | 0.0001414 |
| TRAES3BF021800100CFD_g | Traes_1DS_042E4ED4C | 0.9936068 | 6.12E-05 |
| lncRNA_045725 | Traes_1DS_042E4ED4C | 0.9996628 | 1.71E-07 |
| lncRNA_051428 | Traes_1DS_042E4ED4C | 0.9996628 | 1.71E-07 |
| lncRNA_057614 | Traes_1DS_042E4ED4C | 0.9996628 | 1.71E-07 |
| lncRNA_065747 | Traes_1DS_042E4ED4C | 0.9996628 | 1.71E-07 |
| lncRNA_024152 | Traes_1DS_07AD88BDA | 0.9946262 | 4.32E-05 |
| TRAES3BF052700300CFD_g | Traes_1DS_35B7AED4C | -0.990339 | 0.0001395 |
| lncRNA_051318 | Traes_1DS_3C40BAEFD | -0.992642 | 8.10E-05 |
| lncRNA_006361 | Traes_1DS_3E8E0BE88 | 0.9960406 | 2.35E-05 |
| lncRNA_012610 | Traes_1DS_3E8E0BE88 | 0.9948835 | 3.92E-05 |
| lncRNA_015487 | Traes_1DS_3E8E0BE88 | 0.9903811 | 0.0001383 |
| lncRNA_051318 | Traes_1DS_3E8E0BE88 | 0.9901966 | 0.0001437 |
| lncRNA_024812 | Traes_1DS_4F51F6DFA | 0.9901618 | 0.0001447 |
| lncRNA_015322 | Traes_1DS_65C1FDCD8 | 0.9900434 | 0.0001482 |
| lncRNA_029528 | Traes_1DS_67E26A4F0 | -0.991017 | 0.0001207 |
| lncRNA_084019 | Traes_1DS_67E26A4F0 | -0.99075 | 0.0001279 |
| lncRNA_013231 | Traes_1DS_69740CF6C | 0.9954863 | 3.05E-05 |
| TRAES3BF053100050CFD_g | Traes_1DS_6F9483CAA | 0.9989969 | 1.51E-06 |
| lncRNA_007700 | Traes_1DS_6F9483CAA | 0.9916364 | 0.0001046 |
| lncRNA_013190 | Traes_1DS_6F9483CAA | 0.9947831 | 4.08E-05 |
| lncRNA_018078 | Traes_1DS_6F9483CAA | 0.9985924 | 2.97E-06 |
| lncRNA_043877 | Traes_1DS_6F9483CAA | 0.9958646 | 2.56E-05 |
| lncRNA_051318 | Traes_1DS_6F9483CAA | 0.9900054 | 0.0001493 |
| lncRNA_080648 | Traes_1DS_6F9483CAA | 0.9941574 | 5.11E-05 |
| lncRNA_057390 | Traes_1DS_7BED2191F | -0.992686 | 8.00E-05 |
| lncRNA_006011 | Traes_1DS_80F18285E | 0.9975535 | 8.97E-06 |
| lncRNA_059554 | Traes_1DS_8C0C4C146 | 0.9900105 | 0.0001492 |
| lncRNA_059976 | Traes_1DS_AD06EA5E4 | 0.99632 | 2.03E-05 |
| lncRNA_082364 | Traes_1DS_B1B88D99E | 0.9937439 | 5.86E-05 |
| lncRNA_001839 | Traes_1DS_B282DDC2B | -0.993976 | 5.43E-05 |
| lncRNA_000928 | Traes_1DS_B5F884A16 | 0.9907301 | 0.0001285 |
| lncRNA_019725 | Traes_1DS_B5F884A16 | -0.991253 | 0.0001144 |
| lncRNA_020477 | Traes_1DS_BF178847F | 0.9975392 | 9.08E-06 |
| lncRNA_029013 | Traes_1DS_C00F63EA4 | 0.9943135 | 4.84E-05 |
| lncRNA_064639 | Traes_1DS_CA4185D11 | 0.9952953 | 3.31E-05 |
| TRAES3BF052700300CFD_g | Traes_1DS_CE78BD7E3 | -0.990041 | 0.0001483 |
| lncRNA_008977 | Traes_1DS_CE78BD7E3 | -0.991725 | 0.0001024 |
| lncRNA_074837 | Traes_1DS_D0F1CEB10 | 0.9953345 | 3.26E-05 |
| lncRNA_056853 | Traes_1DS_D4FB403C4 | 0.9906439 | 0.0001309 |
| lncRNA_051318 | Traes_1DS_E55F00FDB | 0.993216 | 6.89E-05 |
| lncRNA_022554 | Traes_1DS_EEBA94F4D | -0.995987 | 2.41E-05 |
| lncRNA_048256 | Traes_1DS_EEBA94F4D | -0.9938 | 5.75E-05 |
| Traes_1BS_B58657408 | Traes_1DS_EFB39AEDC | -0.998425 | 3.72E-06 |
| lncRNA_014501 | Traes_1DS_F3A99134B | 0.9928136 | 7.73E-05 |
| lncRNA_024807 | Traes_1DS_F3A99134B | 0.9906659 | 0.0001303 |
| lncRNA_061738 | Traes_1DS_F3A99134B | 0.9917068 | 0.0001029 |
| lncRNA_048266 | Traes_1DS_F69AA50CA | -0.990613 | 0.0001318 |
| lncRNA_049300 | Traes_1DS_FD3F65B05 | 0.9934876 | 6.35E-05 |
| lncRNA_059323 | Traes_2AL_04F903351 | -0.992512 | 8.39E-05 |
| lncRNA_081387 | Traes_2AL_04F903351 | -0.994499 | 4.53E-05 |
| lncRNA_039943 | Traes_2AL_0A1F83B9E | 0.9990121 | 1.46E-06 |
| lncRNA_051888 | Traes_2AL_0A1F83B9E | -0.993372 | 6.58E-05 |
| lncRNA_064639 | Traes_2AL_0ADF02A1E | 0.9902154 | 0.0001431 |
| lncRNA_008500 | Traes_2AL_0D81DFB10 | 0.9963552 | 1.99E-05 |
| lncRNA_027360 | Traes_2AL_0F6C79FDA | 0.9902978 | 0.0001407 |
| lncRNA_074837 | Traes_2AL_1598A938B | 0.998223 | 4.73E-06 |
| lncRNA_074837 | Traes_2AL_1A5B6CF28 | 0.9929772 | 7.38E-05 |
| lncRNA_013231 | Traes_2AL_1F6431B47 | 0.9961974 | 2.17E-05 |
| lncRNA_009362 | Traes_2AL_22EFC1256 | -0.994607 | 4.35E-05 |
| lncRNA_045725 | Traes_2AL_264BF03F4 | 0.9946457 | 4.29E-05 |
| lncRNA_051428 | Traes_2AL_264BF03F4 | 0.9946457 | 4.29E-05 |
| lncRNA_057614 | Traes_2AL_264BF03F4 | 0.9946457 | 4.29E-05 |
| lncRNA_065747 | Traes_2AL_264BF03F4 | 0.9946457 | 4.29E-05 |
| lncRNA_056853 | Traes_2AL_2655769B3 | -0.996843 | 1.49E-05 |
| lncRNA_048256 | Traes_2AL_2683E57E5 | -0.992723 | 7.92E-05 |
| lncRNA_019725 | Traes_2AL_295E0C7C5 | 0.9913187 | 0.0001127 |
| lncRNA_048256 | Traes_2AL_295E0C7C5 | 0.9912755 | 0.0001138 |
| lncRNA_039943 | Traes_2AL_2B32DB242 | 0.9964531 | 1.88E-05 |
| lncRNA_053766 | Traes_2AL_2B32DB242 | 0.994543 | 4.46E-05 |
| lncRNA_020959 | Traes_2AL_2C50DEE4C | -0.996346 | 2.00E-05 |
| lncRNA_024807 | Traes_2AL_2C50DEE4C | -0.996529 | 1.81E-05 |
| lncRNA_044308 | Traes_2AL_2C50DEE4C | -0.995275 | 3.34E-05 |
| lncRNA_061738 | Traes_2AL_2C50DEE4C | -0.991163 | 0.0001168 |
| lncRNA_007700 | Traes_2AL_2FF604DA9 | 0.9901673 | 0.0001445 |
| lncRNA_018078 | Traes_2AL_2FF604DA9 | 0.9933323 | 6.65E-05 |
| lncRNA_022554 | Traes_2AL_2FF604DA9 | 0.9911207 | 0.0001179 |
| lncRNA_051318 | Traes_2AL_2FF604DA9 | 0.991344 | 0.0001121 |
| lncRNA_008977 | Traes_2AL_3C99F42A5 | -0.993068 | 7.19E-05 |
| lncRNA_007700 | Traes_2AL_3E61D4DE2 | 0.992512 | 8.39E-05 |
| lncRNA_018078 | Traes_2AL_3E61D4DE2 | 0.9919897 | 9.60E-05 |
| lncRNA_029013 | Traes_2AL_3E61D4DE2 | 0.9947799 | 4.08E-05 |
| lncRNA_063127 | Traes_2AL_3E61D4DE2 | -0.993642 | 6.05E-05 |
| lncRNA_020477 | Traes_2AL_3FE5DF00C | 0.9928295 | 7.69E-05 |
| lncRNA_047461 | Traes_2AL_3FE5DF00C | -0.994269 | 4.92E-05 |
| lncRNA_072440 | Traes_2AL_411B944D6 | 0.9927076 | 7.96E-05 |
| Traes_1BS_B58657408 | Traes_2AL_47B1A5BF2 | -0.992278 | 8.92E-05 |
| lncRNA_072167 | Traes_2AL_4E69B4802 | -0.993182 | 6.96E-05 |
| lncRNA_007700 | Traes_2AL_56F4960EB | -0.992369 | 8.71E-05 |
| TRAES3BF052700300CFD_g | Traes_2AL_57C3C7FAC | -0.990064 | 0.0001476 |
| lncRNA_022554 | Traes_2AL_57C3C7FAC | -0.993992 | 5.40E-05 |
| TRAES3BF053100050CFD_g | Traes_2AL_5925A665E | 0.996521 | 1.81E-05 |
| lncRNA_007700 | Traes_2AL_5925A665E | 0.9944613 | 4.59E-05 |
| lncRNA_018078 | Traes_2AL_5925A665E | 0.9981663 | 5.04E-06 |
| lncRNA_043877 | Traes_2AL_5925A665E | 0.9965786 | 1.75E-05 |
| lncRNA_063127 | Traes_2AL_5925A665E | -0.994004 | 5.38E-05 |
| lncRNA_080648 | Traes_2AL_5925A665E | 0.9958716 | 2.55E-05 |
| lncRNA_008977 | Traes_2AL_5C7E76139 | 0.9904149 | 0.0001374 |
| lncRNA_012242 | Traes_2AL_5FF7D6940 | 0.9902961 | 0.0001408 |
| lncRNA_006859 | Traes_2AL_65B300237 | 0.9912043 | 0.0001157 |
| lncRNA_071124 | Traes_2AL_6DD37E6BE | 0.9983995 | 3.84E-06 |
| TRAES3BF021800100CFD_g | Traes_2AL_71D9D4D71 | 0.9959807 | 2.42E-05 |
| lncRNA_045725 | Traes_2AL_71D9D4D71 | 0.998779 | 2.24E-06 |
| lncRNA_051428 | Traes_2AL_71D9D4D71 | 0.998779 | 2.24E-06 |
| lncRNA_057614 | Traes_2AL_71D9D4D71 | 0.998779 | 2.24E-06 |
| lncRNA_065747 | Traes_2AL_71D9D4D71 | 0.998779 | 2.24E-06 |
| lncRNA_008977 | Traes_2AL_805027C23 | 0.9905536 | 0.0001334 |
| lncRNA_048256 | Traes_2AL_820ED5DD81 | -0.991995 | 9.59E-05 |
| lncRNA_008500 | Traes_2AL_8DF1244C4 | 0.9935806 | 6.17E-05 |
| lncRNA_005462 | Traes_2AL_A38FEE372 | 0.9952413 | 3.39E-05 |
| lncRNA_054824 | Traes_2AL_A38FEE372 | 0.9944455 | 4.62E-05 |
| lncRNA_081520 | Traes_2AL_A38FEE372 | 0.9964709 | 1.87E-05 |
| lncRNA_019725 | Traes_2AL_A47310DDA | 0.9929068 | 7.53E-05 |
| lncRNA_043877 | Traes_2AL_A47310DDA | 0.9948636 | 3.95E-05 |
| TRAES3BF052700300CFD_g | Traes_2AL_A7198751F | 0.9938862 | 5.60E-05 |
| lncRNA_061738 | Traes_2AL_A7198751F | 0.9909564 | 0.0001223 |
| lncRNA_017776 | Traes_2AL_AAD8D4641 | -0.993244 | 6.83E-05 |
| lncRNA_032897 | Traes_2AL_B4A22DC4F | 0.9928912 | 7.56E-05 |
| TRAES3BF053100050CFD_g | Traes_2AL_B854D3399 | 0.9940794 | 5.25E-05 |
| lncRNA_007700 | Traes_2AL_B854D3399 | 0.9914411 | 0.0001096 |
| lncRNA_013190 | Traes_2AL_B854D3399 | 0.9954484 | 3.10E-05 |
| lncRNA_018078 | Traes_2AL_B854D3399 | 0.9997162 | 1.21E-07 |
| lncRNA_043877 | Traes_2AL_B854D3399 | 0.9925329 | 8.34E-05 |
| lncRNA_051318 | Traes_2AL_B854D3399 | 0.9956233 | 2.87E-05 |
| lncRNA_063127 | Traes_2AL_B854D3399 | -0.993625 | 6.08E-05 |
| lncRNA_049300 | Traes_2AL_BB6B0F4CB | 0.9910225 | 0.0001205 |
| lncRNA_051888 | Traes_2AL_BBDE2485C | -0.991323 | 0.0001126 |
| lncRNA_058136 | Traes_2AL_BBDE2485C | 0.9938831 | 5.60E-05 |
| lncRNA_010675 | Traes_2AL_C69D52D2E | -0.995847 | 2.58E-05 |
| lncRNA_015322 | Traes_2AL_C97ED96AA | 0.9911134 | 0.0001181 |
| lncRNA_047317 | Traes_2AL_CE06F37FB | 0.9912957 | 0.0001133 |
| lncRNA_048419 | Traes_2AL_CE06F37FB | 0.9902767 | 0.0001414 |
| lncRNA_048256 | Traes_2AL_D0C3751FD | 0.9979826 | 6.10E-06 |
| lncRNA_015487 | Traes_2AL_D344EC2AF | -0.991917 | 9.77E-05 |
| lncRNA_051318 | Traes_2AL_D344EC2AF | -0.990926 | 0.0001231 |
| lncRNA_013190 | Traes_2AL_DE86B9FD4 | 0.9968347 | 1.50E-05 |
| lncRNA_018078 | Traes_2AL_DE86B9FD4 | 0.9935593 | 6.21E-05 |
| lncRNA_048256 | Traes_2AL_DE86B9FD4 | 0.9932067 | 6.91E-05 |
| lncRNA_051318 | Traes_2AL_DE86B9FD4 | 0.9976042 | 8.60E-06 |
| lncRNA_008977 | Traes_2AL_EC86F9FE7 | -0.994769 | 4.10E-05 |
| lncRNA_063009 | Traes_2AL_EC86F9FE7 | -0.994984 | 3.77E-05 |
| lncRNA_000928 | Traes_2AL_F53EDA4C9 | -0.996633 | 1.70E-05 |
| lncRNA_064639 | Traes_2AL_F56A10D11 | -0.997643 | 8.32E-06 |
| lncRNA_006361 | Traes_2AL_FB92D7385 | -0.990181 | 0.0001441 |
| lncRNA_008977 | Traes_2AS_09C4D333F | -0.990851 | 0.0001252 |
| lncRNA_048668 | Traes_2AS_105B1A29A | 0.9946079 | 4.35E-05 |
| lncRNA_010675 | Traes_2AS_106401B5B | 0.9911984 | 0.0001159 |
| lncRNA_048668 | Traes_2AS_106401B5B | 0.9904979 | 0.000135 |
| lncRNA_000928 | Traes_2AS_14EB16865 | -0.998258 | 4.55E-06 |
| lncRNA_009698 | Traes_2AS_14EB16865 | 0.9909912 | 0.0001214 |
| lncRNA_019748 | Traes_2AS_20AD90CA61 | -0.994932 | 3.85E-05 |
| TRAES3BF177500010CFD_g | Traes_2AS_216467F1E | 0.992045 | 9.47E-05 |
| lncRNA_022554 | Traes_2AS_235EEA0AE | -0.991076 | 0.0001191 |
| lncRNA_063009 | Traes_2AS_235EEA0AE | -0.992626 | 8.14E-05 |
| TRAES3BF052700300CFD_g | Traes_2AS_25AACE7BC | 0.9930755 | 7.18E-05 |
| lncRNA_061738 | Traes_2AS_25AACE7BC | 0.9936676 | 6.00E-05 |
| TRAES3BF053100050CFD_g | Traes_2AS_28B3FAEB3 | -0.998164 | 5.05E-06 |
| lncRNA_007700 | Traes_2AS_28B3FAEB3 | -0.992218 | 9.06E-05 |
| lncRNA_013190 | Traes_2AS_28B3FAEB3 | -0.99422 | 5.00E-05 |
| lncRNA_018078 | Traes_2AS_28B3FAEB3 | -0.996901 | 1.44E-05 |
| lncRNA_043877 | Traes_2AS_28B3FAEB3 | -0.990645 | 0.0001309 |
| lncRNA_080648 | Traes_2AS_28B3FAEB3 | -0.991732 | 0.0001023 |
| lncRNA_074434 | Traes_2AS_3144FD589 | 0.9920583 | 9.44E-05 |
| lncRNA_000928 | Traes_2AS_32FC7017A | 0.9902238 | 0.0001429 |
| lncRNA_015322 | Traes_2AS_32FC7017A | 0.9989856 | 1.54E-06 |
| lncRNA_019725 | Traes_2AS_32FC7017A | -0.994423 | 4.66E-05 |
| lncRNA_047461 | Traes_2AS_34272605C | -0.990028 | 0.0001487 |
| TRAES3BF052700300CFD_g | Traes_2AS_3B0E92500 | 0.9904915 | 0.0001352 |
| lncRNA_063009 | Traes_2AS_3B0E92500 | 0.9906604 | 0.0001304 |
| TRAES3BF051200090CFD_g | Traes_2AS_3BB2D3EC0 | 0.9940666 | 5.27E-05 |
| lncRNA_015487 | Traes_2AS_3F25BD910 | -0.990064 | 0.0001476 |
| lncRNA_018111 | Traes_2AS_3F25BD910 | -0.991034 | 0.0001202 |
| lncRNA_047461 | Traes_2AS_3F25BD910 | -0.992199 | 9.10E-05 |
| lncRNA_063009 | Traes_2AS_3F25BD910 | -0.994346 | 4.79E-05 |
| lncRNA_068995 | Traes_2AS_3F458D2CF | 0.9900549 | 0.0001479 |
| lncRNA_015487 | Traes_2AS_467FD5266 | -0.993129 | 7.06E-05 |
| lncRNA_014501 | Traes_2AS_484015532 | 0.9909218 | 0.0001232 |
| lncRNA_024807 | Traes_2AS_484015532 | 0.9927445 | 7.88E-05 |
| lncRNA_039943 | Traes_2AS_4C3C5E990 | 0.9903021 | 0.0001406 |
| lncRNA_053766 | Traes_2AS_4C3C5E990 | 0.9912583 | 0.0001143 |
| lncRNA_074658 | Traes_2AS_537EEA6B2 | -0.995013 | 3.72E-05 |
| lncRNA_007700 | Traes_2AS_5810DD9381 | 0.9905123 | 0.0001346 |
| lncRNA_059554 | Traes_2AS_5810DD9381 | 0.9958538 | 2.58E-05 |
| lncRNA_024812 | Traes_2AS_5884229E4 | 0.9945953 | 4.37E-05 |
| lncRNA_020477 | Traes_2AS_5A5258192 | 0.9942527 | 4.95E-05 |
| lncRNA_047461 | Traes_2AS_5A5258192 | -0.991302 | 0.0001131 |
| lncRNA_048668 | Traes_2AS_5A5258192 | 0.9949015 | 3.89E-05 |
| lncRNA_006011 | Traes_2AS_5C7DAFECA | 0.9953301 | 3.27E-05 |
| TRAES3BF026700070CFD_g | Traes_2AS_6345FCE27 | 0.9910175 | 0.0001207 |
| lncRNA_054623 | Traes_2AS_6345FCE27 | 0.9931378 | 7.05E-05 |
| lncRNA_066190 | Traes_2AS_6345FCE27 | 0.9913029 | 0.0001131 |
| lncRNA_024812 | Traes_2AS_64E41196E | 0.9940695 | 5.27E-05 |
| lncRNA_013053 | Traes_2AS_6AB3D73F7 | 0.994546 | 4.45E-05 |
| lncRNA_000928 | Traes_2AS_6CCC35B60 | 0.9975238 | 9.19E-06 |
| lncRNA_039943 | Traes_2AS_6CCC35B60 | 0.9930432 | 7.24E-05 |
| lncRNA_058136 | Traes_2AS_6CCC35B60 | 0.9923231 | 8.82E-05 |
| lncRNA_024152 | Traes_2AS_6F23A3584 | -0.997454 | 9.72E-06 |
| lncRNA_051318 | Traes_2AS_71B3134F6 | -0.991691 | 0.0001033 |
| lncRNA_048256 | Traes_2AS_73971B9C1 | -0.991766 | 0.0001014 |
| lncRNA_008500 | Traes_2AS_76163A005 | -0.990777 | 0.0001272 |
| lncRNA_022554 | Traes_2AS_78A7576A9 | 0.9909333 | 0.0001229 |
| lncRNA_024152 | Traes_2AS_7FA697DCF | 0.9943247 | 4.82E-05 |
| lncRNA_000833 | Traes_2AS_8558C34D7 | 0.9927166 | 7.94E-05 |
| lncRNA_021433 | Traes_2AS_8AA970F1F1 | 0.9922649 | 8.95E-05 |
| lncRNA_039943 | Traes_2AS_96DEC01C0 | -0.99583 | 2.60E-05 |
| lncRNA_012608 | Traes_2AS_97F57A014 | 0.9928905 | 7.56E-05 |
| lncRNA_000928 | Traes_2AS_A3953C85E | 0.9920845 | 9.37E-05 |
| lncRNA_058136 | Traes_2AS_A3953C85E | 0.9988658 | 1.93E-06 |
| TRAES3BF053100050CFD_g | Traes_2AS_A75E5ABF9 | -0.990863 | 0.0001249 |
| lncRNA_018078 | Traes_2AS_A75E5ABF9 | -0.992981 | 7.37E-05 |
| lncRNA_043877 | Traes_2AS_A75E5ABF9 | -0.993602 | 6.13E-05 |
| lncRNA_051318 | Traes_2AS_A75E5ABF9 | -0.990288 | 0.000141 |
| lncRNA_063127 | Traes_2AS_A75E5ABF9 | 0.9916872 | 0.0001034 |
| lncRNA_080648 | Traes_2AS_A75E5ABF9 | -0.991978 | 9.63E-05 |
| lncRNA_048266 | Traes_2AS_AA5E1E20D | -0.992542 | 8.32E-05 |
| lncRNA_072935 | Traes_2AS_AA5E1E20D | 0.99191 | 9.79E-05 |
| lncRNA_020477 | Traes_2AS_AA84E72D4 | 0.9910469 | 0.0001199 |
| lncRNA_048668 | Traes_2AS_AA84E72D4 | 0.9942267 | 4.99E-05 |
| lncRNA_053766 | Traes_2AS_AA84E72D4 | 0.9914872 | 0.0001084 |
| lncRNA_051888 | Traes_2AS_AFA56788E | 0.993166 | 6.99E-05 |
| lncRNA_058136 | Traes_2AS_AFA56788E | -0.995781 | 2.67E-05 |
| lncRNA_010675 | Traes_2AS_B17A4F9EF | 0.9939317 | 5.51E-05 |
| lncRNA_021433 | Traes_2AS_BF5FA8832 | 0.9906045 | 0.000132 |
| lncRNA_044556 | Traes_2AS_BF5FA8832 | 0.9902494 | 0.0001421 |
| lncRNA_061738 | Traes_2AS_BF5FA8832 | 0.9925155 | 8.38E-05 |
| lncRNA_039420 | Traes_2AS_C205A90BA | -0.990493 | 0.0001351 |
| lncRNA_006361 | Traes_2AS_C69231934 | -0.998661 | 2.69E-06 |
| lncRNA_012610 | Traes_2AS_C69231934 | -0.990472 | 0.0001358 |
| lncRNA_015487 | Traes_2AS_C69231934 | -0.995103 | 3.59E-05 |
| lncRNA_018111 | Traes_2AS_C69231934 | -0.993128 | 7.07E-05 |
| TRAES3BF117100150CFD_g | Traes_2AS_CBF2D98D1 | 0.9902185 | 0.000143 |
| TRAES3BF051200090CFD_g | Traes_2AS_CD170CE8C | 0.9946226 | 4.33E-05 |
| lncRNA_005569 | Traes_2AS_CD170CE8C | 0.9976988 | 7.94E-06 |
| lncRNA_072440 | Traes_2AS_D86455172 | 0.99689 | 1.45E-05 |
| lncRNA_029088 | Traes_2AS_DE0D48CF6 | 0.9904505 | 0.0001364 |
| Traes_1BS_B58657408 | Traes_2AS_DE74C38CD | -0.99156 | 0.0001065 |
| lncRNA_039420 | Traes_2AS_DE74C38CD | -0.992201 | 9.10E-05 |
| lncRNA_020477 | Traes_2AS_DFDA79E58 | 0.9927657 | 7.83E-05 |
| lncRNA_024807 | Traes_2AS_EEADD06D6 | 0.9900074 | 0.0001493 |
| lncRNA_061738 | Traes_2AS_EEADD06D6 | 0.9949656 | 3.80E-05 |
| lncRNA_076127 | Traes_2AS_F46B5FF2A | 0.9908961 | 0.0001239 |
| lncRNA_020959 | Traes_2AS_FA532A2B7 | 0.9909429 | 0.0001227 |
| lncRNA_029013 | Traes_2AS_FA532A2B7 | 0.9957855 | 2.66E-05 |
| lncRNA_074165 | Traes_2AS_FFB730BC6 | -0.990301 | 0.0001407 |
| lncRNA_014501 | Traes_2BL_01FC4C993 | -0.997981 | 6.11E-06 |
| lncRNA_047461 | Traes_2BL_01FC4C993 | -0.991838 | 9.97E-05 |
| lncRNA_061738 | Traes_2BL_01FC4C993 | -0.992609 | 8.17E-05 |
| lncRNA_037690 | Traes_2BL_08FC042C0 | 0.9942681 | 4.92E-05 |
| lncRNA_074658 | Traes_2BL_08FC042C0 | 0.9910045 | 0.000121 |
| lncRNA_059323 | Traes_2BL_0A7F21A11 | -0.994176 | 5.08E-05 |
| lncRNA_046989 | Traes_2BL_0B80B3876 | 0.998347 | 4.10E-06 |
| lncRNA_043877 | Traes_2BL_0D44F4C96 | -0.991641 | 0.0001045 |
| lncRNA_063127 | Traes_2BL_0D44F4C96 | 0.9933419 | 6.63E-05 |
| lncRNA_080648 | Traes_2BL_0D44F4C96 | -0.995423 | 3.14E-05 |
| lncRNA_017777 | Traes_2BL_17348AA3D | 0.9923769 | 8.69E-05 |
| lncRNA_039943 | Traes_2BL_1D68D06FF | -0.993084 | 7.16E-05 |
| lncRNA_056853 | Traes_2BL_1EC425C26 | 0.9976293 | 8.42E-06 |
| lncRNA_006270 | Traes_2BL_253BA6454 | 0.9905128 | 0.0001346 |
| lncRNA_022554 | Traes_2BL_2825A3D0F | -0.991751 | 0.0001018 |
| lncRNA_013190 | Traes_2BL_28A7E0BF8 | 0.9908786 | 0.0001244 |
| lncRNA_018078 | Traes_2BL_28A7E0BF8 | 0.9904912 | 0.0001352 |
| lncRNA_048256 | Traes_2BL_28A7E0BF8 | 0.9945028 | 4.52E-05 |
| lncRNA_051318 | Traes_2BL_28A7E0BF8 | 0.9923209 | 8.82E-05 |
| lncRNA_018111 | Traes_2BL_28B398D53 | 0.9937735 | 5.80E-05 |
| lncRNA_024152 | Traes_2BL_28B398D53 | 0.995757 | 2.70E-05 |
| lncRNA_009698 | Traes_2BL_2A98E7B29 | 0.9963395 | 2.01E-05 |
| lncRNA_009698 | Traes_2BL_2BE05F104 | -0.993857 | 5.65E-05 |
| lncRNA_013190 | Traes_2BL_2BE05F104 | -0.994101 | 5.21E-05 |
| lncRNA_018078 | Traes_2BL_2BE05F104 | -0.992197 | 9.11E-05 |
| lncRNA_019725 | Traes_2BL_2BE05F104 | -0.995045 | 3.68E-05 |
| lncRNA_048256 | Traes_2BL_2BE05F104 | -0.990664 | 0.0001303 |
| lncRNA_051318 | Traes_2BL_2BE05F104 | -0.992046 | 9.46E-05 |
| lncRNA_076127 | Traes_2BL_2D4F1DB93 | 0.9914876 | 0.0001084 |
| TRAES3BF053100050CFD_g | Traes_2BL_2FF046824 | 0.9961526 | 2.22E-05 |
| lncRNA_013190 | Traes_2BL_2FF046824 | 0.9942668 | 4.92E-05 |
| lncRNA_018078 | Traes_2BL_2FF046824 | 0.9977025 | 7.91E-06 |
| lncRNA_043877 | Traes_2BL_2FF046824 | 0.9924821 | 8.46E-05 |
| lncRNA_051318 | Traes_2BL_2FF046824 | 0.9954287 | 3.13E-05 |
| lncRNA_063127 | Traes_2BL_2FF046824 | -0.994628 | 4.32E-05 |
| lncRNA_080648 | Traes_2BL_2FF046824 | 0.9944503 | 4.61E-05 |
| lncRNA_024807 | Traes_2BL_3041037F1 | -0.992525 | 8.36E-05 |
| lncRNA_046989 | Traes_2BL_342BDEA35 | -0.991526 | 0.0001074 |
| TRAES3BF052700300CFD_g | Traes_2BL_34819D129 | 0.9913365 | 0.0001123 |
| lncRNA_014501 | Traes_2BL_34819D129 | 0.9932112 | 6.90E-05 |
| lncRNA_024807 | Traes_2BL_34819D129 | 0.9913847 | 0.000111 |
| lncRNA_061738 | Traes_2BL_34819D129 | 0.9944133 | 4.67E-05 |
| lncRNA_000928 | Traes_2BL_350AC2704 | -0.991903 | 9.81E-05 |
| lncRNA_039943 | Traes_2BL_350AC2704 | -0.995815 | 2.62E-05 |
| lncRNA_024812 | Traes_2BL_36CE51AA2 | -0.99733 | 1.07E-05 |
| lncRNA_076127 | Traes_2BL_41F1A5E5F | 0.9918296 | 9.99E-05 |
| TRAES3BF052700300CFD_g | Traes_2BL_433D3147C | -0.99193 | 9.74E-05 |
| lncRNA_014501 | Traes_2BL_433D3147C | -0.996347 | 2.00E-05 |
| lncRNA_024807 | Traes_2BL_433D3147C | -0.990641 | 0.000131 |
| lncRNA_061738 | Traes_2BL_433D3147C | -0.997844 | 6.96E-06 |
| lncRNA_014538 | Traes_2BL_4A529675B | 0.9913027 | 0.0001131 |
| lncRNA_064639 | Traes_2BL_4AAB8719F | 0.9971399 | 1.23E-05 |
| lncRNA_044308 | Traes_2BL_4AEA2109C | -0.990546 | 0.0001336 |
| lncRNA_045725 | Traes_2BL_4B8B77E73 | 0.9942678 | 4.92E-05 |
| lncRNA_051428 | Traes_2BL_4B8B77E73 | 0.9942678 | 4.92E-05 |
| lncRNA_057614 | Traes_2BL_4B8B77E73 | 0.9942678 | 4.92E-05 |
| lncRNA_065747 | Traes_2BL_4B8B77E73 | 0.9942678 | 4.92E-05 |
| lncRNA_039943 | Traes_2BL_4DE8A07B7 | -0.997612 | 8.55E-06 |
| lncRNA_048668 | Traes_2BL_4E0157603 | -0.994987 | 3.76E-05 |
| lncRNA_053766 | Traes_2BL_4E0157603 | -0.992859 | 7.63E-05 |
| lncRNA_048668 | Traes_2BL_4EA417A3A | 0.9989372 | 1.69E-06 |
| lncRNA_053766 | Traes_2BL_4EA417A3A | 0.991875 | 9.88E-05 |
| lncRNA_006011 | Traes_2BL_52F8DB0D8 | 0.9923197 | 8.83E-05 |
| lncRNA_047317 | Traes_2BL_568EC47D9 | 0.9945096 | 4.51E-05 |
| lncRNA_014504 | Traes_2BL_59D694CE3 | -0.990463 | 0.000136 |
| lncRNA_018078 | Traes_2BL_59D694CE3 | -0.990939 | 0.0001228 |
| lncRNA_029013 | Traes_2BL_59D694CE3 | -0.990512 | 0.0001346 |
| lncRNA_063127 | Traes_2BL_59D694CE3 | 0.9976915 | 7.99E-06 |
| lncRNA_080648 | Traes_2BL_59D694CE3 | -0.992736 | 7.90E-05 |
| lncRNA_063127 | Traes_2BL_5A8297D8E | 0.9938456 | 5.67E-05 |
| lncRNA_007700 | Traes_2BL_5C9966D42 | 0.9941966 | 5.04E-05 |
| lncRNA_013190 | Traes_2BL_5C9966D42 | 0.9939865 | 5.41E-05 |
| lncRNA_018078 | Traes_2BL_5C9966D42 | 0.9969791 | 1.37E-05 |
| lncRNA_048256 | Traes_2BL_5C9966D42 | 0.9933808 | 6.56E-05 |
| lncRNA_051318 | Traes_2BL_5C9966D42 | 0.9924305 | 8.57E-05 |
| lncRNA_020959 | Traes_2BL_5DFE6B3311 | 0.9937036 | 5.93E-05 |
| lncRNA_024807 | Traes_2BL_5DFE6B3311 | 0.9923983 | 8.65E-05 |
| lncRNA_029013 | Traes_2BL_5DFE6B3311 | 0.990891 | 0.0001241 |
| lncRNA_068995 | Traes_2BL_5FD649D78 | -0.998903 | 1.81E-06 |
| lncRNA_022554 | Traes_2BL_62EEAC62E | -0.993855 | 5.65E-05 |
| lncRNA_051888 | Traes_2BL_63CB9A0E1 | 0.9961961 | 2.17E-05 |
| lncRNA_021433 | Traes_2BL_6552196A1 | -0.991663 | 0.000104 |
| lncRNA_008977 | Traes_2BL_6707649D3 | -0.99416 | 5.11E-05 |
| lncRNA_012242 | Traes_2BL_6707649D3 | -0.991444 | 0.0001095 |
| lncRNA_054317 | Traes_2BL_6707649D3 | -0.996712 | 1.62E-05 |
| lncRNA_063009 | Traes_2BL_6707649D3 | -0.995646 | 2.84E-05 |
| lncRNA_029528 | Traes_2BL_690728F61 | -0.993414 | 6.49E-05 |
| lncRNA_020959 | Traes_2BL_78C207CFC | 0.9984346 | 3.67E-06 |
| lncRNA_024807 | Traes_2BL_78C207CFC | 0.9971693 | 1.20E-05 |
| lncRNA_020959 | Traes_2BL_79FE1CC8F | -0.993451 | 6.42E-05 |
| lncRNA_024807 | Traes_2BL_79FE1CC8F | -0.993835 | 5.69E-05 |
| TRAES3BF024700270CFD_g | Traes_2BL_7CD79735B | 0.9907005 | 0.0001293 |
| lncRNA_006859 | Traes_2BL_7CD79735B | 0.9904534 | 0.0001363 |
| lncRNA_012608 | Traes_2BL_7CD79735B | 0.9907337 | 0.0001284 |
| lncRNA_001839 | Traes_2BL_7D2114731 | 0.9953942 | 3.18E-05 |
| lncRNA_020477 | Traes_2BL_84B12F4F8 | 0.9929857 | 7.36E-05 |
| lncRNA_048668 | Traes_2BL_84B12F4F8 | 0.9983263 | 4.20E-06 |
| lncRNA_048256 | Traes_2BL_8672D898D | 0.9931465 | 7.03E-05 |
| lncRNA_059554 | Traes_2BL_8672D898D | 0.9925932 | 8.21E-05 |
| lncRNA_072440 | Traes_2BL_8FED05903 | 0.9929888 | 7.36E-05 |
| lncRNA_076127 | Traes_2BL_94872CC98 | 0.9904584 | 0.0001361 |
| lncRNA_039420 | Traes_2BL_95044D775 | 0.9907897 | 0.0001269 |
| lncRNA_044308 | Traes_2BL_95044D775 | 0.9904801 | 0.0001355 |
| lncRNA_064639 | Traes_2BL_9E4DA3DB5 | 0.9969409 | 1.40E-05 |
| TRAES3BF052700300CFD_g | Traes_2BL_A402261EC1 | -0.994943 | 3.83E-05 |
| lncRNA_048266 | Traes_2BL_A4574D311 | -0.999663 | 1.70E-07 |
| lncRNA_072935 | Traes_2BL_AAE4048B6 | 0.9933788 | 6.56E-05 |
| lncRNA_008500 | Traes_2BL_B0D2559B2 | -0.99186 | 9.91E-05 |
| lncRNA_047317 | Traes_2BL_B57DA0C02 | 0.9935709 | 6.19E-05 |
| lncRNA_048419 | Traes_2BL_B57DA0C02 | 0.9902878 | 0.000141 |
| lncRNA_046989 | Traes_2BL_B805C339B | -0.992233 | 9.02E-05 |
| lncRNA_009698 | Traes_2BL_BD8DBA189 | 0.9957636 | 2.69E-05 |
| lncRNA_013190 | Traes_2BL_BD8DBA189 | 0.9943046 | 4.86E-05 |
| lncRNA_048256 | Traes_2BL_BD8DBA189 | 0.9956156 | 2.88E-05 |
| lncRNA_039803 | Traes_2BL_C2B32378B | 0.9961528 | 2.22E-05 |
| lncRNA_020477 | Traes_2BL_C433BB333 | -0.993593 | 6.15E-05 |
| lncRNA_024152 | Traes_2BL_C433BB333 | 0.9919917 | 9.59E-05 |
| lncRNA_020477 | Traes_2BL_C6677B603 | 0.9936347 | 6.06E-05 |
| lncRNA_047461 | Traes_2BL_C6677B603 | -0.993723 | 5.90E-05 |
| lncRNA_048668 | Traes_2BL_C6677B603 | 0.9942072 | 5.02E-05 |
| lncRNA_033754 | Traes_2BL_C9F83BF85 | -0.992264 | 8.95E-05 |
| lncRNA_064639 | Traes_2BL_C9F83BF85 | -0.99389 | 5.59E-05 |
| lncRNA_007700 | Traes_2BL_CA5412466 | 0.995862 | 2.56E-05 |
| lncRNA_018078 | Traes_2BL_CA5412466 | 0.9955383 | 2.98E-05 |
| lncRNA_074837 | Traes_2BL_CDF259349 | 0.9939463 | 5.49E-05 |
| lncRNA_029088 | Traes_2BL_CF47A74CC | 0.9932606 | 6.80E-05 |
| lncRNA_016209 | Traes_2BL_D12DCF896 | 0.9934962 | 6.33E-05 |
| lncRNA_073681 | Traes_2BL_D12DCF896 | 0.9942837 | 4.89E-05 |
| lncRNA_074165 | Traes_2BL_D12DCF896 | 0.9934131 | 6.49E-05 |
| lncRNA_006361 | Traes_2BL_D506EE7D2 | -0.996709 | 1.62E-05 |
| lncRNA_015487 | Traes_2BL_D506EE7D2 | -0.995066 | 3.65E-05 |
| lncRNA_001839 | Traes_2BL_D5FCF8120 | -0.997442 | 9.81E-06 |
| lncRNA_022554 | Traes_2BL_D94A13D35 | -0.994731 | 4.16E-05 |
| lncRNA_014501 | Traes_2BL_DA7C50D42 | 0.993048 | 7.23E-05 |
| lncRNA_024807 | Traes_2BL_DA7C50D42 | 0.9910428 | 0.00012 |
| lncRNA_047461 | Traes_2BL_DA7C50D42 | 0.9903467 | 0.0001393 |
| lncRNA_061738 | Traes_2BL_DA7C50D42 | 0.9926977 | 7.98E-05 |
| lncRNA_013190 | Traes_2BL_DCAB32D3D | -0.995097 | 3.60E-05 |
| lncRNA_018078 | Traes_2BL_DCAB32D3D | -0.994385 | 4.72E-05 |
| lncRNA_051318 | Traes_2BL_DCAB32D3D | -0.998193 | 4.89E-06 |
| lncRNA_000928 | Traes_2BL_E507901E4 | -0.995412 | 3.15E-05 |
| lncRNA_015322 | Traes_2BL_E507901E4 | -0.994547 | 4.45E-05 |
| lncRNA_019725 | Traes_2BL_E507901E4 | 0.9939937 | 5.40E-05 |
| lncRNA_013231 | Traes_2BL_E5A7188DB | 0.9973207 | 1.08E-05 |
| lncRNA_082364 | Traes_2BL_E5A7188DB | 0.9900237 | 0.0001488 |
| lncRNA_032897 | Traes_2BL_E9C8CE71F | 0.9925047 | 8.41E-05 |
| lncRNA_051888 | Traes_2BL_E9FB5127B | -0.997414 | 1.00E-05 |
| lncRNA_058136 | Traes_2BL_E9FB5127B | 0.9945036 | 4.52E-05 |
| TRAES3BF021800100CFD_g | Traes_2BL_EC520A25A | 0.9951678 | 3.50E-05 |
| lncRNA_045725 | Traes_2BL_EC520A25A | 0.9991693 | 1.03E-06 |
| lncRNA_051428 | Traes_2BL_EC520A25A | 0.9991693 | 1.03E-06 |
| lncRNA_057614 | Traes_2BL_EC520A25A | 0.9991693 | 1.03E-06 |
| lncRNA_065747 | Traes_2BL_EC520A25A | 0.9991693 | 1.03E-06 |
| lncRNA_048668 | Traes_2BL_EF68D6200 | 0.9961099 | 2.27E-05 |
| lncRNA_053766 | Traes_2BL_EF68D6200 | 0.9946671 | 4.26E-05 |
| lncRNA_014504 | Traes_2BL_F3FC093D8 | -0.993274 | 6.77E-05 |
| lncRNA_051888 | Traes_2BL_F3FC093D8 | -0.9906 | 0.0001321 |
| lncRNA_058136 | Traes_2BL_F3FC093D8 | 0.993975 | 5.43E-05 |
| lncRNA_014501 | Traes_2BL_F4B5C2D79 | -0.990135 | 0.0001455 |
| lncRNA_047461 | Traes_2BL_F4B5C2D79 | -0.99385 | 5.66E-05 |
| TRAES3BF052700300CFD_g | Traes_2BL_F93E1C539 | 0.9969903 | 1.36E-05 |
| lncRNA_006270 | Traes_2BL_FC0F8A3DC | 0.99348 | 6.36E-05 |
| lncRNA_066190 | Traes_2BS_0132CA56B | 0.9900656 | 0.0001475 |
| lncRNA_032897 | Traes_2BS_03736803D | 0.9902008 | 0.0001436 |
| lncRNA_014501 | Traes_2BS_03CDC4306 | -0.992881 | 7.58E-05 |
| lncRNA_047461 | Traes_2BS_03CDC4306 | -0.991103 | 0.0001184 |
| lncRNA_006361 | Traes_2BS_046E98640 | -0.991142 | 0.0001173 |
| lncRNA_012610 | Traes_2BS_046E98640 | -0.99047 | 0.0001358 |
| lncRNA_020477 | Traes_2BS_064B02A89 | 0.9911586 | 0.0001169 |
| lncRNA_048668 | Traes_2BS_064B02A89 | 0.9950015 | 3.74E-05 |
| lncRNA_039325 | Traes_2BS_0B2869248 | -0.990564 | 0.0001331 |
| TRAES3BF052700300CFD_g | Traes_2BS_100054707 | -0.990742 | 0.0001282 |
| lncRNA_006011 | Traes_2BS_19F05C27A | 0.9939374 | 5.50E-05 |
| lncRNA_015487 | Traes_2BS_24273473D | -0.992757 | 7.85E-05 |
| lncRNA_054317 | Traes_2BS_24273473D | -0.99222 | 9.06E-05 |
| Traes_1BS_B58657408 | Traes_2BS_24B01ABC4 | -0.990348 | 0.0001393 |
| lncRNA_039420 | Traes_2BS_2B483208E | 0.9967332 | 1.60E-05 |
| Traes_1BS_B58657408 | Traes_2BS_301B4422F | -0.997307 | 1.09E-05 |
| lncRNA_039420 | Traes_2BS_3212EB7DF | -0.990251 | 0.0001421 |
| TRAES3BF052700300CFD_g | Traes_2BS_3BE74AB33 | 0.9947048 | 4.20E-05 |
| lncRNA_058136 | Traes_2BS_40E324D13 | 0.9918936 | 9.83E-05 |
| lncRNA_051318 | Traes_2BS_42BB38B51 | 0.9903576 | 0.000139 |
| lncRNA_063127 | Traes_2BS_42BB38B51 | -0.997886 | 6.70E-06 |
| TRAES3BF052700300CFD_g | Traes_2BS_486296DE7 | -0.993576 | 6.18E-05 |
| lncRNA_029088 | Traes_2BS_4F489C2B9 | 0.9931096 | 7.11E-05 |
| lncRNA_047317 | Traes_2BS_51A5E656A | 0.9954557 | 3.09E-05 |
| lncRNA_005569 | Traes_2BS_58E7D5315 | 0.9928272 | 7.70E-05 |
| lncRNA_006361 | Traes_2BS_5AEC1AA29 | 0.9937169 | 5.91E-05 |
| lncRNA_012610 | Traes_2BS_5AEC1AA29 | 0.9911299 | 0.0001177 |
| lncRNA_004117 | Traes_2BS_5CDCD5152 | 0.9921639 | 9.19E-05 |
| lncRNA_013231 | Traes_2BS_5CDCD5152 | 0.9953481 | 3.24E-05 |
| lncRNA_034367 | Traes_2BS_5D1B05312 | 0.9937058 | 5.93E-05 |
| lncRNA_072935 | Traes_2BS_5D8A12252 | 0.992451 | 8.53E-05 |
| lncRNA_013190 | Traes_2BS_6174F3DEB | -0.990027 | 0.0001487 |
| lncRNA_051318 | Traes_2BS_6174F3DEB | -0.99319 | 6.94E-05 |
| lncRNA_072935 | Traes_2BS_64BD37C41 | -0.996319 | 2.03E-05 |
| lncRNA_022554 | Traes_2BS_66D0C26F4 | 0.9977811 | 7.38E-06 |
| lncRNA_006011 | Traes_2BS_6AF2B850E | 0.9959783 | 2.42E-05 |
| lncRNA_033754 | Traes_2BS_6F3465A31 | 0.9911533 | 0.0001171 |
| TRAES3BF053100050CFD_g | Traes_2BS_73911552F | 0.9975963 | 8.66E-06 |
| lncRNA_013190 | Traes_2BS_73911552F | 0.9970245 | 1.33E-05 |
| lncRNA_018078 | Traes_2BS_73911552F | 0.99469 | 4.22E-05 |
| lncRNA_043877 | Traes_2BS_73911552F | 0.9907074 | 0.0001291 |
| lncRNA_008977 | Traes_2BS_7700613D4 | -0.993908 | 5.56E-05 |
| lncRNA_008500 | Traes_2BS_796061F89 | -0.992288 | 8.90E-05 |
| lncRNA_026968 | Traes_2BS_796061F89 | -0.991472 | 0.0001088 |
| lncRNA_078349 | Traes_2BS_796061F89 | -0.991358 | 0.0001117 |
| lncRNA_072440 | Traes_2BS_7C174F31D | 0.9975738 | 8.82E-06 |
| lncRNA_039420 | Traes_2BS_87E3BCB91 | -0.995218 | 3.42E-05 |
| lncRNA_029013 | Traes_2BS_8A1FE01EE | 0.9921014 | 9.33E-05 |
| lncRNA_043877 | Traes_2BS_8A1FE01EE | 0.991261 | 0.0001142 |
| lncRNA_080648 | Traes_2BS_8A1FE01EE | 0.9930732 | 7.18E-05 |
| lncRNA_015322 | Traes_2BS_8CC1ED8A2 | 0.9973121 | 1.08E-05 |
| lncRNA_047461 | Traes_2BS_91FF9EB1C | -0.991208 | 0.0001156 |
| lncRNA_005462 | Traes_2BS_965A35A11 | 0.9941607 | 5.10E-05 |
| lncRNA_000928 | Traes_2BS_990895438 | 0.9918765 | 9.87E-05 |
| lncRNA_013053 | Traes_2BS_990895438 | 0.9917512 | 0.0001018 |
| lncRNA_015322 | Traes_2BS_990895438 | 0.9946833 | 4.23E-05 |
| lncRNA_029088 | Traes_2BS_9C5BE0FE9 | 0.9940705 | 5.26E-05 |
| lncRNA_014504 | Traes_2BS_A5272B1A1 | -0.993255 | 6.81E-05 |
| lncRNA_051318 | Traes_2BS_A5272B1A1 | -0.991681 | 0.0001035 |
| lncRNA_063127 | Traes_2BS_A5272B1A1 | 0.9923236 | 8.82E-05 |
| lncRNA_039420 | Traes_2BS_AABEC0F2F | -0.99674 | 1.59E-05 |
| lncRNA_068995 | Traes_2BS_AECFFDFDD | 0.9937914 | 5.77E-05 |
| lncRNA_048368 | Traes_2BS_B650F673B | 0.9949914 | 3.76E-05 |
| lncRNA_013053 | Traes_2BS_B973866E7 | 0.9915796 | 0.0001061 |
| lncRNA_015322 | Traes_2BS_B973866E7 | 0.99507 | 3.64E-05 |
| lncRNA_019725 | Traes_2BS_B973866E7 | -0.992052 | 9.45E-05 |
| lncRNA_051888 | Traes_2BS_B973866E7 | -0.990826 | 0.0001258 |
| lncRNA_058136 | Traes_2BS_B973866E7 | 0.9934706 | 6.38E-05 |
| lncRNA_054317 | Traes_2BS_BDB1BA66F | 0.9903645 | 0.0001388 |
| lncRNA_063009 | Traes_2BS_BDB1BA66F | 0.9942665 | 4.92E-05 |
| lncRNA_032897 | Traes_2BS_C0C39661E | 0.9956397 | 2.85E-05 |
| lncRNA_006085 | Traes_2BS_C8FB3060D | -0.995129 | 3.55E-05 |
| lncRNA_033754 | Traes_2BS_C8FB3060D | -0.992601 | 8.19E-05 |
| lncRNA_014501 | Traes_2BS_CAA102CFC | 0.9905064 | 0.0001348 |
| lncRNA_000928 | Traes_2BS_CAA1398DB | 0.9927827 | 7.79E-05 |
| lncRNA_009698 | Traes_2BS_CAA1398DB | -0.992801 | 7.76E-05 |
| lncRNA_013190 | Traes_2BS_CAA1398DB | -0.990284 | 0.0001411 |
| lncRNA_019725 | Traes_2BS_CAA1398DB | -0.996598 | 1.73E-05 |
| lncRNA_051318 | Traes_2BS_CAA1398DB | -0.99164 | 0.0001045 |
| lncRNA_009362 | Traes_2BS_CAEE18AAC | -0.993212 | 6.90E-05 |
| lncRNA_054623 | Traes_2BS_CFACD0134 | 0.9944468 | 4.62E-05 |
| lncRNA_066190 | Traes_2BS_CFACD0134 | 0.9964301 | 1.91E-05 |
| lncRNA_051551 | Traes_2BS_D02FBEFB5 | 0.9951468 | 3.53E-05 |
| TRAES3BF026700070CFD_g | Traes_2BS_D26800667 | 0.9984815 | 3.46E-06 |
| lncRNA_049669 | Traes_2BS_D26800667 | 0.9907304 | 0.0001285 |
| lncRNA_008500 | Traes_2BS_D762617B7 | -0.991392 | 0.0001108 |
| lncRNA_014501 | Traes_2BS_E0B8D21CD | -0.994421 | 4.66E-05 |
| lncRNA_061738 | Traes_2BS_E0B8D21CD | -0.990317 | 0.0001402 |
| lncRNA_068995 | Traes_2BS_ECC74F149 | 0.9915243 | 0.0001075 |
| lncRNA_008977 | Traes_2BS_F19F39996 | -0.990593 | 0.0001323 |
| lncRNA_018111 | Traes_2BS_F19F39996 | -0.990026 | 0.0001487 |
| lncRNA_024152 | Traes_2BS_F19F39996 | -0.990491 | 0.0001352 |
| lncRNA_047461 | Traes_2BS_F19F39996 | -0.995142 | 3.53E-05 |
| lncRNA_063009 | Traes_2BS_F19F39996 | -0.990809 | 0.0001263 |
| lncRNA_014538 | Traes_2BS_F73EFC0DE | 0.9950998 | 3.60E-05 |
| lncRNA_074434 | Traes_2BS_F73EFC0DE | 0.9955317 | 2.99E-05 |
| lncRNA_020477 | Traes_2DL_000136878 | 0.9969372 | 1.41E-05 |
| lncRNA_048668 | Traes_2DL_000136878 | 0.9919876 | 9.60E-05 |
| lncRNA_029384 | Traes_2DL_02A40DAE4 | 0.993122 | 7.08E-05 |
| lncRNA_008977 | Traes_2DL_04535D371 | 0.9941489 | 5.13E-05 |
| TRAES3BF052700300CFD_g | Traes_2DL_06741A74F | 0.9920194 | 9.53E-05 |
| lncRNA_008977 | Traes_2DL_06741A74F | 0.9951746 | 3.49E-05 |
| lncRNA_047461 | Traes_2DL_06741A74F | 0.994009 | 5.37E-05 |
| TRAES3BF052700300CFD_g | Traes_2DL_07F08C844 | 0.9924504 | 8.53E-05 |
| lncRNA_064639 | Traes_2DL_0B13E5B2D | -0.997071 | 1.29E-05 |
| lncRNA_029013 | Traes_2DL_0EA361FF0 | -0.990467 | 0.0001359 |
| lncRNA_001557 | Traes_2DL_0F9239600 | -0.990751 | 0.0001279 |
| lncRNA_001839 | Traes_2DL_0F9239600 | 0.9921173 | 9.30E-05 |
| lncRNA_009698 | Traes_2DL_10C74B50E | 0.9927141 | 7.94E-05 |
| lncRNA_039289 | Traes_2DL_11F6A8A27 | -0.991043 | 0.00012 |
| lncRNA_026943 | Traes_2DL_13533D388 | 0.9949368 | 3.84E-05 |
| lncRNA_039943 | Traes_2DL_18D53A586 | 0.9919774 | 9.63E-05 |
| lncRNA_053766 | Traes_2DL_18D53A586 | 0.9961639 | 2.20E-05 |
| lncRNA_068995 | Traes_2DL_1C46C0BB9 | 0.9918419 | 9.96E-05 |
| TRAES3BF052700300CFD_g | Traes_2DL_28DFAC79D | 0.9930876 | 7.15E-05 |
| lncRNA_061738 | Traes_2DL_28DFAC79D | 0.994045 | 5.31E-05 |
| lncRNA_059323 | Traes_2DL_28FF6250C | -0.993381 | 6.56E-05 |
| lncRNA_021433 | Traes_2DL_33ECF2D6A | 0.9938503 | 5.66E-05 |
| lncRNA_025391 | Traes_2DL_3DEEA1F72 | -0.996101 | 2.28E-05 |
| lncRNA_009362 | Traes_2DL_41666605D | -0.990343 | 0.0001394 |
| lncRNA_048668 | Traes_2DL_49AA3F1D1 | 0.9978307 | 7.05E-06 |
| lncRNA_051888 | Traes_2DL_49AA3F1D1 | -0.993706 | 5.93E-05 |
| lncRNA_053766 | Traes_2DL_49AA3F1D1 | 0.9948176 | 4.02E-05 |
| lncRNA_039943 | Traes_2DL_53CC6EB25 | 0.9902455 | 0.0001423 |
| lncRNA_015487 | Traes_2DL_5436C046D | 0.991676 | 0.0001036 |
| lncRNA_018111 | Traes_2DL_5436C046D | 0.9901519 | 0.000145 |
| lncRNA_048256 | Traes_2DL_5436C046D | 0.991558 | 0.0001066 |
| lncRNA_063009 | Traes_2DL_5436C046D | 0.9948373 | 3.99E-05 |
| lncRNA_039420 | Traes_2DL_59DE7446E | -0.995562 | 2.95E-05 |
| lncRNA_026968 | Traes_2DL_5DAAA0DF7 | 0.9903315 | 0.0001398 |
| lncRNA_072935 | Traes_2DL_6047E6000 | -0.990958 | 0.0001223 |
| lncRNA_046989 | Traes_2DL_6196F8454 | -0.993024 | 7.28E-05 |
| lncRNA_024812 | Traes_2DL_62B1A4D13 | 0.9905758 | 0.0001328 |
| TRAES3BF053100050CFD_g | Traes_2DL_6CD064E13 | 0.9914312 | 0.0001098 |
| lncRNA_013190 | Traes_2DL_6CD064E13 | 0.9985016 | 3.37E-06 |
| lncRNA_018078 | Traes_2DL_6CD064E13 | 0.996439 | 1.90E-05 |
| lncRNA_048256 | Traes_2DL_6CD064E13 | 0.9922901 | 8.89E-05 |
| lncRNA_051318 | Traes_2DL_6CD064E13 | 0.9969591 | 1.39E-05 |
| lncRNA_006859 | Traes_2DL_6E8DD862E | 0.9927688 | 7.82E-05 |
| lncRNA_074837 | Traes_2DL_6EAD0DD1C | 0.9959091 | 2.51E-05 |
| lncRNA_000928 | Traes_2DL_7341B95A4 | 0.997964 | 6.21E-06 |
| lncRNA_039943 | Traes_2DL_7341B95A4 | 0.9943323 | 4.81E-05 |
| lncRNA_058136 | Traes_2DL_7341B95A4 | 0.9908335 | 0.0001257 |
| lncRNA_001557 | Traes_2DL_7799F1F75 | 0.9929242 | 7.49E-05 |
| TRAES3BF053100050CFD_g | Traes_2DL_77FEB7329 | 0.9943647 | 4.75E-05 |
| lncRNA_007700 | Traes_2DL_77FEB7329 | 0.9902092 | 0.0001433 |
| lncRNA_013190 | Traes_2DL_77FEB7329 | 0.9978407 | 6.99E-06 |
| lncRNA_018078 | Traes_2DL_77FEB7329 | 0.9966794 | 1.65E-05 |
| lncRNA_048256 | Traes_2DL_77FEB7329 | 0.9914944 | 0.0001082 |
| lncRNA_051318 | Traes_2DL_77FEB7329 | 0.9931573 | 7.01E-05 |
| lncRNA_018078 | Traes_2DL_7B858F797 | 0.9905726 | 0.0001329 |
| lncRNA_043877 | Traes_2DL_7B858F797 | 0.9943487 | 4.78E-05 |
| lncRNA_012105 | Traes_2DL_7FCE012A2 | 0.991628 | 0.0001048 |
| lncRNA_007700 | Traes_2DL_828315764 | 0.9952768 | 3.34E-05 |
| lncRNA_018078 | Traes_2DL_828315764 | 0.995541 | 2.98E-05 |
| lncRNA_029013 | Traes_2DL_828315764 | 0.9929091 | 7.52E-05 |
| lncRNA_043877 | Traes_2DL_828315764 | 0.9908094 | 0.0001263 |
| lncRNA_063127 | Traes_2DL_828315764 | -0.994291 | 4.88E-05 |
| lncRNA_029500 | Traes_2DL_8377D32A2 | -0.991983 | 9.61E-05 |
| lncRNA_045725 | Traes_2DL_85AFEAF70 | 0.9983503 | 4.08E-06 |
| lncRNA_051428 | Traes_2DL_85AFEAF70 | 0.9983503 | 4.08E-06 |
| lncRNA_057614 | Traes_2DL_85AFEAF70 | 0.9983503 | 4.08E-06 |
| lncRNA_065747 | Traes_2DL_85AFEAF70 | 0.9983503 | 4.08E-06 |
| lncRNA_026968 | Traes_2DL_892F83E0B | -0.995226 | 3.41E-05 |
| lncRNA_063547 | Traes_2DL_892F83E0B | -0.991873 | 9.88E-05 |
| lncRNA_078349 | Traes_2DL_892F83E0B | -0.995678 | 2.80E-05 |
| lncRNA_012610 | Traes_2DL_95760A8D5 | 0.9919147 | 9.78E-05 |
| lncRNA_015487 | Traes_2DL_95760A8D5 | 0.9922733 | 8.93E-05 |
| lncRNA_018111 | Traes_2DL_95760A8D5 | 0.9950125 | 3.73E-05 |
| lncRNA_054317 | Traes_2DL_95760A8D5 | 0.9940044 | 5.38E-05 |
| lncRNA_063009 | Traes_2DL_95760A8D5 | 0.9960995 | 2.28E-05 |
| lncRNA_051551 | Traes_2DL_9A7A2A9E2 | 0.9954154 | 3.15E-05 |
| TRAES3BF053100050CFD_g | Traes_2DL_9D3C220D8 | 0.993537 | 6.25E-05 |
| lncRNA_009698 | Traes_2DL_9D3C220D8 | 0.9937901 | 5.77E-05 |
| lncRNA_013190 | Traes_2DL_9D3C220D8 | 0.9943631 | 4.76E-05 |
| lncRNA_018078 | Traes_2DL_9D3C220D8 | 0.9909028 | 0.0001238 |
| lncRNA_034367 | Traes_2DL_9D3C220D8 | 0.9903917 | 0.000138 |
| lncRNA_043877 | Traes_2DL_9D3C220D8 | 0.9932765 | 6.77E-05 |
| lncRNA_008977 | Traes_2DL_9EE6B788E | 0.9928101 | 7.74E-05 |
| lncRNA_083996 | Traes_2DL_A8E6D38B1 | 0.9927348 | 7.90E-05 |
| lncRNA_039420 | Traes_2DL_AE9C5933A | 0.9927481 | 7.87E-05 |
| lncRNA_017776 | Traes_2DL_B143285AE | -0.992108 | 9.32E-05 |
| lncRNA_014501 | Traes_2DL_B6C06C49C | -0.992322 | 8.82E-05 |
| lncRNA_047461 | Traes_2DL_B6C06C49C | -0.993837 | 5.69E-05 |
| TRAES3BF021800100CFD_g | Traes_2DL_BA1B746DF | 0.9903301 | 0.0001398 |
| lncRNA_045725 | Traes_2DL_BA1B746DF | 0.9999679 | 1.55E-09 |
| lncRNA_051428 | Traes_2DL_BA1B746DF | 0.9999679 | 1.55E-09 |
| lncRNA_057614 | Traes_2DL_BA1B746DF | 0.9999679 | 1.55E-09 |
| lncRNA_065747 | Traes_2DL_BA1B746DF | 0.9999679 | 1.55E-09 |
| lncRNA_039943 | Traes_2DL_BE5A2ECE6 | -0.993736 | 5.87E-05 |
| lncRNA_072935 | Traes_2DL_C5C149794 | 0.9916675 | 0.0001039 |
| lncRNA_022554 | Traes_2DL_D02E6E48B | -0.998074 | 5.56E-06 |
| TRAES3BF053100050CFD_g | Traes_2DL_D17DCB9CB | 0.9943778 | 4.73E-05 |
| lncRNA_013190 | Traes_2DL_D17DCB9CB | 0.9973383 | 1.06E-05 |
| lncRNA_018078 | Traes_2DL_D17DCB9CB | 0.9986993 | 2.54E-06 |
| lncRNA_043877 | Traes_2DL_D17DCB9CB | 0.9913568 | 0.0001117 |
| lncRNA_051318 | Traes_2DL_D17DCB9CB | 0.9971276 | 1.24E-05 |
| lncRNA_063127 | Traes_2DL_D17DCB9CB | -0.992405 | 8.63E-05 |
| lncRNA_076127 | Traes_2DL_D45DB0F78 | 0.9912529 | 0.0001144 |
| TRAES3BF051200090CFD_g | Traes_2DL_D75A1F97C | 0.9928756 | 7.60E-05 |
| lncRNA_048266 | Traes_2DL_D75A1F97C | -0.990544 | 0.0001337 |
| lncRNA_072440 | Traes_2DL_DE3909A32 | 0.9911289 | 0.0001177 |
| lncRNA_026968 | Traes_2DL_E1640BFDC | 0.9959138 | 2.50E-05 |
| lncRNA_078349 | Traes_2DL_E1640BFDC | 0.9957995 | 2.64E-05 |
| lncRNA_037690 | Traes_2DL_E32F2C069 | 0.994101 | 5.21E-05 |
| lncRNA_006859 | Traes_2DL_E72DCBDC9 | 0.9970956 | 1.26E-05 |
| Traes_1BS_B58657408 | Traes_2DL_EC8C5E66B | -0.993882 | 5.60E-05 |
| lncRNA_045949 | Traes_2DL_EFD3CE389 | 0.9969373 | 1.41E-05 |
| TRAES3BF052700300CFD_g | Traes_2DL_F311FFC60 | -0.992834 | 7.68E-05 |
| lncRNA_051318 | Traes_2DL_F3A7C2E7B | -0.993899 | 5.57E-05 |
| TRAES3BF053100050CFD_g | Traes_2DL_F714CB6DF | 0.9936341 | 6.07E-05 |
| lncRNA_013190 | Traes_2DL_F714CB6DF | 0.9915524 | 0.0001067 |
| lncRNA_018078 | Traes_2DL_F714CB6DF | 0.9983999 | 3.84E-06 |
| lncRNA_043877 | Traes_2DL_F714CB6DF | 0.9967911 | 1.54E-05 |
| lncRNA_051318 | Traes_2DL_F714CB6DF | 0.9916619 | 0.000104 |
| lncRNA_063127 | Traes_2DL_F714CB6DF | -0.993579 | 6.17E-05 |
| lncRNA_080648 | Traes_2DL_F714CB6DF | 0.9919168 | 9.77E-05 |
| lncRNA_014504 | Traes_2DL_FA7A15F63 | -0.990984 | 0.0001216 |
| lncRNA_019725 | Traes_2DL_FA7A15F63 | -0.993992 | 5.40E-05 |
| lncRNA_044556 | Traes_2DL_FBABE17F1 | 0.9974433 | 9.80E-06 |
| lncRNA_021433 | Traes_2DS_093D3F605 | 0.9905643 | 0.0001331 |
| lncRNA_022554 | Traes_2DS_178B18A13 | -0.990971 | 0.0001219 |
| lncRNA_047461 | Traes_2DS_1D89E0C7E | -0.991091 | 0.0001187 |
| lncRNA_048256 | Traes_2DS_1D89E0C7E | -0.991678 | 0.0001036 |
| lncRNA_013053 | Traes_2DS_228272C06 | 0.9947865 | 4.07E-05 |
| lncRNA_051888 | Traes_2DS_228272C06 | -0.991212 | 0.0001155 |
| lncRNA_001839 | Traes_2DS_22C1F133C | -0.998924 | 1.74E-06 |
| lncRNA_014504 | Traes_2DS_250969F3A | 0.9904023 | 0.0001377 |
| lncRNA_029013 | Traes_2DS_250969F3A | 0.9948257 | 4.01E-05 |
| lncRNA_009293 | Traes_2DS_285D8EB35 | 0.9968964 | 1.44E-05 |
| lncRNA_047461 | Traes_2DS_326E97FCE | 0.990797 | 0.0001267 |
| lncRNA_001072 | Traes_2DS_361FA5A12 | 1 | 0 |
| lncRNA_024464 | Traes_2DS_361FA5A12 | 1 | 0 |
| lncRNA_054394 | Traes_2DS_361FA5A12 | 1 | 0 |
| lncRNA_061348 | Traes_2DS_361FA5A12 | 1 | 0 |
| lncRNA_065442 | Traes_2DS_361FA5A12 | 0.9975408 | 9.06E-06 |
| lncRNA_006011 | Traes_2DS_3AC11B9D8 | 0.9915757 | 0.0001062 |
| lncRNA_026943 | Traes_2DS_3C61BC6AC | 0.9932969 | 6.72E-05 |
| lncRNA_071008 | Traes_2DS_3C61BC6AC | 0.9916325 | 0.0001047 |
| lncRNA_045725 | Traes_2DS_3D2C53D93 | 0.9915557 | 0.0001067 |
| lncRNA_051428 | Traes_2DS_3D2C53D93 | 0.9915557 | 0.0001067 |
| lncRNA_057614 | Traes_2DS_3D2C53D93 | 0.9915557 | 0.0001067 |
| lncRNA_065747 | Traes_2DS_3D2C53D93 | 0.9915557 | 0.0001067 |
| lncRNA_024812 | Traes_2DS_3E640A897 | 0.993364 | 6.59E-05 |
| lncRNA_014501 | Traes_2DS_4646885A5 | 0.994071 | 5.26E-05 |
| lncRNA_061738 | Traes_2DS_4646885A5 | 0.9949387 | 3.84E-05 |
| lncRNA_046989 | Traes_2DS_46AF4C8FE | 0.9924107 | 8.62E-05 |
| lncRNA_048825 | Traes_2DS_561549A20 | 0.9950526 | 3.67E-05 |
| lncRNA_021433 | Traes_2DS_58A206074 | 0.9926105 | 8.17E-05 |
| TRAES3BF177500010CFD_g | Traes_2DS_5AAE4D28E | 0.9974496 | 9.75E-06 |
| lncRNA_072935 | Traes_2DS_5ADE6AC11 | 0.9956181 | 2.88E-05 |
| lncRNA_021433 | Traes_2DS_5FB770318 | -0.990603 | 0.000132 |
| lncRNA_013053 | Traes_2DS_6973E2FF5 | 0.9901586 | 0.0001448 |
| lncRNA_015322 | Traes_2DS_6973E2FF5 | 0.9904369 | 0.0001367 |
| lncRNA_039420 | Traes_2DS_6E504ACA9 | 0.9908451 | 0.0001253 |
| TRAES3BF053100050CFD_g | Traes_2DS_6E564A7CF | -0.995325 | 3.27E-05 |
| lncRNA_007700 | Traes_2DS_6E564A7CF | -0.990757 | 0.0001277 |
| lncRNA_013190 | Traes_2DS_6E564A7CF | -0.997484 | 9.49E-06 |
| lncRNA_018078 | Traes_2DS_6E564A7CF | -0.999026 | 1.42E-06 |
| lncRNA_043877 | Traes_2DS_6E564A7CF | -0.993836 | 5.69E-05 |
| lncRNA_048256 | Traes_2DS_6E564A7CF | -0.990627 | 0.0001314 |
| lncRNA_051318 | Traes_2DS_6E564A7CF | -0.99384 | 5.68E-05 |
| TRAES3BF052700300CFD_g | Traes_2DS_6F88878F7 | 0.9909009 | 0.0001238 |
| lncRNA_014501 | Traes_2DS_6F88878F7 | 0.9980091 | 5.94E-06 |
| lncRNA_047461 | Traes_2DS_6F88878F7 | 0.9953352 | 3.26E-05 |
| lncRNA_061738 | Traes_2DS_6F88878F7 | 0.9931783 | 6.96E-05 |
| lncRNA_039803 | Traes_2DS_70E0D80B3 | 0.993534 | 6.26E-05 |
| lncRNA_029013 | Traes_2DS_7478A1CD1 | 0.9976034 | 8.61E-06 |
| lncRNA_078349 | Traes_2DS_7478A1CD1 | 0.9929608 | 7.42E-05 |
| TRAES3BF052700300CFD_g | Traes_2DS_7704B7B03 | -0.996596 | 1.74E-05 |
| lncRNA_048419 | Traes_2DS_7CAA07C39 | 0.994691 | 4.22E-05 |
| lncRNA_016209 | Traes_2DS_82AF233B4 | 0.9962262 | 2.13E-05 |
| lncRNA_054824 | Traes_2DS_82AF233B4 | 0.9982057 | 4.83E-06 |
| lncRNA_073681 | Traes_2DS_82AF233B4 | 0.9966532 | 1.68E-05 |
| lncRNA_074165 | Traes_2DS_82AF233B4 | 0.9914725 | 0.0001088 |
| lncRNA_076127 | Traes_2DS_8497BBFF9 | 0.9984614 | 3.55E-06 |
| TRAES3BF052700300CFD_g | Traes_2DS_85E22FAC3 | -0.993857 | 5.65E-05 |
| lncRNA_054824 | Traes_2DS_88075F0F3 | 0.993039 | 7.25E-05 |
| lncRNA_073681 | Traes_2DS_88075F0F3 | 0.9914926 | 0.0001083 |
| lncRNA_009293 | Traes_2DS_92645528E | 0.9910988 | 0.0001185 |
| lncRNA_074837 | Traes_2DS_92645528E | 0.9924798 | 8.46E-05 |
| lncRNA_048256 | Traes_2DS_9520F146E | 0.9970505 | 1.30E-05 |
| lncRNA_013190 | Traes_2DS_A270E9A2B | -0.993097 | 7.13E-05 |
| lncRNA_022554 | Traes_2DS_A270E9A2B | -0.996043 | 2.35E-05 |
| lncRNA_048256 | Traes_2DS_A270E9A2B | -0.990238 | 0.0001425 |
| lncRNA_072167 | Traes_2DS_A886F6C92 | -0.990332 | 0.0001398 |
| TRAES3BF052700300CFD_g | Traes_2DS_AC885F2A7 | -0.994976 | 3.78E-05 |
| lncRNA_018111 | Traes_2DS_AECCE3235 | 0.9904933 | 0.0001351 |
| lncRNA_039420 | Traes_2DS_B13F5A980 | -0.990612 | 0.0001318 |
| lncRNA_074837 | Traes_2DS_B13F5A980 | 0.9971741 | 1.20E-05 |
| lncRNA_020939 | Traes_2DS_B174E583E | 0.9942008 | 5.03E-05 |
| lncRNA_045949 | Traes_2DS_B174E583E | 0.99695 | 1.39E-05 |
| lncRNA_048668 | Traes_2DS_B44C394FB | 0.9975882 | 8.72E-06 |
| lncRNA_051888 | Traes_2DS_B44C394FB | -0.99231 | 8.85E-05 |
| lncRNA_053766 | Traes_2DS_B44C394FB | 0.9912519 | 0.0001145 |
| lncRNA_018111 | Traes_2DS_B77283745 | -0.990267 | 0.0001416 |
| lncRNA_051318 | Traes_2DS_B77283745 | -0.99028 | 0.0001413 |
| lncRNA_044308 | Traes_2DS_B9F32C292 | -0.994255 | 4.94E-05 |
| lncRNA_064639 | Traes_2DS_C4237A91B | 0.9925771 | 8.24E-05 |
| lncRNA_015487 | Traes_2DS_C53EC62E8 | 0.993629 | 6.08E-05 |
| lncRNA_009698 | Traes_2DS_C6B631387 | -0.993264 | 6.79E-05 |
| lncRNA_012610 | Traes_2DS_C6B631387 | -0.996892 | 1.45E-05 |
| lncRNA_013190 | Traes_2DS_C6B631387 | -0.99265 | 8.08E-05 |
| lncRNA_048256 | Traes_2DS_C6B631387 | -0.990201 | 0.0001436 |
| lncRNA_053766 | Traes_2DS_C80293002 | 0.9937917 | 5.77E-05 |
| TRAES3BF052700300CFD_g | Traes_2DS_D3BA8D38F1 | -0.994384 | 4.72E-05 |
| lncRNA_000833 | Traes_2DS_DEF497499 | 0.9918061 | 0.0001004 |
| lncRNA_006859 | Traes_2DS_DEF497499 | 0.9951032 | 3.59E-05 |
| lncRNA_026968 | Traes_2DS_EB5F25337 | 0.9947916 | 4.06E-05 |
| lncRNA_078349 | Traes_2DS_EB5F25337 | 0.9963576 | 1.99E-05 |
| lncRNA_022554 | Traes_2DS_EBDE1B880 | -0.996129 | 2.24E-05 |
| lncRNA_008500 | Traes_2DS_EE17BA910 | -0.99588 | 2.54E-05 |
| lncRNA_049300 | Traes_2DS_F6307AF21 | 0.990889 | 0.0001241 |
| lncRNA_009293 | Traes_2DS_FF6CC8441 | 0.9956317 | 2.86E-05 |
| lncRNA_001839 | Traes_3AL_07A02215A | -0.996 | 2.40E-05 |
| lncRNA_000928 | Traes_3AL_0B471F1F6 | 0.9977689 | 7.46E-06 |
| lncRNA_058136 | Traes_3AL_0B471F1F6 | 0.9906313 | 0.0001312 |
| lncRNA_066190 | Traes_3AL_1AB3746B5 | 0.9918163 | 0.0001002 |
| lncRNA_048256 | Traes_3AL_1B5591BEC | -0.991352 | 0.0001119 |
| lncRNA_039325 | Traes_3AL_24EF0F7FE | -0.997275 | 1.11E-05 |
| lncRNA_059976 | Traes_3AL_32C0599AE | -0.99092 | 0.0001233 |
| TRAES3BF060500060CFD_g | Traes_3AL_3395D52D9 | 0.9900063 | 0.0001493 |
| lncRNA_074837 | Traes_3AL_33B515FA4 | 0.9924545 | 8.52E-05 |
| TRAES3BF021800100CFD_g | Traes_3AL_3619D5B88 | 0.9967052 | 1.63E-05 |
| lncRNA_007700 | Traes_3AL_3F641F579 | -0.991566 | 0.0001064 |
| lncRNA_014504 | Traes_3AL_3F641F579 | -0.99365 | 6.04E-05 |
| lncRNA_007700 | Traes_3AL_426C7668F | 0.9929401 | 7.46E-05 |
| lncRNA_063547 | Traes_3AL_426C7668F | 0.9917832 | 0.000101 |
| lncRNA_044556 | Traes_3AL_52A44CE87 | -0.993056 | 7.22E-05 |
| lncRNA_061738 | Traes_3AL_52A44CE87 | -0.990553 | 0.0001334 |
| lncRNA_074658 | Traes_3AL_52A44CE87 | -0.994686 | 4.23E-05 |
| lncRNA_020959 | Traes_3AL_585690EFD | 0.9972705 | 1.12E-05 |
| lncRNA_024807 | Traes_3AL_585690EFD | 0.9958652 | 2.56E-05 |
| lncRNA_044308 | Traes_3AL_585690EFD | 0.9961642 | 2.20E-05 |
| Traes_1BS_B58657408 | Traes_3AL_61E79425F | -0.990112 | 0.0001462 |
| TRAES3BF117100150CFD_g | Traes_3AL_669FE0293 | -0.99355 | 6.23E-05 |
| TRAES3BF117100150CFD_g | Traes_3AL_66B3C6DAF | 0.9911996 | 0.0001158 |
| lncRNA_012242 | Traes_3AL_66B3C6DAF | -0.994855 | 3.96E-05 |
| lncRNA_054317 | Traes_3AL_66B3C6DAF | -0.996809 | 1.53E-05 |
| lncRNA_063009 | Traes_3AL_66B3C6DAF | -0.990781 | 0.0001271 |
| lncRNA_026968 | Traes_3AL_67F605C27 | -0.995388 | 3.19E-05 |
| lncRNA_063547 | Traes_3AL_67F605C27 | -0.995293 | 3.32E-05 |
| lncRNA_078349 | Traes_3AL_67F605C27 | -0.998168 | 5.03E-06 |
| lncRNA_020959 | Traes_3AL_697247353 | 0.9974936 | 9.42E-06 |
| lncRNA_024807 | Traes_3AL_697247353 | 0.9930583 | 7.21E-05 |
| lncRNA_048256 | Traes_3AL_6D1917532 | -0.996883 | 1.46E-05 |
| lncRNA_054623 | Traes_3AL_6FBCF6458 | 0.9903253 | 0.0001399 |
| lncRNA_015487 | Traes_3AL_716569701 | 0.9969032 | 1.44E-05 |
| lncRNA_018111 | Traes_3AL_716569701 | 0.9927626 | 7.84E-05 |
| lncRNA_063009 | Traes_3AL_716569701 | 0.9951412 | 3.54E-05 |
| lncRNA_006011 | Traes_3AL_729C392FB | 0.992495 | 8.43E-05 |
| lncRNA_019725 | Traes_3AL_74A14ACC1 | 0.9906954 | 0.0001295 |
| lncRNA_044308 | Traes_3AL_7658BB10E | 0.9936057 | 6.12E-05 |
| lncRNA_044308 | Traes_3AL_81435CD25 | -0.990274 | 0.0001414 |
| lncRNA_039325 | Traes_3AL_8A6402537 | -0.992586 | 8.23E-05 |
| lncRNA_005569 | Traes_3AL_8BBF67A63 | -0.990613 | 0.0001318 |
| lncRNA_007700 | Traes_3AL_8EAE58688 | 0.9951345 | 3.55E-05 |
| lncRNA_014504 | Traes_3AL_8EAE58688 | 0.9905702 | 0.000133 |
| lncRNA_018078 | Traes_3AL_8EAE58688 | 0.9911859 | 0.0001162 |
| lncRNA_029013 | Traes_3AL_8EAE58688 | 0.9931144 | 7.10E-05 |
| lncRNA_006011 | Traes_3AL_98EAAEB65 | 0.9908126 | 0.0001262 |
| lncRNA_014538 | Traes_3AL_9E1DC3D43 | 0.9931929 | 6.93E-05 |
| lncRNA_016209 | Traes_3AL_A5A6F8761 | 0.9918846 | 9.85E-05 |
| lncRNA_056853 | Traes_3AL_A5A6F8761 | 0.990222 | 0.0001429 |
| lncRNA_073681 | Traes_3AL_A5A6F8761 | 0.9905527 | 0.0001335 |
| lncRNA_074165 | Traes_3AL_A5A6F8761 | 0.9967617 | 1.57E-05 |
| lncRNA_020477 | Traes_3AL_A66270792 | 0.9954177 | 3.14E-05 |
| lncRNA_074658 | Traes_3AL_A89EDC668 | 0.996389 | 1.95E-05 |
| lncRNA_009293 | Traes_3AL_AFA8932CD1 | 0.9919619 | 9.67E-05 |
| lncRNA_020477 | Traes_3AL_AFA8932CD1 | 0.994078 | 5.25E-05 |
| TRAES3BF051200090CFD_g | Traes_3AL_B0D4B56FE | 0.9916528 | 0.0001042 |
| TRAES3BF052700300CFD_g | Traes_3AL_B1E3D8BA1 | 0.993427 | 6.47E-05 |
| lncRNA_026968 | Traes_3AL_B3DB454DC | -0.994557 | 4.44E-05 |
| lncRNA_078349 | Traes_3AL_B3DB454DC | -0.995034 | 3.69E-05 |
| lncRNA_058136 | Traes_3AL_BAB883928 | 0.9900149 | 0.0001491 |
| lncRNA_001839 | Traes_3AL_BD746E324 | -0.996723 | 1.61E-05 |
| TRAES3BF052700300CFD_g | Traes_3AL_BF66C9C95 | -0.992372 | 8.70E-05 |
| lncRNA_001839 | Traes_3AL_BF66C9C95 | -0.990707 | 0.0001291 |
| TRAES3BF053100050CFD_g | Traes_3AL_C0DE37B98 | -0.995776 | 2.67E-05 |
| lncRNA_013190 | Traes_3AL_C0DE37B98 | -0.996729 | 1.60E-05 |
| lncRNA_018078 | Traes_3AL_C0DE37B98 | -0.998904 | 1.80E-06 |
| lncRNA_043877 | Traes_3AL_C0DE37B98 | -0.991576 | 0.0001062 |
| lncRNA_051318 | Traes_3AL_C0DE37B98 | -0.996508 | 1.83E-05 |
| lncRNA_063127 | Traes_3AL_C0DE37B98 | 0.9931889 | 6.94E-05 |
| lncRNA_080648 | Traes_3AL_C0DE37B98 | -0.991529 | 0.0001073 |
| lncRNA_001839 | Traes_3AL_C0F339854 | 0.992632 | 8.12E-05 |
| lncRNA_051551 | Traes_3AL_C178A21CD | 0.9938192 | 5.72E-05 |
| lncRNA_007700 | Traes_3AL_C3A46A4FB | 0.9920734 | 9.40E-05 |
| lncRNA_029013 | Traes_3AL_C3A46A4FB | 0.993889 | 5.59E-05 |
| lncRNA_063547 | Traes_3AL_C3A46A4FB | 0.9932048 | 6.91E-05 |
| lncRNA_017751 | Traes_3AL_CF4245036 | -0.996594 | 1.74E-05 |
| lncRNA_025391 | Traes_3AL_CF4245036 | -0.999007 | 1.48E-06 |
| lncRNA_007700 | Traes_3AL_D2D34DED1 | 0.9932327 | 6.85E-05 |
| lncRNA_018078 | Traes_3AL_D2D34DED1 | 0.9965405 | 1.79E-05 |
| lncRNA_051318 | Traes_3AL_D2D34DED1 | 0.9925842 | 8.23E-05 |
| lncRNA_063127 | Traes_3AL_D2D34DED1 | -0.992856 | 7.64E-05 |
| lncRNA_051551 | Traes_3AL_D426C4603 | 0.9927926 | 7.77E-05 |
| lncRNA_046989 | Traes_3AL_D50C1D6C6 | 0.9927472 | 7.87E-05 |
| lncRNA_009293 | Traes_3AL_E9A97403A | 0.9942287 | 4.99E-05 |
| lncRNA_029384 | Traes_3AL_F3125B233 | 0.994306 | 4.85E-05 |
| lncRNA_009293 | Traes_3AL_FB2DE9BE9 | 0.9945682 | 4.42E-05 |
| TRAES3BF053100050CFD_g | Traes_3AL_FCAB1100B | 0.9917658 | 0.0001014 |
| lncRNA_018078 | Traes_3AL_FCAB1100B | 0.9900603 | 0.0001477 |
| lncRNA_043877 | Traes_3AL_FCAB1100B | 0.9988024 | 2.15E-06 |
| lncRNA_080648 | Traes_3AL_FCAB1100B | 0.9936342 | 6.07E-05 |
| lncRNA_013053 | Traes_3AL_FE33443AF | 0.9944898 | 4.55E-05 |
| lncRNA_068995 | Traes_3AS_074469002 | 0.9900882 | 0.0001469 |
| lncRNA_012242 | Traes_3AS_09D148F37 | 0.9913818 | 0.0001111 |
| lncRNA_014501 | Traes_3AS_0C477EE8D | -0.992186 | 9.13E-05 |
| lncRNA_047461 | Traes_3AS_0C477EE8D | -0.995743 | 2.71E-05 |
| TRAES3BF021800100CFD_g | Traes_3AS_1669C55DA | 0.9968265 | 1.51E-05 |
| lncRNA_008977 | Traes_3AS_1DD0D7B7A | 0.9924862 | 8.45E-05 |
| lncRNA_047461 | Traes_3AS_1DD0D7B7A | 0.9980605 | 5.64E-06 |
| lncRNA_054804 | Traes_3AS_21DDCCFCD | 0.9966804 | 1.65E-05 |
| TRAES3BF052700300CFD_g | Traes_3AS_2CD6A2085 | -0.995713 | 2.75E-05 |
| Traes_1BS_B58657408 | Traes_3AS_369371CF7 | -0.995024 | 3.71E-05 |
| Traes_1BS_B58657408 | Traes_3AS_48DBB1984 | -0.995327 | 3.27E-05 |
| lncRNA_006361 | Traes_3AS_541E182FC | -0.997225 | 1.15E-05 |
| lncRNA_015487 | Traes_3AS_541E182FC | -0.992939 | 7.46E-05 |
| lncRNA_039420 | Traes_3AS_58EAB01FE | -0.999001 | 1.50E-06 |
| lncRNA_047317 | Traes_3AS_5E3A6377C | 0.9934017 | 6.52E-05 |
| lncRNA_009293 | Traes_3AS_5E5EA077A | 0.9953232 | 3.28E-05 |
| lncRNA_048825 | Traes_3AS_5F931AEE4 | 0.9914521 | 0.0001093 |
| lncRNA_009293 | Traes_3AS_617ECD6F6 | 0.9903703 | 0.0001387 |
| lncRNA_010675 | Traes_3AS_639F4A8B2 | 0.994081 | 5.24E-05 |
| lncRNA_069396 | Traes_3AS_68240596D | -0.991541 | 0.000107 |
| lncRNA_013053 | Traes_3AS_8A727B48F | 0.9912799 | 0.0001137 |
| lncRNA_058136 | Traes_3AS_8A727B48F | 0.9941057 | 5.20E-05 |
| lncRNA_014639 | Traes_3AS_965E2F790 | 0.9929307 | 7.48E-05 |
| lncRNA_032048 | Traes_3AS_9892592E5 | 0.998069 | 5.59E-06 |
| lncRNA_082364 | Traes_3AS_A9D946CDB | -0.993142 | 7.04E-05 |
| lncRNA_048668 | Traes_3AS_AAA495D32 | 0.9986624 | 2.68E-06 |
| lncRNA_051888 | Traes_3AS_AAA495D32 | -0.993684 | 5.97E-05 |
| lncRNA_053766 | Traes_3AS_AAA495D32 | 0.9918759 | 9.87E-05 |
| lncRNA_014501 | Traes_3AS_AE28A51DB | 0.9940049 | 5.38E-05 |
| lncRNA_024807 | Traes_3AS_AE28A51DB | 0.9948039 | 4.04E-05 |
| lncRNA_061738 | Traes_3AS_AE28A51DB | 0.9991258 | 1.15E-06 |
| lncRNA_004117 | Traes_3AS_B3B799BFB | 0.9908365 | 0.0001256 |
| lncRNA_020477 | Traes_3AS_B8C00449D | 0.9919459 | 9.70E-05 |
| lncRNA_018036 | Traes_3AS_BD5BDD5EE | 0.9966154 | 1.72E-05 |
| lncRNA_027203 | Traes_3AS_BD5BDD5EE | 0.9977558 | 7.55E-06 |
| lncRNA_048266 | Traes_3AS_C1830DBAE | 0.9952004 | 3.45E-05 |
| lncRNA_030379 | Traes_3AS_C25151458 | 0.9955481 | 2.97E-05 |
| lncRNA_039420 | Traes_3AS_D1E1079AA | -0.991647 | 0.0001044 |
| lncRNA_074837 | Traes_3AS_D6CBEB7BA | 0.9904598 | 0.0001361 |
| lncRNA_083996 | Traes_3AS_E01675F4E | 0.99881 | 2.12E-06 |
| lncRNA_051888 | Traes_3AS_F23A6A83C | -0.991771 | 0.0001013 |
| lncRNA_054623 | Traes_3AS_F441DDE21 | 0.9953573 | 3.23E-05 |
| lncRNA_066190 | Traes_3AS_F441DDE21 | 0.9953538 | 3.23E-05 |
| lncRNA_009698 | Traes_3AS_FD018268B | 0.9912456 | 0.0001146 |
| lncRNA_013190 | Traes_3AS_FD018268B | 0.9948736 | 3.94E-05 |
| lncRNA_018078 | Traes_3AS_FD018268B | 0.9910696 | 0.0001193 |
| lncRNA_019725 | Traes_3AS_FD018268B | 0.9905415 | 0.0001338 |
| lncRNA_048256 | Traes_3AS_FD018268B | 0.9936537 | 6.03E-05 |
| lncRNA_051318 | Traes_3AS_FD018268B | 0.9949981 | 3.75E-05 |
| lncRNA_024152 | Traes_3DL_01383BD6A | -0.990012 | 0.0001491 |
| lncRNA_048825 | Traes_3DL_07B4DE0AB | 0.9956076 | 2.89E-05 |
| lncRNA_066190 | Traes_3DL_07B4DE0AB | 0.9937267 | 5.89E-05 |
| lncRNA_059323 | Traes_3DL_0D2EA3B33 | -0.992194 | 9.12E-05 |
| lncRNA_020477 | Traes_3DL_0E549AF9C | 0.9936337 | 6.07E-05 |
| lncRNA_039420 | Traes_3DL_1174C3707 | -0.993893 | 5.58E-05 |
| lncRNA_044308 | Traes_3DL_1174C3707 | -0.99278 | 7.80E-05 |
| lncRNA_072935 | Traes_3DL_13F4E0414 | -0.991222 | 0.0001152 |
| lncRNA_000928 | Traes_3DL_15B155464 | 0.9943165 | 4.84E-05 |
| lncRNA_019725 | Traes_3DL_15B155464 | -0.990958 | 0.0001223 |
| lncRNA_037690 | Traes_3DL_16D408F20 | -0.99599 | 2.41E-05 |
| lncRNA_080648 | Traes_3DL_1F17A7942 | 0.9927152 | 7.94E-05 |
| lncRNA_022554 | Traes_3DL_2114C4621 | -0.993835 | 5.69E-05 |
| lncRNA_063009 | Traes_3DL_2114C4621 | -0.994014 | 5.36E-05 |
| lncRNA_046989 | Traes_3DL_2FE7E877E | 0.9951823 | 3.48E-05 |
| lncRNA_022554 | Traes_3DL_3856FE114 | -0.992655 | 8.07E-05 |
| lncRNA_029528 | Traes_3DL_4A818FD98 | 0.9942503 | 4.95E-05 |
| lncRNA_084019 | Traes_3DL_4A818FD98 | 0.9900052 | 0.0001493 |
| lncRNA_039420 | Traes_3DL_4C255B900 | -0.992402 | 8.64E-05 |
| lncRNA_014538 | Traes_3DL_500F628BA | 0.9956093 | 2.89E-05 |
| lncRNA_049300 | Traes_3DL_500F628BA | 0.9965379 | 1.80E-05 |
| lncRNA_026968 | Traes_3DL_55B6B2012 | -0.993011 | 7.31E-05 |
| lncRNA_078349 | Traes_3DL_55B6B2012 | -0.991074 | 0.0001192 |
| lncRNA_074658 | Traes_3DL_667C1450C | 0.9930266 | 7.28E-05 |
| lncRNA_018111 | Traes_3DL_71C489A10 | 0.9916036 | 0.0001055 |
| lncRNA_024152 | Traes_3DL_71C489A10 | 0.9972512 | 1.13E-05 |
| lncRNA_018078 | Traes_3DL_7387D0C5C | 0.9949355 | 3.84E-05 |
| lncRNA_051318 | Traes_3DL_7387D0C5C | 0.9944426 | 4.62E-05 |
| lncRNA_063127 | Traes_3DL_7387D0C5C | -0.992768 | 7.83E-05 |
| lncRNA_074837 | Traes_3DL_87A1921B5 | 0.9929686 | 7.40E-05 |
| lncRNA_029088 | Traes_3DL_8FD0F859B | 0.9937052 | 5.93E-05 |
| lncRNA_012610 | Traes_3DL_92D59112E | -0.992943 | 7.45E-05 |
| lncRNA_018111 | Traes_3DL_92D59112E | -0.99166 | 0.0001041 |
| lncRNA_063009 | Traes_3DL_92D59112E | -0.994362 | 4.76E-05 |
| lncRNA_083996 | Traes_3DL_9640B3E35 | 0.9965363 | 1.80E-05 |
| lncRNA_000928 | Traes_3DL_967EE0DE0 | 0.9944318 | 4.64E-05 |
| lncRNA_019725 | Traes_3DL_967EE0DE0 | -0.990026 | 0.0001487 |
| lncRNA_039943 | Traes_3DL_967EE0DE0 | 0.9928735 | 7.60E-05 |
| lncRNA_051888 | Traes_3DL_967EE0DE0 | -0.990972 | 0.0001219 |
| lncRNA_058136 | Traes_3DL_967EE0DE0 | 0.9969802 | 1.37E-05 |
| lncRNA_006011 | Traes_3DL_A25B225DB | 0.9937462 | 5.85E-05 |
| lncRNA_017776 | Traes_3DL_A51D7C6E3 | -0.991064 | 0.0001194 |
| TRAES3BF052700300CFD_g | Traes_3DL_AB617A475 | -0.99487 | 3.94E-05 |
| lncRNA_084019 | Traes_3DL_B62A5FB0E | 0.9937181 | 5.91E-05 |
| lncRNA_029013 | Traes_3DL_BFACAD173 | 0.9924193 | 8.60E-05 |
| lncRNA_072167 | Traes_3DL_CDBB45EBA | 0.9941475 | 5.13E-05 |
| lncRNA_000928 | Traes_3DL_D174E1D63 | -0.991042 | 0.00012 |
| lncRNA_051318 | Traes_3DL_D174E1D63 | 0.9914038 | 0.0001105 |
| lncRNA_058136 | Traes_3DL_D174E1D63 | -0.997756 | 7.55E-06 |
| lncRNA_006270 | Traes_3DL_D292A338C | -0.993202 | 6.92E-05 |
| lncRNA_000928 | Traes_3DL_DBFBC0273 | -0.990782 | 0.0001271 |
| lncRNA_019725 | Traes_3DL_DBFBC0273 | 0.9919366 | 9.73E-05 |
| lncRNA_051318 | Traes_3DL_DBFBC0273 | 0.9958494 | 2.58E-05 |
| lncRNA_058136 | Traes_3DL_DBFBC0273 | -0.994394 | 4.71E-05 |
| lncRNA_014538 | Traes_3DL_E2C3A375E | 0.9935266 | 6.27E-05 |
| TRAES3BF053100050CFD_g | Traes_3DL_EE0699FDC | 0.9921186 | 9.29E-05 |
| lncRNA_013190 | Traes_3DL_EE0699FDC | 0.9905792 | 0.0001327 |
| lncRNA_014504 | Traes_3DL_EE0699FDC | 0.990709 | 0.0001291 |
| lncRNA_018078 | Traes_3DL_EE0699FDC | 0.9980123 | 5.92E-06 |
| lncRNA_019725 | Traes_3DL_EE0699FDC | 0.9904558 | 0.0001362 |
| lncRNA_043877 | Traes_3DL_EE0699FDC | 0.9954403 | 3.11E-05 |
| lncRNA_051318 | Traes_3DL_EE0699FDC | 0.9926729 | 8.03E-05 |
| lncRNA_063127 | Traes_3DL_EE0699FDC | -0.994911 | 3.88E-05 |
| lncRNA_080648 | Traes_3DL_EE0699FDC | 0.991387 | 0.000111 |
| lncRNA_025391 | Traes_3DL_F31768892 | -0.991904 | 9.81E-05 |
| lncRNA_017776 | Traes_3DL_F4962E8FC | -0.992523 | 8.37E-05 |
| lncRNA_074658 | Traes_3DS_094739A97 | 0.9912421 | 0.0001147 |
| TRAES3BF052700300CFD_g | Traes_3DS_2154A90E2 | -0.990626 | 0.0001314 |
| lncRNA_016209 | Traes_3DS_43D0A7613 | 0.9954548 | 3.09E-05 |
| lncRNA_024433 | Traes_3DS_43D0A7613 | 0.9916981 | 0.0001031 |
| lncRNA_074165 | Traes_3DS_43D0A7613 | 0.9930957 | 7.13E-05 |
| lncRNA_015487 | Traes_3DS_84218E926 | 0.9945203 | 4.50E-05 |
| lncRNA_054317 | Traes_3DS_84218E926 | 0.9971217 | 1.24E-05 |
| lncRNA_063009 | Traes_3DS_84218E926 | 0.9968698 | 1.47E-05 |
| lncRNA_063547 | Traes_3DS_910415CA8 | 0.9901191 | 0.000146 |
| lncRNA_000833 | Traes_3DS_955C15EBC | 0.9935243 | 6.28E-05 |
| lncRNA_007700 | Traes_3DS_9E292BCAF | 0.9929133 | 7.52E-05 |
| lncRNA_018078 | Traes_3DS_9E292BCAF | 0.9900537 | 0.0001479 |
| lncRNA_022554 | Traes_3DS_9E292BCAF | 0.9936964 | 5.95E-05 |
| TRAES3BF117100150CFD_g | Traes_3DS_BA1FCA793 | 0.9931462 | 7.03E-05 |
| lncRNA_012610 | Traes_3DS_BA1FCA793 | -0.990258 | 0.0001419 |
| lncRNA_013190 | Traes_3DS_C0A4FDDDD | -0.997148 | 1.22E-05 |
| lncRNA_018078 | Traes_3DS_C0A4FDDDD | -0.990975 | 0.0001218 |
| lncRNA_022554 | Traes_3DS_C0A4FDDDD | -0.992013 | 9.54E-05 |
| lncRNA_048256 | Traes_3DS_C0A4FDDDD | -0.997177 | 1.19E-05 |
| lncRNA_051318 | Traes_3DS_C0A4FDDDD | -0.991267 | 0.0001141 |
| lncRNA_024152 | Traes_3DS_C917FF785 | 0.9954662 | 3.08E-05 |
| lncRNA_013190 | Traes_3DS_EE5D85DE2 | 0.9902117 | 0.0001432 |
| lncRNA_051318 | Traes_3DS_EE5D85DE2 | 0.9967495 | 1.58E-05 |
| lncRNA_024812 | Traes_4AL_00DB56D41 | 0.992516 | 8.38E-05 |
| lncRNA_068995 | Traes_4AL_03FA973CB | 0.9937475 | 5.85E-05 |
| lncRNA_000928 | Traes_4AL_0C44C35BE | 0.9986014 | 2.93E-06 |
| lncRNA_015322 | Traes_4AL_0C44C35BE | 0.9908189 | 0.0001261 |
| lncRNA_019725 | Traes_4AL_0C44C35BE | -0.996114 | 2.26E-05 |
| lncRNA_058136 | Traes_4AL_0C44C35BE | 0.9917936 | 0.0001007 |
| lncRNA_063127 | Traes_4AL_0DF1F5B781 | 0.994309 | 4.85E-05 |
| lncRNA_080648 | Traes_4AL_0DF1F5B781 | -0.992415 | 8.61E-05 |
| lncRNA_054317 | Traes_4AL_1CD626203 | 0.9947581 | 4.11E-05 |
| lncRNA_024812 | Traes_4AL_1D4A5919A1 | 0.9901824 | 0.0001441 |
| lncRNA_006361 | Traes_4AL_234D9FEC7 | 0.9970774 | 1.28E-05 |
| lncRNA_012610 | Traes_4AL_234D9FEC7 | 0.9946063 | 4.36E-05 |
| lncRNA_015487 | Traes_4AL_234D9FEC7 | 0.998411 | 3.79E-06 |
| lncRNA_018111 | Traes_4AL_234D9FEC7 | 0.9904366 | 0.0001368 |
| lncRNA_068995 | Traes_4AL_23BAF9DBB | 0.9922712 | 8.94E-05 |
| lncRNA_048825 | Traes_4AL_24331D947 | 0.9925323 | 8.34E-05 |
| lncRNA_054623 | Traes_4AL_289715EA4 | 0.993414 | 6.49E-05 |
| lncRNA_066190 | Traes_4AL_289715EA4 | 0.990217 | 0.0001431 |
| lncRNA_049461 | Traes_4AL_350F24589 | -0.99683 | 1.51E-05 |
| lncRNA_069396 | Traes_4AL_41570A84C | -0.997747 | 7.61E-06 |
| TRAES3BF021800100CFD_g | Traes_4AL_4994005CD | 0.9925885 | 8.22E-05 |
| lncRNA_032287 | Traes_4AL_4A9F98ED3 | -0.997468 | 9.61E-06 |
| lncRNA_010675 | Traes_4AL_4B9D56131 | 0.9904933 | 0.0001351 |
| lncRNA_066381 | Traes_4AL_4E9796DCB | 0.9900394 | 0.0001483 |
| TRAES3BF053100050CFD_g | Traes_4AL_50918719B | 0.9912985 | 0.0001132 |
| lncRNA_013190 | Traes_4AL_50918719B | 0.9941271 | 5.16E-05 |
| lncRNA_018078 | Traes_4AL_50918719B | 0.9976054 | 8.59E-06 |
| lncRNA_019725 | Traes_4AL_50918719B | 0.9910461 | 0.0001199 |
| lncRNA_043877 | Traes_4AL_50918719B | 0.9926491 | 8.09E-05 |
| lncRNA_051318 | Traes_4AL_50918719B | 0.9960446 | 2.34E-05 |
| lncRNA_063127 | Traes_4AL_50918719B | -0.993416 | 6.49E-05 |
| lncRNA_018111 | Traes_4AL_53D1C32E4 | -0.993418 | 6.48E-05 |
| lncRNA_057390 | Traes_4AL_56B8D9731 | -0.990267 | 0.0001416 |
| lncRNA_061738 | Traes_4AL_59F5010A5 | 0.9902602 | 0.0001418 |
| lncRNA_039943 | Traes_4AL_5DF356F5A | 0.9938732 | 5.62E-05 |
| lncRNA_006361 | Traes_4AL_5F552AAAD | 0.9924887 | 8.44E-05 |
| lncRNA_015487 | Traes_4AL_5F552AAAD | 0.9930472 | 7.23E-05 |
| lncRNA_048668 | Traes_4AL_61A99532C | -0.99717 | 1.20E-05 |
| TRAES3BF052700300CFD_g | Traes_4AL_6855E4C57 | 0.9905353 | 0.0001339 |
| lncRNA_081387 | Traes_4AL_6B540C95C | -0.995331 | 3.27E-05 |
| lncRNA_039420 | Traes_4AL_71305BD00 | -0.994521 | 4.50E-05 |
| lncRNA_074837 | Traes_4AL_71305BD00 | 0.9944099 | 4.68E-05 |
| lncRNA_049300 | Traes_4AL_7A21DB8FE | 0.9930613 | 7.21E-05 |
| lncRNA_037690 | Traes_4AL_7F88C0A92 | -0.990287 | 0.000141 |
| lncRNA_048825 | Traes_4AL_80E3D678B | 0.9910715 | 0.0001192 |
| lncRNA_044581 | Traes_4AL_82AB2E772 | 0.9923441 | 8.77E-05 |
| lncRNA_048419 | Traes_4AL_82AB2E772 | 0.9937333 | 5.88E-05 |
| lncRNA_006361 | Traes_4AL_8845F411B | -0.991073 | 0.0001192 |
| lncRNA_015487 | Traes_4AL_8845F411B | -0.996043 | 2.35E-05 |
| lncRNA_018111 | Traes_4AL_8845F411B | -0.991088 | 0.0001188 |
| lncRNA_051318 | Traes_4AL_8845F411B | -0.990808 | 0.0001264 |
| TRAES3BF052700300CFD_g | Traes_4AL_88CB7B650 | -0.994585 | 4.39E-05 |
| lncRNA_047461 | Traes_4AL_88CB7B650 | -0.994377 | 4.73E-05 |
| lncRNA_022554 | Traes_4AL_8911073AB | -0.991065 | 0.0001194 |
| lncRNA_015487 | Traes_4AL_92708BAA0 | 0.9919676 | 9.65E-05 |
| lncRNA_022554 | Traes_4AL_92708BAA0 | 0.990602 | 0.0001321 |
| lncRNA_048256 | Traes_4AL_92708BAA0 | 0.9920757 | 9.39E-05 |
| lncRNA_063009 | Traes_4AL_92708BAA0 | 0.9969776 | 1.37E-05 |
| lncRNA_020959 | Traes_4AL_92F24A5B9 | 0.9914713 | 0.0001088 |
| lncRNA_024807 | Traes_4AL_92F24A5B9 | 0.9918743 | 9.88E-05 |
| lncRNA_044556 | Traes_4AL_944EFB50F | 0.9912494 | 0.0001145 |
| TRAES3BF052700300CFD_g | Traes_4AL_948151631 | -0.992037 | 9.49E-05 |
| lncRNA_008977 | Traes_4AL_97D9897B41 | -0.991471 | 0.0001088 |
| TRAES3BF052700300CFD_g | Traes_4AL_9AE77B9B9 | -0.993573 | 6.18E-05 |
| lncRNA_012610 | Traes_4AL_9D0E18A90 | 0.9915862 | 0.0001059 |
| lncRNA_013190 | Traes_4AL_9D0E18A90 | 0.9928975 | 7.55E-05 |
| lncRNA_048256 | Traes_4AL_9D0E18A90 | 0.9982919 | 4.37E-06 |
| lncRNA_063009 | Traes_4AL_9D0E18A90 | 0.9935049 | 6.31E-05 |
| lncRNA_059554 | Traes_4AL_9DAC6ABE4 | 0.9945852 | 4.39E-05 |
| lncRNA_013190 | Traes_4AL_A3091D983 | -0.990663 | 0.0001304 |
| lncRNA_014504 | Traes_4AL_A3091D983 | -0.991766 | 0.0001014 |
| lncRNA_018078 | Traes_4AL_A3091D983 | -0.99423 | 4.98E-05 |
| lncRNA_019725 | Traes_4AL_A3091D983 | -0.992685 | 8.01E-05 |
| lncRNA_051318 | Traes_4AL_A3091D983 | -0.993291 | 6.74E-05 |
| lncRNA_047461 | Traes_4AL_A73AD2DAD | 0.9981126 | 5.34E-06 |
| lncRNA_025391 | Traes_4AL_B528D14EC | -0.992193 | 9.12E-05 |
| lncRNA_000928 | Traes_4AL_BDE58EA71 | 0.9948027 | 4.04E-05 |
| lncRNA_009698 | Traes_4AL_BDE58EA71 | -0.995755 | 2.70E-05 |
| lncRNA_019725 | Traes_4AL_BDE58EA71 | -0.993251 | 6.82E-05 |
| lncRNA_044556 | Traes_4AL_BE80FCB64 | 0.9943521 | 4.78E-05 |
| TRAES3BF052700300CFD_g | Traes_4AL_C2A08A56A | 0.9955253 | 3.00E-05 |
| lncRNA_014501 | Traes_4AL_C2A08A56A | 0.9904015 | 0.0001378 |
| lncRNA_061738 | Traes_4AL_C2A08A56A | 0.9943169 | 4.84E-05 |
| lncRNA_048825 | Traes_4AL_C2E041581 | 0.9903523 | 0.0001392 |
| lncRNA_008500 | Traes_4AL_C4C082F05 | 0.9929589 | 7.42E-05 |
| lncRNA_000928 | Traes_4AL_C56125840 | 0.9913579 | 0.0001117 |
| lncRNA_019725 | Traes_4AL_C56125840 | -0.991236 | 0.0001149 |
| lncRNA_008977 | Traes_4AL_CB455818E | 0.9964122 | 1.93E-05 |
| lncRNA_013190 | Traes_4AL_D25430175 | 0.9929531 | 7.43E-05 |
| lncRNA_018078 | Traes_4AL_D25430175 | 0.9911087 | 0.0001182 |
| lncRNA_048256 | Traes_4AL_D25430175 | 0.9931337 | 7.06E-05 |
| lncRNA_051318 | Traes_4AL_D25430175 | 0.9960769 | 2.31E-05 |
| lncRNA_074434 | Traes_4AL_D9FD217F8 | 0.9916639 | 0.0001039 |
| lncRNA_082364 | Traes_4AL_DA2E8C68F | 0.9929883 | 7.36E-05 |
| lncRNA_020959 | Traes_4AL_DF55853C2 | 0.9991203 | 1.16E-06 |
| lncRNA_024807 | Traes_4AL_DF55853C2 | 0.9997573 | 8.83E-08 |
| lncRNA_061738 | Traes_4AL_DF55853C2 | 0.9948866 | 3.92E-05 |
| lncRNA_026968 | Traes_4AL_E47637735 | 0.9971277 | 1.24E-05 |
| lncRNA_078349 | Traes_4AL_E47637735 | 0.9983102 | 4.28E-06 |
| TRAES3BF052700300CFD_g | Traes_4AL_E8EEC0D6E | -0.993998 | 5.39E-05 |
| lncRNA_014501 | Traes_4AL_E8EEC0D6E | -0.99461 | 4.35E-05 |
| lncRNA_061738 | Traes_4AL_E8EEC0D6E | -0.996565 | 1.77E-05 |
| TRAES3BF051200090CFD_g | Traes_4AL_E96501F35 | 0.9953251 | 3.27E-05 |
| lncRNA_017776 | Traes_4AL_E96501F35 | -0.990209 | 0.0001433 |
| lncRNA_074165 | Traes_4AL_EE0AD342B | 0.9914123 | 0.0001103 |
| lncRNA_048825 | Traes_4AL_FDBF6AA87 | 0.9916695 | 0.0001038 |
| lncRNA_008784 | Traes_4AL_FF377A215 | -0.998289 | 4.39E-06 |
| lncRNA_072748 | Traes_4AS_02B607421 | 0.9940107 | 5.37E-05 |
| lncRNA_014501 | Traes_4AS_0812D941C | -0.993833 | 5.69E-05 |
| lncRNA_061738 | Traes_4AS_0812D941C | -0.992947 | 7.44E-05 |
| lncRNA_074658 | Traes_4AS_0812D941C | -0.993996 | 5.40E-05 |
| lncRNA_048256 | Traes_4AS_0A6EA57C3 | -0.990468 | 0.0001359 |
| Traes_1BS_B58657408 | Traes_4AS_156ABD2F4 | -0.990799 | 0.0001266 |
| lncRNA_006361 | Traes_4AS_1938645BA | -0.992098 | 9.34E-05 |
| lncRNA_012610 | Traes_4AS_1938645BA | -0.996865 | 1.47E-05 |
| lncRNA_015487 | Traes_4AS_1938645BA | -0.995747 | 2.71E-05 |
| lncRNA_018111 | Traes_4AS_1938645BA | -0.997386 | 1.02E-05 |
| lncRNA_051318 | Traes_4AS_1938645BA | -0.990764 | 0.0001276 |
| lncRNA_063009 | Traes_4AS_1938645BA | -0.990793 | 0.0001268 |
| TRAES3BF052700300CFD_g | Traes_4AS_1BEB20E15 | -0.990686 | 0.0001297 |
| lncRNA_014501 | Traes_4AS_1BEB20E15 | -0.991907 | 9.80E-05 |
| lncRNA_047461 | Traes_4AS_1BEB20E15 | -0.998466 | 3.53E-06 |
| lncRNA_044556 | Traes_4AS_1E79E1072 | 0.9903743 | 0.0001385 |
| lncRNA_074658 | Traes_4AS_1E79E1072 | 0.9928881 | 7.57E-05 |
| lncRNA_009698 | Traes_4AS_2CFCD8D62 | 0.9928539 | 7.64E-05 |
| lncRNA_012610 | Traes_4AS_2CFCD8D62 | 0.9947308 | 4.16E-05 |
| lncRNA_013190 | Traes_4AS_2CFCD8D62 | 0.9931153 | 7.09E-05 |
| lncRNA_018111 | Traes_4AS_2CFCD8D62 | 0.9911284 | 0.0001177 |
| lncRNA_048256 | Traes_4AS_2CFCD8D62 | 0.9947307 | 4.16E-05 |
| lncRNA_051318 | Traes_4AS_2CFCD8D62 | 0.9901279 | 0.0001457 |
| lncRNA_059323 | Traes_4AS_37F805FF1 | -0.996443 | 1.90E-05 |
| lncRNA_049461 | Traes_4AS_3FEDC3E8F | -0.996386 | 1.96E-05 |
| lncRNA_018111 | Traes_4AS_46D587139 | 0.9932901 | 6.74E-05 |
| lncRNA_022554 | Traes_4AS_49955C28B | 0.9970197 | 1.33E-05 |
| lncRNA_039420 | Traes_4AS_4B47AEE0D | -0.99351 | 6.30E-05 |
| lncRNA_074837 | Traes_4AS_4B47AEE0D | 0.9961738 | 2.19E-05 |
| lncRNA_006085 | Traes_4AS_4BEF2DFAD | -0.995943 | 2.47E-05 |
| lncRNA_012242 | Traes_4AS_4C25DF6F4 | -0.996953 | 1.39E-05 |
| lncRNA_054317 | Traes_4AS_4C25DF6F4 | -0.994106 | 5.20E-05 |
| lncRNA_063009 | Traes_4AS_4C25DF6F4 | -0.991285 | 0.0001136 |
| lncRNA_020959 | Traes_4AS_4C529A3CF | -0.996162 | 2.21E-05 |
| lncRNA_024807 | Traes_4AS_4C529A3CF | -0.996663 | 1.67E-05 |
| lncRNA_044308 | Traes_4AS_4C529A3CF | -0.994474 | 4.57E-05 |
| lncRNA_061738 | Traes_4AS_4C529A3CF | -0.990048 | 0.0001481 |
| lncRNA_006361 | Traes_4AS_56D09C48C | -0.99922 | 9.13E-07 |
| lncRNA_012610 | Traes_4AS_56D09C48C | -0.990872 | 0.0001246 |
| lncRNA_015487 | Traes_4AS_56D09C48C | -0.994054 | 5.29E-05 |
| lncRNA_018111 | Traes_4AS_56D09C48C | -0.993466 | 6.39E-05 |
| lncRNA_076127 | Traes_4AS_637D98EC4 | 0.9909941 | 0.0001213 |
| lncRNA_009293 | Traes_4AS_6448382BF | 0.9921779 | 9.15E-05 |
| Traes_1BS_B58657408 | Traes_4AS_6CBE6E93E | -0.997626 | 8.44E-06 |
| lncRNA_009698 | Traes_4AS_6D8493110 | 0.991368 | 0.0001114 |
| lncRNA_043877 | Traes_4AS_6D8493110 | 0.9932358 | 6.85E-05 |
| TRAES3BF117100150CFD_g | Traes_4AS_6F3D0F407 | 0.9906152 | 0.0001317 |
| lncRNA_012610 | Traes_4AS_6F3D0F407 | -0.994312 | 4.84E-05 |
| lncRNA_013190 | Traes_4AS_6F3D0F407 | -0.990784 | 0.000127 |
| lncRNA_022554 | Traes_4AS_714D540BA | -0.990816 | 0.0001261 |
| lncRNA_048256 | Traes_4AS_714D540BA | -0.99096 | 0.0001222 |
| lncRNA_014504 | Traes_4AS_72BEF89AC | -0.992944 | 7.45E-05 |
| lncRNA_032429 | Traes_4AS_8407C90D81 | -0.9912 | 0.0001158 |
| lncRNA_001557 | Traes_4AS_8B4BD912B | -0.992398 | 8.65E-05 |
| lncRNA_059323 | Traes_4AS_98267C6F6 | -0.993124 | 7.07E-05 |
| lncRNA_047461 | Traes_4AS_9EEABCE1C | 0.9935115 | 6.30E-05 |
| lncRNA_048668 | Traes_4AS_9EEABCE1C | -0.990323 | 0.00014 |
| lncRNA_009698 | Traes_4AS_A3EAF8C80 | -0.990795 | 0.0001267 |
| lncRNA_012610 | Traes_4AS_A3EAF8C80 | -0.990516 | 0.0001345 |
| lncRNA_013190 | Traes_4AS_A3EAF8C80 | -0.99747 | 9.59E-06 |
| lncRNA_018078 | Traes_4AS_A3EAF8C80 | -0.991097 | 0.0001185 |
| lncRNA_048256 | Traes_4AS_A3EAF8C80 | -0.99827 | 4.49E-06 |
| lncRNA_051318 | Traes_4AS_A3EAF8C80 | -0.993122 | 7.08E-05 |
| lncRNA_048256 | Traes_4AS_A538B5587 | -0.997137 | 1.23E-05 |
| lncRNA_022554 | Traes_4AS_A65DDCD05 | -0.991347 | 0.000112 |
| lncRNA_029384 | Traes_4AS_B95E89A28 | 0.9937755 | 5.80E-05 |
| Traes_1BS_B58657408 | Traes_4AS_CC36B67F4 | -0.998049 | 5.71E-06 |
| TRAES3BF021800100CFD_g | Traes_4AS_D58530D14 | 0.995971 | 2.43E-05 |
| lncRNA_045725 | Traes_4AS_D58530D14 | 0.9973357 | 1.06E-05 |
| lncRNA_051428 | Traes_4AS_D58530D14 | 0.9973357 | 1.06E-05 |
| lncRNA_057614 | Traes_4AS_D58530D14 | 0.9973357 | 1.06E-05 |
| lncRNA_065747 | Traes_4AS_D58530D14 | 0.9973357 | 1.06E-05 |
| lncRNA_029013 | Traes_4AS_DA6AF313C | 0.9918944 | 9.83E-05 |
| TRAES3BF053100050CFD_g | Traes_4AS_DCA9D3963 | 0.9982223 | 4.74E-06 |
| lncRNA_013190 | Traes_4AS_DCA9D3963 | 0.9947436 | 4.14E-05 |
| lncRNA_018078 | Traes_4AS_DCA9D3963 | 0.998434 | 3.68E-06 |
| lncRNA_043877 | Traes_4AS_DCA9D3963 | 0.9960044 | 2.39E-05 |
| lncRNA_051318 | Traes_4AS_DCA9D3963 | 0.9921961 | 9.11E-05 |
| lncRNA_063127 | Traes_4AS_DCA9D3963 | -0.992085 | 9.37E-05 |
| lncRNA_080648 | Traes_4AS_DCA9D3963 | 0.9950151 | 3.72E-05 |
| lncRNA_012610 | Traes_4AS_EC04D9E8A | 0.9951043 | 3.59E-05 |
| lncRNA_013190 | Traes_4AS_EC04D9E8A | 0.9959794 | 2.42E-05 |
| lncRNA_015487 | Traes_4AS_EC04D9E8A | 0.9921467 | 9.23E-05 |
| lncRNA_048256 | Traes_4AS_EC04D9E8A | 0.9952204 | 3.42E-05 |
| lncRNA_063009 | Traes_4AS_EC04D9E8A | 0.9925787 | 8.24E-05 |
| lncRNA_017776 | Traes_4AS_ED1CA3109 | -0.991149 | 0.0001172 |
| lncRNA_009293 | Traes_4AS_F04DD4409 | 0.9952145 | 3.43E-05 |
| lncRNA_020477 | Traes_4AS_F04DD4409 | 0.9909871 | 0.0001215 |
| lncRNA_006859 | Traes_4AS_FD9FAE3D8 | 0.9938234 | 5.71E-05 |
| lncRNA_012608 | Traes_4AS_FD9FAE3D8 | 0.9941358 | 5.15E-05 |
| lncRNA_029088 | Traes_4BL_0ED171DF5 | 0.993008 | 7.32E-05 |
| lncRNA_046989 | Traes_4BL_0F72E76F1 | 0.9922794 | 8.92E-05 |
| lncRNA_048256 | Traes_4BL_0F72E76F1 | -0.994322 | 4.83E-05 |
| lncRNA_022554 | Traes_4BL_15DBF043B | -0.995612 | 2.88E-05 |
| lncRNA_015322 | Traes_4BL_19891B81D1 | 0.9913621 | 0.0001116 |
| lncRNA_020477 | Traes_4BL_19FA6DCAD | -0.993074 | 7.18E-05 |
| lncRNA_048668 | Traes_4BL_19FA6DCAD | -0.996111 | 2.27E-05 |
| lncRNA_001557 | Traes_4BL_1B9CC8519 | -0.993401 | 6.52E-05 |
| lncRNA_049300 | Traes_4BL_265B4B877 | 0.9900086 | 0.0001492 |
| lncRNA_014504 | Traes_4BL_2D0FA827C1 | -0.997629 | 8.43E-06 |
| lncRNA_014639 | Traes_4BL_2D32FE93C | 0.9937642 | 5.82E-05 |
| lncRNA_044556 | Traes_4BL_2EC596132 | -0.990724 | 0.0001287 |
| lncRNA_074658 | Traes_4BL_2EC596132 | -0.996733 | 1.60E-05 |
| lncRNA_014504 | Traes_4BL_35EEC85F3 | -0.99434 | 4.80E-05 |
| lncRNA_014501 | Traes_4BL_396369E0B | 0.9942922 | 4.88E-05 |
| lncRNA_061738 | Traes_4BL_396369E0B | 0.9938141 | 5.73E-05 |
| TRAES3BF052700300CFD_g | Traes_4BL_39E80EB81 | -0.995114 | 3.58E-05 |
| lncRNA_029528 | Traes_4BL_3C2787B06 | -0.990372 | 0.0001386 |
| TRAES3BF117100150CFD_g | Traes_4BL_455C9E392 | 0.9994964 | 3.80E-07 |
| lncRNA_006085 | Traes_4BL_455C9E392 | -0.993321 | 6.68E-05 |
| lncRNA_006270 | Traes_4BL_46DFECB43 | 0.9915475 | 0.0001069 |
| lncRNA_001839 | Traes_4BL_470D88AD3 | -0.991426 | 0.00011 |
| lncRNA_009698 | Traes_4BL_47F579FE4 | 0.9919056 | 9.80E-05 |
| lncRNA_013190 | Traes_4BL_47F579FE4 | 0.9921183 | 9.29E-05 |
| lncRNA_018078 | Traes_4BL_47F579FE4 | 0.9909491 | 0.0001225 |
| lncRNA_019725 | Traes_4BL_47F579FE4 | 0.9962771 | 2.08E-05 |
| lncRNA_051318 | Traes_4BL_47F579FE4 | 0.9932199 | 6.88E-05 |
| lncRNA_047461 | Traes_4BL_4CC0E0AAA | -0.991475 | 0.0001087 |
| lncRNA_048668 | Traes_4BL_4CC0E0AAA | 0.9956056 | 2.89E-05 |
| lncRNA_068995 | Traes_4BL_4D57AE1AF | 0.9938313 | 5.70E-05 |
| TRAES3BF052700300CFD_g | Traes_4BL_554B7CDD0 | -0.999554 | 2.98E-07 |
| lncRNA_006361 | Traes_4BL_5664064B6 | -0.990319 | 0.0001401 |
| lncRNA_009698 | Traes_4BL_5664064B6 | -0.991069 | 0.0001193 |
| lncRNA_012610 | Traes_4BL_5664064B6 | -0.999092 | 1.24E-06 |
| lncRNA_013190 | Traes_4BL_5664064B6 | -0.990761 | 0.0001277 |
| lncRNA_015487 | Traes_4BL_5664064B6 | -0.993661 | 6.01E-05 |
| lncRNA_018111 | Traes_4BL_5664064B6 | -0.994482 | 4.56E-05 |
| lncRNA_063009 | Traes_4BL_5664064B6 | -0.991533 | 0.0001072 |
| lncRNA_008977 | Traes_4BL_5A55280B4 | 0.9943782 | 4.73E-05 |
| lncRNA_048256 | Traes_4BL_5A55280B4 | 0.9902679 | 0.0001416 |
| lncRNA_063009 | Traes_4BL_5A55280B4 | 0.9911775 | 0.0001164 |
| lncRNA_046989 | Traes_4BL_61C703587 | 0.9916233 | 0.000105 |
| lncRNA_053766 | Traes_4BL_6AD875D11 | 0.9914626 | 0.000109 |
| TRAES3BF021800100CFD_g | Traes_4BL_6F50F272E | 0.9903399 | 0.0001395 |
| lncRNA_045725 | Traes_4BL_6F50F272E | 1 | 0 |
| lncRNA_051428 | Traes_4BL_6F50F272E | 1 | 0 |
| lncRNA_057614 | Traes_4BL_6F50F272E | 1 | 0 |
| lncRNA_065747 | Traes_4BL_6F50F272E | 1 | 0 |
| lncRNA_051551 | Traes_4BL_778386B52 | 0.9915713 | 0.0001063 |
| lncRNA_012242 | Traes_4BL_7F0860AB5 | -0.991957 | 9.68E-05 |
| TRAES3BF021800100CFD_g | Traes_4BL_7FC8FF797 | 0.9938226 | 5.71E-05 |
| lncRNA_045725 | Traes_4BL_7FC8FF797 | 0.9941309 | 5.16E-05 |
| lncRNA_051428 | Traes_4BL_7FC8FF797 | 0.9941309 | 5.16E-05 |
| lncRNA_057614 | Traes_4BL_7FC8FF797 | 0.9941309 | 5.16E-05 |
| lncRNA_065747 | Traes_4BL_7FC8FF797 | 0.9941309 | 5.16E-05 |
| lncRNA_039420 | Traes_4BL_8A8D56D7B | -0.994362 | 4.76E-05 |
| lncRNA_074837 | Traes_4BL_8A8D56D7B | 0.9964713 | 1.87E-05 |
| lncRNA_015487 | Traes_4BL_8E6854176 | -0.99032 | 0.0001401 |
| lncRNA_054317 | Traes_4BL_8E6854176 | -0.991812 | 0.0001003 |
| lncRNA_063009 | Traes_4BL_8E6854176 | -0.99423 | 4.98E-05 |
| lncRNA_029013 | Traes_4BL_8E9618E24 | 0.9991028 | 1.21E-06 |
| lncRNA_048825 | Traes_4BL_8FCA8CED0 | 0.9956621 | 2.82E-05 |
| lncRNA_049669 | Traes_4BL_8FCA8CED0 | 0.9938088 | 5.74E-05 |
| lncRNA_083996 | Traes_4BL_92A21FF72 | 0.9965054 | 1.83E-05 |
| lncRNA_046989 | Traes_4BL_95197062C | 0.9909239 | 0.0001232 |
| lncRNA_006361 | Traes_4BL_99A055A57 | -0.99468 | 4.24E-05 |
| lncRNA_015487 | Traes_4BL_9BCD28A4E | -0.994502 | 4.53E-05 |
| lncRNA_051318 | Traes_4BL_9BCD28A4E | -0.992201 | 9.10E-05 |
| lncRNA_039420 | Traes_4BL_9F1BFA87A | 0.9907393 | 0.0001282 |
| lncRNA_039325 | Traes_4BL_9F2A26511 | 0.991039 | 0.0001201 |
| lncRNA_022554 | Traes_4BL_A632599AF | -0.991732 | 0.0001022 |
| TRAES3BF021800100CFD_g | Traes_4BL_B603D10B1 | 0.9903399 | 0.0001395 |
| lncRNA_045725 | Traes_4BL_B603D10B1 | 1 | 0 |
| lncRNA_051428 | Traes_4BL_B603D10B1 | 1 | 0 |
| lncRNA_057614 | Traes_4BL_B603D10B1 | 1 | 0 |
| lncRNA_065747 | Traes_4BL_B603D10B1 | 1 | 0 |
| lncRNA_072440 | Traes_4BL_BCC3FB7E7 | 0.9995053 | 3.67E-07 |
| lncRNA_022554 | Traes_4BL_BF17A941B | -0.995485 | 3.05E-05 |
| lncRNA_059323 | Traes_4BL_C247E3780 | -0.994903 | 3.89E-05 |
| lncRNA_039325 | Traes_4BL_C4A35201C | -0.993446 | 6.43E-05 |
| lncRNA_051551 | Traes_4BL_C4C4076B7 | 0.9924555 | 8.52E-05 |
| TRAES3BF053100050CFD_g | Traes_4BL_C74A9C0D0 | 0.9965636 | 1.77E-05 |
| lncRNA_007700 | Traes_4BL_C74A9C0D0 | 0.9900365 | 0.0001484 |
| lncRNA_013190 | Traes_4BL_C74A9C0D0 | 0.9936695 | 6.00E-05 |
| lncRNA_018078 | Traes_4BL_C74A9C0D0 | 0.9963959 | 1.95E-05 |
| lncRNA_043877 | Traes_4BL_C74A9C0D0 | 0.9978155 | 7.15E-06 |
| lncRNA_080648 | Traes_4BL_C74A9C0D0 | 0.9900145 | 0.0001491 |
| lncRNA_009698 | Traes_4BL_C7B902A3C | 0.9974161 | 1.00E-05 |
| lncRNA_012610 | Traes_4BL_C7B902A3C | 0.9967206 | 1.61E-05 |
| lncRNA_033754 | Traes_4BL_C7B902A3C | 0.990458 | 0.0001361 |
| lncRNA_051318 | Traes_4BL_CE24F83D3 | -0.996549 | 1.78E-05 |
| lncRNA_006011 | Traes_4BL_D059936E3 | 0.9925593 | 8.28E-05 |
| lncRNA_029088 | Traes_4BL_DE8D7904E | 0.9914727 | 0.0001088 |
| TRAES3BF052700300CFD_g | Traes_4BL_E2E2C4E1D | -0.995565 | 2.95E-05 |
| lncRNA_014501 | Traes_4BL_E2E2C4E1D | -0.990311 | 0.0001404 |
| lncRNA_061738 | Traes_4BL_E2E2C4E1D | -0.991998 | 9.58E-05 |
| lncRNA_014501 | Traes_4BL_E43C1BB11 | -0.992617 | 8.16E-05 |
| lncRNA_024807 | Traes_4BL_E43C1BB11 | -0.991409 | 0.0001104 |
| lncRNA_061738 | Traes_4BL_E43C1BB11 | -0.992906 | 7.53E-05 |
| lncRNA_012242 | Traes_4BL_E8D63587E | -0.992691 | 7.99E-05 |
| lncRNA_077025 | Traes_4BL_EB421721D | 0.9957185 | 2.75E-05 |
| lncRNA_006011 | Traes_4BL_ED719E854 | 0.9933243 | 6.67E-05 |
| lncRNA_000928 | Traes_4BL_F42F809FF | 0.9934192 | 6.48E-05 |
| lncRNA_015322 | Traes_4BL_F42F809FF | 0.9914666 | 0.0001089 |
| lncRNA_019725 | Traes_4BL_F42F809FF | -0.998364 | 4.01E-06 |
| lncRNA_044308 | Traes_4BL_F7AFF4D6C | -0.992767 | 7.83E-05 |
| lncRNA_001839 | Traes_4BL_F858A2E97 | -0.990159 | 0.0001448 |
| lncRNA_013053 | Traes_4BL_FBA9B0C69 | 0.9986309 | 2.81E-06 |
| lncRNA_000928 | Traes_4BS_02C5D7625 | 0.9954764 | 3.06E-05 |
| lncRNA_001557 | Traes_4BS_0A234D9E6 | -0.99015 | 0.0001451 |
| lncRNA_047461 | Traes_4BS_1687E4DC6 | 0.9905544 | 0.0001334 |
| lncRNA_019748 | Traes_4BS_1D0369514 | -0.994597 | 4.37E-05 |
| lncRNA_014538 | Traes_4BS_28D2F8459 | 0.9941655 | 5.10E-05 |
| TRAES3BF052700300CFD_g | Traes_4BS_2BDEF5A53 | 0.9960262 | 2.37E-05 |
| lncRNA_074658 | Traes_4BS_2F4B2C30D | 0.9911411 | 0.0001174 |
| TRAES3BF117100150CFD_g | Traes_4BS_3207E3555 | -0.994189 | 5.06E-05 |
| lncRNA_006361 | Traes_4BS_3207E3555 | 0.9904709 | 0.0001358 |
| lncRNA_001839 | Traes_4BS_3BA45A792 | -0.992363 | 8.73E-05 |
| lncRNA_046989 | Traes_4BS_418FCBBD3 | 0.9945882 | 4.39E-05 |
| lncRNA_024152 | Traes_4BS_4AD56C4F8 | 0.9922852 | 8.90E-05 |
| lncRNA_006361 | Traes_4BS_4BC62B7F7 | 0.9968767 | 1.46E-05 |
| lncRNA_012610 | Traes_4BS_4BC62B7F7 | 0.9960383 | 2.35E-05 |
| lncRNA_015487 | Traes_4BS_4BC62B7F7 | 0.9950414 | 3.68E-05 |
| lncRNA_018111 | Traes_4BS_4BC62B7F7 | 0.9951866 | 3.47E-05 |
| lncRNA_051318 | Traes_4BS_4BC62B7F7 | 0.9945358 | 4.47E-05 |
| lncRNA_051318 | Traes_4BS_4EB2C19CF | -0.994596 | 4.37E-05 |
| TRAES3BF052700300CFD_g | Traes_4BS_5222C50FC | -0.992614 | 8.16E-05 |
| lncRNA_039420 | Traes_4BS_590ABA25F | 0.9952205 | 3.42E-05 |
| lncRNA_044308 | Traes_4BS_590ABA25F | 0.9924761 | 8.47E-05 |
| lncRNA_044308 | Traes_4BS_60B8B106E | -0.997789 | 7.33E-06 |
| lncRNA_013190 | Traes_4BS_62FE27332 | 0.991003 | 0.0001211 |
| lncRNA_024812 | Traes_4BS_6B669D720 | -0.996047 | 2.34E-05 |
| lncRNA_068995 | Traes_4BS_7700FC74D | 0.9905967 | 0.0001322 |
| lncRNA_013190 | Traes_4BS_773925576 | -0.993436 | 6.45E-05 |
| lncRNA_018078 | Traes_4BS_773925576 | -0.995241 | 3.39E-05 |
| lncRNA_051318 | Traes_4BS_773925576 | -0.998226 | 4.72E-06 |
| lncRNA_063127 | Traes_4BS_773925576 | 0.9914428 | 0.0001095 |
| lncRNA_047461 | Traes_4BS_85666ADAF | -0.992024 | 9.52E-05 |
| TRAES3BF021800100CFD_g | Traes_4BS_92426F5BD | 0.992295 | 8.88E-05 |
| lncRNA_045725 | Traes_4BS_92426F5BD | 0.9998039 | 5.77E-08 |
| lncRNA_051428 | Traes_4BS_92426F5BD | 0.9998039 | 5.77E-08 |
| lncRNA_057614 | Traes_4BS_92426F5BD | 0.9998039 | 5.77E-08 |
| lncRNA_065747 | Traes_4BS_92426F5BD | 0.9998039 | 5.77E-08 |
| lncRNA_006011 | Traes_4BS_93E76D6AE | 0.990859 | 0.000125 |
| lncRNA_074837 | Traes_4BS_93E76D6AE | 0.9953177 | 3.28E-05 |
| lncRNA_017777 | Traes_4BS_97AF26E24 | 0.9954776 | 3.06E-05 |
| lncRNA_012610 | Traes_4BS_9940A14C1 | -0.992716 | 7.94E-05 |
| lncRNA_013190 | Traes_4BS_9940A14C1 | -0.996599 | 1.73E-05 |
| lncRNA_018078 | Traes_4BS_9940A14C1 | -0.992371 | 8.71E-05 |
| lncRNA_051318 | Traes_4BS_9940A14C1 | -0.998757 | 2.32E-06 |
| lncRNA_012242 | Traes_4BS_A229FBF7E | -0.993396 | 6.53E-05 |
| lncRNA_054317 | Traes_4BS_A229FBF7E | -0.994666 | 4.26E-05 |
| lncRNA_063009 | Traes_4BS_A229FBF7E | -0.997268 | 1.12E-05 |
| lncRNA_025391 | Traes_4BS_A260A207D | -0.99674 | 1.59E-05 |
| lncRNA_008977 | Traes_4BS_A4FF7F3EA | 0.9935806 | 6.17E-05 |
| lncRNA_054317 | Traes_4BS_A4FF7F3EA | 0.991261 | 0.0001142 |
| lncRNA_063009 | Traes_4BS_A4FF7F3EA | 0.9951797 | 3.48E-05 |
| lncRNA_053829 | Traes_4BS_A63200F27 | 0.9918827 | 9.86E-05 |
| lncRNA_021433 | Traes_4BS_AE3ECD813 | 0.9980343 | 5.79E-06 |
| lncRNA_049461 | Traes_4BS_B03DE0CDA | -0.990442 | 0.0001366 |
| lncRNA_029384 | Traes_4BS_B2497F675 | 0.9963637 | 1.98E-05 |
| TRAES3BF052700300CFD_g | Traes_4BS_C1AB2DDA8 | 0.9933243 | 6.67E-05 |
| lncRNA_013053 | Traes_4BS_C203755F7 | 0.9934403 | 6.44E-05 |
| lncRNA_015322 | Traes_4BS_C203755F7 | 0.9947551 | 4.12E-05 |
| lncRNA_083996 | Traes_4BS_C991EEFCA | 0.9938745 | 5.62E-05 |
| lncRNA_000928 | Traes_4BS_D325D8F34 | 0.9957651 | 2.69E-05 |
| lncRNA_009698 | Traes_4BS_D325D8F34 | -0.991013 | 0.0001208 |
| lncRNA_012610 | Traes_4BS_D325D8F34 | -0.992352 | 8.75E-05 |
| lncRNA_019725 | Traes_4BS_D325D8F34 | -0.990024 | 0.0001488 |
| lncRNA_058136 | Traes_4BS_D325D8F34 | 0.990094 | 0.0001467 |
| lncRNA_072440 | Traes_4BS_DD10197D5 | 0.9949342 | 3.84E-05 |
| lncRNA_020959 | Traes_4BS_E2F6D8493 | 0.9928092 | 7.74E-05 |
| lncRNA_024807 | Traes_4BS_E2F6D8493 | 0.993201 | 6.92E-05 |
| lncRNA_044308 | Traes_4BS_E2F6D8493 | 0.995961 | 2.44E-05 |
| lncRNA_007700 | Traes_4BS_E6A1E95AC | 0.991777 | 0.0001012 |
| lncRNA_018078 | Traes_4BS_E6A1E95AC | 0.9925027 | 8.41E-05 |
| lncRNA_043877 | Traes_4BS_E6A1E95AC | 0.9929005 | 7.54E-05 |
| lncRNA_048256 | Traes_4BS_E6A1E95AC | 0.990133 | 0.0001456 |
| lncRNA_048825 | Traes_4BS_F094E8394 | 0.9922657 | 8.95E-05 |
| lncRNA_047461 | Traes_4BS_F2B98DC82 | -0.99501 | 3.73E-05 |
| lncRNA_008977 | Traes_4BS_F395A220A | 0.9951607 | 3.51E-05 |
| lncRNA_054317 | Traes_4BS_F395A220A | 0.9937244 | 5.90E-05 |
| lncRNA_063009 | Traes_4BS_F395A220A | 0.9989175 | 1.76E-06 |
| lncRNA_051318 | Traes_4BS_F7359FA2E | 0.9962067 | 2.16E-05 |
| lncRNA_048668 | Traes_4BS_FE8F8C8BC | 0.9909405 | 0.0001227 |
| lncRNA_051888 | Traes_4BS_FE8F8C8BC | -0.991 | 0.0001211 |
| lncRNA_053766 | Traes_4BS_FE8F8C8BC | 0.9948637 | 3.95E-05 |
| lncRNA_048668 | Traes_4DL_056EBD3F1 | 0.9900905 | 0.0001468 |
| lncRNA_051888 | Traes_4DL_056EBD3F1 | -0.999724 | 1.15E-07 |
| lncRNA_053766 | Traes_4DL_056EBD3F1 | 0.9900235 | 0.0001488 |
| lncRNA_014639 | Traes_4DL_08BACD57F | -0.991804 | 0.0001005 |
| TRAES3BF052700300CFD_g | Traes_4DL_0B1ABB56F | -0.994648 | 4.29E-05 |
| lncRNA_008977 | Traes_4DL_1A9D268CC | -0.991034 | 0.0001202 |
| lncRNA_063009 | Traes_4DL_1A9D268CC | -0.990351 | 0.0001392 |
| TRAES3BF117100150CFD_g | Traes_4DL_1D631D399 | 0.996601 | 1.73E-05 |
| TRAES3BF053100050CFD_g | Traes_4DL_1F426D07D | 0.9907653 | 0.0001275 |
| lncRNA_029013 | Traes_4DL_1F426D07D | 0.9913329 | 0.0001124 |
| lncRNA_063127 | Traes_4DL_1F426D07D | -0.991888 | 9.84E-05 |
| lncRNA_063547 | Traes_4DL_1F426D07D | 0.9931707 | 6.98E-05 |
| lncRNA_080648 | Traes_4DL_1F426D07D | 0.9961036 | 2.27E-05 |
| lncRNA_014504 | Traes_4DL_1F8BA4DF8 | 0.9930735 | 7.18E-05 |
| lncRNA_018078 | Traes_4DL_1F8BA4DF8 | 0.9916294 | 0.0001048 |
| lncRNA_029013 | Traes_4DL_1F8BA4DF8 | 0.9953955 | 3.18E-05 |
| lncRNA_063127 | Traes_4DL_1F8BA4DF8 | -0.997158 | 1.21E-05 |
| lncRNA_007700 | Traes_4DL_230F5D947 | -0.99332 | 6.68E-05 |
| lncRNA_014504 | Traes_4DL_230F5D947 | -0.993474 | 6.37E-05 |
| lncRNA_018078 | Traes_4DL_230F5D947 | -0.992288 | 8.90E-05 |
| lncRNA_029013 | Traes_4DL_230F5D947 | -0.991066 | 0.0001194 |
| lncRNA_043877 | Traes_4DL_230F5D947 | -0.991822 | 0.0001 |
| lncRNA_032287 | Traes_4DL_2527CA8BF | -0.993742 | 5.86E-05 |
| lncRNA_033754 | Traes_4DL_2527CA8BF | -0.995907 | 2.51E-05 |
| lncRNA_008784 | Traes_4DL_270AF9312 | -0.991971 | 9.64E-05 |
| lncRNA_039420 | Traes_4DL_2C636B5DB | -0.993095 | 7.14E-05 |
| lncRNA_057390 | Traes_4DL_2DBB03D7E | 0.9911294 | 0.0001177 |
| lncRNA_021433 | Traes_4DL_300A4A216 | 0.9933839 | 6.55E-05 |
| lncRNA_001839 | Traes_4DL_32D57D606 | -0.992892 | 7.56E-05 |
| lncRNA_001839 | Traes_4DL_357EA96FB1 | 0.9944366 | 4.63E-05 |
| lncRNA_083996 | Traes_4DL_382DEDEB6 | 0.9951692 | 3.49E-05 |
| lncRNA_048256 | Traes_4DL_3A1814A74 | -0.994471 | 4.58E-05 |
| lncRNA_072935 | Traes_4DL_3B4E92F73 | 0.994713 | 4.19E-05 |
| lncRNA_047461 | Traes_4DL_3D374FF4C | -0.996251 | 2.11E-05 |
| lncRNA_009362 | Traes_4DL_3FD11FE1C | -0.995156 | 3.51E-05 |
| Traes_1BS_B58657408 | Traes_4DL_413A99ECF | -0.990402 | 0.0001378 |
| lncRNA_059323 | Traes_4DL_453E3B607 | -0.994638 | 4.30E-05 |
| lncRNA_013053 | Traes_4DL_478BB6FEF | 0.9988599 | 1.95E-06 |
| lncRNA_058136 | Traes_4DL_478BB6FEF | 0.9939566 | 5.47E-05 |
| lncRNA_001557 | Traes_4DL_4A15E5967 | 0.9956845 | 2.79E-05 |
| TRAES3BF021800100CFD_g | Traes_4DL_4C9D6ADB4 | 0.9927415 | 7.88E-05 |
| lncRNA_045725 | Traes_4DL_4C9D6ADB4 | 0.999828 | 4.44E-08 |
| lncRNA_051428 | Traes_4DL_4C9D6ADB4 | 0.999828 | 4.44E-08 |
| lncRNA_057614 | Traes_4DL_4C9D6ADB4 | 0.999828 | 4.44E-08 |
| lncRNA_065747 | Traes_4DL_4C9D6ADB4 | 0.999828 | 4.44E-08 |
| lncRNA_000928 | Traes_4DL_4E3C922FB | -0.995055 | 3.66E-05 |
| lncRNA_058136 | Traes_4DL_4E3C922FB | -0.997804 | 7.23E-06 |
| lncRNA_063127 | Traes_4DL_4E68C58BB | -0.996863 | 1.47E-05 |
| lncRNA_012608 | Traes_4DL_56B3A5D47 | 0.9951735 | 3.49E-05 |
| lncRNA_048368 | Traes_4DL_5760FBC19 | 0.9922611 | 8.96E-05 |
| lncRNA_014501 | Traes_4DL_59311B671 | -0.999655 | 1.79E-07 |
| lncRNA_061738 | Traes_4DL_59311B671 | -0.996715 | 1.62E-05 |
| lncRNA_009293 | Traes_4DL_5BF5D3B8F | 0.9961024 | 2.28E-05 |
| lncRNA_014538 | Traes_4DL_5BFBDD55E | 0.9905466 | 0.0001336 |
| lncRNA_049300 | Traes_4DL_5BFBDD55E | 0.9918475 | 9.94E-05 |
| lncRNA_056853 | Traes_4DL_5F7C9AF64 | -0.995999 | 2.40E-05 |
| TRAES3BF021800100CFD_g | Traes_4DL_6041ECB9A | 0.9937301 | 5.88E-05 |
| lncRNA_045725 | Traes_4DL_6041ECB9A | 0.9985341 | 3.22E-06 |
| lncRNA_051428 | Traes_4DL_6041ECB9A | 0.9985341 | 3.22E-06 |
| lncRNA_057614 | Traes_4DL_6041ECB9A | 0.9985341 | 3.22E-06 |
| lncRNA_065747 | Traes_4DL_6041ECB9A | 0.9985341 | 3.22E-06 |
| lncRNA_053766 | Traes_4DL_64E445D4A | 0.9972805 | 1.11E-05 |
| lncRNA_001557 | Traes_4DL_65CDCF95A | -0.992412 | 8.62E-05 |
| lncRNA_012610 | Traes_4DL_6834985AD | 0.99181 | 0.0001003 |
| lncRNA_018111 | Traes_4DL_6834985AD | 0.9917556 | 0.0001017 |
| lncRNA_009127 | Traes_4DL_68EAC9343 | 0.9969626 | 1.38E-05 |
| TRAES3BF053100050CFD_g | Traes_4DL_6B139C9A6 | 0.9982841 | 4.41E-06 |
| lncRNA_013190 | Traes_4DL_6B139C9A6 | 0.9958219 | 2.61E-05 |
| lncRNA_018078 | Traes_4DL_6B139C9A6 | 0.9981586 | 5.08E-06 |
| lncRNA_043877 | Traes_4DL_6B139C9A6 | 0.9962728 | 2.08E-05 |
| lncRNA_051318 | Traes_4DL_6B139C9A6 | 0.9908206 | 0.000126 |
| lncRNA_080648 | Traes_4DL_6B139C9A6 | 0.9931803 | 6.96E-05 |
| lncRNA_032287 | Traes_4DL_6CCF8E7B0 | -0.994038 | 5.32E-05 |
| lncRNA_064639 | Traes_4DL_6CCF8E7B0 | -0.993202 | 6.92E-05 |
| lncRNA_000833 | Traes_4DL_71DE87AB7 | 0.9951662 | 3.50E-05 |
| lncRNA_066381 | Traes_4DL_71DE87AB7 | 0.9944307 | 4.64E-05 |
| lncRNA_009127 | Traes_4DL_788BB68B8 | 0.9912903 | 0.0001135 |
| lncRNA_000823 | Traes_4DL_83EDE466C | -0.993831 | 5.70E-05 |
| lncRNA_009362 | Traes_4DL_866EB8D27 | -0.992289 | 8.90E-05 |
| lncRNA_014501 | Traes_4DL_8883F7012 | -0.991677 | 0.0001036 |
| lncRNA_008977 | Traes_4DL_88E010FD6 | -0.993857 | 5.65E-05 |
| lncRNA_047461 | Traes_4DL_88E010FD6 | -0.992387 | 8.67E-05 |
| lncRNA_022554 | Traes_4DL_8D4180F54 | 0.9919081 | 9.80E-05 |
| lncRNA_020959 | Traes_4DL_8E3C8AE92 | 0.9938719 | 5.62E-05 |
| lncRNA_029013 | Traes_4DL_8E3C8AE92 | 0.9924891 | 8.44E-05 |
| lncRNA_012242 | Traes_4DL_8E805248E | 0.9905679 | 0.000133 |
| lncRNA_054317 | Traes_4DL_8E805248E | 0.9913504 | 0.0001119 |
| lncRNA_029384 | Traes_4DL_90DB24B4F | 0.9941022 | 5.21E-05 |
| lncRNA_014501 | Traes_4DL_92D72827C | 0.9914081 | 0.0001104 |
| lncRNA_020959 | Traes_4DL_92D72827C | 0.9907489 | 0.000128 |
| lncRNA_024807 | Traes_4DL_92D72827C | 0.9952978 | 3.31E-05 |
| lncRNA_061738 | Traes_4DL_92D72827C | 0.997506 | 9.32E-06 |
| lncRNA_081387 | Traes_4DL_93D4DED90 | -0.995649 | 2.84E-05 |
| lncRNA_008977 | Traes_4DL_9C912D3EE | -0.991334 | 0.0001123 |
| lncRNA_081387 | Traes_4DL_9CB89AFD3 | 0.9926486 | 8.09E-05 |
| TRAES3BF021800100CFD_g | Traes_4DL_9F1D382C6 | 0.9962828 | 2.07E-05 |
| lncRNA_045725 | Traes_4DL_9F1D382C6 | 0.9986032 | 2.93E-06 |
| lncRNA_051428 | Traes_4DL_9F1D382C6 | 0.9986032 | 2.93E-06 |
| lncRNA_057614 | Traes_4DL_9F1D382C6 | 0.9986032 | 2.93E-06 |
| lncRNA_065747 | Traes_4DL_9F1D382C6 | 0.9986032 | 2.93E-06 |
| lncRNA_015322 | Traes_4DL_A379ABAE9 | 0.9982894 | 4.39E-06 |
| lncRNA_019725 | Traes_4DL_A379ABAE9 | -0.990868 | 0.0001247 |
| lncRNA_033754 | Traes_4DL_A780BF320 | -0.994061 | 5.28E-05 |
| lncRNA_064639 | Traes_4DL_A780BF320 | -0.990424 | 0.0001371 |
| lncRNA_019725 | Traes_4DL_A7C1B0A43 | 0.9912678 | 0.000114 |
| lncRNA_048668 | Traes_4DL_A7C1B0A43 | -0.991563 | 0.0001065 |
| lncRNA_019725 | Traes_4DL_A7FFC65F0 | -0.992934 | 7.47E-05 |
| lncRNA_048668 | Traes_4DL_A7FFC65F0 | 0.9926365 | 8.11E-05 |
| lncRNA_051888 | Traes_4DL_A7FFC65F0 | -0.993107 | 7.11E-05 |
| lncRNA_048825 | Traes_4DL_A943EB2A5 | 0.9903791 | 0.0001384 |
| lncRNA_054623 | Traes_4DL_AF0869DDB | 0.9913723 | 0.0001113 |
| lncRNA_066190 | Traes_4DL_AF0869DDB | 0.9925224 | 8.37E-05 |
| lncRNA_024812 | Traes_4DL_B6353C71C | 0.997494 | 9.41E-06 |
| lncRNA_056853 | Traes_4DL_BAFFF6E71 | 0.9908885 | 0.0001242 |
| lncRNA_015487 | Traes_4DL_BC04763DE | 0.9963369 | 2.01E-05 |
| lncRNA_063009 | Traes_4DL_BC04763DE | 0.9927812 | 7.80E-05 |
| lncRNA_015322 | Traes_4DL_C02A2A149 | 0.990465 | 0.0001359 |
| lncRNA_039319 | Traes_4DL_C63934F2C | 0.9915039 | 0.000108 |
| lncRNA_015487 | Traes_4DL_CBD89C78D | -0.9952 | 3.45E-05 |
| lncRNA_063009 | Traes_4DL_CBD89C78D | -0.992999 | 7.34E-05 |
| lncRNA_029088 | Traes_4DL_CC0D99B5F | 0.9941363 | 5.15E-05 |
| lncRNA_029384 | Traes_4DL_CE24009DC | 0.9928938 | 7.56E-05 |
| lncRNA_008977 | Traes_4DL_CE5AAA3C0 | -0.990809 | 0.0001263 |
| lncRNA_021433 | Traes_4DL_D7F5EDD0E | 0.9903496 | 0.0001392 |
| lncRNA_018111 | Traes_4DL_D95D2246C | -0.991518 | 0.0001076 |
| lncRNA_051318 | Traes_4DL_D95D2246C | -0.995084 | 3.62E-05 |
| lncRNA_058136 | Traes_4DL_D95D2246C | 0.9904523 | 0.0001363 |
| TRAES3BF021800100CFD_g | Traes_4DL_E6B97D6D1 | 0.9903399 | 0.0001395 |
| lncRNA_045725 | Traes_4DL_E6B97D6D1 | 1 | 0 |
| lncRNA_051428 | Traes_4DL_E6B97D6D1 | 1 | 0 |
| lncRNA_057614 | Traes_4DL_E6B97D6D1 | 1 | 0 |
| lncRNA_065747 | Traes_4DL_E6B97D6D1 | 1 | 0 |
| lncRNA_017751 | Traes_4DL_EAF90696F | -0.991401 | 0.0001106 |
| lncRNA_025391 | Traes_4DL_EAF90696F | -0.996501 | 1.83E-05 |
| lncRNA_046989 | Traes_4DL_EC4D8752B | 0.9942414 | 4.96E-05 |
| lncRNA_008500 | Traes_4DL_EDE04BD04 | -0.996557 | 1.78E-05 |
| lncRNA_047317 | Traes_4DL_F1D0EAB201 | 0.9930843 | 7.16E-05 |
| lncRNA_059554 | Traes_4DL_F7B5BD883 | -0.991505 | 0.000108 |
| lncRNA_006361 | Traes_4DS_03983ABEE | -0.992082 | 9.38E-05 |
| lncRNA_008977 | Traes_4DS_0B056789B | 0.9922158 | 9.07E-05 |
| lncRNA_047461 | Traes_4DS_0B056789B | 0.9925164 | 8.38E-05 |
| lncRNA_048256 | Traes_4DS_0B056789B | 0.9934203 | 6.48E-05 |
| lncRNA_063009 | Traes_4DS_0B056789B | 0.9921642 | 9.19E-05 |
| lncRNA_020959 | Traes_4DS_138E9F824 | -0.993881 | 5.61E-05 |
| lncRNA_024807 | Traes_4DS_138E9F824 | -0.995247 | 3.38E-05 |
| lncRNA_044308 | Traes_4DS_138E9F824 | -0.995675 | 2.80E-05 |
| lncRNA_061738 | Traes_4DS_138E9F824 | -0.991469 | 0.0001089 |
| lncRNA_072440 | Traes_4DS_214C38862 | 0.9940348 | 5.33E-05 |
| lncRNA_025391 | Traes_4DS_220628F6B | -0.995803 | 2.64E-05 |
| lncRNA_027894 | Traes_4DS_26600563E | 0.9970229 | 1.33E-05 |
| TRAES3BF053100050CFD_g | Traes_4DS_39F851D36 | 0.9961062 | 2.27E-05 |
| lncRNA_018078 | Traes_4DS_39F851D36 | 0.9971512 | 1.22E-05 |
| lncRNA_043877 | Traes_4DS_39F851D36 | 0.9971812 | 1.19E-05 |
| lncRNA_063127 | Traes_4DS_39F851D36 | -0.99546 | 3.09E-05 |
| lncRNA_080648 | Traes_4DS_39F851D36 | 0.9976409 | 8.34E-06 |
| lncRNA_044308 | Traes_4DS_4C1265002 | 0.9913363 | 0.0001123 |
| lncRNA_047461 | Traes_4DS_4C6846850 | -0.990928 | 0.0001231 |
| lncRNA_048668 | Traes_4DS_4D761086B | -0.994583 | 4.39E-05 |
| lncRNA_051888 | Traes_4DS_4D761086B | 0.9916941 | 0.0001032 |
| lncRNA_053766 | Traes_4DS_4D761086B | -0.991657 | 0.0001041 |
| lncRNA_049669 | Traes_4DS_539982125 | 0.9924977 | 8.42E-05 |
| lncRNA_081387 | Traes_4DS_56FBA9FD6 | -0.990188 | 0.0001439 |
| lncRNA_001557 | Traes_4DS_5A7ABE019 | 0.992618 | 8.15E-05 |
| lncRNA_063547 | Traes_4DS_5AAF38D73 | 0.9916124 | 0.0001052 |
| lncRNA_054317 | Traes_4DS_5FF031B15 | -0.992737 | 7.89E-05 |
| lncRNA_009698 | Traes_4DS_64893FC94 | 0.9932217 | 6.88E-05 |
| lncRNA_012610 | Traes_4DS_64893FC94 | 0.9950791 | 3.63E-05 |
| lncRNA_013190 | Traes_4DS_64893FC94 | 0.9978363 | 7.02E-06 |
| lncRNA_018078 | Traes_4DS_64893FC94 | 0.9911449 | 0.0001173 |
| lncRNA_048256 | Traes_4DS_64893FC94 | 0.9905755 | 0.0001328 |
| lncRNA_051318 | Traes_4DS_64893FC94 | 0.9970194 | 1.33E-05 |
| lncRNA_006361 | Traes_4DS_64893FC941 | 0.9919364 | 9.73E-05 |
| lncRNA_009698 | Traes_4DS_64893FC941 | 0.9900014 | 0.0001495 |
| lncRNA_012610 | Traes_4DS_64893FC941 | 0.9983877 | 3.90E-06 |
| lncRNA_013190 | Traes_4DS_64893FC941 | 0.994974 | 3.78E-05 |
| lncRNA_015487 | Traes_4DS_64893FC941 | 0.995095 | 3.60E-05 |
| lncRNA_018111 | Traes_4DS_64893FC941 | 0.9927169 | 7.94E-05 |
| lncRNA_048256 | Traes_4DS_64893FC941 | 0.9912386 | 0.0001148 |
| lncRNA_051318 | Traes_4DS_64893FC941 | 0.9915574 | 0.0001066 |
| lncRNA_063009 | Traes_4DS_64893FC941 | 0.9902136 | 0.0001432 |
| TRAES3BF052700300CFD_g | Traes_4DS_6A7D1C662 | 0.9912395 | 0.0001148 |
| lncRNA_014501 | Traes_4DS_6A7D1C662 | 0.9930049 | 7.32E-05 |
| lncRNA_047461 | Traes_4DS_6A7D1C662 | 0.9947879 | 4.07E-05 |
| lncRNA_037690 | Traes_4DS_76E977C68 | 0.9976967 | 7.95E-06 |
| TRAES3BF024700270CFD_g | Traes_4DS_77A2652E5 | 0.9927718 | 7.82E-05 |
| lncRNA_014538 | Traes_4DS_77A2652E5 | 0.9900694 | 0.0001474 |
| lncRNA_049300 | Traes_4DS_77A2652E5 | 0.9929426 | 7.45E-05 |
| lncRNA_000928 | Traes_4DS_7F3D9E4D9 | 0.9941536 | 5.12E-05 |
| lncRNA_058136 | Traes_4DS_7F3D9E4D9 | 0.9919719 | 9.64E-05 |
| lncRNA_045725 | Traes_4DS_897D0A9DB | 0.9910155 | 0.0001207 |
| lncRNA_051428 | Traes_4DS_897D0A9DB | 0.9910155 | 0.0001207 |
| lncRNA_057614 | Traes_4DS_897D0A9DB | 0.9910155 | 0.0001207 |
| lncRNA_065747 | Traes_4DS_897D0A9DB | 0.9910155 | 0.0001207 |
| lncRNA_015322 | Traes_4DS_8D5B431C7 | 0.9962674 | 2.09E-05 |
| lncRNA_048256 | Traes_4DS_94005294B | 0.9939197 | 5.53E-05 |
| lncRNA_024807 | Traes_4DS_993693839 | 0.9902536 | 0.000142 |
| lncRNA_059323 | Traes_4DS_A5BF2203B | -0.991941 | 9.72E-05 |
| lncRNA_081387 | Traes_4DS_A5BF2203B | -0.990785 | 0.000127 |
| lncRNA_039943 | Traes_4DS_BD48EEAD8 | 0.9953662 | 3.22E-05 |
| lncRNA_009698 | Traes_4DS_CC45FE96E | 0.993867 | 5.63E-05 |
| lncRNA_012610 | Traes_4DS_CC45FE96E | 0.998715 | 2.48E-06 |
| lncRNA_018111 | Traes_4DS_CC45FE96E | 0.9931788 | 6.96E-05 |
| lncRNA_033754 | Traes_4DS_CC45FE96E | 0.9911809 | 0.0001163 |
| lncRNA_072935 | Traes_4DS_E96A8F05D | 0.9913716 | 0.0001114 |
| lncRNA_048256 | Traes_4DS_EC9D5061D | 0.9944819 | 4.56E-05 |
| lncRNA_001839 | Traes_4DS_ED5F49735 | 0.9980895 | 5.47E-06 |
| lncRNA_012608 | Traes_4DS_F2FFC249F | 0.9942584 | 4.94E-05 |
| lncRNA_014538 | Traes_4DS_F2FFC249F | 0.9908781 | 0.0001244 |
| lncRNA_074434 | Traes_4DS_F2FFC249F | 0.9933785 | 6.56E-05 |
| lncRNA_009362 | Traes_4DS_F7F6E4552 | -0.990144 | 0.0001452 |
| lncRNA_043877 | Traes_4DS_FAF06B392 | 0.9914156 | 0.0001102 |
| lncRNA_024152 | Traes_5AL_056A80914 | 0.9923441 | 8.77E-05 |
| lncRNA_039420 | Traes_5AL_0591A6D88 | -0.991477 | 0.0001087 |
| lncRNA_074837 | Traes_5AL_0591A6D88 | 0.9956072 | 2.89E-05 |
| lncRNA_022554 | Traes_5AL_099F2FB96 | 0.9937045 | 5.93E-05 |
| lncRNA_048256 | Traes_5AL_099F2FB96 | 0.9949597 | 3.80E-05 |
| lncRNA_048256 | Traes_5AL_19DBD0B75 | -0.991248 | 0.0001146 |
| TRAES3BF052700300CFD_g | Traes_5AL_1A6CFF268 | -0.994393 | 4.71E-05 |
| lncRNA_014501 | Traes_5AL_1BA61DAB5 | -0.991076 | 0.0001191 |
| lncRNA_020959 | Traes_5AL_1BA61DAB5 | -0.994206 | 5.03E-05 |
| lncRNA_024807 | Traes_5AL_1BA61DAB5 | -0.997404 | 1.01E-05 |
| lncRNA_061738 | Traes_5AL_1BA61DAB5 | -0.998197 | 4.87E-06 |
| TRAES3BF052700300CFD_g | Traes_5AL_1C77D8859 | -0.994277 | 4.90E-05 |
| lncRNA_029088 | Traes_5AL_2DC07A5CF | 0.9944242 | 4.65E-05 |
| lncRNA_081387 | Traes_5AL_3517C0CE2 | -0.991751 | 0.0001018 |
| lncRNA_022554 | Traes_5AL_3803F504A | 0.9901283 | 0.0001457 |
| lncRNA_048256 | Traes_5AL_3803F504A | 0.9944107 | 4.68E-05 |
| lncRNA_063009 | Traes_5AL_3803F504A | 0.9945086 | 4.52E-05 |
| lncRNA_001839 | Traes_5AL_3BD3DF934 | -0.995084 | 3.62E-05 |
| lncRNA_014538 | Traes_5AL_3C74F0AAC | 0.9951943 | 3.46E-05 |
| lncRNA_074434 | Traes_5AL_3C74F0AAC | 0.9948812 | 3.92E-05 |
| lncRNA_080683 | Traes_5AL_3E0C865DF | 0.992507 | 8.40E-05 |
| TRAES3BF053100050CFD_g | Traes_5AL_542D62D3E | -0.993203 | 6.91E-05 |
| lncRNA_007700 | Traes_5AL_542D62D3E | -0.992545 | 8.32E-05 |
| lncRNA_013190 | Traes_5AL_542D62D3E | -0.995651 | 2.83E-05 |
| lncRNA_018078 | Traes_5AL_542D62D3E | -0.997899 | 6.62E-06 |
| lncRNA_043877 | Traes_5AL_542D62D3E | -0.994513 | 4.51E-05 |
| lncRNA_048256 | Traes_5AL_542D62D3E | -0.991972 | 9.64E-05 |
| lncRNA_007700 | Traes_5AL_55CFC4B56 | 0.9967221 | 1.61E-05 |
| lncRNA_018078 | Traes_5AL_55CFC4B56 | 0.9952511 | 3.38E-05 |
| lncRNA_009698 | Traes_5AL_5A2A6E1FF | -0.990423 | 0.0001371 |
| lncRNA_048256 | Traes_5AL_5A2A6E1FF | -0.991058 | 0.0001196 |
| lncRNA_024152 | Traes_5AL_5A3A592D9 | -0.992101 | 9.33E-05 |
| lncRNA_047461 | Traes_5AL_5A3A592D9 | -0.993621 | 6.09E-05 |
| lncRNA_006011 | Traes_5AL_6284FBBD8 | 0.9964678 | 1.87E-05 |
| lncRNA_074837 | Traes_5AL_6284FBBD8 | 0.9954361 | 3.12E-05 |
| TRAES3BF052700300CFD_g | Traes_5AL_629EDDFC3 | -0.996625 | 1.71E-05 |
| lncRNA_047461 | Traes_5AL_629EDDFC3 | -0.995103 | 3.59E-05 |
| lncRNA_001839 | Traes_5AL_65FC8CBE2 | -0.990919 | 0.0001233 |
| lncRNA_068995 | Traes_5AL_68844CE9B | 0.9961006 | 2.28E-05 |
| lncRNA_022554 | Traes_5AL_68A0BD67A | -0.990312 | 0.0001403 |
| lncRNA_059323 | Traes_5AL_6B2E76ED8 | -0.992495 | 8.43E-05 |
| TRAES3BF053100050CFD_g | Traes_5AL_6D9620C16 | 0.9923535 | 8.75E-05 |
| lncRNA_018078 | Traes_5AL_6D9620C16 | 0.9942838 | 4.89E-05 |
| lncRNA_051318 | Traes_5AL_6D9620C16 | 0.9906841 | 0.0001298 |
| lncRNA_063127 | Traes_5AL_6D9620C16 | -0.992915 | 7.51E-05 |
| lncRNA_080648 | Traes_5AL_6D9620C16 | 0.9904483 | 0.0001364 |
| Traes_1BS_B58657408 | Traes_5AL_6F83AEE98 | -0.995183 | 3.48E-05 |
| lncRNA_018078 | Traes_5AL_81745FA28 | -0.993668 | 6.00E-05 |
| lncRNA_051318 | Traes_5AL_81745FA28 | -0.996276 | 2.08E-05 |
| lncRNA_063127 | Traes_5AL_81745FA28 | 0.9951424 | 3.53E-05 |
| lncRNA_014538 | Traes_5AL_8282D7D5A | 0.992732 | 7.90E-05 |
| lncRNA_049300 | Traes_5AL_8282D7D5A | 0.9957498 | 2.71E-05 |
| lncRNA_005569 | Traes_5AL_8349DE248 | -0.996841 | 1.50E-05 |
| lncRNA_014501 | Traes_5AL_8990624B8 | 0.9957192 | 2.74E-05 |
| lncRNA_024807 | Traes_5AL_8990624B8 | 0.9944176 | 4.67E-05 |
| lncRNA_061738 | Traes_5AL_8990624B8 | 0.9953057 | 3.30E-05 |
| lncRNA_006361 | Traes_5AL_90BCD474E | 0.9994775 | 4.09E-07 |
| lncRNA_012610 | Traes_5AL_90BCD474E | 0.9960304 | 2.36E-05 |
| lncRNA_015487 | Traes_5AL_90BCD474E | 0.9956741 | 2.80E-05 |
| lncRNA_018111 | Traes_5AL_90BCD474E | 0.9933963 | 6.53E-05 |
| lncRNA_001557 | Traes_5AL_96E30FD9B | 0.9972504 | 1.13E-05 |
| lncRNA_047317 | Traes_5AL_9E1236CE1 | 0.9908295 | 0.0001258 |
| lncRNA_017776 | Traes_5AL_A041A47C4 | -0.99361 | 6.11E-05 |
| lncRNA_006361 | Traes_5AL_ACB202E80 | -0.991889 | 9.84E-05 |
| lncRNA_012610 | Traes_5AL_ACB202E80 | -0.990902 | 0.0001238 |
| lncRNA_015487 | Traes_5AL_ACB202E80 | -0.995809 | 2.63E-05 |
| lncRNA_018111 | Traes_5AL_ACB202E80 | -0.998975 | 1.58E-06 |
| lncRNA_026968 | Traes_5AL_B4B3A41C8 | 0.9907808 | 0.0001271 |
| lncRNA_078349 | Traes_5AL_B4B3A41C8 | 0.9943479 | 4.78E-05 |
| lncRNA_072440 | Traes_5AL_BB55F989A | 0.9985702 | 3.06E-06 |
| lncRNA_046989 | Traes_5AL_BBEF740F0 | 0.9940418 | 5.31E-05 |
| lncRNA_020959 | Traes_5AL_BCA187DFF | 0.9946792 | 4.24E-05 |
| lncRNA_024807 | Traes_5AL_BCA187DFF | 0.9928475 | 7.66E-05 |
| lncRNA_029013 | Traes_5AL_BCA187DFF | 0.990322 | 0.00014 |
| lncRNA_029013 | Traes_5AL_C9159E011 | -0.992573 | 8.25E-05 |
| lncRNA_063127 | Traes_5AL_C9159E011 | 0.9958303 | 2.60E-05 |
| lncRNA_080648 | Traes_5AL_C9159E011 | -0.993629 | 6.08E-05 |
| TRAES3BF053100050CFD_g | Traes_5AL_CA4D25841 | 0.9935282 | 6.27E-05 |
| lncRNA_007700 | Traes_5AL_CA4D25841 | 0.9924147 | 8.61E-05 |
| lncRNA_013190 | Traes_5AL_CA4D25841 | 0.9965938 | 1.74E-05 |
| lncRNA_018078 | Traes_5AL_CA4D25841 | 0.994941 | 3.83E-05 |
| lncRNA_048256 | Traes_5AL_CA4D25841 | 0.993636 | 6.06E-05 |
| lncRNA_059554 | Traes_5AL_CA4D25841 | 0.9912946 | 0.0001133 |
| lncRNA_024812 | Traes_5AL_CBB3EE721 | 0.9916578 | 0.0001041 |
| lncRNA_069396 | Traes_5AL_D1358AF6E | -0.99052 | 0.0001344 |
| lncRNA_009293 | Traes_5AL_D78221B31 | 0.991555 | 0.0001067 |
| lncRNA_029384 | Traes_5AL_D98C7CE92 | 0.9902375 | 0.0001425 |
| lncRNA_006361 | Traes_5AL_DABA58392 | -0.991878 | 9.87E-05 |
| lncRNA_012610 | Traes_5AL_DABA58392 | -0.99488 | 3.93E-05 |
| lncRNA_015487 | Traes_5AL_DABA58392 | -0.992326 | 8.81E-05 |
| lncRNA_018111 | Traes_5AL_DABA58392 | -0.999634 | 2.01E-07 |
| Traes_1BS_B58657408 | Traes_5AL_DE8EB60C0 | -0.990348 | 0.0001393 |
| lncRNA_048668 | Traes_5AL_DF30DE757 | 0.9920656 | 9.42E-05 |
| lncRNA_039325 | Traes_5AL_E03FF5EC3 | 0.9930815 | 7.16E-05 |
| lncRNA_048320 | Traes_5AL_E4E5B111A | 0.9931776 | 6.97E-05 |
| lncRNA_029384 | Traes_5AL_E5FB83F56 | 0.9982594 | 4.54E-06 |
| lncRNA_024152 | Traes_5AL_EFC792322 | -0.993815 | 5.73E-05 |
| lncRNA_017776 | Traes_5AL_F3BAE66F1 | -0.990226 | 0.0001428 |
| lncRNA_012242 | Traes_5AL_F96AEDDB8 | 0.9945088 | 4.51E-05 |
| TRAES3BF021800100CFD_g | Traes_5AL_F9A06C1F7 | 0.9957592 | 2.69E-05 |
| lncRNA_045725 | Traes_5AL_F9A06C1F7 | 0.9988966 | 1.83E-06 |
| lncRNA_051428 | Traes_5AL_F9A06C1F7 | 0.9988966 | 1.83E-06 |
| lncRNA_057614 | Traes_5AL_F9A06C1F7 | 0.9988966 | 1.83E-06 |
| lncRNA_065747 | Traes_5AL_F9A06C1F7 | 0.9988966 | 1.83E-06 |
| lncRNA_044308 | Traes_5AL_FC1665DA7 | -0.990001 | 0.0001495 |
| lncRNA_012242 | Traes_5AL_FE34AC127 | 0.994092 | 5.23E-05 |
| lncRNA_054317 | Traes_5AL_FE34AC127 | 0.9970475 | 1.31E-05 |
| lncRNA_063009 | Traes_5AL_FE34AC127 | 0.9976507 | 8.27E-06 |
| lncRNA_054623 | Traes_5AL_FEF4F30AA | 0.9909805 | 0.0001217 |
| lncRNA_058136 | Traes_5AS_178DFC4E3 | 0.9920653 | 9.42E-05 |
| lncRNA_008977 | Traes_5AS_20FB9ECDA | -0.991297 | 0.0001133 |
| lncRNA_024152 | Traes_5AS_20FB9ECDA | -0.995084 | 3.62E-05 |
| lncRNA_001557 | Traes_5AS_41309F3D0 | 0.9948499 | 3.97E-05 |
| lncRNA_009293 | Traes_5AS_509D1D882 | 0.991805 | 0.0001005 |
| lncRNA_022554 | Traes_5AS_51DBC1F49 | -0.994439 | 4.63E-05 |
| TRAES3BF053100050CFD_g | Traes_5AS_9236CB587 | 0.9908747 | 0.0001245 |
| lncRNA_059554 | Traes_5AS_9236CB587 | 0.9973904 | 1.02E-05 |
| lncRNA_008977 | Traes_5AS_9689E345A | -0.992448 | 8.53E-05 |
| lncRNA_048256 | Traes_5AS_9689E345A | -0.990369 | 0.0001387 |
| lncRNA_063009 | Traes_5AS_9689E345A | -0.991498 | 0.0001081 |
| lncRNA_084019 | Traes_5AS_9C4E6CE77 | -0.990793 | 0.0001268 |
| lncRNA_005569 | Traes_5AS_B85789CFB | -0.995092 | 3.61E-05 |
| lncRNA_013190 | Traes_5AS_EDF185C68 | 0.9951736 | 3.49E-05 |
| lncRNA_018078 | Traes_5AS_EDF185C68 | 0.9947842 | 4.07E-05 |
| lncRNA_048256 | Traes_5AS_EDF185C68 | 0.9927949 | 7.77E-05 |
| lncRNA_051318 | Traes_5AS_EDF185C68 | 0.997225 | 1.15E-05 |
| lncRNA_022554 | Traes_5BL_010D71116 | 0.9909564 | 0.0001223 |
| lncRNA_051318 | Traes_5BL_025C74BDB | -0.99568 | 2.80E-05 |
| lncRNA_058136 | Traes_5BL_025C74BDB | 0.9916078 | 0.0001053 |
| lncRNA_009362 | Traes_5BL_0999942FA | -0.994767 | 4.10E-05 |
| lncRNA_068995 | Traes_5BL_0B5C3EEC6 | 0.9915226 | 0.0001075 |
| lncRNA_013231 | Traes_5BL_0BFAE33B9 | 0.9938604 | 5.64E-05 |
| lncRNA_010675 | Traes_5BL_10BF821D1 | 0.9906736 | 0.0001301 |
| lncRNA_006859 | Traes_5BL_1126D831F | 0.9907135 | 0.000129 |
| lncRNA_012608 | Traes_5BL_1126D831F | 0.9977216 | 7.78E-06 |
| lncRNA_051888 | Traes_5BL_1315614DC | -0.996096 | 2.28E-05 |
| lncRNA_053766 | Traes_5BL_1315614DC | 0.995185 | 3.47E-05 |
| lncRNA_014538 | Traes_5BL_13AE34978 | 0.9924569 | 8.51E-05 |
| lncRNA_074434 | Traes_5BL_13AE34978 | 0.9924122 | 8.61E-05 |
| lncRNA_029528 | Traes_5BL_1505DCD78 | -0.994879 | 3.93E-05 |
| lncRNA_084019 | Traes_5BL_1505DCD78 | -0.991748 | 0.0001019 |
| lncRNA_037690 | Traes_5BL_177EB593A | 0.9936976 | 5.95E-05 |
| lncRNA_024812 | Traes_5BL_194ED46CE | 0.9920278 | 9.51E-05 |
| lncRNA_013190 | Traes_5BL_1CF10603C | -0.995562 | 2.95E-05 |
| lncRNA_048256 | Traes_5BL_1CF10603C | -0.999366 | 6.03E-07 |
| lncRNA_007700 | Traes_5BL_20059327B | 0.9921165 | 9.30E-05 |
| lncRNA_029013 | Traes_5BL_20059327B | 0.9964994 | 1.84E-05 |
| lncRNA_010675 | Traes_5BL_223CEEDBA | 0.991305 | 0.0001131 |
| TRAES3BF053100050CFD_g | Traes_5BL_281A4A114 | 0.997744 | 7.63E-06 |
| lncRNA_013190 | Traes_5BL_281A4A114 | 0.9909198 | 0.0001233 |
| lncRNA_018078 | Traes_5BL_281A4A114 | 0.9915547 | 0.0001067 |
| lncRNA_080648 | Traes_5BL_281A4A114 | 0.9920358 | 9.49E-05 |
| TRAES3BF052700300CFD_g | Traes_5BL_29F85D616 | -0.991981 | 9.62E-05 |
| lncRNA_043943 | Traes_5BL_2BBD58AA9 | 0.990764 | 0.0001276 |
| lncRNA_076127 | Traes_5BL_36B495DAF | 0.9987761 | 2.25E-06 |
| lncRNA_012242 | Traes_5BL_36EBD512B | -0.996087 | 2.29E-05 |
| lncRNA_017751 | Traes_5BL_36EBD512B | -0.990348 | 0.0001393 |
| lncRNA_025391 | Traes_5BL_36EBD512B | -0.990978 | 0.0001217 |
| lncRNA_054317 | Traes_5BL_36EBD512B | -0.992329 | 8.80E-05 |
| lncRNA_014538 | Traes_5BL_3892C17DC | 0.9942024 | 5.03E-05 |
| lncRNA_005569 | Traes_5BL_38D5C4BE1 | 0.994731 | 4.16E-05 |
| lncRNA_017776 | Traes_5BL_38D5C4BE1 | -0.992696 | 7.98E-05 |
| lncRNA_020477 | Traes_5BL_3AC8B8F76 | 0.9940528 | 5.29E-05 |
| lncRNA_054824 | Traes_5BL_3B390119D | 0.9954132 | 3.15E-05 |
| lncRNA_048266 | Traes_5BL_3D522D8D2 | -0.990586 | 0.0001325 |
| lncRNA_020959 | Traes_5BL_3E60DDEA1 | -0.9947 | 4.21E-05 |
| lncRNA_024807 | Traes_5BL_3E60DDEA1 | -0.994545 | 4.46E-05 |
| lncRNA_044308 | Traes_5BL_3E60DDEA1 | -0.994041 | 5.32E-05 |
| lncRNA_022554 | Traes_5BL_3E817DD44 | -0.994193 | 5.05E-05 |
| lncRNA_063009 | Traes_5BL_3E817DD44 | -0.991068 | 0.0001193 |
| lncRNA_039420 | Traes_5BL_41A447CB6 | -0.995289 | 3.32E-05 |
| lncRNA_006361 | Traes_5BL_47994EFBD | -0.994459 | 4.60E-05 |
| lncRNA_012610 | Traes_5BL_47994EFBD | -0.991944 | 9.71E-05 |
| lncRNA_015487 | Traes_5BL_47994EFBD | -0.998192 | 4.90E-06 |
| lncRNA_018111 | Traes_5BL_47994EFBD | -0.997513 | 9.27E-06 |
| lncRNA_022554 | Traes_5BL_479A5DFA0 | -0.995428 | 3.13E-05 |
| lncRNA_072935 | Traes_5BL_48A35287E | 0.9949187 | 3.87E-05 |
| lncRNA_009293 | Traes_5BL_5135C4ABA | 0.9922179 | 9.06E-05 |
| lncRNA_014504 | Traes_5BL_565873505 | -0.991311 | 0.0001129 |
| lncRNA_019725 | Traes_5BL_565873505 | -0.996843 | 1.49E-05 |
| lncRNA_059554 | Traes_5BL_5A9F5F455 | 0.9949035 | 3.89E-05 |
| lncRNA_012610 | Traes_5BL_62D9B877B | 0.9927583 | 7.85E-05 |
| lncRNA_015487 | Traes_5BL_62D9B877B | 0.9942612 | 4.93E-05 |
| lncRNA_018111 | Traes_5BL_62D9B877B | 0.9901794 | 0.0001442 |
| lncRNA_048256 | Traes_5BL_62D9B877B | 0.9921473 | 9.23E-05 |
| lncRNA_054317 | Traes_5BL_62D9B877B | 0.9912167 | 0.0001154 |
| lncRNA_063009 | Traes_5BL_62D9B877B | 0.9980752 | 5.55E-06 |
| lncRNA_017776 | Traes_5BL_634C3B43F | -0.992066 | 9.42E-05 |
| lncRNA_012610 | Traes_5BL_648E3BB39 | -0.992332 | 8.80E-05 |
| lncRNA_018111 | Traes_5BL_648E3BB39 | -0.99366 | 6.02E-05 |
| lncRNA_012242 | Traes_5BL_64C4C409F | -0.996859 | 1.48E-05 |
| lncRNA_072935 | Traes_5BL_6647931E2 | 0.9993743 | 5.87E-07 |
| lncRNA_044556 | Traes_5BL_667D47F86 | 0.9901767 | 0.0001443 |
| lncRNA_008977 | Traes_5BL_679C187B0 | 0.9981777 | 4.98E-06 |
| lncRNA_013190 | Traes_5BL_67DAB39A6 | 0.9966745 | 1.66E-05 |
| lncRNA_022554 | Traes_5BL_67DAB39A6 | 0.9930329 | 7.26E-05 |
| lncRNA_048256 | Traes_5BL_67DAB39A6 | 0.9937176 | 5.91E-05 |
| lncRNA_051318 | Traes_5BL_67DAB39A6 | 0.9910575 | 0.0001196 |
| lncRNA_047461 | Traes_5BL_71C9BC55C | 0.9957873 | 2.66E-05 |
| TRAES3BF052700300CFD_g | Traes_5BL_71F88DB88 | -0.996174 | 2.19E-05 |
| TRAES3BF052700300CFD_g | Traes_5BL_733D552E81 | -0.99066 | 0.0001304 |
| lncRNA_014501 | Traes_5BL_733D552E81 | -0.995331 | 3.26E-05 |
| lncRNA_024807 | Traes_5BL_733D552E81 | -0.993226 | 6.87E-05 |
| lncRNA_061738 | Traes_5BL_733D552E81 | -0.997924 | 6.46E-06 |
| lncRNA_032287 | Traes_5BL_775D43287 | -0.999328 | 6.78E-07 |
| lncRNA_051551 | Traes_5BL_7C7817ACA | 0.9940492 | 5.30E-05 |
| lncRNA_039420 | Traes_5BL_7D5EC9052 | -0.998728 | 2.42E-06 |
| lncRNA_066190 | Traes_5BL_7D8CED1FF | 0.9923223 | 8.82E-05 |
| lncRNA_013231 | Traes_5BL_8512C24F7 | 0.9984004 | 3.84E-06 |
| lncRNA_044556 | Traes_5BL_8512C24F7 | 0.9929467 | 7.44E-05 |
| lncRNA_053766 | Traes_5BL_86CA8C1DD | 0.9950367 | 3.69E-05 |
| lncRNA_020959 | Traes_5BL_8B9B2D843 | 0.9937377 | 5.87E-05 |
| lncRNA_024807 | Traes_5BL_8B9B2D843 | 0.9954526 | 3.10E-05 |
| lncRNA_061738 | Traes_5BL_8B9B2D843 | 0.9951551 | 3.52E-05 |
| lncRNA_076127 | Traes_5BL_8C2F32515 | 0.9945989 | 4.37E-05 |
| lncRNA_009362 | Traes_5BL_8CB3F7560 | -0.991082 | 0.0001189 |
| lncRNA_039420 | Traes_5BL_8DF4FE5A9 | -0.993184 | 6.95E-05 |
| lncRNA_013053 | Traes_5BL_8FD1FB0E1 | 0.9931263 | 7.07E-05 |
| lncRNA_058136 | Traes_5BL_8FD1FB0E1 | 0.9956455 | 2.84E-05 |
| lncRNA_022554 | Traes_5BL_9059C131F | 0.9913485 | 0.0001119 |
| lncRNA_029384 | Traes_5BL_917DF323B | 0.9942322 | 4.98E-05 |
| lncRNA_000823 | Traes_5BL_922358DB7 | 0.9935345 | 6.26E-05 |
| lncRNA_013190 | Traes_5BL_97BEC5636 | 0.9946856 | 4.23E-05 |
| lncRNA_018078 | Traes_5BL_97BEC5636 | 0.9954723 | 3.07E-05 |
| lncRNA_048256 | Traes_5BL_97BEC5636 | 0.9913232 | 0.0001126 |
| lncRNA_051318 | Traes_5BL_97BEC5636 | 0.9975901 | 8.70E-06 |
| TRAES3BF053100050CFD_g | Traes_5BL_98F07B12D | 0.9920267 | 9.51E-05 |
| lncRNA_013190 | Traes_5BL_98F07B12D | 0.9968649 | 1.47E-05 |
| lncRNA_018078 | Traes_5BL_98F07B12D | 0.9981668 | 5.04E-06 |
| lncRNA_051318 | Traes_5BL_98F07B12D | 0.9983524 | 4.07E-06 |
| lncRNA_063127 | Traes_5BL_98F07B12D | -0.992793 | 7.77E-05 |
| lncRNA_047461 | Traes_5BL_999585B72 | -0.990039 | 0.0001483 |
| lncRNA_024152 | Traes_5BL_99AB709F2 | -0.990999 | 0.0001212 |
| lncRNA_051318 | Traes_5BL_9CC98DD38 | -0.992454 | 8.52E-05 |
| lncRNA_006011 | Traes_5BL_9F4C228C1 | 0.9916831 | 0.0001035 |
| TRAES3BF052700300CFD_g | Traes_5BL_A0350B6AB | -0.990942 | 0.0001227 |
| lncRNA_014501 | Traes_5BL_A0350B6AB | -0.990031 | 0.0001486 |
| lncRNA_047461 | Traes_5BL_A0350B6AB | -0.998303 | 4.31E-06 |
| lncRNA_068995 | Traes_5BL_A8B4F5EC6 | 0.9919243 | 9.76E-05 |
| lncRNA_012242 | Traes_5BL_AD05117E7 | -0.991743 | 0.000102 |
| lncRNA_054317 | Traes_5BL_AD05117E7 | -0.99208 | 9.39E-05 |
| lncRNA_044581 | Traes_5BL_AE99FDF89 | 0.9947996 | 4.05E-05 |
| lncRNA_000928 | Traes_5BL_B27792F88 | 0.9905718 | 0.0001329 |
| lncRNA_029384 | Traes_5BL_B27792F88 | 0.9948746 | 3.93E-05 |
| lncRNA_044308 | Traes_5BL_B5395A54A | -0.990316 | 0.0001402 |
| lncRNA_061738 | Traes_5BL_B5395A54A | -0.990437 | 0.0001367 |
| lncRNA_029384 | Traes_5BL_B84D64E12 | 0.9973254 | 1.07E-05 |
| lncRNA_084019 | Traes_5BL_C41169E6A | -0.992495 | 8.43E-05 |
| lncRNA_066381 | Traes_5BL_CC850DA60 | 0.9907232 | 0.0001287 |
| lncRNA_024152 | Traes_5BL_CDB206601 | -0.993723 | 5.90E-05 |
| lncRNA_001839 | Traes_5BL_CE4CF3B5C | -0.996987 | 1.36E-05 |
| TRAES3BF021800100CFD_g | Traes_5BL_CE73B6BFF | 0.9914674 | 0.0001089 |
| lncRNA_045725 | Traes_5BL_CE73B6BFF | 0.9999422 | 5.01E-09 |
| lncRNA_051428 | Traes_5BL_CE73B6BFF | 0.9999422 | 5.01E-09 |
| lncRNA_057614 | Traes_5BL_CE73B6BFF | 0.9999422 | 5.01E-09 |
| lncRNA_065747 | Traes_5BL_CE73B6BFF | 0.9999422 | 5.01E-09 |
| lncRNA_076127 | Traes_5BL_CF34FCA90 | 0.9943109 | 4.85E-05 |
| lncRNA_015487 | Traes_5BL_D190954CB | -0.991893 | 9.83E-05 |
| lncRNA_047461 | Traes_5BL_D8D745F37 | -0.991223 | 0.0001152 |
| lncRNA_007700 | Traes_5BL_DF8C0753E | 0.9904197 | 0.0001372 |
| lncRNA_013190 | Traes_5BL_DF8C0753E | 0.9902543 | 0.000142 |
| lncRNA_018078 | Traes_5BL_DF8C0753E | 0.992481 | 8.46E-05 |
| lncRNA_043877 | Traes_5BL_DF8C0753E | 0.9923054 | 8.86E-05 |
| lncRNA_048256 | Traes_5BL_DF8C0753E | 0.9912315 | 0.000115 |
| lncRNA_045725 | Traes_5BL_E3BC16326 | 0.996417 | 1.92E-05 |
| lncRNA_051428 | Traes_5BL_E3BC16326 | 0.996417 | 1.92E-05 |
| lncRNA_057614 | Traes_5BL_E3BC16326 | 0.996417 | 1.92E-05 |
| lncRNA_065747 | Traes_5BL_E3BC16326 | 0.996417 | 1.92E-05 |
| lncRNA_015322 | Traes_5BL_E77938325 | 0.991371 | 0.0001114 |
| lncRNA_044556 | Traes_5BL_E9DE35F831 | 0.998736 | 2.40E-06 |
| lncRNA_000928 | Traes_5BL_EA85CB6BC | 0.9926231 | 8.14E-05 |
| lncRNA_029384 | Traes_5BL_EA85CB6BC | 0.9964455 | 1.89E-05 |
| lncRNA_026968 | Traes_5BL_EDB3E6AD6 | 0.9975074 | 9.31E-06 |
| lncRNA_078349 | Traes_5BL_EDB3E6AD6 | 0.9982784 | 4.44E-06 |
| lncRNA_006361 | Traes_5BL_EFB550AE3 | -0.99029 | 0.000141 |
| lncRNA_051318 | Traes_5BL_EFB550AE3 | -0.990758 | 0.0001277 |
| TRAES3BF117100150CFD_g | Traes_5BL_F0C37D1AE1 | 0.9949253 | 3.86E-05 |
| lncRNA_012610 | Traes_5BL_F0C37D1AE1 | -0.991252 | 0.0001145 |
| lncRNA_033754 | Traes_5BL_F0C37D1AE1 | -0.99879 | 2.19E-06 |
| lncRNA_006361 | Traes_5BL_F0C94BA2D | -0.99013 | 0.0001456 |
| lncRNA_015487 | Traes_5BL_F0C94BA2D | -0.996552 | 1.78E-05 |
| lncRNA_018111 | Traes_5BL_F0C94BA2D | -0.99226 | 8.96E-05 |
| lncRNA_068995 | Traes_5BL_F44E89105 | -0.993571 | 6.19E-05 |
| lncRNA_029384 | Traes_5BL_F5D379AFC | 0.9920067 | 9.56E-05 |
| lncRNA_029528 | Traes_5BL_F664C293E | 0.9921913 | 9.12E-05 |
| lncRNA_009293 | Traes_5BL_F6D090701 | 0.9922448 | 9.00E-05 |
| TRAES3BF053100050CFD_g | Traes_5BL_F7E6E35B8 | -0.994502 | 4.53E-05 |
| lncRNA_007700 | Traes_5BL_F7E6E35B8 | -0.992992 | 7.35E-05 |
| lncRNA_043877 | Traes_5BL_F7E6E35B8 | -0.993993 | 5.40E-05 |
| lncRNA_059554 | Traes_5BL_F7E6E35B8 | -0.991699 | 0.0001031 |
| lncRNA_080648 | Traes_5BL_F7E6E35B8 | -0.990037 | 0.0001484 |
| lncRNA_006361 | Traes_5BS_19E54A2E0 | -0.993733 | 5.88E-05 |
| lncRNA_032048 | Traes_5BS_1D2221052 | 0.9964597 | 1.88E-05 |
| lncRNA_001557 | Traes_5BS_24494BD87 | 0.9957519 | 2.70E-05 |
| Traes_1BS_B58657408 | Traes_5BS_366B42DDB | 0.99614 | 2.23E-05 |
| lncRNA_013190 | Traes_5BS_4179BB1C5 | -0.991157 | 0.000117 |
| lncRNA_018078 | Traes_5BS_4179BB1C5 | -0.994654 | 4.28E-05 |
| lncRNA_019725 | Traes_5BS_4179BB1C5 | -0.99449 | 4.55E-05 |
| lncRNA_043877 | Traes_5BS_4179BB1C5 | -0.993369 | 6.58E-05 |
| lncRNA_051318 | Traes_5BS_4179BB1C5 | -0.992431 | 8.57E-05 |
| lncRNA_063127 | Traes_5BS_4179BB1C5 | 0.9904764 | 0.0001356 |
| lncRNA_019725 | Traes_5BS_6BC7A44AD | 0.9968143 | 1.52E-05 |
| lncRNA_047461 | Traes_5BS_72DE7510F | 0.9941814 | 5.07E-05 |
| lncRNA_010675 | Traes_5BS_825C8B39E | -0.996021 | 2.37E-05 |
| lncRNA_014501 | Traes_5BS_825C8B39E | 0.9920144 | 9.54E-05 |
| lncRNA_009293 | Traes_5BS_86C1C9A9F | -0.990551 | 0.0001335 |
| TRAES3BF053100050CFD_g | Traes_5BS_92F775461 | 0.9941527 | 5.12E-05 |
| lncRNA_007700 | Traes_5BS_92F775461 | 0.9912681 | 0.000114 |
| lncRNA_013190 | Traes_5BS_92F775461 | 0.9912915 | 0.0001134 |
| lncRNA_018078 | Traes_5BS_92F775461 | 0.9993531 | 6.28E-07 |
| lncRNA_043877 | Traes_5BS_92F775461 | 0.9956654 | 2.81E-05 |
| lncRNA_051318 | Traes_5BS_92F775461 | 0.9925111 | 8.39E-05 |
| lncRNA_063127 | Traes_5BS_92F775461 | -0.995473 | 3.07E-05 |
| lncRNA_080648 | Traes_5BS_92F775461 | 0.9927032 | 7.97E-05 |
| lncRNA_014501 | Traes_5BS_99ED7E919 | -0.991369 | 0.0001114 |
| lncRNA_047461 | Traes_5BS_99ED7E919 | -0.991643 | 0.0001045 |
| lncRNA_006011 | Traes_5BS_AD7853E99 | 0.9959025 | 2.52E-05 |
| lncRNA_017777 | Traes_5BS_AFCAA8A89 | -0.994095 | 5.22E-05 |
| lncRNA_005569 | Traes_5BS_B593A6FB7 | 0.9946627 | 4.27E-05 |
| lncRNA_072440 | Traes_5BS_BC276D951 | 0.9957024 | 2.77E-05 |
| lncRNA_080648 | Traes_5BS_F4517F3F8 | -0.996247 | 2.11E-05 |
| lncRNA_048825 | Traes_5BS_F51F454B1 | 0.9926169 | 8.16E-05 |
| lncRNA_014501 | Traes_5BS_F93273CED | -0.991413 | 0.0001103 |
| lncRNA_024807 | Traes_5DL_0ED2854E2 | -0.990393 | 0.000138 |
| lncRNA_022554 | Traes_5DL_11F618A4D | -0.99155 | 0.0001068 |
| lncRNA_018111 | Traes_5DL_128F9DE77 | -0.995705 | 2.76E-05 |
| lncRNA_074165 | Traes_5DL_134F29727 | 0.9949673 | 3.79E-05 |
| lncRNA_004117 | Traes_5DL_20A09D99E | 0.9955248 | 3.00E-05 |
| lncRNA_046989 | Traes_5DL_20FF8C565 | 0.9916771 | 0.0001036 |
| lncRNA_000823 | Traes_5DL_21529A726 | 0.9949929 | 3.75E-05 |
| lncRNA_068995 | Traes_5DL_23362968A | 0.990317 | 0.0001402 |
| Traes_1BS_B58657408 | Traes_5DL_24E161E8F | -0.993353 | 6.61E-05 |
| lncRNA_009293 | Traes_5DL_24E161E8F | 0.9907377 | 0.0001283 |
| lncRNA_020477 | Traes_5DL_24E161E8F | 0.9921107 | 9.31E-05 |
| lncRNA_072935 | Traes_5DL_2548F67F5 | 0.9965849 | 1.75E-05 |
| lncRNA_048256 | Traes_5DL_25A67F16E | -0.994408 | 4.68E-05 |
| Traes_1BS_B58657408 | Traes_5DL_25C753D84 | -0.990484 | 0.0001354 |
| lncRNA_018111 | Traes_5DL_277B71A63 | -0.991273 | 0.0001139 |
| lncRNA_048668 | Traes_5DL_28CE15993 | 0.997197 | 1.18E-05 |
| lncRNA_009698 | Traes_5DL_2D0C11D58 | -0.990949 | 0.0001225 |
| lncRNA_034367 | Traes_5DL_2D0C11D58 | -0.992602 | 8.19E-05 |
| Traes_1BS_B58657408 | Traes_5DL_2D0D83A55 | -0.991753 | 0.0001018 |
| lncRNA_006011 | Traes_5DL_2D0D83A55 | 0.9927851 | 7.79E-05 |
| lncRNA_039420 | Traes_5DL_2D0D83A55 | -0.992192 | 9.12E-05 |
| lncRNA_074837 | Traes_5DL_2D0D83A55 | 0.9907504 | 0.0001279 |
| lncRNA_034367 | Traes_5DL_309650024 | -0.991699 | 0.0001031 |
| TRAES3BF021800100CFD_g | Traes_5DL_343F3EE59 | 0.9904065 | 0.0001376 |
| lncRNA_045725 | Traes_5DL_343F3EE59 | 0.9959443 | 2.46E-05 |
| lncRNA_051428 | Traes_5DL_343F3EE59 | 0.9959443 | 2.46E-05 |
| lncRNA_057614 | Traes_5DL_343F3EE59 | 0.9959443 | 2.46E-05 |
| lncRNA_065747 | Traes_5DL_343F3EE59 | 0.9959443 | 2.46E-05 |
| lncRNA_039319 | Traes_5DL_398615728 | -0.993106 | 7.11E-05 |
| lncRNA_048668 | Traes_5DL_39A62425E | 0.9953484 | 3.24E-05 |
| TRAES3BF052700300CFD_g | Traes_5DL_412D1CEF6 | 0.9918202 | 0.0001001 |
| lncRNA_061738 | Traes_5DL_412D1CEF6 | 0.9932547 | 6.81E-05 |
| lncRNA_072440 | Traes_5DL_4186C5347 | 0.997718 | 7.81E-06 |
| Traes_1BS_B58657408 | Traes_5DL_46901C98E | -0.990772 | 0.0001274 |
| lncRNA_030379 | Traes_5DL_491E0EE41 | 0.9945622 | 4.43E-05 |
| lncRNA_014504 | Traes_5DL_4C2E23565 | 0.9954643 | 3.08E-05 |
| lncRNA_043877 | Traes_5DL_4C2E23565 | 0.990631 | 0.0001313 |
| lncRNA_015322 | Traes_5DL_5D1620AA5 | 0.9911858 | 0.0001162 |
| lncRNA_039420 | Traes_5DL_5F0417763 | -0.991018 | 0.0001207 |
| lncRNA_074837 | Traes_5DL_5F0417763 | 0.9980977 | 5.42E-06 |
| lncRNA_013053 | Traes_5DL_5F402393B | 0.9930069 | 7.32E-05 |
| lncRNA_008784 | Traes_5DL_62CE05147 | -0.990905 | 0.0001237 |
| lncRNA_032048 | Traes_5DL_62CE05147 | 0.9921367 | 9.25E-05 |
| lncRNA_009698 | Traes_5DL_69DE08EB5 | -0.996372 | 1.97E-05 |
| lncRNA_012610 | Traes_5DL_69DE08EB5 | -0.991228 | 0.0001151 |
| lncRNA_013190 | Traes_5DL_69DE08EB5 | -0.996008 | 2.39E-05 |
| lncRNA_048256 | Traes_5DL_69DE08EB5 | -0.996937 | 1.41E-05 |
| lncRNA_009293 | Traes_5DL_736BC1383 | 0.9970551 | 1.30E-05 |
| lncRNA_059554 | Traes_5DL_7514D124D | 0.9911848 | 0.0001162 |
| lncRNA_066381 | Traes_5DL_753A1DCA0 | 0.9948753 | 3.93E-05 |
| lncRNA_054317 | Traes_5DL_75912CDD6 | 0.9961424 | 2.23E-05 |
| lncRNA_048825 | Traes_5DL_7B261BB16 | 0.9921549 | 9.21E-05 |
| lncRNA_049669 | Traes_5DL_7B261BB16 | 0.9969265 | 1.42E-05 |
| lncRNA_026968 | Traes_5DL_7BE279972 | 0.9972714 | 1.12E-05 |
| lncRNA_063547 | Traes_5DL_7BE279972 | 0.994476 | 4.57E-05 |
| lncRNA_078349 | Traes_5DL_7BE279972 | 0.9977693 | 7.46E-06 |
| lncRNA_029013 | Traes_5DL_80A6FDB52 | 0.9981725 | 5.01E-06 |
| lncRNA_063127 | Traes_5DL_80A6FDB52 | -0.990766 | 0.0001275 |
| TRAES3BF052700300CFD_g | Traes_5DL_834E4B05E | -0.995438 | 3.12E-05 |
| lncRNA_047461 | Traes_5DL_834E4B05E | -0.99254 | 8.33E-05 |
| lncRNA_072935 | Traes_5DL_91AE6CA271 | 0.9935585 | 6.21E-05 |
| lncRNA_081387 | Traes_5DL_99B0AF349 | -0.990321 | 0.0001401 |
| lncRNA_006085 | Traes_5DL_A2A5A19B4 | 0.9968589 | 1.48E-05 |
| lncRNA_032287 | Traes_5DL_A2A5A19B4 | 0.9935766 | 6.18E-05 |
| lncRNA_033754 | Traes_5DL_A2A5A19B4 | 0.9914015 | 0.0001106 |
| lncRNA_048368 | Traes_5DL_A6EC2A121 | 0.9917586 | 0.0001016 |
| lncRNA_074837 | Traes_5DL_AC0C3A9BD | 0.9968458 | 1.49E-05 |
| lncRNA_082364 | Traes_5DL_AC5E58001 | 0.9949006 | 3.89E-05 |
| lncRNA_029384 | Traes_5DL_ADFFAE33D | 0.9905528 | 0.0001335 |
| lncRNA_063127 | Traes_5DL_B38FE5CB3 | 0.9923179 | 8.83E-05 |
| lncRNA_081387 | Traes_5DL_B4752381D | -0.992027 | 9.51E-05 |
| lncRNA_014504 | Traes_5DL_B5491B1D9 | -0.993791 | 5.77E-05 |
| lncRNA_043877 | Traes_5DL_B5491B1D9 | -0.993952 | 5.47E-05 |
| lncRNA_018078 | Traes_5DL_BB61F5EC0 | 0.9902269 | 0.0001428 |
| TRAES3BF177500010CFD_g | Traes_5DL_BFB1E5328 | 0.9949749 | 3.78E-05 |
| lncRNA_024812 | Traes_5DL_C05F32EE4 | 0.9962128 | 2.15E-05 |
| TRAES3BF052700300CFD_g | Traes_5DL_C381C2BE4 | -0.991326 | 0.0001125 |
| lncRNA_009362 | Traes_5DL_C3AE031ED | -0.997921 | 6.48E-06 |
| lncRNA_068995 | Traes_5DL_C8B663B86 | 0.9932318 | 6.86E-05 |
| lncRNA_009362 | Traes_5DL_CD95EFB8F | -0.995412 | 3.15E-05 |
| lncRNA_039420 | Traes_5DL_CE81982AA | -0.993043 | 7.24E-05 |
| lncRNA_006361 | Traes_5DL_D34420AE3 | -0.993817 | 5.72E-05 |
| lncRNA_018111 | Traes_5DL_D34420AE3 | -0.990358 | 0.000139 |
| lncRNA_084019 | Traes_5DL_D38E31F121 | -0.992888 | 7.57E-05 |
| lncRNA_024812 | Traes_5DL_D4AC8C679 | 0.9906544 | 0.0001306 |
| lncRNA_000928 | Traes_5DL_DAE12FAE0 | 0.9901797 | 0.0001442 |
| lncRNA_083996 | Traes_5DL_DB8A076AB | 0.9901666 | 0.0001446 |
| lncRNA_059323 | Traes_5DL_E2AF5FEA6 | -0.99289 | 7.57E-05 |
| lncRNA_022554 | Traes_5DL_E4168273F | -0.992159 | 9.20E-05 |
| lncRNA_063127 | Traes_5DL_E7DBBB54A | 0.991457 | 0.0001092 |
| lncRNA_063547 | Traes_5DL_E7DBBB54A | -0.990654 | 0.0001306 |
| lncRNA_022554 | Traes_5DL_EADC17A7D | -0.992028 | 9.51E-05 |
| lncRNA_047461 | Traes_5DL_ECC866A4B1 | -0.994552 | 4.44E-05 |
| lncRNA_007700 | Traes_5DL_F13C5B5C6 | 0.9917575 | 0.0001016 |
| lncRNA_018078 | Traes_5DL_F13C5B5C6 | 0.9906471 | 0.0001308 |
| lncRNA_020959 | Traes_5DL_F13C5B5C6 | 0.9907654 | 0.0001275 |
| lncRNA_024807 | Traes_5DL_F13C5B5C6 | 0.9907316 | 0.0001285 |
| lncRNA_059323 | Traes_5DL_FB19DD1BE | -0.992411 | 8.62E-05 |
| lncRNA_081387 | Traes_5DL_FB19DD1BE | -0.995649 | 2.83E-05 |
| lncRNA_074658 | Traes_5DS_2F6D9BD24 | 0.9958558 | 2.57E-05 |
| lncRNA_032287 | Traes_5DS_3B00C6225 | 0.9933112 | 6.70E-05 |
| lncRNA_015487 | Traes_5DS_42A1A9BAA | 0.9907676 | 0.0001275 |
| lncRNA_051318 | Traes_5DS_42A1A9BAA | 0.9947851 | 4.07E-05 |
| lncRNA_018111 | Traes_5DS_48052B9FC | -0.992788 | 7.78E-05 |
| lncRNA_024152 | Traes_5DS_48052B9FC | -0.993804 | 5.75E-05 |
| lncRNA_039420 | Traes_5DS_581B4DB50 | -0.997752 | 7.58E-06 |
| lncRNA_072935 | Traes_5DS_643ED07F6 | -0.990443 | 0.0001366 |
| lncRNA_008500 | Traes_5DS_64C30A803 | 0.9940489 | 5.30E-05 |
| lncRNA_014501 | Traes_5DS_7722ED6BA | 0.9951637 | 3.50E-05 |
| lncRNA_061738 | Traes_5DS_7722ED6BA | 0.9942987 | 4.87E-05 |
| lncRNA_074658 | Traes_5DS_7722ED6BA | 0.9908418 | 0.0001254 |
| lncRNA_043877 | Traes_5DS_79791EF20 | 0.990113 | 0.0001461 |
| lncRNA_080648 | Traes_5DS_79791EF20 | 0.9963759 | 1.97E-05 |
| lncRNA_039943 | Traes_5DS_7A0ECD4AB | 0.991368 | 0.0001114 |
| TRAES3BF117100150CFD_g | Traes_5DS_9277AC663 | -0.996977 | 1.37E-05 |
| lncRNA_006011 | Traes_5DS_B1785331F | 0.9918048 | 0.0001005 |
| lncRNA_017776 | Traes_5DS_B9BFD5BEC | 0.9942684 | 4.92E-05 |
| lncRNA_024152 | Traes_5DS_BE4C8D436 | 0.9957982 | 2.64E-05 |
| lncRNA_007700 | Traes_5DS_BF18807E5 | -0.993109 | 7.11E-05 |
| lncRNA_072440 | Traes_5DS_DF8E59628 | 0.9932066 | 6.91E-05 |
| lncRNA_017751 | Traes_5DS_E289017BD | -0.997883 | 6.72E-06 |
| lncRNA_006361 | Traes_6AL_01DB61EF6 | -0.995785 | 2.66E-05 |
| lncRNA_012610 | Traes_6AL_01DB61EF6 | -0.996134 | 2.24E-05 |
| lncRNA_013190 | Traes_6AL_01DB61EF6 | -0.993525 | 6.27E-05 |
| lncRNA_015487 | Traes_6AL_01DB61EF6 | -0.993563 | 6.20E-05 |
| lncRNA_018111 | Traes_6AL_01DB61EF6 | -0.99026 | 0.0001419 |
| lncRNA_051318 | Traes_6AL_01DB61EF6 | -0.995638 | 2.85E-05 |
| lncRNA_001839 | Traes_6AL_033D6C680 | 0.9947335 | 4.15E-05 |
| lncRNA_051318 | Traes_6AL_0361DD7E9 | 0.9927809 | 7.80E-05 |
| lncRNA_007700 | Traes_6AL_074141030 | 0.9919389 | 9.72E-05 |
| lncRNA_015322 | Traes_6AL_077524EE1 | 0.9950923 | 3.61E-05 |
| lncRNA_012242 | Traes_6AL_0BCC7C4C8 | -0.990822 | 0.000126 |
| lncRNA_064639 | Traes_6AL_0BCC7C4C8 | -0.994991 | 3.76E-05 |
| lncRNA_015487 | Traes_6AL_0C0899C15 | -0.992894 | 7.56E-05 |
| lncRNA_018111 | Traes_6AL_0C0899C15 | -0.994889 | 3.91E-05 |
| lncRNA_012610 | Traes_6AL_116E0BA24 | 0.9925343 | 8.34E-05 |
| lncRNA_013190 | Traes_6AL_116E0BA24 | 0.9946631 | 4.26E-05 |
| lncRNA_048256 | Traes_6AL_116E0BA24 | 0.9943853 | 4.72E-05 |
| lncRNA_051318 | Traes_6AL_116E0BA24 | 0.9951951 | 3.46E-05 |
| lncRNA_024807 | Traes_6AL_1571A073E | -0.991622 | 0.000105 |
| lncRNA_061738 | Traes_6AL_1571A073E | -0.991141 | 0.0001174 |
| lncRNA_046989 | Traes_6AL_1CF4D8935 | 0.990688 | 0.0001297 |
| lncRNA_048256 | Traes_6AL_1CF4D8935 | -0.992297 | 8.88E-05 |
| lncRNA_012610 | Traes_6AL_2017727C4 | -0.992925 | 7.49E-05 |
| lncRNA_013190 | Traes_6AL_2017727C4 | -0.997118 | 1.24E-05 |
| lncRNA_018078 | Traes_6AL_2017727C4 | -0.992674 | 8.03E-05 |
| lncRNA_051318 | Traes_6AL_2017727C4 | -0.998766 | 2.28E-06 |
| lncRNA_008977 | Traes_6AL_212F055E4 | -0.990074 | 0.0001473 |
| lncRNA_024152 | Traes_6AL_212F055E4 | -0.992929 | 7.48E-05 |
| Traes_1BS_B58657408 | Traes_6AL_23918FB79 | -0.995224 | 3.42E-05 |
| lncRNA_009293 | Traes_6AL_23918FB79 | 0.9948098 | 4.03E-05 |
| lncRNA_015322 | Traes_6AL_23B31796F | 0.9938176 | 5.72E-05 |
| lncRNA_014538 | Traes_6AL_32FADD06A | 0.9911313 | 0.0001176 |
| TRAES3BF060500060CFD_g | Traes_6AL_38BACA187 | 0.9900813 | 0.0001471 |
| lncRNA_000833 | Traes_6AL_4986D4968 | 0.9937618 | 5.83E-05 |
| lncRNA_066381 | Traes_6AL_4986D4968 | 0.9927961 | 7.77E-05 |
| lncRNA_053766 | Traes_6AL_55DCB41C2 | 0.9916893 | 0.0001033 |
| lncRNA_013190 | Traes_6AL_5940122BE | 0.9950895 | 3.61E-05 |
| lncRNA_018078 | Traes_6AL_5940122BE | 0.998228 | 4.71E-06 |
| lncRNA_051318 | Traes_6AL_5940122BE | 0.9974318 | 9.89E-06 |
| lncRNA_063127 | Traes_6AL_5940122BE | -0.992824 | 7.71E-05 |
| lncRNA_032287 | Traes_6AL_5BFD42A7C | -0.994584 | 4.39E-05 |
| lncRNA_064639 | Traes_6AL_5BFD42A7C | -0.995302 | 3.31E-05 |
| lncRNA_068995 | Traes_6AL_5E348F7AB | 0.9926568 | 8.07E-05 |
| lncRNA_013053 | Traes_6AL_643CE8D7C | 0.9956873 | 2.79E-05 |
| lncRNA_020477 | Traes_6AL_697008F53 | 0.995802 | 2.64E-05 |
| lncRNA_039943 | Traes_6AL_6EB10C68E | 0.9938694 | 5.63E-05 |
| lncRNA_053766 | Traes_6AL_6EB10C68E | 0.9928413 | 7.67E-05 |
| lncRNA_022554 | Traes_6AL_72D2B7C35 | 0.9975829 | 8.76E-06 |
| lncRNA_000823 | Traes_6AL_7B0512B64 | 0.9919213 | 9.76E-05 |
| lncRNA_009698 | Traes_6AL_920563984 | -0.990235 | 0.0001426 |
| lncRNA_012610 | Traes_6AL_920563984 | -0.993369 | 6.58E-05 |
| lncRNA_013190 | Traes_6AL_920563984 | -0.995621 | 2.87E-05 |
| lncRNA_048256 | Traes_6AL_920563984 | -0.992207 | 9.09E-05 |
| lncRNA_051318 | Traes_6AL_920563984 | -0.997126 | 1.24E-05 |
| lncRNA_059323 | Traes_6AL_939CC781A | -0.995268 | 3.35E-05 |
| lncRNA_009698 | Traes_6AL_9437F730F | -0.990155 | 0.0001449 |
| lncRNA_048256 | Traes_6AL_9437F730F | -0.996545 | 1.79E-05 |
| lncRNA_021433 | Traes_6AL_986248807 | -0.994004 | 5.38E-05 |
| lncRNA_044308 | Traes_6AL_986248807 | -0.991425 | 0.00011 |
| lncRNA_048668 | Traes_6AL_9AC15F055 | 0.9905082 | 0.0001347 |
| lncRNA_083996 | Traes_6AL_9B873CB22 | 0.9906946 | 0.0001295 |
| lncRNA_081387 | Traes_6AL_9E0046317 | -0.993171 | 6.98E-05 |
| lncRNA_074837 | Traes_6AL_A69551CA61 | 0.993061 | 7.21E-05 |
| lncRNA_024812 | Traes_6AL_B79748FE8 | 0.9903821 | 0.0001383 |
| lncRNA_006011 | Traes_6AL_D0315EFD0 | 0.991928 | 9.75E-05 |
| lncRNA_074837 | Traes_6AL_D0315EFD0 | 0.9904961 | 0.0001351 |
| TRAES3BF024700270CFD_g | Traes_6AL_DC2A1F000 | 0.9960506 | 2.34E-05 |
| lncRNA_022554 | Traes_6AL_E3E3F32E0 | 0.9937487 | 5.85E-05 |
| lncRNA_016209 | Traes_6AL_E54708BB8 | 0.9960298 | 2.36E-05 |
| lncRNA_054824 | Traes_6AL_E54708BB8 | 0.9985166 | 3.30E-06 |
| lncRNA_073681 | Traes_6AL_E54708BB8 | 0.9955961 | 2.90E-05 |
| lncRNA_074165 | Traes_6AL_E54708BB8 | 0.9901291 | 0.0001457 |
| TRAES3BF052700300CFD_g | Traes_6AL_E9A292FCD | -0.992013 | 9.54E-05 |
| lncRNA_063009 | Traes_6AL_E9A292FCD | -0.99045 | 0.0001364 |
| lncRNA_057390 | Traes_6AL_ED2D3DFDF | -0.990655 | 0.0001306 |
| lncRNA_014504 | Traes_6AL_F0CA40293 | 0.9905053 | 0.0001348 |
| lncRNA_020959 | Traes_6AL_F0CA40293 | 0.9904004 | 0.0001378 |
| lncRNA_000928 | Traes_6AL_F25923EFA | 0.9912738 | 0.0001139 |
| lncRNA_039943 | Traes_6AL_F25923EFA | 0.9944023 | 4.69E-05 |
| lncRNA_058136 | Traes_6AL_F25923EFA | 0.9966558 | 1.68E-05 |
| lncRNA_020477 | Traes_6AL_F3638D620 | 0.9909336 | 0.0001229 |
| lncRNA_007700 | Traes_6AL_F53BA3D0C | 0.9933157 | 6.69E-05 |
| lncRNA_018078 | Traes_6AL_F53BA3D0C | 0.9953423 | 3.25E-05 |
| lncRNA_029013 | Traes_6AL_F53BA3D0C | 0.9938854 | 5.60E-05 |
| lncRNA_043877 | Traes_6AL_F53BA3D0C | 0.9909333 | 0.0001229 |
| lncRNA_063127 | Traes_6AL_F53BA3D0C | -0.996021 | 2.37E-05 |
| lncRNA_080648 | Traes_6AL_F53BA3D0C | 0.992362 | 8.73E-05 |
| lncRNA_007700 | Traes_6AL_FBFBA69E9 | -0.994571 | 4.41E-05 |
| lncRNA_046989 | Traes_6AL_FE462C6F8 | 0.9948115 | 4.03E-05 |
| lncRNA_074837 | Traes_6AL_FE462C6F8 | 0.9922265 | 9.04E-05 |
| lncRNA_037690 | Traes_6AS_077A9B3E1 | 0.9914801 | 0.0001086 |
| lncRNA_014501 | Traes_6AS_0A0B33CEF | -0.997022 | 1.33E-05 |
| lncRNA_061738 | Traes_6AS_0A0B33CEF | -0.995994 | 2.40E-05 |
| lncRNA_022554 | Traes_6AS_174DF9EAB | 0.9961349 | 2.24E-05 |
| lncRNA_022554 | Traes_6AS_263764436 | 0.9956573 | 2.82E-05 |
| lncRNA_015322 | Traes_6AS_2A59D8EDC | 0.9930429 | 7.24E-05 |
| lncRNA_024152 | Traes_6AS_2C86173661 | -0.996601 | 1.73E-05 |
| lncRNA_009698 | Traes_6AS_32423383C | -0.99103 | 0.0001203 |
| lncRNA_012610 | Traes_6AS_32423383C | -0.992221 | 9.05E-05 |
| lncRNA_013190 | Traes_6AS_32423383C | -0.99683 | 1.51E-05 |
| lncRNA_018078 | Traes_6AS_32423383C | -0.992942 | 7.46E-05 |
| lncRNA_048256 | Traes_6AS_32423383C | -0.990414 | 0.0001374 |
| lncRNA_051318 | Traes_6AS_32423383C | -0.998178 | 4.98E-06 |
| lncRNA_039420 | Traes_6AS_375237984 | -0.998609 | 2.90E-06 |
| lncRNA_044556 | Traes_6AS_37E167F1A | -0.994392 | 4.71E-05 |
| lncRNA_015322 | Traes_6AS_390A9B9F4 | 0.9903793 | 0.0001384 |
| lncRNA_019725 | Traes_6AS_390A9B9F4 | -0.991745 | 0.0001019 |
| lncRNA_001839 | Traes_6AS_428546CD6 | -0.996493 | 1.84E-05 |
| lncRNA_001839 | Traes_6AS_4A7414EA9 | 0.9926946 | 7.99E-05 |
| lncRNA_006361 | Traes_6AS_4C5D39772 | -0.993112 | 7.10E-05 |
| lncRNA_015487 | Traes_6AS_4C5D39772 | -0.995567 | 2.94E-05 |
| lncRNA_051318 | Traes_6AS_4C5D39772 | -0.992687 | 8.00E-05 |
| lncRNA_039943 | Traes_6AS_4E111F83D | 0.993765 | 5.82E-05 |
| lncRNA_059323 | Traes_6AS_547E4724D | 0.99497 | 3.79E-05 |
| lncRNA_044581 | Traes_6AS_5F02CA081 | 0.9924614 | 8.50E-05 |
| lncRNA_048419 | Traes_6AS_5F02CA081 | 0.9975186 | 9.23E-06 |
| lncRNA_053766 | Traes_6AS_621A7A571 | 0.9915097 | 0.0001078 |
| TRAES3BF177500010CFD_g | Traes_6AS_69A0BEB68 | 0.9914442 | 0.0001095 |
| lncRNA_048368 | Traes_6AS_69A0BEB68 | 0.9916654 | 0.0001039 |
| lncRNA_006859 | Traes_6AS_706F252FA | 0.992288 | 8.90E-05 |
| lncRNA_012608 | Traes_6AS_706F252FA | 0.9929418 | 7.46E-05 |
| lncRNA_006859 | Traes_6AS_730ED4A10 | 0.9920003 | 9.57E-05 |
| lncRNA_039420 | Traes_6AS_7B7ECDCB3 | -0.996241 | 2.12E-05 |
| lncRNA_006361 | Traes_6AS_7C785BA32 | -0.992982 | 7.37E-05 |
| lncRNA_018111 | Traes_6AS_7C785BA32 | -0.995018 | 3.72E-05 |
| TRAES3BF021800100CFD_g | Traes_6AS_7FB8F9A66 | 0.9937814 | 5.79E-05 |
| lncRNA_006361 | Traes_6AS_8B3190A99 | -0.991037 | 0.0001201 |
| lncRNA_039943 | Traes_6AS_901774D0F | 0.9923475 | 8.76E-05 |
| lncRNA_058136 | Traes_6AS_901774D0F | 0.991479 | 0.0001086 |
| lncRNA_006361 | Traes_6AS_976D36CDD | -0.997039 | 1.31E-05 |
| lncRNA_015487 | Traes_6AS_976D36CDD | -0.997756 | 7.55E-06 |
| lncRNA_018111 | Traes_6AS_976D36CDD | -0.996122 | 2.25E-05 |
| lncRNA_020477 | Traes_6AS_991B780D1 | 0.9918499 | 9.94E-05 |
| lncRNA_048668 | Traes_6AS_991B780D1 | 0.996233 | 2.13E-05 |
| lncRNA_053766 | Traes_6AS_991B780D1 | 0.9900865 | 0.0001469 |
| lncRNA_047461 | Traes_6AS_9B913DF6F | 0.9957093 | 2.76E-05 |
| TRAES3BF117100150CFD_g | Traes_6AS_A42E71A00 | 0.9916238 | 0.0001049 |
| lncRNA_000928 | Traes_6AS_A883BC2B1 | 0.99473 | 4.16E-05 |
| lncRNA_009698 | Traes_6AS_A883BC2B1 | -0.993622 | 6.09E-05 |
| lncRNA_014538 | Traes_6AS_A9E51FA04 | 0.990908 | 0.0001236 |
| lncRNA_024152 | Traes_6AS_AA9004965 | -0.994281 | 4.90E-05 |
| lncRNA_014504 | Traes_6AS_B3BE9F388 | -0.996047 | 2.34E-05 |
| lncRNA_012608 | Traes_6AS_CC5E41F10 | 0.994306 | 4.85E-05 |
| lncRNA_046989 | Traes_6AS_CEA02191C | 0.9931471 | 7.03E-05 |
| lncRNA_009698 | Traes_6AS_CF6D8CD28 | -0.991422 | 0.0001101 |
| lncRNA_048256 | Traes_6AS_CF6D8CD28 | -0.990324 | 0.00014 |
| TRAES3BF177500010CFD_g | Traes_6AS_D09BF5BF8 | 0.9912614 | 0.0001142 |
| lncRNA_048419 | Traes_6AS_D09BF5BF8 | 0.9924245 | 8.59E-05 |
| lncRNA_008977 | Traes_6AS_D49C93E84 | -0.991046 | 0.0001199 |
| lncRNA_001839 | Traes_6AS_D7528CBFE | -0.990476 | 0.0001356 |
| lncRNA_081387 | Traes_6AS_D81ED78CF | -0.991789 | 0.0001009 |
| lncRNA_048266 | Traes_6AS_E2BC4706B | -0.993397 | 6.53E-05 |
| TRAES3BF052700300CFD_g | Traes_6AS_F8A11E678 | 0.9952534 | 3.37E-05 |
| lncRNA_008977 | Traes_6AS_F8A11E678 | 0.9903779 | 0.0001384 |
| TRAES3BF052700300CFD_g | Traes_6AS_FAFAAFD12 | -0.997816 | 7.15E-06 |
| lncRNA_061738 | Traes_6AS_FAFAAFD12 | -0.990345 | 0.0001394 |
| lncRNA_006361 | Traes_6BL_09104A767 | -0.991184 | 0.0001162 |
| lncRNA_022554 | Traes_6BL_14F6009C3 | 0.9969527 | 1.39E-05 |
| lncRNA_048256 | Traes_6BL_14F6009C3 | 0.9900293 | 0.0001486 |
| lncRNA_008977 | Traes_6BL_22FB1D3F4 | 0.991983 | 9.62E-05 |
| lncRNA_047461 | Traes_6BL_2546C9F2A | 0.9959369 | 2.47E-05 |
| lncRNA_013231 | Traes_6BL_29F6A3588 | 0.9939111 | 5.55E-05 |
| lncRNA_082364 | Traes_6BL_29F6A3588 | 0.9910815 | 0.000119 |
| lncRNA_048668 | Traes_6BL_2B7EC4BEA | 0.9945323 | 4.48E-05 |
| lncRNA_051888 | Traes_6BL_2B7EC4BEA | -0.990279 | 0.0001413 |
| lncRNA_022554 | Traes_6BL_2BD543A76 | 0.9937453 | 5.86E-05 |
| lncRNA_048256 | Traes_6BL_2BD543A76 | 0.9907482 | 0.000128 |
| lncRNA_024152 | Traes_6BL_34B081BBE | -0.993118 | 7.09E-05 |
| lncRNA_006859 | Traes_6BL_3BE1AC22A | 0.9934867 | 6.35E-05 |
| lncRNA_006361 | Traes_6BL_40771391A | -0.995274 | 3.35E-05 |
| lncRNA_015487 | Traes_6BL_40771391A | -0.991211 | 0.0001155 |
| lncRNA_012242 | Traes_6BL_478B0A257 | -0.99173 | 0.0001023 |
| lncRNA_024812 | Traes_6BL_5068A26B6 | 0.9947616 | 4.11E-05 |
| lncRNA_059323 | Traes_6BL_51A18A82E | -0.996617 | 1.71E-05 |
| lncRNA_069396 | Traes_6BL_55CCE9D5A | 0.992857 | 7.64E-05 |
| lncRNA_024152 | Traes_6BL_56372A914 | -0.997778 | 7.40E-06 |
| lncRNA_034367 | Traes_6BL_56D72F728 | 0.9933685 | 6.58E-05 |
| lncRNA_007700 | Traes_6BL_5B70744B1 | 0.9908753 | 0.0001245 |
| lncRNA_013190 | Traes_6BL_5B70744B1 | 0.9901746 | 0.0001443 |
| lncRNA_018078 | Traes_6BL_5B70744B1 | 0.9967154 | 1.62E-05 |
| lncRNA_051318 | Traes_6BL_5B70744B1 | 0.9949519 | 3.82E-05 |
| lncRNA_063127 | Traes_6BL_5B70744B1 | -0.993243 | 6.83E-05 |
| lncRNA_044308 | Traes_6BL_63FB12617 | 0.9965276 | 1.81E-05 |
| lncRNA_026968 | Traes_6BL_6CA698871 | 0.9900327 | 0.0001485 |
| lncRNA_024152 | Traes_6BL_79F146A90 | -0.996692 | 1.64E-05 |
| lncRNA_020959 | Traes_6BL_8B9E1AEAF | 0.9965281 | 1.81E-05 |
| lncRNA_024807 | Traes_6BL_8B9E1AEAF | 0.9949158 | 3.87E-05 |
| lncRNA_039420 | Traes_6BL_8D21A769D | -0.994457 | 4.60E-05 |
| lncRNA_016209 | Traes_6BL_905A374B2 | 0.9975506 | 8.99E-06 |
| lncRNA_074165 | Traes_6BL_905A374B2 | 0.9956639 | 2.82E-05 |
| TRAES3BF053100050CFD_g | Traes_6BL_95C7F7123 | -0.997761 | 7.52E-06 |
| lncRNA_080648 | Traes_6BL_95C7F7123 | -0.994089 | 5.23E-05 |
| lncRNA_009698 | Traes_6BL_A3E9C02F0 | 0.9917435 | 0.000102 |
| lncRNA_013190 | Traes_6BL_A3E9C02F0 | 0.9911883 | 0.0001161 |
| lncRNA_034367 | Traes_6BL_A3E9C02F0 | 0.9919863 | 9.61E-05 |
| lncRNA_000928 | Traes_6BL_B1B21B021 | 0.9947163 | 4.18E-05 |
| lncRNA_015322 | Traes_6BL_B1B21B021 | 0.9938489 | 5.66E-05 |
| lncRNA_019725 | Traes_6BL_B1B21B021 | -0.996746 | 1.59E-05 |
| lncRNA_009698 | Traes_6BL_D26547564 | 0.9924889 | 8.44E-05 |
| lncRNA_018078 | Traes_6BL_D26547564 | 0.9910619 | 0.0001195 |
| lncRNA_019725 | Traes_6BL_D26547564 | 0.994893 | 3.91E-05 |
| lncRNA_043877 | Traes_6BL_D26547564 | 0.9942308 | 4.98E-05 |
| lncRNA_007700 | Traes_6BL_D93192C47 | 0.9928114 | 7.73E-05 |
| lncRNA_006361 | Traes_6BL_E9173BD9B | 0.9909185 | 0.0001233 |
| lncRNA_012610 | Traes_6BL_E9173BD9B | 0.99741 | 1.01E-05 |
| lncRNA_013190 | Traes_6BL_E9173BD9B | 0.9937002 | 5.94E-05 |
| lncRNA_015487 | Traes_6BL_E9173BD9B | 0.9945677 | 4.42E-05 |
| lncRNA_018111 | Traes_6BL_E9173BD9B | 0.994742 | 4.14E-05 |
| lncRNA_048256 | Traes_6BL_E9173BD9B | 0.9918502 | 9.94E-05 |
| lncRNA_051318 | Traes_6BL_E9173BD9B | 0.9929157 | 7.51E-05 |
| lncRNA_063009 | Traes_6BL_E9173BD9B | 0.9903966 | 0.0001379 |
| lncRNA_000833 | Traes_6BL_F321C9050 | 0.9956725 | 2.81E-05 |
| lncRNA_013190 | Traes_6BL_F4597CA77 | 0.9939999 | 5.39E-05 |
| lncRNA_018078 | Traes_6BL_F4597CA77 | 0.9952869 | 3.33E-05 |
| lncRNA_048256 | Traes_6BL_F4597CA77 | 0.9905626 | 0.0001332 |
| lncRNA_051318 | Traes_6BL_F4597CA77 | 0.998175 | 4.99E-06 |
| lncRNA_063127 | Traes_6BL_F4597CA77 | -0.990287 | 0.0001411 |
| lncRNA_008977 | Traes_6BS_01CD46D81 | -0.9945 | 4.53E-05 |
| lncRNA_029013 | Traes_6BS_1BC106302 | -0.998283 | 4.42E-06 |
| lncRNA_063547 | Traes_6BS_1BC106302 | -0.99366 | 6.02E-05 |
| lncRNA_078349 | Traes_6BS_1BC106302 | -0.990629 | 0.0001313 |
| lncRNA_059323 | Traes_6BS_257A12B34 | -0.995454 | 3.10E-05 |
| lncRNA_032048 | Traes_6BS_2848AC23A | 0.9901048 | 0.0001464 |
| lncRNA_022554 | Traes_6BS_399B7B111 | 0.9908144 | 0.0001262 |
| lncRNA_008977 | Traes_6BS_4B652CCA0 | -0.99118 | 0.0001164 |
| lncRNA_013231 | Traes_6BS_4DA724E79 | 0.9923623 | 8.73E-05 |
| lncRNA_082364 | Traes_6BS_4DA724E79 | 0.9916976 | 0.0001031 |
| lncRNA_039420 | Traes_6BS_5CF265676 | -0.990721 | 0.0001288 |
| lncRNA_014501 | Traes_6BS_6D2A626DE | -0.99512 | 3.57E-05 |
| TRAES3BF053100050CFD_g | Traes_6BS_6D9026EAE | 0.9994228 | 5.00E-07 |
| lncRNA_013190 | Traes_6BS_6D9026EAE | 0.993131 | 7.06E-05 |
| lncRNA_018078 | Traes_6BS_6D9026EAE | 0.9962807 | 2.07E-05 |
| lncRNA_043877 | Traes_6BS_6D9026EAE | 0.992395 | 8.65E-05 |
| lncRNA_080648 | Traes_6BS_6D9026EAE | 0.995389 | 3.18E-05 |
| lncRNA_015322 | Traes_6BS_8020617A51 | 0.9919997 | 9.58E-05 |
| lncRNA_006011 | Traes_6BS_81C9A2EC2 | 0.9930998 | 7.13E-05 |
| lncRNA_000823 | Traes_6BS_85E5E342C | 0.997235 | 1.15E-05 |
| lncRNA_029088 | Traes_6BS_896A8401E | 0.9908389 | 0.0001255 |
| lncRNA_000928 | Traes_6BS_8CB3E58AE | 0.9915254 | 0.0001074 |
| lncRNA_058136 | Traes_6BS_8CB3E58AE | 0.9960743 | 2.31E-05 |
| lncRNA_039943 | Traes_6BS_8DA290AC9 | 0.9900015 | 0.0001495 |
| lncRNA_009362 | Traes_6BS_9752E1BBC | 0.990354 | 0.0001391 |
| lncRNA_063547 | Traes_6BS_A6D7B4C7F | 0.9904222 | 0.0001372 |
| lncRNA_008977 | Traes_6BS_B38F89538 | -0.994793 | 4.06E-05 |
| lncRNA_048256 | Traes_6BS_B38F89538 | -0.990827 | 0.0001258 |
| lncRNA_063009 | Traes_6BS_B38F89538 | -0.994792 | 4.06E-05 |
| lncRNA_068995 | Traes_6BS_C6217BCE3 | 0.9902923 | 0.0001409 |
| lncRNA_053766 | Traes_6BS_D3182B77A | 0.9930304 | 7.27E-05 |
| lncRNA_008977 | Traes_6BS_F0D0B2F85 | -0.990624 | 0.0001314 |
| lncRNA_047461 | Traes_6BS_F0D0B2F85 | -0.991345 | 0.000112 |
| lncRNA_018111 | Traes_6BS_F204AC147 | -0.9915 | 0.0001081 |
| Traes_1BS_B58657408 | Traes_6DL_13B51D4DC | -0.99064 | 0.000131 |
| TRAES3BF053100050CFD_g | Traes_6DL_18B245CAB | 0.9948752 | 3.93E-05 |
| lncRNA_007700 | Traes_6DL_18B245CAB | 0.9927711 | 7.82E-05 |
| lncRNA_013190 | Traes_6DL_18B245CAB | 0.9956924 | 2.78E-05 |
| lncRNA_018078 | Traes_6DL_18B245CAB | 0.9997507 | 9.32E-08 |
| lncRNA_043877 | Traes_6DL_18B245CAB | 0.992237 | 9.02E-05 |
| lncRNA_051318 | Traes_6DL_18B245CAB | 0.9948717 | 3.94E-05 |
| lncRNA_063127 | Traes_6DL_18B245CAB | -0.992787 | 7.79E-05 |
| lncRNA_022554 | Traes_6DL_24A8AB125 | -0.997444 | 9.79E-06 |
| TRAES3BF053100050CFD_g | Traes_6DL_2807D89841 | -0.990333 | 0.0001397 |
| lncRNA_015322 | Traes_6DL_28AA9AE0E | 0.9932229 | 6.87E-05 |
| lncRNA_012608 | Traes_6DL_2E3280F2E | 0.9902265 | 0.0001428 |
| lncRNA_014538 | Traes_6DL_2E3280F2E | 0.9910536 | 0.0001197 |
| lncRNA_030379 | Traes_6DL_325F9AE8C1 | 0.9936153 | 6.10E-05 |
| lncRNA_047461 | Traes_6DL_37C85BEEF | -0.990456 | 0.0001362 |
| lncRNA_039420 | Traes_6DL_3A44079B9 | -0.994468 | 4.58E-05 |
| lncRNA_000928 | Traes_6DL_3CC592FDB | 0.9944691 | 4.58E-05 |
| lncRNA_015322 | Traes_6DL_3CC592FDB | 0.9953553 | 3.23E-05 |
| lncRNA_019725 | Traes_6DL_3CC592FDB | -0.991429 | 0.0001099 |
| lncRNA_000928 | Traes_6DL_3E5249DE3 | 0.9958075 | 2.63E-05 |
| lncRNA_019725 | Traes_6DL_3E5249DE3 | -0.993136 | 7.05E-05 |
| lncRNA_039943 | Traes_6DL_3E5249DE3 | 0.9928543 | 7.64E-05 |
| lncRNA_051888 | Traes_6DL_3E5249DE3 | -0.990941 | 0.0001227 |
| lncRNA_058136 | Traes_6DL_3E5249DE3 | 0.9944758 | 4.57E-05 |
| lncRNA_068995 | Traes_6DL_3ED4C3974 | 0.9905159 | 0.0001345 |
| lncRNA_022554 | Traes_6DL_4B1BCBFD7 | -0.992629 | 8.13E-05 |
| TRAES3BF052700300CFD_g | Traes_6DL_4E727207F | 0.9912242 | 0.0001152 |
| lncRNA_046989 | Traes_6DL_4F21B8BCE | 0.9907089 | 0.0001291 |
| lncRNA_074837 | Traes_6DL_4F21B8BCE | 0.9931595 | 7.00E-05 |
| lncRNA_044556 | Traes_6DL_58620B158 | 0.9925835 | 8.23E-05 |
| lncRNA_074658 | Traes_6DL_58B5347C8 | -0.991875 | 9.87E-05 |
| lncRNA_027360 | Traes_6DL_5C5EC66B5 | 0.9950051 | 3.74E-05 |
| lncRNA_029500 | Traes_6DL_5C5EC66B5 | -0.995295 | 3.31E-05 |
| lncRNA_064639 | Traes_6DL_61AA02A3D | -0.993202 | 6.92E-05 |
| lncRNA_019725 | Traes_6DL_62A15AED6 | -0.995431 | 3.13E-05 |
| lncRNA_022554 | Traes_6DL_669361D62 | -0.996763 | 1.57E-05 |
| lncRNA_046989 | Traes_6DL_67F5944C6 | 0.9914557 | 0.0001092 |
| lncRNA_021433 | Traes_6DL_6DC75B590 | 0.9967337 | 1.60E-05 |
| lncRNA_007700 | Traes_6DL_7398FDE14 | 0.9908426 | 0.0001254 |
| lncRNA_014504 | Traes_6DL_7398FDE14 | 0.9925009 | 8.41E-05 |
| lncRNA_018078 | Traes_6DL_7398FDE14 | 0.9962444 | 2.11E-05 |
| lncRNA_051318 | Traes_6DL_7398FDE14 | 0.9924231 | 8.59E-05 |
| lncRNA_063127 | Traes_6DL_7398FDE14 | -0.991354 | 0.0001118 |
| lncRNA_039325 | Traes_6DL_74673564D | -0.993449 | 6.42E-05 |
| lncRNA_015487 | Traes_6DL_7626A87FF | -0.993238 | 6.84E-05 |
| lncRNA_029013 | Traes_6DL_82C9277E6 | -0.991001 | 0.0001211 |
| lncRNA_063547 | Traes_6DL_82C9277E6 | -0.999071 | 1.29E-06 |
| lncRNA_009698 | Traes_6DL_909B865C0 | 0.9926784 | 8.02E-05 |
| lncRNA_012610 | Traes_6DL_909B865C0 | 0.9946484 | 4.29E-05 |
| lncRNA_013190 | Traes_6DL_909B865C0 | 0.996943 | 1.40E-05 |
| lncRNA_048256 | Traes_6DL_909B865C0 | 0.994674 | 4.25E-05 |
| lncRNA_051318 | Traes_6DL_909B865C0 | 0.9954016 | 3.17E-05 |
| lncRNA_019725 | Traes_6DL_A2771B03C | -0.993608 | 6.12E-05 |
| lncRNA_007700 | Traes_6DL_A48AB792D | 0.9930918 | 7.14E-05 |
| lncRNA_018078 | Traes_6DL_A48AB792D | 0.9913634 | 0.0001116 |
| lncRNA_029013 | Traes_6DL_A48AB792D | 0.9935963 | 6.14E-05 |
| lncRNA_063127 | Traes_6DL_A48AB792D | -0.991732 | 0.0001023 |
| lncRNA_048668 | Traes_6DL_AB95B0CE0 | 0.9940603 | 5.28E-05 |
| lncRNA_053766 | Traes_6DL_AB95B0CE0 | 0.9946673 | 4.26E-05 |
| lncRNA_039289 | Traes_6DL_B280097F0 | -0.994811 | 4.03E-05 |
| lncRNA_029013 | Traes_6DL_B6553E146 | 0.9965022 | 1.83E-05 |
| lncRNA_014538 | Traes_6DL_BC8B75A69 | 0.993639 | 6.06E-05 |
| lncRNA_022554 | Traes_6DL_BEDFBC80F | 0.9961158 | 2.26E-05 |
| lncRNA_048256 | Traes_6DL_BEDFBC80F | 0.9920072 | 9.56E-05 |
| lncRNA_013190 | Traes_6DL_C26461F78 | 0.9921197 | 9.29E-05 |
| lncRNA_018078 | Traes_6DL_C26461F78 | 0.9918121 | 0.0001003 |
| lncRNA_048256 | Traes_6DL_C26461F78 | 0.9906933 | 0.0001295 |
| lncRNA_051318 | Traes_6DL_C26461F78 | 0.997397 | 1.02E-05 |
| lncRNA_001557 | Traes_6DL_C3FEE9DFF | 0.9956056 | 2.89E-05 |
| Traes_1BS_B58657408 | Traes_6DL_C42964D93 | -0.990076 | 0.0001472 |
| lncRNA_054317 | Traes_6DL_C733B2EEA | 0.9922526 | 8.98E-05 |
| TRAES3BF052700300CFD_g | Traes_6DL_CE987E811 | 0.9909337 | 0.0001229 |
| lncRNA_014501 | Traes_6DL_CE987E811 | 0.9921307 | 9.26E-05 |
| lncRNA_024807 | Traes_6DL_CE987E811 | 0.9933722 | 6.57E-05 |
| lncRNA_061738 | Traes_6DL_CE987E811 | 0.9952445 | 3.39E-05 |
| lncRNA_048256 | Traes_6DL_D764400441 | -0.99114 | 0.0001174 |
| lncRNA_059554 | Traes_6DL_D764400441 | -0.99151 | 0.0001078 |
| TRAES3BF024700270CFD_g | Traes_6DL_E00D38C9D | 0.998128 | 5.25E-06 |
| lncRNA_044308 | Traes_6DL_E1A4AEAA0 | -0.993207 | 6.91E-05 |
| lncRNA_054623 | Traes_6DL_E5C93660D | 0.9934842 | 6.35E-05 |
| lncRNA_022554 | Traes_6DL_EAF038D23 | 0.9954163 | 3.15E-05 |
| lncRNA_051888 | Traes_6DL_EFFCA5F6F | -0.992052 | 9.45E-05 |
| lncRNA_010675 | Traes_6DL_F9589C62A | 0.9900575 | 0.0001478 |
| lncRNA_029384 | Traes_6DS_0896B94C8 | 0.991362 | 0.0001116 |
| lncRNA_074658 | Traes_6DS_0AA8FD567 | -0.996707 | 1.63E-05 |
| lncRNA_021433 | Traes_6DS_149D69197 | 0.9947241 | 4.17E-05 |
| lncRNA_044556 | Traes_6DS_149D69197 | 0.9929702 | 7.40E-05 |
| lncRNA_006859 | Traes_6DS_1FDCDEAD0 | 0.991089 | 0.0001188 |
| lncRNA_012608 | Traes_6DS_1FDCDEAD0 | 0.9968882 | 1.45E-05 |
| lncRNA_039420 | Traes_6DS_204661A07 | -0.996833 | 1.50E-05 |
| lncRNA_074837 | Traes_6DS_204661A07 | 0.9934329 | 6.45E-05 |
| lncRNA_013231 | Traes_6DS_2A2F4ED98 | 0.9904003 | 0.0001378 |
| lncRNA_048368 | Traes_6DS_2E767B57A | 0.9921734 | 9.16E-05 |
| lncRNA_012242 | Traes_6DS_474C54942 | -0.99119 | 0.0001161 |
| lncRNA_054317 | Traes_6DS_474C54942 | -0.998013 | 5.92E-06 |
| lncRNA_063009 | Traes_6DS_474C54942 | -0.994415 | 4.67E-05 |
| lncRNA_047317 | Traes_6DS_52C0ACEDB | 0.9903155 | 0.0001402 |
| lncRNA_013053 | Traes_6DS_536814F70 | 0.9902706 | 0.0001415 |
| lncRNA_058136 | Traes_6DS_536814F70 | 0.9938999 | 5.57E-05 |
| TRAES3BF024700270CFD_g | Traes_6DS_569545CCF | 0.9923174 | 8.83E-05 |
| lncRNA_044581 | Traes_6DS_58521AB60 | 0.9916208 | 0.000105 |
| lncRNA_015487 | Traes_6DS_6BD4CA5B2 | -0.991199 | 0.0001159 |
| lncRNA_063009 | Traes_6DS_6BD4CA5B2 | -0.99042 | 0.0001372 |
| lncRNA_048256 | Traes_6DS_6C2C902AB | -0.993794 | 5.77E-05 |
| lncRNA_024152 | Traes_6DS_75C2FBF08 | -0.995287 | 3.33E-05 |
| lncRNA_014504 | Traes_6DS_813EBCBA9 | -0.995432 | 3.12E-05 |
| lncRNA_039420 | Traes_6DS_905702C89 | -0.992451 | 8.53E-05 |
| lncRNA_008977 | Traes_6DS_98585F07B | -0.998107 | 5.37E-06 |
| lncRNA_063009 | Traes_6DS_98585F07B | -0.990377 | 0.0001385 |
| lncRNA_056853 | Traes_6DS_9D2014869 | -0.998843 | 2.01E-06 |
| lncRNA_007700 | Traes_6DS_9FA053DF8 | 0.9923682 | 8.71E-05 |
| TRAES3BF052700300CFD_g | Traes_6DS_A720DE7E0 | -0.996755 | 1.58E-05 |
| lncRNA_061738 | Traes_6DS_A720DE7E0 | -0.990941 | 0.0001227 |
| lncRNA_084019 | Traes_6DS_A7F86156A | -0.990179 | 0.0001442 |
| lncRNA_021433 | Traes_6DS_B73BE95F4 | 0.992378 | 8.69E-05 |
| lncRNA_006011 | Traes_6DS_B8F70E63B | 0.9901066 | 0.0001463 |
| lncRNA_074837 | Traes_6DS_B8F70E63B | 0.9934138 | 6.49E-05 |
| TRAES3BF053100050CFD_g | Traes_6DS_C142E17BD | 0.9948871 | 3.91E-05 |
| lncRNA_080648 | Traes_6DS_C142E17BD | 0.9960707 | 2.31E-05 |
| TRAES3BF052700300CFD_g | Traes_6DS_CDB16CE3F | -0.992537 | 8.33E-05 |
| lncRNA_014501 | Traes_6DS_CDB16CE3F | -0.992744 | 7.88E-05 |
| lncRNA_061738 | Traes_6DS_CDB16CE3F | -0.996774 | 1.56E-05 |
| lncRNA_009293 | Traes_6DS_F0FC6A475 | 0.9907808 | 0.0001271 |
| lncRNA_034367 | Traes_6DS_F5907B97A | 0.9978646 | 6.84E-06 |
| lncRNA_008977 | Traes_7AL_0B358E4B4 | -0.994937 | 3.84E-05 |
| lncRNA_047461 | Traes_7AL_0B358E4B4 | -0.99308 | 7.17E-05 |
| lncRNA_063009 | Traes_7AL_0B358E4B4 | -0.992624 | 8.14E-05 |
| lncRNA_059323 | Traes_7AL_1784D5698 | -0.990644 | 0.0001309 |
| lncRNA_053766 | Traes_7AL_25850F96F | -0.994553 | 4.44E-05 |
| lncRNA_033754 | Traes_7AL_2585DE9FB | 0.9952141 | 3.43E-05 |
| TRAES3BF052700300CFD_g | Traes_7AL_2DB6CCCCF | -0.99826 | 4.54E-06 |
| lncRNA_018078 | Traes_7AL_2EA20BAA3 | 0.9906447 | 0.0001309 |
| lncRNA_051318 | Traes_7AL_2EA20BAA3 | 0.9931978 | 6.92E-05 |
| lncRNA_063127 | Traes_7AL_2EA20BAA3 | -0.991714 | 0.0001027 |
| lncRNA_047461 | Traes_7AL_38E309909 | 0.9967949 | 1.54E-05 |
| lncRNA_048668 | Traes_7AL_38E309909 | -0.990448 | 0.0001364 |
| lncRNA_027360 | Traes_7AL_45D72F80A | -0.99728 | 1.11E-05 |
| lncRNA_076127 | Traes_7AL_4878FA904 | 0.9953973 | 3.17E-05 |
| lncRNA_048668 | Traes_7AL_4EFFD9BDF | 0.9954773 | 3.06E-05 |
| lncRNA_051888 | Traes_7AL_4EFFD9BDF | -0.990065 | 0.0001476 |
| lncRNA_039420 | Traes_7AL_50C6E1770 | -0.99126 | 0.0001142 |
| lncRNA_044308 | Traes_7AL_50C6E1770 | -0.995437 | 3.12E-05 |
| lncRNA_046989 | Traes_7AL_53D418420 | 0.9974685 | 9.60E-06 |
| Traes_1BS_B58657408 | Traes_7AL_554BB7138 | -0.993316 | 6.69E-05 |
| lncRNA_027360 | Traes_7AL_570C9BD7F | 0.9901668 | 0.0001446 |
| lncRNA_059554 | Traes_7AL_570C9BD7F | 0.9944991 | 4.53E-05 |
| lncRNA_074658 | Traes_7AL_58009BF65 | -0.990133 | 0.0001456 |
| lncRNA_014504 | Traes_7AL_5C9D36038 | -0.990855 | 0.0001251 |
| lncRNA_034367 | Traes_7AL_5DF69C863 | 0.993591 | 6.15E-05 |
| lncRNA_017751 | Traes_7AL_61F7071D6 | -0.991929 | 9.75E-05 |
| lncRNA_025391 | Traes_7AL_61F7071D6 | -0.992696 | 7.98E-05 |
| lncRNA_015487 | Traes_7AL_65F481DB9 | -0.990533 | 0.000134 |
| lncRNA_056853 | Traes_7AL_6BB53B46B | 0.9960682 | 2.32E-05 |
| lncRNA_072440 | Traes_7AL_7E1C0EDEE | 0.9920391 | 9.48E-05 |
| lncRNA_051888 | Traes_7AL_83483EC2B | 0.9963112 | 2.04E-05 |
| lncRNA_013053 | Traes_7AL_8354C26D1 | 0.9985778 | 3.03E-06 |
| lncRNA_015322 | Traes_7AL_8354C26D1 | 0.990214 | 0.0001432 |
| lncRNA_058136 | Traes_7AL_8354C26D1 | 0.9907114 | 0.000129 |
| lncRNA_054317 | Traes_7AL_A1EA63AD2 | -0.992129 | 9.27E-05 |
| lncRNA_063009 | Traes_7AL_A1EA63AD2 | -0.994199 | 5.04E-05 |
| lncRNA_072440 | Traes_7AL_A29227860 | 0.990955 | 0.0001223 |
| Traes_1BS_B58657408 | Traes_7AL_A343EC774 | -0.995968 | 2.43E-05 |
| lncRNA_006011 | Traes_7AL_A343EC774 | 0.9934934 | 6.34E-05 |
| lncRNA_007700 | Traes_7AL_ACBB0F83B | 0.9910671 | 0.0001193 |
| lncRNA_014504 | Traes_7AL_ACBB0F83B | 0.993185 | 6.95E-05 |
| lncRNA_018078 | Traes_7AL_ACBB0F83B | 0.99366 | 6.02E-05 |
| lncRNA_043877 | Traes_7AL_ACBB0F83B | 0.992933 | 7.47E-05 |
| lncRNA_006011 | Traes_7AL_AF81980D6 | 0.9911196 | 0.0001179 |
| TRAES3BF052700300CFD_g | Traes_7AL_B23CC9F9F | -0.993411 | 6.50E-05 |
| lncRNA_006011 | Traes_7AL_B42D18A78 | 0.9975648 | 8.89E-06 |
| lncRNA_074837 | Traes_7AL_B42D18A78 | 0.9926842 | 8.01E-05 |
| lncRNA_029384 | Traes_7AL_B4329E36B | 0.9926627 | 8.06E-05 |
| lncRNA_001839 | Traes_7AL_B72D3D89F | -0.990127 | 0.0001457 |
| lncRNA_012242 | Traes_7AL_B7810831E | -0.994102 | 5.21E-05 |
| lncRNA_054317 | Traes_7AL_B7810831E | -0.991004 | 0.000121 |
| lncRNA_043148 | Traes_7AL_BB8CDE628 | 0.9962236 | 2.14E-05 |
| TRAES3BF052700300CFD_g | Traes_7AL_C4A1E88AB | -0.993269 | 6.78E-05 |
| lncRNA_008977 | Traes_7AL_C4A1E88AB | -0.99317 | 6.98E-05 |
| lncRNA_006859 | Traes_7AL_C82A28683 | 0.9964868 | 1.85E-05 |
| lncRNA_039420 | Traes_7AL_D1CAF31D3 | -0.99515 | 3.52E-05 |
| lncRNA_046989 | Traes_7AL_D1CAF31D3 | 0.9907429 | 0.0001281 |
| lncRNA_001557 | Traes_7AL_D1DB1B9EE | 0.9931008 | 7.12E-05 |
| lncRNA_072167 | Traes_7AL_D69B0DC0B | 0.9971903 | 1.18E-05 |
| Traes_1BS_B58657408 | Traes_7AL_D84A76A1E | -0.994958 | 3.81E-05 |
| lncRNA_020477 | Traes_7AL_D84A76A1E | 0.9927856 | 7.79E-05 |
| lncRNA_013231 | Traes_7AL_E9733B552 | 0.9953694 | 3.21E-05 |
| lncRNA_044556 | Traes_7AL_E9733B552 | 0.9903181 | 0.0001402 |
| lncRNA_000823 | Traes_7AL_EB1BE4011 | 0.9923305 | 8.80E-05 |
| lncRNA_024152 | Traes_7AL_F0E04F6F0 | -0.990388 | 0.0001382 |
| lncRNA_014504 | Traes_7AL_FC7B2C8E0 | -0.99917 | 1.03E-06 |
| lncRNA_019725 | Traes_7AL_FC7B2C8E0 | -0.991123 | 0.0001179 |
| lncRNA_048266 | Traes_7AL_FF8D82E06 | -0.990873 | 0.0001246 |
| lncRNA_007700 | Traes_7AS_04B7C0B3F | 0.9935887 | 6.15E-05 |
| lncRNA_018078 | Traes_7AS_04B7C0B3F | 0.9930875 | 7.15E-05 |
| lncRNA_048256 | Traes_7AS_04B7C0B3F | 0.9913487 | 0.0001119 |
| lncRNA_072440 | Traes_7AS_20DF4A591 | 0.9912701 | 0.000114 |
| lncRNA_083996 | Traes_7AS_26CAC27FF | 0.9944885 | 4.55E-05 |
| lncRNA_006361 | Traes_7AS_2BF62E1DE | 0.9917777 | 0.0001011 |
| lncRNA_012610 | Traes_7AS_2BF62E1DE | 0.9902117 | 0.0001432 |
| lncRNA_048668 | Traes_7AS_2EB9E44921 | 0.9962 | 2.16E-05 |
| lncRNA_015322 | Traes_7AS_30366404B | 0.9917734 | 0.0001012 |
| lncRNA_053766 | Traes_7AS_30366404B | 0.9900146 | 0.0001491 |
| lncRNA_014501 | Traes_7AS_3B388628C | 0.9910888 | 0.0001188 |
| lncRNA_020959 | Traes_7AS_3B388628C | 0.9962512 | 2.11E-05 |
| lncRNA_024807 | Traes_7AS_3B388628C | 0.9985052 | 3.35E-06 |
| lncRNA_044308 | Traes_7AS_3B388628C | 0.9933764 | 6.57E-05 |
| lncRNA_061738 | Traes_7AS_3B388628C | 0.9950507 | 3.67E-05 |
| TRAES3BF053100050CFD_g | Traes_7AS_4844916A8 | 0.9903274 | 0.0001399 |
| lncRNA_013190 | Traes_7AS_4844916A8 | 0.9937251 | 5.89E-05 |
| lncRNA_018078 | Traes_7AS_4844916A8 | 0.9950174 | 3.72E-05 |
| lncRNA_051318 | Traes_7AS_4844916A8 | 0.9979818 | 6.11E-06 |
| lncRNA_063127 | Traes_7AS_4844916A8 | -0.993623 | 6.09E-05 |
| lncRNA_022554 | Traes_7AS_4B8075360 | -0.994678 | 4.24E-05 |
| lncRNA_026968 | Traes_7AS_5321EDCCF | -0.991684 | 0.0001034 |
| lncRNA_078349 | Traes_7AS_5321EDCCF | -0.994965 | 3.80E-05 |
| lncRNA_051888 | Traes_7AS_53BE131BE | -0.992658 | 8.07E-05 |
| lncRNA_029013 | Traes_7AS_57E069383 | 0.9934387 | 6.44E-05 |
| lncRNA_063547 | Traes_7AS_57E069383 | 0.9971983 | 1.18E-05 |
| lncRNA_049300 | Traes_7AS_59F273AA2 | 0.9927414 | 7.88E-05 |
| lncRNA_014501 | Traes_7AS_5E7A92F55 | -0.994661 | 4.27E-05 |
| lncRNA_061738 | Traes_7AS_5E7A92F55 | -0.997436 | 9.85E-06 |
| lncRNA_048825 | Traes_7AS_6FBCCA079 | 0.9930219 | 7.29E-05 |
| lncRNA_049669 | Traes_7AS_6FBCCA079 | 0.991989 | 9.60E-05 |
| lncRNA_006361 | Traes_7AS_779E8A5D2 | 0.990364 | 0.0001388 |
| lncRNA_013190 | Traes_7AS_779E8A5D2 | 0.9925908 | 8.21E-05 |
| lncRNA_015487 | Traes_7AS_779E8A5D2 | 0.9911574 | 0.0001169 |
| lncRNA_051318 | Traes_7AS_779E8A5D2 | 0.9966413 | 1.69E-05 |
| lncRNA_074658 | Traes_7AS_799E72BE7 | -0.991289 | 0.0001135 |
| Traes_1BS_B58657408 | Traes_7AS_7E46CDBE9 | -0.997673 | 8.12E-06 |
| lncRNA_012242 | Traes_7AS_82047CC67 | 0.9928917 | 7.56E-05 |
| lncRNA_021433 | Traes_7AS_838CF89DA | -0.990802 | 0.0001265 |
| lncRNA_022554 | Traes_7AS_85178E0D7 | 0.9990441 | 1.37E-06 |
| lncRNA_048256 | Traes_7AS_85178E0D7 | 0.9907183 | 0.0001288 |
| lncRNA_047461 | Traes_7AS_8D8503F70 | -0.994696 | 4.21E-05 |
| TRAES3BF021800100CFD_g | Traes_7AS_8FA03045B1 | 0.9903399 | 0.0001395 |
| lncRNA_045725 | Traes_7AS_8FA03045B1 | 1 | 0 |
| lncRNA_051428 | Traes_7AS_8FA03045B1 | 1 | 0 |
| lncRNA_057614 | Traes_7AS_8FA03045B1 | 1 | 0 |
| lncRNA_065747 | Traes_7AS_8FA03045B1 | 1 | 0 |
| lncRNA_006011 | Traes_7AS_9F56E0BB1 | 0.9930906 | 7.14E-05 |
| lncRNA_074837 | Traes_7AS_9F56E0BB1 | 0.9962065 | 2.16E-05 |
| lncRNA_006361 | Traes_7AS_9FD687625 | -0.992274 | 8.93E-05 |
| lncRNA_007700 | Traes_7AS_A36C6DE1B | 0.9904097 | 0.0001375 |
| lncRNA_044581 | Traes_7AS_A3CC7900C | 0.9922887 | 8.90E-05 |
| lncRNA_047317 | Traes_7AS_A47BFC5DD | 0.9920363 | 9.49E-05 |
| lncRNA_009698 | Traes_7AS_A94491411 | 0.9945001 | 4.53E-05 |
| lncRNA_034367 | Traes_7AS_A94491411 | 0.9951244 | 3.56E-05 |
| TRAES3BF053100050CFD_g | Traes_7AS_AEE31C405 | 0.9963254 | 2.02E-05 |
| lncRNA_013190 | Traes_7AS_AEE31C405 | 0.9936969 | 5.95E-05 |
| lncRNA_018078 | Traes_7AS_AEE31C405 | 0.998184 | 4.94E-06 |
| lncRNA_043877 | Traes_7AS_AEE31C405 | 0.9914942 | 0.0001082 |
| lncRNA_051318 | Traes_7AS_AEE31C405 | 0.9954611 | 3.09E-05 |
| lncRNA_063127 | Traes_7AS_AEE31C405 | -0.995588 | 2.92E-05 |
| lncRNA_080648 | Traes_7AS_AEE31C405 | 0.9947053 | 4.20E-05 |
| lncRNA_066381 | Traes_7AS_B70DF258F | 0.9903181 | 0.0001402 |
| lncRNA_074658 | Traes_7AS_C8B1BB7DC | -0.999153 | 1.08E-06 |
| lncRNA_034367 | Traes_7AS_DA1089F6B | -0.991596 | 0.0001057 |
| lncRNA_048668 | Traes_7AS_E0F56CCBF1 | 0.9936599 | 6.02E-05 |
| lncRNA_051888 | Traes_7AS_E0F56CCBF1 | -0.991734 | 0.0001022 |
| lncRNA_008977 | Traes_7AS_E2D5BBB00 | -0.994768 | 4.10E-05 |
| lncRNA_014538 | Traes_7AS_E78E87CB8 | 0.9921561 | 9.20E-05 |
| lncRNA_000823 | Traes_7AS_E98EFC7A8 | 0.9901196 | 0.000146 |
| lncRNA_024812 | Traes_7AS_ED5778D91 | 0.9925224 | 8.37E-05 |
| lncRNA_074837 | Traes_7AS_F1E2FE61B | 0.9920166 | 9.53E-05 |
| lncRNA_015487 | Traes_7AS_F4286E9E7 | -0.992974 | 7.39E-05 |
| lncRNA_054317 | Traes_7AS_F4286E9E7 | -0.994866 | 3.95E-05 |
| lncRNA_032287 | Traes_7AS_F4E9E380A | 0.9947708 | 4.09E-05 |
| lncRNA_044556 | Traes_7AS_FC4D18A1D | 0.9966718 | 1.66E-05 |
| lncRNA_047461 | Traes_7AS_FFB7CAFC3 | 0.9969788 | 1.37E-05 |
| TRAES3BF053100050CFD_g | Traes_7BL_04D6F3E9E | -0.990396 | 0.0001379 |
| lncRNA_007700 | Traes_7BL_04D6F3E9E | -0.992215 | 9.07E-05 |
| lncRNA_013190 | Traes_7BL_04D6F3E9E | -0.991406 | 0.0001105 |
| lncRNA_018078 | Traes_7BL_04D6F3E9E | -0.998649 | 2.74E-06 |
| lncRNA_051318 | Traes_7BL_04D6F3E9E | -0.995034 | 3.69E-05 |
| lncRNA_063127 | Traes_7BL_04D6F3E9E | 0.9953345 | 3.26E-05 |
| TRAES3BF052700300CFD_g | Traes_7BL_0C2221400 | -0.996178 | 2.19E-05 |
| lncRNA_048668 | Traes_7BL_0D3422654 | 0.9956278 | 2.86E-05 |
| TRAES3BF053100050CFD_g | Traes_7BL_1140EF871 | 0.9920925 | 9.35E-05 |
| lncRNA_009698 | Traes_7BL_1140EF871 | 0.9930101 | 7.31E-05 |
| lncRNA_012610 | Traes_7BL_1140EF871 | 0.9910287 | 0.0001204 |
| lncRNA_013190 | Traes_7BL_1140EF871 | 0.9996861 | 1.48E-07 |
| lncRNA_018078 | Traes_7BL_1140EF871 | 0.9930824 | 7.16E-05 |
| lncRNA_048256 | Traes_7BL_1140EF871 | 0.9915094 | 0.0001078 |
| lncRNA_051318 | Traes_7BL_1140EF871 | 0.9927544 | 7.86E-05 |
| lncRNA_008500 | Traes_7BL_1AB45EC08 | 0.994928 | 3.85E-05 |
| lncRNA_074658 | Traes_7BL_1AB45EC08 | 0.9911023 | 0.0001184 |
| lncRNA_048266 | Traes_7BL_20078E949 | -0.992221 | 9.05E-05 |
| lncRNA_007700 | Traes_7BL_2090E6665 | 0.9900716 | 0.0001474 |
| lncRNA_020959 | Traes_7BL_2090E6665 | 0.9902418 | 0.0001424 |
| lncRNA_029013 | Traes_7BL_2090E6665 | 0.9952897 | 3.32E-05 |
| lncRNA_032287 | Traes_7BL_2225AB0B4 | -0.99465 | 4.29E-05 |
| lncRNA_029013 | Traes_7BL_2BA6E31FC | 0.9956223 | 2.87E-05 |
| lncRNA_063127 | Traes_7BL_2BA6E31FC | -0.990228 | 0.0001428 |
| TRAES3BF117100150CFD_g | Traes_7BL_306E3FC40 | 0.9937429 | 5.86E-05 |
| lncRNA_012610 | Traes_7BL_306E3FC40 | -0.995093 | 3.61E-05 |
| lncRNA_033754 | Traes_7BL_306E3FC40 | -0.991794 | 0.0001007 |
| lncRNA_039420 | Traes_7BL_311DDF828 | -0.997625 | 8.46E-06 |
| lncRNA_053891 | Traes_7BL_342CAC6CE | -0.993811 | 5.73E-05 |
| lncRNA_012242 | Traes_7BL_446890760 | 0.9928006 | 7.76E-05 |
| lncRNA_054317 | Traes_7BL_446890760 | 0.9938668 | 5.63E-05 |
| lncRNA_009698 | Traes_7BL_483EFD85F | 0.9960311 | 2.36E-05 |
| lncRNA_012610 | Traes_7BL_483EFD85F | 0.9943079 | 4.85E-05 |
| lncRNA_013190 | Traes_7BL_483EFD85F | 0.9960012 | 2.40E-05 |
| lncRNA_048256 | Traes_7BL_483EFD85F | 0.9941703 | 5.09E-05 |
| lncRNA_061738 | Traes_7BL_4F9263744 | -0.990536 | 0.0001339 |
| lncRNA_006361 | Traes_7BL_5A5A096A41 | -0.997957 | 6.25E-06 |
| lncRNA_015487 | Traes_7BL_5A5A096A41 | -0.99337 | 6.58E-05 |
| lncRNA_018111 | Traes_7BL_5A5A096A41 | -0.992238 | 9.01E-05 |
| lncRNA_014504 | Traes_7BL_625F55A12 | 0.9949501 | 3.82E-05 |
| lncRNA_019725 | Traes_7BL_625F55A12 | 0.9902412 | 0.0001424 |
| lncRNA_043877 | Traes_7BL_625F55A12 | 0.9928726 | 7.60E-05 |
| lncRNA_014504 | Traes_7BL_69588C86C | -0.996388 | 1.95E-05 |
| lncRNA_001839 | Traes_7BL_69A11C4AF | -0.990602 | 0.0001321 |
| lncRNA_020959 | Traes_7BL_6A345CEBA | 0.990944 | 0.0001226 |
| lncRNA_059554 | Traes_7BL_71FFD7DB2 | -0.994063 | 5.28E-05 |
| lncRNA_006011 | Traes_7BL_83400E481 | 0.9941494 | 5.12E-05 |
| lncRNA_006011 | Traes_7BL_85CD08BEA | 0.9939838 | 5.42E-05 |
| lncRNA_029384 | Traes_7BL_89235BE1B | 0.9948584 | 3.96E-05 |
| lncRNA_051551 | Traes_7BL_8F49CE9D6 | 0.9930781 | 7.17E-05 |
| lncRNA_072440 | Traes_7BL_A4C3C56C1 | 0.9945111 | 4.51E-05 |
| lncRNA_012242 | Traes_7BL_ABEAC99C8 | -0.991077 | 0.0001191 |
| lncRNA_022554 | Traes_7BL_ACCC05746 | -0.993997 | 5.39E-05 |
| lncRNA_020959 | Traes_7BL_AFB3A9477 | 0.9940549 | 5.29E-05 |
| lncRNA_024807 | Traes_7BL_AFB3A9477 | 0.9927284 | 7.91E-05 |
| lncRNA_020477 | Traes_7BL_B14ACE95C | 0.9922561 | 8.97E-05 |
| lncRNA_051551 | Traes_7BL_B47AEC3EA | 0.9931723 | 6.98E-05 |
| lncRNA_033754 | Traes_7BL_BA7E33E96 | 0.9947 | 4.21E-05 |
| lncRNA_064639 | Traes_7BL_BA7E33E96 | 0.9918125 | 0.0001003 |
| lncRNA_007700 | Traes_7BL_C86E000C5 | 0.9912779 | 0.0001138 |
| lncRNA_018078 | Traes_7BL_C86E000C5 | 0.9969757 | 1.37E-05 |
| lncRNA_051318 | Traes_7BL_C86E000C5 | 0.994328 | 4.82E-05 |
| lncRNA_063127 | Traes_7BL_C86E000C5 | -0.99537 | 3.21E-05 |
| lncRNA_074434 | Traes_7BL_CF330AE96 | 0.9928186 | 7.72E-05 |
| lncRNA_047461 | Traes_7BL_D1311836E | -0.990992 | 0.0001214 |
| TRAES3BF024700270CFD_g | Traes_7BL_D3A25B6C7 | 0.9900103 | 0.0001492 |
| lncRNA_009698 | Traes_7BL_F590C0638 | 0.9946297 | 4.32E-05 |
| lncRNA_012610 | Traes_7BL_F590C0638 | 0.9922853 | 8.90E-05 |
| lncRNA_013190 | Traes_7BL_F590C0638 | 0.9992274 | 8.95E-07 |
| lncRNA_018078 | Traes_7BL_F590C0638 | 0.9903869 | 0.0001382 |
| lncRNA_048256 | Traes_7BL_F590C0638 | 0.9961925 | 2.17E-05 |
| lncRNA_051318 | Traes_7BL_F590C0638 | 0.9905364 | 0.0001339 |
| TRAES3BF117100150CFD_g | Traes_7BL_FB18085F9 | -0.994663 | 4.26E-05 |
| lncRNA_034367 | Traes_7BL_FB18085F9 | 0.9924801 | 8.46E-05 |
| lncRNA_080683 | Traes_7BL_FB18085F9 | 0.9908737 | 0.0001246 |
| lncRNA_006270 | Traes_7BL_FDFA49DCF | 0.990022 | 0.0001488 |
| lncRNA_021433 | Traes_7BL_FDFA49DCF | 0.9943265 | 4.82E-05 |
| lncRNA_006085 | Traes_7BS_0720C54AF | -0.993951 | 5.48E-05 |
| lncRNA_012242 | Traes_7BS_0720C54AF | -0.990342 | 0.0001395 |
| lncRNA_032287 | Traes_7BS_0720C54AF | -0.991322 | 0.0001126 |
| lncRNA_033754 | Traes_7BS_0720C54AF | -0.992062 | 9.43E-05 |
| lncRNA_064639 | Traes_7BS_0720C54AF | -0.992823 | 7.71E-05 |
| lncRNA_072440 | Traes_7BS_1060BA69E | 0.9945415 | 4.46E-05 |
| lncRNA_022554 | Traes_7BS_1D9F44049 | 0.9917925 | 0.0001008 |
| lncRNA_048256 | Traes_7BS_1D9F44049 | 0.9929924 | 7.35E-05 |
| lncRNA_046989 | Traes_7BS_1E34E3CCC | 0.9900434 | 0.0001482 |
| lncRNA_044308 | Traes_7BS_273BAAA15 | 0.9916457 | 0.0001044 |
| TRAES3BF117100150CFD_g | Traes_7BS_2DA5DB033 | 0.9904485 | 0.0001364 |
| lncRNA_012242 | Traes_7BS_2DA5DB033 | -0.997643 | 8.32E-06 |
| lncRNA_054317 | Traes_7BS_2DA5DB033 | -0.991294 | 0.0001134 |
| lncRNA_047461 | Traes_7BS_2EA0B317B | -0.9922 | 9.10E-05 |
| lncRNA_048256 | Traes_7BS_2EA0B317B | -0.990723 | 0.0001287 |
| lncRNA_082364 | Traes_7BS_3FD78F8D5 | 0.9908626 | 0.0001249 |
| TRAES3BF060500060CFD_g | Traes_7BS_4306A478C | 0.9955754 | 2.93E-05 |
| TRAES3BF021800100CFD_g | Traes_7BS_455289375 | 0.9903399 | 0.0001395 |
| lncRNA_045725 | Traes_7BS_455289375 | 1 | 0 |
| lncRNA_051428 | Traes_7BS_455289375 | 1 | 0 |
| lncRNA_057614 | Traes_7BS_455289375 | 1 | 0 |
| lncRNA_065747 | Traes_7BS_455289375 | 1 | 0 |
| Traes_1BS_B58657408 | Traes_7BS_46E1123CE | -0.998055 | 5.67E-06 |
| lncRNA_068995 | Traes_7BS_49D2E9794 | 0.9912377 | 0.0001148 |
| lncRNA_020477 | Traes_7BS_4BC05D388 | 0.9929281 | 7.48E-05 |
| lncRNA_048668 | Traes_7BS_4BC05D388 | 0.9902304 | 0.0001427 |
| lncRNA_048256 | Traes_7BS_601206A5E | -0.994157 | 5.11E-05 |
| lncRNA_007700 | Traes_7BS_725BE98D0 | 0.9958373 | 2.60E-05 |
| lncRNA_018078 | Traes_7BS_725BE98D0 | 0.9940375 | 5.32E-05 |
| lncRNA_029013 | Traes_7BS_725BE98D0 | 0.9928521 | 7.65E-05 |
| lncRNA_043877 | Traes_7BS_725BE98D0 | 0.9901931 | 0.0001438 |
| lncRNA_063127 | Traes_7BS_725BE98D0 | -0.99247 | 8.48E-05 |
| lncRNA_080648 | Traes_7BS_725BE98D0 | 0.9910349 | 0.0001202 |
| Traes_1BS_B58657408 | Traes_7BS_7849253B3 | -0.993481 | 6.36E-05 |
| lncRNA_010675 | Traes_7BS_81AD0B25D | 0.9971719 | 1.20E-05 |
| lncRNA_068995 | Traes_7BS_8D9D397291 | 0.9903898 | 0.0001381 |
| lncRNA_081387 | Traes_7BS_8F1C8872E | 0.9920411 | 9.48E-05 |
| lncRNA_039420 | Traes_7BS_94EB3B3D6 | -0.995426 | 3.13E-05 |
| lncRNA_074837 | Traes_7BS_94EB3B3D6 | 0.9929276 | 7.49E-05 |
| lncRNA_057390 | Traes_7BS_A17E3A1E2 | -0.991149 | 0.0001172 |
| lncRNA_066381 | Traes_7BS_A2B219440 | 0.9922738 | 8.93E-05 |
| lncRNA_020477 | Traes_7BS_BA93A05F0 | 0.9963491 | 2.00E-05 |
| lncRNA_048668 | Traes_7BS_BA93A05F0 | 0.9959138 | 2.50E-05 |
| lncRNA_014501 | Traes_7BS_C0604DCF9 | -0.996008 | 2.39E-05 |
| lncRNA_039420 | Traes_7BS_C222B0121 | -0.996877 | 1.46E-05 |
| lncRNA_074837 | Traes_7BS_C222B0121 | 0.9928061 | 7.74E-05 |
| Traes_1BS_B58657408 | Traes_7BS_D1C00337F | -0.994061 | 5.28E-05 |
| Traes_1BS_B58657408 | Traes_7BS_D6A3AE648 | -0.990428 | 0.000137 |
| lncRNA_039420 | Traes_7BS_D6A3AE648 | -0.991227 | 0.0001151 |
| lncRNA_018111 | Traes_7BS_E11EC3E6E | 0.9910523 | 0.0001197 |
| lncRNA_047461 | Traes_7BS_E11EC3E6E | 0.992555 | 8.29E-05 |
| lncRNA_029384 | Traes_7BS_E5F156EF0 | 0.9918266 | 9.99E-05 |
| lncRNA_056853 | Traes_7BS_EC453C646 | -0.990821 | 0.000126 |
| lncRNA_014504 | Traes_7BS_FFDE56585 | -0.992575 | 8.25E-05 |
| lncRNA_024807 | Traes_7BS_FFDE56585 | -0.990646 | 0.0001308 |
| lncRNA_048266 | Traes_7DL_07D295BFC | -0.991694 | 0.0001032 |
| lncRNA_008977 | Traes_7DL_090D0E08E | 0.9912961 | 0.0001133 |
| lncRNA_047461 | Traes_7DL_090D0E08E | 0.991048 | 0.0001198 |
| TRAES3BF053100050CFD_g | Traes_7DL_0B4336C25 | -0.997798 | 7.27E-06 |
| lncRNA_080648 | Traes_7DL_0B4336C25 | -0.993411 | 6.50E-05 |
| lncRNA_048825 | Traes_7DL_0D01F15FF | 0.9900757 | 0.0001473 |
| lncRNA_081387 | Traes_7DL_0E95F6220 | -0.996794 | 1.54E-05 |
| Traes_1BS_B58657408 | Traes_7DL_15ACB06B8 | -0.990156 | 0.0001449 |
| lncRNA_013190 | Traes_7DL_175492122 | 0.9905224 | 0.0001343 |
| lncRNA_018078 | Traes_7DL_175492122 | 0.9930132 | 7.31E-05 |
| lncRNA_051318 | Traes_7DL_175492122 | 0.9992013 | 9.57E-07 |
| lncRNA_063127 | Traes_7DL_175492122 | -0.99327 | 6.78E-05 |
| lncRNA_020939 | Traes_7DL_1C864D382 | 0.9913927 | 0.0001108 |
| lncRNA_013190 | Traes_7DL_2A079DBC4 | -0.99247 | 8.48E-05 |
| lncRNA_022554 | Traes_7DL_2A079DBC4 | -0.995414 | 3.15E-05 |
| lncRNA_048256 | Traes_7DL_2A079DBC4 | -0.996173 | 2.19E-05 |
| lncRNA_006011 | Traes_7DL_2A6098BC2 | 0.9907939 | 0.0001267 |
| lncRNA_049461 | Traes_7DL_2AFCB81A4 | -0.996428 | 1.91E-05 |
| lncRNA_012105 | Traes_7DL_2D9B5BD66 | 0.9913547 | 0.0001118 |
| lncRNA_073681 | Traes_7DL_2D9B5BD66 | 0.9903515 | 0.0001392 |
| lncRNA_032287 | Traes_7DL_2DF9C185B | 0.9900467 | 0.0001481 |
| lncRNA_033754 | Traes_7DL_2DF9C185B | 0.9916856 | 0.0001034 |
| lncRNA_020959 | Traes_7DL_2EDF5CB11 | 0.9902535 | 0.000142 |
| lncRNA_024807 | Traes_7DL_2EDF5CB11 | 0.993672 | 5.99E-05 |
| lncRNA_044308 | Traes_7DL_2EDF5CB11 | 0.9952246 | 3.42E-05 |
| lncRNA_061738 | Traes_7DL_2EDF5CB11 | 0.9915708 | 0.0001063 |
| lncRNA_013053 | Traes_7DL_2F8330C38 | 0.9967444 | 1.59E-05 |
| lncRNA_032429 | Traes_7DL_3031D8DF9 | -0.990836 | 0.0001256 |
| lncRNA_063547 | Traes_7DL_310E46F15 | 0.9938949 | 5.58E-05 |
| lncRNA_029013 | Traes_7DL_374E11131 | -0.997239 | 1.14E-05 |
| lncRNA_063127 | Traes_7DL_374E11131 | 0.9959606 | 2.44E-05 |
| Traes_1BS_B58657408 | Traes_7DL_3E770E98B | -0.99261 | 8.17E-05 |
| lncRNA_063127 | Traes_7DL_3EECFACC5 | 0.9907785 | 0.0001272 |
| lncRNA_068995 | Traes_7DL_40E0AD332 | 0.9930495 | 7.23E-05 |
| lncRNA_000928 | Traes_7DL_446C318E9 | 0.9973822 | 1.03E-05 |
| lncRNA_000833 | Traes_7DL_46A4DB1C5 | 0.9905093 | 0.0001347 |
| lncRNA_020959 | Traes_7DL_4A9E55BEB | -0.994827 | 4.01E-05 |
| lncRNA_024807 | Traes_7DL_4A9E55BEB | -0.994494 | 4.54E-05 |
| lncRNA_044308 | Traes_7DL_4A9E55BEB | -0.996717 | 1.62E-05 |
| lncRNA_048825 | Traes_7DL_4C9B51BF6 | 0.9908258 | 0.0001259 |
| lncRNA_017776 | Traes_7DL_540E96109 | -0.995009 | 3.73E-05 |
| lncRNA_039420 | Traes_7DL_57CEECAF6 | -0.995599 | 2.90E-05 |
| lncRNA_074837 | Traes_7DL_57CEECAF6 | 0.9952271 | 3.41E-05 |
| lncRNA_032287 | Traes_7DL_5CA7F86A2 | -0.99041 | 0.0001375 |
| lncRNA_039420 | Traes_7DL_6233C6F03 | -0.997496 | 9.39E-06 |
| lncRNA_074837 | Traes_7DL_6233C6F03 | 0.9914869 | 0.0001084 |
| lncRNA_047461 | Traes_7DL_63118F92C | -0.991007 | 0.0001209 |
| lncRNA_048256 | Traes_7DL_63118F92C | -0.993322 | 6.67E-05 |
| lncRNA_063009 | Traes_7DL_63118F92C | -0.992844 | 7.66E-05 |
| TRAES3BF117100150CFD_g | Traes_7DL_6511AD958 | 0.9944048 | 4.69E-05 |
| lncRNA_033754 | Traes_7DL_6511AD958 | -0.993179 | 6.96E-05 |
| lncRNA_020477 | Traes_7DL_6599172BA | 0.9991393 | 1.11E-06 |
| lncRNA_018111 | Traes_7DL_71ED1C153 | 0.9939666 | 5.45E-05 |
| lncRNA_047461 | Traes_7DL_71ED1C153 | 0.9934341 | 6.45E-05 |
| lncRNA_066190 | Traes_7DL_772961158 | 0.9905759 | 0.0001328 |
| lncRNA_006859 | Traes_7DL_7ACE53E78 | 0.990163 | 0.0001447 |
| lncRNA_048256 | Traes_7DL_88DE10A78 | -0.994286 | 4.89E-05 |
| lncRNA_015487 | Traes_7DL_98EC3AE06 | -0.99056 | 0.0001333 |
| lncRNA_020959 | Traes_7DL_A1D314F33 | 0.9962222 | 2.14E-05 |
| lncRNA_024807 | Traes_7DL_A1D314F33 | 0.9959958 | 2.40E-05 |
| lncRNA_061738 | Traes_7DL_A1D314F33 | 0.9910774 | 0.0001191 |
| lncRNA_015487 | Traes_7DL_A321818FB | -0.991185 | 0.0001162 |
| lncRNA_051318 | Traes_7DL_A321818FB | -0.990383 | 0.0001383 |
| lncRNA_000928 | Traes_7DL_A558E74DF | -0.99123 | 0.000115 |
| lncRNA_009698 | Traes_7DL_A558E74DF | 0.9956532 | 2.83E-05 |
| lncRNA_026943 | Traes_7DL_A5AB2A3C5 | 0.9918763 | 9.87E-05 |
| lncRNA_013231 | Traes_7DL_A9EF00572 | 0.9946444 | 4.29E-05 |
| lncRNA_026968 | Traes_7DL_A9EF00572 | 0.9961666 | 2.20E-05 |
| lncRNA_078349 | Traes_7DL_A9EF00572 | 0.9927985 | 7.76E-05 |
| TRAES3BF052700300CFD_g | Traes_7DL_AAD0A273B1 | 0.9929147 | 7.51E-05 |
| lncRNA_061738 | Traes_7DL_AAD0A273B1 | 0.9920536 | 9.45E-05 |
| lncRNA_029088 | Traes_7DL_AF8F31A6B1 | 0.9956407 | 2.85E-05 |
| lncRNA_024812 | Traes_7DL_B80093097 | 0.9920039 | 9.57E-05 |
| lncRNA_019748 | Traes_7DL_B8D42442D | -0.99422 | 5.00E-05 |
| lncRNA_008977 | Traes_7DL_C020A321F | -0.992199 | 9.11E-05 |
| lncRNA_014504 | Traes_7DL_C1A7BA0C7 | -0.990391 | 0.0001381 |
| TRAES3BF177500010CFD_g | Traes_7DL_C5D5AF434 | 0.9945093 | 4.51E-05 |
| lncRNA_039943 | Traes_7DL_D36421981 | 0.993351 | 6.62E-05 |
| lncRNA_051888 | Traes_7DL_D36421981 | -0.990544 | 0.0001337 |
| lncRNA_053766 | Traes_7DL_D36421981 | 0.9985545 | 3.13E-06 |
| lncRNA_014501 | Traes_7DL_D54D54535 | -0.990875 | 0.0001245 |
| lncRNA_047461 | Traes_7DL_D54D54535 | -0.995324 | 3.28E-05 |
| lncRNA_008977 | Traes_7DL_D810BBA8B | -0.99352 | 6.29E-05 |
| lncRNA_047461 | Traes_7DL_D810BBA8B | -0.990578 | 0.0001327 |
| lncRNA_018111 | Traes_7DL_DB32ADFAE | -0.990319 | 0.0001401 |
| lncRNA_039943 | Traes_7DL_DB32ADFAE | 0.9904333 | 0.0001368 |
| lncRNA_015322 | Traes_7DL_DB6471BF0 | 0.9921069 | 9.32E-05 |
| lncRNA_051888 | Traes_7DL_DB6471BF0 | -0.990355 | 0.0001391 |
| lncRNA_014504 | Traes_7DL_E67BFDBAF | 0.9904334 | 0.0001368 |
| lncRNA_076127 | Traes_7DL_E71001BF31 | 0.9946332 | 4.31E-05 |
| lncRNA_029384 | Traes_7DL_E733CFD4C | 0.9911744 | 0.0001165 |
| TRAES3BF060500060CFD_g | Traes_7DL_F50060F73 | 0.992215 | 9.07E-05 |
| Traes_1BS_B58657408 | Traes_7DL_F842DB269 | -0.997181 | 1.19E-05 |
| lncRNA_009698 | Traes_7DL_F9F606152 | 0.9942566 | 4.94E-05 |
| lncRNA_012610 | Traes_7DL_F9F606152 | 0.9944662 | 4.59E-05 |
| lncRNA_013190 | Traes_7DL_F9F606152 | 0.9995731 | 2.73E-07 |
| lncRNA_018078 | Traes_7DL_F9F606152 | 0.9909418 | 0.0001227 |
| lncRNA_048256 | Traes_7DL_F9F606152 | 0.9934764 | 6.37E-05 |
| lncRNA_051318 | Traes_7DL_F9F606152 | 0.9935853 | 6.16E-05 |
| lncRNA_006859 | Traes_7DS_06734A236 | 0.9930029 | 7.33E-05 |
| lncRNA_012608 | Traes_7DS_06734A236 | 0.9900478 | 0.0001481 |
| lncRNA_014538 | Traes_7DS_06734A236 | 0.9938616 | 5.64E-05 |
| TRAES3BF052700300CFD_g | Traes_7DS_0EDCADCB9 | -0.994021 | 5.35E-05 |
| lncRNA_013231 | Traes_7DS_1690B1EC2 | 0.990321 | 0.0001401 |
| lncRNA_044556 | Traes_7DS_1690B1EC2 | 0.9905724 | 0.0001329 |
| lncRNA_014501 | Traes_7DS_1D44614AE | 0.9952122 | 3.43E-05 |
| lncRNA_024807 | Traes_7DS_1D44614AE | 0.9916776 | 0.0001036 |
| lncRNA_061738 | Traes_7DS_1D44614AE | 0.9934753 | 6.37E-05 |
| lncRNA_053655 | Traes_7DS_1DAA0FD56 | 0.9930593 | 7.21E-05 |
| TRAES3BF021800100CFD_g | Traes_7DS_208E8C4FA | 0.9944355 | 4.64E-05 |
| lncRNA_039420 | Traes_7DS_2565207CB | 0.9972999 | 1.09E-05 |
| lncRNA_021433 | Traes_7DS_308B45EF2 | 0.9921951 | 9.11E-05 |
| lncRNA_044556 | Traes_7DS_308B45EF2 | 0.9976708 | 8.13E-06 |
| lncRNA_044556 | Traes_7DS_326DC5875 | -0.990289 | 0.000141 |
| lncRNA_039943 | Traes_7DS_3CC764005 | 0.9912055 | 0.0001157 |
| lncRNA_051888 | Traes_7DS_3CC764005 | -0.993201 | 6.92E-05 |
| lncRNA_053766 | Traes_7DS_3CC764005 | 0.9969506 | 1.39E-05 |
| lncRNA_020477 | Traes_7DS_3F6DCEAA8 | -0.994887 | 3.91E-05 |
| lncRNA_013190 | Traes_7DS_44D3A516A | -0.99805 | 5.70E-06 |
| lncRNA_018078 | Traes_7DS_44D3A516A | -0.990229 | 0.0001427 |
| lncRNA_022554 | Traes_7DS_44D3A516A | -0.99003 | 0.0001486 |
| lncRNA_048256 | Traes_7DS_44D3A516A | -0.997109 | 1.25E-05 |
| TRAES3BF021800100CFD_g | Traes_7DS_4D232A410 | 0.9957938 | 2.65E-05 |
| lncRNA_066381 | Traes_7DS_4D753C0D8 | 0.9948151 | 4.03E-05 |
| lncRNA_056853 | Traes_7DS_57360433C | 0.9936087 | 6.11E-05 |
| lncRNA_022554 | Traes_7DS_5D913121E | -0.993653 | 6.03E-05 |
| lncRNA_012608 | Traes_7DS_6233E5A7D | 0.9911983 | 0.0001159 |
| TRAES3BF053100050CFD_g | Traes_7DS_630675687 | 0.9901349 | 0.0001455 |
| lncRNA_009698 | Traes_7DS_630675687 | 0.9914907 | 0.0001083 |
| lncRNA_013190 | Traes_7DS_630675687 | 0.9971076 | 1.25E-05 |
| lncRNA_018078 | Traes_7DS_630675687 | 0.9968279 | 1.51E-05 |
| lncRNA_019725 | Traes_7DS_630675687 | 0.990706 | 0.0001292 |
| lncRNA_043877 | Traes_7DS_630675687 | 0.990883 | 0.0001243 |
| lncRNA_048256 | Traes_7DS_630675687 | 0.9911489 | 0.0001172 |
| lncRNA_051318 | Traes_7DS_630675687 | 0.9956318 | 2.86E-05 |
| lncRNA_014504 | Traes_7DS_86E0BBC6D | 0.9924867 | 8.45E-05 |
| lncRNA_048668 | Traes_7DS_8A36B9C98 | 0.9924702 | 8.48E-05 |
| lncRNA_051888 | Traes_7DS_8A36B9C98 | -0.991942 | 9.71E-05 |
| lncRNA_046989 | Traes_7DS_8A552FC5D | 0.9973861 | 1.02E-05 |
| lncRNA_014538 | Traes_7DS_9764DBD72 | 0.9929264 | 7.49E-05 |
| lncRNA_008977 | Traes_7DS_98F9E4E55 | -0.997264 | 1.12E-05 |
| lncRNA_014504 | Traes_7DS_9EEB5C26C | 0.9986572 | 2.70E-06 |
| lncRNA_057390 | Traes_7DS_A7468F6DA | -0.990583 | 0.0001326 |
| lncRNA_009698 | Traes_7DS_AD276B1B8 | 0.9909931 | 0.0001213 |
| lncRNA_032287 | Traes_7DS_AD276B1B8 | 0.9953324 | 3.26E-05 |
| lncRNA_006085 | Traes_7DS_AFD45A1BD | 0.9905824 | 0.0001326 |
| lncRNA_032287 | Traes_7DS_AFD45A1BD | 0.9910633 | 0.0001194 |
| lncRNA_006361 | Traes_7DS_B57694259 | -0.996968 | 1.38E-05 |
| lncRNA_015487 | Traes_7DS_B57694259 | -0.992598 | 8.20E-05 |
| lncRNA_026968 | Traes_7DS_BAA539E8E | 0.9919442 | 9.71E-05 |
| Traes_1BS_B58657408 | Traes_7DS_BE9F3B065 | -0.99124 | 0.0001148 |
| lncRNA_013190 | Traes_7DS_C80D1B7F1 | 0.9906805 | 0.0001299 |
| lncRNA_022554 | Traes_7DS_C80D1B7F1 | 0.9912227 | 0.0001152 |
| lncRNA_048256 | Traes_7DS_C80D1B7F1 | 0.9959336 | 2.48E-05 |
| lncRNA_032287 | Traes_7DS_CABE66833 | -0.996927 | 1.41E-05 |
| lncRNA_001557 | Traes_7DS_CCC202043 | -0.992134 | 9.26E-05 |
| lncRNA_053766 | Traes_7DS_D0D8AA00F | 0.9959784 | 2.42E-05 |
| lncRNA_074658 | Traes_7DS_D2D58778F | 0.9907482 | 0.000128 |
| lncRNA_039420 | Traes_7DS_D42371FA3 | -0.993378 | 6.56E-05 |
| lncRNA_046989 | Traes_7DS_D42371FA3 | 0.9915239 | 0.0001075 |
| lncRNA_074837 | Traes_7DS_D42371FA3 | 0.991662 | 0.000104 |
| TRAES3BF117100150CFD_g | Traes_7DS_D9A6063F0 | -0.990505 | 0.0001348 |
| lncRNA_012242 | Traes_7DS_D9A6063F0 | 0.9956165 | 2.88E-05 |
| lncRNA_054317 | Traes_7DS_D9A6063F0 | 0.9924898 | 8.44E-05 |
| lncRNA_056853 | Traes_7DS_ED407C2E7 | -0.994951 | 3.82E-05 |
| lncRNA_001839 | Traes_7DS_EEE208470 | -0.995487 | 3.05E-05 |
| lncRNA_013231 | Traes_7DS_F770E4011 | 0.9943826 | 4.72E-05 |
| lncRNA_082364 | Traes_7DS_F770E4011 | 0.9904736 | 0.0001357 |
| lncRNA_039420 | Traes_7DS_FDFC9633D | -0.992583 | 8.23E-05 |
| lncRNA_008500 | Traes_7DS_FF911FA4A | -0.993232 | 6.85E-05 |

**Table S6.** The correlation of miRNA-lncRNA co-expression

| Source | Target | Correlation | P.value |
| --- | --- | --- | --- |
| tae-m3821-3p | Traes_1BS_B58657408 | -0.99487453 | 3.93E-05 |
| oha-miR-199c-3p_R2-22L22 | lncRNA_006011 | 0.992541416 | 8.32E-05 |
| hsa-miR-6882-5p_R15-1L22 | lncRNA_006085 | 0.993174433 | 6.97E-05 |
| pma-miR-4543_R8-22L22 | lncRNA_006085 | 0.993174433 | 6.97E-05 |
| tae-m0966-5p | lncRNA_006085 | 0.993174433 | 6.97E-05 |
| tae-m1217-5p | lncRNA_006085 | 0.993174433 | 6.97E-05 |
| tae-m1682-5p | lncRNA_006085 | 0.993174433 | 6.97E-05 |
| gra-miR7505b_R6-20L21 | lncRNA_009698 | 0.99389066 | 5.59E-05 |
| mml-miR-892c-3p_R1-17L22 | lncRNA_009698 | 0.99389066 | 5.59E-05 |
| tae-m0403-5p | lncRNA_009698 | 0.99389066 | 5.59E-05 |
| tae-m2665-5p | lncRNA_009698 | 0.99389066 | 5.59E-05 |
| tae-m2876-5p | lncRNA_009698 | 0.99389066 | 5.59E-05 |
| tae-m4081-5p | lncRNA_009698 | 0.99389066 | 5.59E-05 |
| tae-m0298-5p | lncRNA_010675 | -0.99261547 | 8.16E-05 |
| gra-miR7505b_R6-20L21 | lncRNA_012610 | 0.996602166 | 1.73E-05 |
| mml-miR-892c-3p_R1-17L22 | lncRNA_012610 | 0.996602166 | 1.73E-05 |
| tae-m0403-5p | lncRNA_012610 | 0.996602166 | 1.73E-05 |
| tae-m2665-5p | lncRNA_012610 | 0.996602166 | 1.73E-05 |
| tae-m2876-5p | lncRNA_012610 | 0.996602166 | 1.73E-05 |
| tae-m4081-5p | lncRNA_012610 | 0.996602166 | 1.73E-05 |
| ata-miR172b-5p_R1-20L21 | lncRNA_013053 | 0.993613253 | 6.11E-05 |
| bmo-miR-3302_R19-5L23 | lncRNA_013053 | 0.993613253 | 6.11E-05 |
| tae-m0631-5p | lncRNA_013053 | 0.993613253 | 6.11E-05 |
| gra-miR7505b_R6-20L21 | lncRNA_013190 | 0.99632559 | 2.02E-05 |
| mml-miR-892c-3p_R1-17L22 | lncRNA_013190 | 0.99632559 | 2.02E-05 |
| tae-m0403-5p | lncRNA_013190 | 0.99632559 | 2.02E-05 |
| tae-m2665-5p | lncRNA_013190 | 0.99632559 | 2.02E-05 |
| tae-m2876-5p | lncRNA_013190 | 0.99632559 | 2.02E-05 |
| tae-m4081-5p | lncRNA_013190 | 0.99632559 | 2.02E-05 |
| smo-miR159_R2-21L21 | lncRNA_013231 | 0.991080314 | 0.000118986 |
| tae-m1832-5p | lncRNA_013231 | 0.991080314 | 0.000118986 |
| tae-m2038-5p | lncRNA_013231 | 0.991080314 | 0.000118986 |
| tae-m3208-5p | lncRNA_013231 | 0.991080314 | 0.000118986 |
| ata-miR167e-5p_R1-21L21 | lncRNA_014501 | 0.99787821 | 6.75E-06 |
| ssp-miR444b.2_R15-1L21 | lncRNA_014501 | -0.99422098 | 5.00E-05 |
| tae-m0298-5p | lncRNA_014501 | 0.990332499 | 0.000139739 |
| tae-m3157-5p | lncRNA_014501 | 0.99787821 | 6.75E-06 |
| tae-m2905-5p | lncRNA_015322 | -0.99040337 | 0.000137701 |
| tae-m3294-3p | lncRNA_015487 | -0.99410661 | 5.20E-05 |
| tae-m0561-5p | lncRNA_016209 | 0.998717599 | 2.47E-06 |
| tae-m1456-5p | lncRNA_017751 | -0.99240628 | 8.63E-05 |
| gga-miR-7475-5p_R20-6L20 | lncRNA_022554 | -0.99385535 | 5.65E-05 |
| bbe-miR-4857-5p_R3-17L23 | lncRNA_024812 | 0.990508937 | 0.000134693 |
| hsa-miR-3677-5p_R17-3L22 | lncRNA_024812 | 0.990508937 | 0.000134693 |
| ppy-miR-4451_R1-16L18 | lncRNA_026968 | 0.991654073 | 0.000104191 |
| tae-m4432-5p | lncRNA_029384 | 0.995879337 | 2.54E-05 |
| bdi-miR390a-3p | lncRNA_032048 | 0.998387403 | 3.90E-06 |
| tae-m2840-5p | lncRNA_033754 | 0.993589162 | 6.15E-05 |
| mtr-miR7701-5p_R18-4L21 | lncRNA_039325 | 0.996371878 | 1.97E-05 |
| bta-miR-2429_R19-5L20 | lncRNA_039420 | -0.99570649 | 2.76E-05 |
| nta-miR156f | lncRNA_039420 | -0.99570649 | 2.76E-05 |
| tae-m4030-5p | lncRNA_039420 | -0.99570649 | 2.76E-05 |
| tae-m4349-5p | lncRNA_039420 | -0.99570649 | 2.76E-05 |
| tae-miR9659-3p | lncRNA_047461 | 0.994131176 | 5.16E-05 |
| gra-miR7505b_R6-20L21 | lncRNA_048256 | 0.992536657 | 8.33E-05 |
| mml-miR-892c-3p_R1-17L22 | lncRNA_048256 | 0.992536657 | 8.33E-05 |
| tae-m0403-5p | lncRNA_048256 | 0.992536657 | 8.33E-05 |
| tae-m2665-5p | lncRNA_048256 | 0.992536657 | 8.33E-05 |
| tae-m2876-5p | lncRNA_048256 | 0.992536657 | 8.33E-05 |
| tae-m4081-5p | lncRNA_048256 | 0.992536657 | 8.33E-05 |
| tch-miR-378a-3p | lncRNA_048256 | -0.99576346 | 2.69E-05 |
| tae-m4368-5p | lncRNA_048320 | 0.998240123 | 4.64E-06 |
| ata-miR9772b-3p_R1-20L21 | lncRNA_048368 | -0.99250935 | 8.40E-05 |
| tae-m0982-5p | lncRNA_048668 | 0.995049042 | 3.67E-05 |
| tae-m3294-3p | lncRNA_051318 | -0.99090879 | 0.0001236 |
| ata-miR172b-5p_R1-20L21 | lncRNA_051888 | -0.99398752 | 5.41E-05 |
| bmo-miR-3302_R19-5L23 | lncRNA_051888 | -0.99398752 | 5.41E-05 |
| tae-m0631-5p | lncRNA_051888 | -0.99398752 | 5.41E-05 |
| tae-m1918-5p | lncRNA_056853 | 0.99696955 | 1.38E-05 |
| tae-m2015-5p | lncRNA_056853 | 0.99696955 | 1.38E-05 |
| tae-m3263-5p | lncRNA_056853 | 0.99696955 | 1.38E-05 |
| tae-m4217-3p | lncRNA_056853 | 0.99696955 | 1.38E-05 |
| ata-miR167e-5p_R1-21L21 | lncRNA_061738 | 0.995582409 | 2.92E-05 |
| ssp-miR444b.2_R15-1L21 | lncRNA_061738 | -0.99419909 | 5.04E-05 |
| tae-m3157-5p | lncRNA_061738 | 0.995582409 | 2.92E-05 |
| tae-m2333-5p | lncRNA_068995 | 0.992600758 | 8.19E-05 |
| tae-m3808-5p | lncRNA_068995 | 0.992600758 | 8.19E-05 |
| tae-miR396-5p_R1-21L21 | lncRNA_068995 | -0.9916197 | 0.00010505 |
| tae-m0561-5p | lncRNA_073681 | 0.991991033 | 9.60E-05 |
| tae-m0561-5p | lncRNA_074165 | 0.997621013 | 8.48E-06 |
| smo-miR159_R2-21L21 | lncRNA_074658 | 0.99033945 | 0.000139539 |
| tae-m1832-5p | lncRNA_074658 | 0.99033945 | 0.000139539 |
| tae-m2038-5p | lncRNA_074658 | 0.99033945 | 0.000139539 |
| tae-m3208-5p | lncRNA_074658 | 0.99033945 | 0.000139539 |
| tae-m2660-5p | lncRNA_080683 | 0.996488437 | 1.85E-05 |

**Table S7.** The correlation of miRNA-mRNA co-expression

| Source | Target | Correlation | P.value |
| --- | --- | --- | --- |
| ssp-miR444b.2_R15-1L21 | Traes_4BL_396369E0B | -0.999944867 | 4.56E-09 |
| tae-m3294-3p | Traes_2DS_C53EC62E8 | -0.999697495 | 1.37E-07 |
| tae-m2905-5p | Traes_1DS_65C1FDCD8 | -0.999670298 | 1.63E-07 |
| bdi-miR159b-3p.1_R1-21L21 | Traes_7BS_197D44BFD | -0.999593183 | 2.48E-07 |
| ata-miR167e-5p_R1-21L21 | Traes_6AS_0A0B33CEF | -0.999574914 | 2.71E-07 |
| tae-m3157-5p | Traes_6AS_0A0B33CEF | -0.999574914 | 2.71E-07 |
| tae-m1602-5p | Traes_7BS_D9BC83281 | -0.999553709 | 2.99E-07 |
| efu-miR-9189e_R24-4L24_8A-C | Traes_2AS_E77AB4BD1 | -0.999362085 | 6.10E-07 |
| gra-miR7505b_R6-20L21 | Traes_2DS_C6B631387 | -0.999334267 | 6.65E-07 |
| mml-miR-892c-3p_R1-17L22 | Traes_2DS_C6B631387 | -0.999334267 | 6.65E-07 |
| tae-m0403-5p | Traes_2DS_C6B631387 | -0.999334267 | 6.65E-07 |
| tae-m2665-5p | Traes_2DS_C6B631387 | -0.999334267 | 6.65E-07 |
| tae-m2876-5p | Traes_2DS_C6B631387 | -0.999334267 | 6.65E-07 |
| tae-m4081-5p | Traes_2DS_C6B631387 | -0.999334267 | 6.65E-07 |
| tae-m3294-3p | TRAES3BF046300070CFD_g | -0.999182333 | 1.00E-06 |
| tae-m0982-5p | Traes_4DL_A7C1B0A43 | -0.99893761 | 1.69E-06 |
| gga-miR-7475-5p_R20-6L20 | Traes_4DL_8D4180F54 | -0.998925924 | 1.73E-06 |
| tae-m3949-5p | Traes_7AL_B80AB336B | -0.998818493 | 2.09E-06 |
| tae-miR396-5p_R1-21L21 | Traes_2DL_1C46C0BB9 | -0.998755544 | 2.32E-06 |
| hsa-miR-6882-5p_R15-1L22 | Traes_2BS_C8FB3060D | -0.998747799 | 2.35E-06 |
| pma-miR-4543_R8-22L22 | Traes_2BS_C8FB3060D | -0.998747799 | 2.35E-06 |
| tae-m0966-5p | Traes_2BS_C8FB3060D | -0.998747799 | 2.35E-06 |
| tae-m1217-5p | Traes_2BS_C8FB3060D | -0.998747799 | 2.35E-06 |
| tae-m1682-5p | Traes_2BS_C8FB3060D | -0.998747799 | 2.35E-06 |
| gga-miR-7475-5p_R20-6L20 | Traes_1DL_83E665871 | -0.998745675 | 2.36E-06 |
| tae-m2905-5p | Traes_3DL_FAF514819 | -0.998727314 | 2.43E-06 |
| tch-miR-378a-3p | TRAES3BF059200050CFD_g | -0.998719305 | 2.46E-06 |
| tae-m1918-5p | Traes_6DS_9D2014869 | -0.998656081 | 2.71E-06 |
| tae-m2015-5p | Traes_6DS_9D2014869 | -0.998656081 | 2.71E-06 |
| tae-m3263-5p | Traes_6DS_9D2014869 | -0.998656081 | 2.71E-06 |
| tae-m4217-3p | Traes_6DS_9D2014869 | -0.998656081 | 2.71E-06 |
| tae-m3738-5p | Traes_7DS_991860897 | -0.998596957 | 2.95E-06 |
| gma-miR6300_R3-18L18 | Traes_6AL_D50E09BFC | -0.998586633 | 2.99E-06 |
| bmo-miR-3208_R4-19L22 | Traes_6AS_F9E75ED21 | -0.998503622 | 3.36E-06 |
| tae-m1452-5p | Traes_6AS_F9E75ED21 | -0.998503622 | 3.36E-06 |
| ssp-miR444b.2_R15-1L21 | Traes_5DS_7722ED6BA | -0.998436619 | 3.66E-06 |
| gga-miR-7475-5p_R20-6L20 | Traes_6DL_BEDFBC80F | -0.998394048 | 3.87E-06 |
| tae-m2840-5p | Traes_4DL_2527CA8BF | -0.998286812 | 4.40E-06 |
| tae-miR9659-3p | Traes_7AL_21A57AB64 | -0.998227193 | 4.71E-06 |
| tae-miR9659-3p | Traes_2AS_3F25BD910 | -0.998215643 | 4.77E-06 |
| tae-m2905-5p | Traes_7AL_0AA2FF9F0 | -0.998215371 | 4.77E-06 |
| tae-m1498-5p | TRAES3BF037500020CFD_g | -0.998197292 | 4.87E-06 |
| tae-m4236-5p | TRAES3BF037500020CFD_g | -0.998197292 | 4.87E-06 |
| ata-miR167d-5p_R1-22L22 | Traes_2DS_4B56AF2D6 | -0.998185687 | 4.93E-06 |
| tae-m3821-3p | Traes_3AL_9DF250DD8 | -0.998165859 | 5.04E-06 |
| hsa-miR-6882-5p_R15-1L22 | Traes_1AL_101B391E1 | -0.998091477 | 5.46E-06 |
| pma-miR-4543_R8-22L22 | Traes_1AL_101B391E1 | -0.998091477 | 5.46E-06 |
| tae-m0966-5p | Traes_1AL_101B391E1 | -0.998091477 | 5.46E-06 |
| tae-m1217-5p | Traes_1AL_101B391E1 | -0.998091477 | 5.46E-06 |
| tae-m1682-5p | Traes_1AL_101B391E1 | -0.998091477 | 5.46E-06 |
| tae-m1918-5p | Traes_2AL_2655769B3 | -0.998069458 | 5.59E-06 |
| tae-m2015-5p | Traes_2AL_2655769B3 | -0.998069458 | 5.59E-06 |
| tae-m3263-5p | Traes_2AL_2655769B3 | -0.998069458 | 5.59E-06 |
| tae-m4217-3p | Traes_2AL_2655769B3 | -0.998069458 | 5.59E-06 |
| stu-miR6027_R6-20L22 | Traes_4BS_B4E4E0A9C | -0.998059187 | 5.65E-06 |
| tch-miR-378a-3p | Traes_4DS_EC9D5061D | -0.997996737 | 6.02E-06 |
| tch-miR-378a-3p | Traes_6DL_F1E787360 | -0.997950789 | 6.29E-06 |
| ata-miR167e-5p_R1-21L21 | Traes_4DL_59311B671 | -0.997925763 | 6.45E-06 |
| tae-m3157-5p | Traes_4DL_59311B671 | -0.997925763 | 6.45E-06 |
| tae-m0298-5p | Traes_5AL_5640EAF9E | -0.997902271 | 6.60E-06 |
| gga-miR-7475-5p_R20-6L20 | Traes_7DS_C80D1B7F1 | -0.997831097 | 7.05E-06 |
| bdi-miR529-3p_R20-6L21 | Traes_2BL_EBDF3725F | -0.997791828 | 7.31E-06 |
| tch-miR-378a-3p | Traes_4DS_0B056789B | -0.997698663 | 7.94E-06 |
| bdi-miR159b-3p.1_R1-21L21 | Traes_1AS_1D65FC1BA | -0.997697478 | 7.95E-06 |
| gga-miR-7475-5p_R20-6L20 | Traes_1DL_ADAD51121 | -0.997663629 | 8.18E-06 |
| ata-miR9672-3p_R19-2L21 | Traes_5DL_1299E23A4 | -0.997590109 | 8.70E-06 |
| gga-miR-7475-5p_R20-6L20 | Traes_3DS_9E292BCAF | -0.997539178 | 9.08E-06 |
| gga-miR-7475-5p_R20-6L20 | Traes_6AS_263764436 | -0.997491345 | 9.43E-06 |
| tae-miR1131_R15-1L22 | TRAES3BF115600050CFD_g | -0.99748091 | 9.51E-06 |
| tae-miR9659-3p | Traes_4AL_9AE77B9B9 | -0.997405922 | 1.01E-05 |
| ata-miR9772b-3p_R1-20L21 | Traes_4AS_108523DC2 | -0.99739989 | 1.01E-05 |
| gga-miR-7475-5p_R20-6L20 | Traes_1AL_36750809D | -0.997386884 | 1.02E-05 |
| ppt-miR894_R1-17L20 | Traes_2AS_72EC97B33 | -0.997378505 | 1.03E-05 |
| tch-miR-378a-3p | Traes_1AL_2F2E5017B | -0.997369469 | 1.04E-05 |
| tae-miR1137a_R15-1L20 | Traes_5BS_7FCDB6383 | -0.997343894 | 1.06E-05 |
| tae-miR396-5p_R1-21L21 | Traes_2BL_0A7F21A11 | -0.997337031 | 1.06E-05 |
| hsa-miR-6882-5p_R15-1L22 | Traes_1AL_5B26F6A14 | -0.997321914 | 1.07E-05 |
| pma-miR-4543_R8-22L22 | Traes_1AL_5B26F6A14 | -0.997321914 | 1.07E-05 |
| tae-m0966-5p | Traes_1AL_5B26F6A14 | -0.997321914 | 1.07E-05 |
| tae-m1217-5p | Traes_1AL_5B26F6A14 | -0.997321914 | 1.07E-05 |
| tae-m1682-5p | Traes_1AL_5B26F6A14 | -0.997321914 | 1.07E-05 |
| tae-miR9659-3p | Traes_4BS_85666ADAF | -0.997317217 | 1.08E-05 |
| tae-miR1120a_R24-4L24 | Traes_5AL_0807B656A | -0.997269987 | 1.12E-05 |
| gra-miR7505b_R6-20L21 | Traes_4BL_5664064B6 | -0.997268015 | 1.12E-05 |
| mml-miR-892c-3p_R1-17L22 | Traes_4BL_5664064B6 | -0.997268015 | 1.12E-05 |
| tae-m0403-5p | Traes_4BL_5664064B6 | -0.997268015 | 1.12E-05 |
| tae-m2665-5p | Traes_4BL_5664064B6 | -0.997268015 | 1.12E-05 |
| tae-m2876-5p | Traes_4BL_5664064B6 | -0.997268015 | 1.12E-05 |
| tae-m4081-5p | Traes_4BL_5664064B6 | -0.997268015 | 1.12E-05 |
| bdi-miR5181d_R7-21L21 | Traes_4BL_0B3145B8C | -0.997267073 | 1.12E-05 |
| tae-m2905-5p | Traes_6AS_2A59D8EDC | -0.997249489 | 1.13E-05 |
| mmu-miR-1187_R7-21L23 | Traes_6AL_D50E09BFC | -0.997247426 | 1.14E-05 |
| tae-m2170-5p | Traes_6AL_D50E09BFC | -0.997247426 | 1.14E-05 |
| tch-miR-378a-3p | Traes_2BL_28A7E0BF8 | -0.997240901 | 1.14E-05 |
| cpa-miR8155_R4-18L19 | Traes_3DS_862E4ADF7 | -0.997220722 | 1.16E-05 |
| ata-miR167d-5p_R1-22L22 | Traes_6AL_BA5F44F2B | -0.997209383 | 1.17E-05 |
| tae-m3294-3p | Traes_5DS_42A1A9BAA | -0.997121428 | 1.24E-05 |
| ssp-miR444b.2_R15-1L21 | Traes_2AS_BF5FA8832 | -0.997099954 | 1.26E-05 |
| tae-m0044-5p | Traes_6AL_D7116C036 | -0.997058767 | 1.30E-05 |
| tae-miR9659-3p | Traes_1BL_28DB0E01C | -0.997020481 | 1.33E-05 |
| tae-miR9659-3p | Traes_4AS_1BEB20E15 | -0.997008187 | 1.34E-05 |
| gga-miR-7475-5p_R20-6L20 | Traes_2BS_457A03DD9 | -0.997000724 | 1.35E-05 |
| ata-miR167e-5p_R1-21L21 | Traes_7BS_C0604DCF9 | -0.996982986 | 1.36E-05 |
| tae-m3157-5p | Traes_7BS_C0604DCF9 | -0.996982986 | 1.36E-05 |
| tae-miR9659-3p | Traes_7DS_51E4B75F7 | -0.996955377 | 1.39E-05 |
| ata-miR167e-5p_R1-21L21 | Traes_4AS_0812D941C | -0.996937898 | 1.41E-05 |
| tae-m3157-5p | Traes_4AS_0812D941C | -0.996937898 | 1.41E-05 |
| mml-miR-7163-5p_R18-4L19 | Traes_3AL_32C0599AE | -0.996805518 | 1.53E-05 |
| lus-miR159b_R2-21L21 | Traes_7AS_BAD262E31 | -0.996795859 | 1.54E-05 |
| gga-miR-7475-5p_R20-6L20 | Traes_5DL_BB61F5EC0 | -0.996758248 | 1.57E-05 |
| tae-m3949-5p | Traes_6AL_DF6863EF7 | -0.996733773 | 1.60E-05 |
| tae-m0298-5p | Traes_5DL_1D8CB272F | -0.996689173 | 1.64E-05 |
| tae-m1521-5p | Traes_5DL_A5A32C538 | -0.996646675 | 1.68E-05 |
| tae-m2489-5p | Traes_5DL_A5A32C538 | -0.996646675 | 1.68E-05 |
| tae-m4416-5p | Traes_5DL_A5A32C538 | -0.996646675 | 1.68E-05 |
| tch-miR-378a-3p | Traes_2AL_D0C3751FD | -0.996632578 | 1.70E-05 |
| gra-miR7505b_R6-20L21 | Traes_7BL_306E3FC40 | -0.996610814 | 1.72E-05 |
| mml-miR-892c-3p_R1-17L22 | Traes_7BL_306E3FC40 | -0.996610814 | 1.72E-05 |
| tae-m0403-5p | Traes_7BL_306E3FC40 | -0.996610814 | 1.72E-05 |
| tae-m2665-5p | Traes_7BL_306E3FC40 | -0.996610814 | 1.72E-05 |
| tae-m2876-5p | Traes_7BL_306E3FC40 | -0.996610814 | 1.72E-05 |
| tae-m4081-5p | Traes_7BL_306E3FC40 | -0.996610814 | 1.72E-05 |
| gga-miR-7475-5p_R20-6L20 | Traes_1DL_CA3D844E5 | -0.996527251 | 1.81E-05 |
| ata-miR9772b-3p_R1-20L21 | Traes_5BL_8CB3F7560 | -0.996525751 | 1.81E-05 |
| tae-m0025-5p | TRAES3BF013000050CFD_g | -0.996515347 | 1.82E-05 |
| tae-m2737-5p | TRAES3BF013000050CFD_g | -0.996515347 | 1.82E-05 |
| chi-miR-133a-3p_R2-23L23 | Traes_7DL_C681F3459 | -0.996496971 | 1.84E-05 |
| gga-miR-7475-5p_R20-6L20 | Traes_5AS_EDF185C68 | -0.996472598 | 1.86E-05 |
| gma-miR5037a_R19-5L22 | Traes_1DS_C6BE97C0E | -0.996455484 | 1.88E-05 |
| ptr-miR-3937_R1-15L24 | TRAES3BF016700080CFD_g | -0.996447268 | 1.89E-05 |
| mtr-miR7701-5p_R18-4L21 | Traes_3AL_24EF0F7FE | -0.996417818 | 1.92E-05 |
| ptr-miR-3937_R1-15L24 | Traes_3DL_D083DCF99 | -0.99639299 | 1.95E-05 |
| tch-miR-378a-3p | Traes_7DS_C80D1B7F1 | -0.996316915 | 2.03E-05 |
| bdi-miR5174e-3p.2_R7-21L21 | Traes_2DL_C0FF8F111 | -0.996299061 | 2.05E-05 |
| gga-miR-7475-5p_R20-6L20 | Traes_2AL_2FF604DA9 | -0.9962955 | 2.06E-05 |
| tae-m2905-5p | Traes_7BL_F6D21882A | -0.996290711 | 2.06E-05 |
| tae-m0195-5p | Traes_5BL_458576406 | -0.996255895 | 2.10E-05 |
| tae-m0298-5p | TRAES3BF073700180CFD_g | -0.996227371 | 2.13E-05 |
| tae-m3294-3p | Traes_3AL_716569701 | -0.996183767 | 2.18E-05 |
| dme-miR-954-3p_R15-1L21 | Traes_4AS_9421592BC | -0.996182457 | 2.18E-05 |
| cpa-miR8155_R4-18L19 | Traes_5DL_AB1CB70B9 | -0.996169075 | 2.20E-05 |
| gga-miR-7475-5p_R20-6L20 | Traes_5BL_97BEC5636 | -0.996165943 | 2.20E-05 |
| tae-miR396-5p_R1-21L21 | Traes_1BL_8601EC2E0 | -0.996128146 | 2.25E-05 |
| gga-miR-7475-5p_R20-6L20 | Traes_1BL_EDDD35B93 | -0.99611215 | 2.26E-05 |
| tae-miR9659-3p | Traes_2BS_F19F39996 | -0.996101866 | 2.28E-05 |
| tae-m0599-5p | Traes_3AL_B8DBDA3BD | -0.996073075 | 2.31E-05 |
| osa-miR5072_R6-20L22 | Traes_1AS_E58B0B23A | -0.996058747 | 2.33E-05 |
| gra-miR7505b_R6-20L21 | Traes_7DS_44D3A516A | -0.996029555 | 2.36E-05 |
| mml-miR-892c-3p_R1-17L22 | Traes_7DS_44D3A516A | -0.996029555 | 2.36E-05 |
| tae-m0403-5p | Traes_7DS_44D3A516A | -0.996029555 | 2.36E-05 |
| tae-m2665-5p | Traes_7DS_44D3A516A | -0.996029555 | 2.36E-05 |
| tae-m2876-5p | Traes_7DS_44D3A516A | -0.996029555 | 2.36E-05 |
| tae-m4081-5p | Traes_7DS_44D3A516A | -0.996029555 | 2.36E-05 |
| ata-miR9672-3p_R19-2L21 | Traes_6DS_BDFEA01CE | -0.996017893 | 2.38E-05 |
| mmu-miR-1187_R7-21L23 | Traes_5BL_ECB922F43 | -0.996008378 | 2.39E-05 |
| tae-m2170-5p | Traes_5BL_ECB922F43 | -0.996008378 | 2.39E-05 |
| tae-m0143-5p | Traes_3AL_4ACBEDEF9 | -0.995943029 | 2.47E-05 |
| tae-m3967-5p | Traes_2DS_E72225729 | -0.995939307 | 2.47E-05 |
| tae-m3294-3p | Traes_5BL_010D71116 | -0.995924531 | 2.49E-05 |
| tae-m3294-3p | Traes_7AS_779E8A5D2 | -0.995902608 | 2.51E-05 |
| bdi-miR529-3p_R20-6L21 | Traes_4DS_B7F6F6B61 | -0.995897258 | 2.52E-05 |
| tae-miR9659-3p | Traes_7DL_D54D54535 | -0.995896702 | 2.52E-05 |
| tae-m3294-3p | Traes_2AS_78A7576A9 | -0.995884778 | 2.54E-05 |
| gga-miR-7475-5p_R20-6L20 | Traes_1DL_4D7C10235 | -0.995865008 | 2.56E-05 |
| tae-m2790-5p | Traes_5DL_0C788D94B | -0.995815132 | 2.62E-05 |
| ata-miR167e-5p_R1-21L21 | Traes_2BL_433D3147C | -0.995784346 | 2.66E-05 |
| tae-m3157-5p | Traes_2BL_433D3147C | -0.995784346 | 2.66E-05 |
| gra-miR7505b_R6-20L21 | Traes_4AS_6F3D0F407 | -0.995768766 | 2.68E-05 |
| mml-miR-892c-3p_R1-17L22 | Traes_4AS_6F3D0F407 | -0.995768766 | 2.68E-05 |
| tae-m0403-5p | Traes_4AS_6F3D0F407 | -0.995768766 | 2.68E-05 |
| tae-m2665-5p | Traes_4AS_6F3D0F407 | -0.995768766 | 2.68E-05 |
| tae-m2876-5p | Traes_4AS_6F3D0F407 | -0.995768766 | 2.68E-05 |
| tae-m4081-5p | Traes_4AS_6F3D0F407 | -0.995768766 | 2.68E-05 |
| cpa-miR8155_R4-18L19 | Traes_2DS_C106D3AD41 | -0.995766827 | 2.68E-05 |
| tae-m3709-5p | Traes_3AS_EF8D70BCB | -0.995738248 | 2.72E-05 |
| dpr-miR397_R17-1L21 | Traes_1DL_69D4A3E8B | -0.99570758 | 2.76E-05 |
| tae-m1322-5p | Traes_1DL_69D4A3E8B | -0.99570758 | 2.76E-05 |
| tae-m3641-5p | Traes_1DL_69D4A3E8B | -0.99570758 | 2.76E-05 |
| xtr-miR-428a_R7-22L22 | Traes_1DL_69D4A3E8B | -0.99570758 | 2.76E-05 |
| ata-miR167e-5p_R1-21L21 | Traes_7AS_5E7A92F55 | -0.995701478 | 2.77E-05 |
| tae-m3157-5p | Traes_7AS_5E7A92F55 | -0.995701478 | 2.77E-05 |
| tae-m0025-5p | Traes_4BL_8C7E52871 | -0.995686169 | 2.79E-05 |
| tae-m2737-5p | Traes_4BL_8C7E52871 | -0.995686169 | 2.79E-05 |
| tae-m3294-3p | Traes_1DS_E55F00FDB | -0.995679804 | 2.80E-05 |
| ssp-miR444b.2_R15-1L21 | TRAES3BF026100050CFD_g | -0.995659757 | 2.82E-05 |
| tae-m2507-5p | TRAES3BF096800020CFD_g | -0.995659339 | 2.82E-05 |
| cpa-miR8155_R4-18L19 | Traes_7BS_A765F2B82 | -0.995654071 | 2.83E-05 |
| gga-miR-7475-5p_R20-6L20 | Traes_6AL_72D2B7C35 | -0.995638418 | 2.85E-05 |
| tae-m2905-5p | Traes_6DL_3CC592FDB | -0.995633553 | 2.86E-05 |
| tae-m1477-5p | Traes_7DL_C074DC1F6 | -0.99563056 | 2.86E-05 |
| gga-miR-7475-5p_R20-6L20 | Traes_1DS_E55F00FDB | -0.995614852 | 2.88E-05 |
| ptr-miR-3937_R1-15L24 | Traes_2BS_2FD1D68FB | -0.995600526 | 2.90E-05 |
| hvu-miR6177_R6-22L22 | Traes_6AL_59221E786 | -0.995574915 | 2.93E-05 |
| tae-miR396-5p_R1-21L21 | Traes_2BS_AECFFDFDD | -0.995570569 | 2.94E-05 |
| bdi-miR529-3p_R20-6L21 | Traes_7AS_A8D17AEAC | -0.995558033 | 2.96E-05 |
| gga-miR-7475-5p_R20-6L20 | Traes_4BS_F7359FA2E | -0.995527363 | 3.00E-05 |
| mmu-miR-6903-3p_R2-16L20 | Traes_5DS_CE69C025E | -0.995524298 | 3.00E-05 |
| cpa-miR8155_R4-18L19 | Traes_5DL_C3E576653 | -0.995478489 | 3.06E-05 |
| tae-m3294-3p | Traes_4BS_F7359FA2E | -0.995477441 | 3.06E-05 |
| tae-m4103-5p | Traes_4BL_45EEA5E2E | -0.995473089 | 3.07E-05 |
| gga-miR-7475-5p_R20-6L20 | Traes_2AL_DE86B9FD4 | -0.995465623 | 3.08E-05 |
| ata-miR167e-5p_R1-21L21 | Traes_5BL_733D552E81 | -0.995430297 | 3.13E-05 |
| tae-m3157-5p | Traes_5BL_733D552E81 | -0.995430297 | 3.13E-05 |
| tae-m0025-5p | Traes_5DL_BFB1E5328 | -0.995429461 | 3.13E-05 |
| tae-m2737-5p | Traes_5DL_BFB1E5328 | -0.995429461 | 3.13E-05 |
| bdi-miR529-3p_R20-6L21 | Traes_6AS_7E92D1C0A | -0.99542862 | 3.13E-05 |
| tae-miR1121_R3-21L22 | Traes_4AS_5D6957D15 | -0.995405101 | 3.16E-05 |
| tae-m3949-5p | Traes_5BL_A9BFB8F58 | -0.995381584 | 3.19E-05 |
| ssp-miR444b.2_R15-1L21 | Traes_2DS_4646885A5 | -0.995379915 | 3.20E-05 |
| ata-miR167e-5p_R1-21L21 | Traes_2BS_E0B8D21CD | -0.995376645 | 3.20E-05 |
| tae-m3157-5p | Traes_2BS_E0B8D21CD | -0.995376645 | 3.20E-05 |
| ata-miR167f-5p_17A-G | Traes_2BS_1CDFC2FF61 | -0.995342688 | 3.25E-05 |
| gga-miR-7475-5p_R20-6L20 | Traes_2BL_CA5412466 | -0.995311735 | 3.29E-05 |
| mmu-miR-3535_R7-21L26 | TRAES3BF174600020CFD_g | -0.995299253 | 3.31E-05 |
| tae-miR9659-3p | TRAES3BF136200010CFD_g | -0.995291207 | 3.32E-05 |
| osa-miR5072_R6-20L22 | Traes_3AL_F5DBA35E4 | -0.995287534 | 3.33E-05 |
| tch-miR-378a-3p | Traes_2BS_457A03DD9 | -0.995281493 | 3.33E-05 |
| tae-miR1120a_R24-4L24 | Traes_5BL_95AD5074C | -0.995258653 | 3.37E-05 |
| tch-miR-378a-3p | TRAES3BF018900010CFD_g | -0.995236515 | 3.40E-05 |
| gra-miR7505b_R6-20L21 | Traes_5DL_69DE08EB5 | -0.995231476 | 3.41E-05 |
| mml-miR-892c-3p_R1-17L22 | Traes_5DL_69DE08EB5 | -0.995231476 | 3.41E-05 |
| tae-m0403-5p | Traes_5DL_69DE08EB5 | -0.995231476 | 3.41E-05 |
| tae-m2665-5p | Traes_5DL_69DE08EB5 | -0.995231476 | 3.41E-05 |
| tae-m2876-5p | Traes_5DL_69DE08EB5 | -0.995231476 | 3.41E-05 |
| tae-m4081-5p | Traes_5DL_69DE08EB5 | -0.995231476 | 3.41E-05 |
| smo-miR159_R2-21L21 | Traes_6AS_37E167F1A | -0.995205549 | 3.44E-05 |
| tae-m1832-5p | Traes_6AS_37E167F1A | -0.995205549 | 3.44E-05 |
| tae-m2038-5p | Traes_6AS_37E167F1A | -0.995205549 | 3.44E-05 |
| tae-m3208-5p | Traes_6AS_37E167F1A | -0.995205549 | 3.44E-05 |
| bmo-miR-3208_R4-19L22 | Traes_2BL_6552196A1 | -0.995203181 | 3.45E-05 |
| tae-m1452-5p | Traes_2BL_6552196A1 | -0.995203181 | 3.45E-05 |
| bta-miR-2429_R19-5L20 | Traes_2BS_2B483208E | -0.995186841 | 3.47E-05 |
| nta-miR156f | Traes_2BS_2B483208E | -0.995186841 | 3.47E-05 |
| tae-m4030-5p | Traes_2BS_2B483208E | -0.995186841 | 3.47E-05 |
| tae-m4349-5p | Traes_2BS_2B483208E | -0.995186841 | 3.47E-05 |
| tae-miR1121_R3-21L22 | Traes_5DL_3C48E8747 | -0.995146601 | 3.53E-05 |
| bdi-miR529-3p_R20-6L21 | Traes_5DS_82640BC66 | -0.995134651 | 3.54E-05 |
| ata-miR167e-5p_R1-21L21 | Traes_6DS_CDB16CE3F | -0.995099262 | 3.60E-05 |
| tae-m3157-5p | Traes_6DS_CDB16CE3F | -0.995099262 | 3.60E-05 |
| ata-miR9672-3p_R19-2L21 | Traes_5AL_5CCCCBEE1 | -0.995072398 | 3.64E-05 |
| tae-m3949-5p | Traes_2AL_B4A22DC4F | -0.995043726 | 3.68E-05 |
| tae-m0982-5p | Traes_2BL_4E0157603 | -0.995030744 | 3.70E-05 |
| bta-miR-2429_R19-5L20 | Traes_2BL_B805C339B | -0.994999502 | 3.74E-05 |
| nta-miR156f | Traes_2BL_B805C339B | -0.994999502 | 3.74E-05 |
| tae-m4030-5p | Traes_2BL_B805C339B | -0.994999502 | 3.74E-05 |
| tae-m4349-5p | Traes_2BL_B805C339B | -0.994999502 | 3.74E-05 |
| sly-miR5300_R21-6L22 | Traes_1BL_22EDF8650 | -0.994981707 | 3.77E-05 |
| tae-miR9659-3p | Traes_7DL_98EC3AE06 | -0.994971959 | 3.79E-05 |
| tae-m3294-3p | Traes_1AL_25576148E | -0.994968963 | 3.79E-05 |
| stu-miR6027_R6-20L22 | Traes_4BL_E6E34D4A3 | -0.994956648 | 3.81E-05 |
| tae-m0298-5p | TRAES3BF175100090CFD_g | -0.994948771 | 3.82E-05 |
| tae-m1602-5p | Traes_2BL_339C6874F | -0.994944418 | 3.83E-05 |
| tae-miR396-5p_R1-21L21 | TRAES3BF033900100CFD_g | -0.994934211 | 3.84E-05 |
| stu-miR6027_R6-20L22 | TRAES3BF266400050CFD_g | -0.994930446 | 3.85E-05 |
| tae-miR396-5p_R1-21L21 | Traes_6AL_939CC781A | -0.994925451 | 3.86E-05 |
| tae-m0298-5p | Traes_4DL_8883F7012 | -0.994923908 | 3.86E-05 |
| hsa-miR-6882-5p_R15-1L22 | Traes_4AS_4BEF2DFAD | -0.994905599 | 3.89E-05 |
| pma-miR-4543_R8-22L22 | Traes_4AS_4BEF2DFAD | -0.994905599 | 3.89E-05 |
| tae-m0966-5p | Traes_4AS_4BEF2DFAD | -0.994905599 | 3.89E-05 |
| tae-m1217-5p | Traes_4AS_4BEF2DFAD | -0.994905599 | 3.89E-05 |
| tae-m1682-5p | Traes_4AS_4BEF2DFAD | -0.994905599 | 3.89E-05 |
| tae-m3294-3p | Traes_4DL_BC04763DE | -0.994891349 | 3.91E-05 |
| bta-miR-2429_R19-5L20 | Traes_1DL_D5512C9E1 | -0.994879168 | 3.93E-05 |
| nta-miR156f | Traes_1DL_D5512C9E1 | -0.994879168 | 3.93E-05 |
| tae-m4030-5p | Traes_1DL_D5512C9E1 | -0.994879168 | 3.93E-05 |
| tae-m4349-5p | Traes_1DL_D5512C9E1 | -0.994879168 | 3.93E-05 |
| tae-miR9659-3p | Traes_2DL_B6C06C49C | -0.994877586 | 3.93E-05 |
| gga-miR-7475-5p_R20-6L20 | Traes_2BS_66D0C26F4 | -0.994860799 | 3.95E-05 |
| tae-miR396-5p_R1-21L21 | Traes_5AL_6B2E76ED8 | -0.994847549 | 3.98E-05 |
| gga-miR-7475-5p_R20-6L20 | Traes_2BL_5C9966D42 | -0.994841415 | 3.98E-05 |
| tae-m1602-5p | Traes_3AL_A306585EF | -0.994830444 | 4.00E-05 |
| ata-miR9672-3p_R19-2L21 | Traes_4BS_BB2611662 | -0.994818383 | 4.02E-05 |
| smo-miR159_R2-21L21 | Traes_3AL_52A44CE87 | -0.994814362 | 4.03E-05 |
| tae-m1832-5p | Traes_3AL_52A44CE87 | -0.994814362 | 4.03E-05 |
| tae-m2038-5p | Traes_3AL_52A44CE87 | -0.994814362 | 4.03E-05 |
| tae-m3208-5p | Traes_3AL_52A44CE87 | -0.994814362 | 4.03E-05 |
| gga-miR-7475-5p_R20-6L20 | Traes_3DL_7387D0C5C | -0.994808985 | 4.04E-05 |
| tae-m0982-5p | Traes_4AL_61A99532C | -0.994791358 | 4.06E-05 |
| tae-miR396-5p_R1-21L21 | Traes_7BS_49D2E9794 | -0.994763237 | 4.11E-05 |
| tae-m2905-5p | Traes_1BL_59A688795 | -0.994756518 | 4.12E-05 |
| gra-miR7505b_R6-20L21 | Traes_4AS_A3EAF8C80 | -0.994717458 | 4.18E-05 |
| mml-miR-892c-3p_R1-17L22 | Traes_4AS_A3EAF8C80 | -0.994717458 | 4.18E-05 |
| tae-m0403-5p | Traes_4AS_A3EAF8C80 | -0.994717458 | 4.18E-05 |
| tae-m2665-5p | Traes_4AS_A3EAF8C80 | -0.994717458 | 4.18E-05 |
| tae-m2876-5p | Traes_4AS_A3EAF8C80 | -0.994717458 | 4.18E-05 |
| tae-m4081-5p | Traes_4AS_A3EAF8C80 | -0.994717458 | 4.18E-05 |
| ata-miR9772b-3p_R1-20L21 | Traes_4BS_78A2FF067 | -0.994713046 | 4.19E-05 |
| gra-miR7505b_R6-20L21 | Traes_1AL_CC3EB56C4 | -0.994697245 | 4.21E-05 |
| mml-miR-892c-3p_R1-17L22 | Traes_1AL_CC3EB56C4 | -0.994697245 | 4.21E-05 |
| tae-m0403-5p | Traes_1AL_CC3EB56C4 | -0.994697245 | 4.21E-05 |
| tae-m2665-5p | Traes_1AL_CC3EB56C4 | -0.994697245 | 4.21E-05 |
| tae-m2876-5p | Traes_1AL_CC3EB56C4 | -0.994697245 | 4.21E-05 |
| tae-m4081-5p | Traes_1AL_CC3EB56C4 | -0.994697245 | 4.21E-05 |
| gga-miR-7475-5p_R20-6L20 | Traes_6DL_C26461F78 | -0.99469003 | 4.22E-05 |
| gga-miR-7475-5p_R20-6L20 | Traes_7AS_85178E0D7 | -0.994688681 | 4.22E-05 |
| bdi-miR529-3p_R20-6L21 | Traes_6AL_3CBFEF197 | -0.994678741 | 4.24E-05 |
| tae-m1477-5p | Traes_7AL_09A36AAE41 | -0.994672028 | 4.25E-05 |
| tae-m0298-5p | Traes_4AL_4B9D56131 | -0.994644039 | 4.30E-05 |
| tch-miR-378a-3p | Traes_4AL_D25430175 | -0.994635096 | 4.31E-05 |
| gga-miR-7475-5p_R20-6L20 | Traes_6AL_074141030 | -0.99462569 | 4.32E-05 |
| tae-m3294-3p | Traes_6DL_D02E85399 | -0.994612026 | 4.35E-05 |
| ata-miR9672-3p_R19-2L21 | Traes_7AL_88DBB5A9A | -0.99460057 | 4.37E-05 |
| tae-m0298-5p | Traes_5BL_02932F5DC | -0.994573312 | 4.41E-05 |
| tae-m0298-5p | Traes_2DL_A80613857 | -0.994571244 | 4.41E-05 |
| tae-m2905-5p | Traes_3DL_7D692A7FF | -0.994569071 | 4.42E-05 |
| tch-miR-378a-3p | Traes_1DL_4D7C10235 | -0.994542186 | 4.46E-05 |
| tae-m0169-5p | Traes_5DL_7E789B13F | -0.994522424 | 4.49E-05 |
| tae-m1477-5p | Traes_7DL_FAD36DCB8 | -0.994516808 | 4.50E-05 |
| ata-miR167e-5p_R1-21L21 | Traes_4AL_E8EEC0D6E | -0.994505734 | 4.52E-05 |
| tae-m3157-5p | Traes_4AL_E8EEC0D6E | -0.994505734 | 4.52E-05 |
| tae-m4432-5p | Traes_2DL_BE5A2ECE6 | -0.994489019 | 4.55E-05 |
| bbe-miR-4857-5p_R3-17L23 | Traes_4BS_6B669D720 | -0.994474556 | 4.57E-05 |
| hsa-miR-3677-5p_R17-3L22 | Traes_4BS_6B669D720 | -0.994474556 | 4.57E-05 |
| tch-miR-378a-3p | Traes_2AL_295E0C7C5 | -0.994459503 | 4.60E-05 |
| mtr-miR7701-5p_R18-4L21 | Traes_6DL_3A42E1C80 | -0.994447261 | 4.62E-05 |
| ssp-miR444b.2_R15-1L21 | Traes_3AS_AE28A51DB | -0.994433732 | 4.64E-05 |
| ata-miR9772b-3p_R1-20L21 | Traes_5BL_19B18C00C | -0.994432896 | 4.64E-05 |
| tch-miR-378a-3p | Traes_1BL_5CD8FB94C | -0.994431017 | 4.64E-05 |
| gma-miR6300_R3-18L18 | Traes_5BL_F63566208 | -0.994415022 | 4.67E-05 |
| tch-miR-378a-3p | Traes_6AL_8B1EA1513 | -0.99439701 | 4.70E-05 |
| tae-m3294-3p | Traes_2DL_5436C046D | -0.994386759 | 4.72E-05 |
| tch-miR-378a-3p | Traes_4AL_9D0E18A90 | -0.994385279 | 4.72E-05 |
| bdi-miR529-3p_R20-6L21 | Traes_6BS_46BD91D05 | -0.994379373 | 4.73E-05 |
| efu-miR-9189e_R24-4L24_8A-C | Traes_2AS_9ADD06F95 | -0.994371493 | 4.74E-05 |
| tae-miR9659-3p | Traes_5BL_A0350B6AB | -0.994362612 | 4.76E-05 |
| tae-miR171a_R1-16L21 | Traes_1AS_BEEC10025 | -0.994344181 | 4.79E-05 |
| tch-miR-378a-3p | Traes_1AL_34404D5D8 | -0.994343362 | 4.79E-05 |
| gga-miR-7475-5p_R20-6L20 | Traes_6AL_E3E3F32E0 | -0.994333368 | 4.81E-05 |
| tae-m0488-5p | Traes_3DL_D292A338C | -0.994332503 | 4.81E-05 |
| tae-m4293-5p | Traes_3DL_D292A338C | -0.994332503 | 4.81E-05 |
| mmu-miR-1187_R7-21L23 | Traes_7AL_3F7F0AA7B | -0.994314892 | 4.84E-05 |
| tae-m2170-5p | Traes_7AL_3F7F0AA7B | -0.994314892 | 4.84E-05 |
| gra-miR7505b_R6-20L21 | Traes_4BL_8E6854176 | -0.994297535 | 4.87E-05 |
| mml-miR-892c-3p_R1-17L22 | Traes_4BL_8E6854176 | -0.994297535 | 4.87E-05 |
| tae-m0403-5p | Traes_4BL_8E6854176 | -0.994297535 | 4.87E-05 |
| tae-m2665-5p | Traes_4BL_8E6854176 | -0.994297535 | 4.87E-05 |
| tae-m2876-5p | Traes_4BL_8E6854176 | -0.994297535 | 4.87E-05 |
| tae-m4081-5p | Traes_4BL_8E6854176 | -0.994297535 | 4.87E-05 |
| tae-miR9659-3p | Traes_4AL_BEAEAEE4A | -0.994242898 | 4.96E-05 |
| tae-m3294-3p | Traes_2AL_DE86B9FD4 | -0.994240537 | 4.97E-05 |
| tae-m3294-3p | Traes_1DL_ADAD51121 | -0.99423897 | 4.97E-05 |
| ppy-miR-608_R7-21L25 | Traes_7DS_86E306316 | -0.994226149 | 4.99E-05 |
| gga-miR-7475-5p_R20-6L20 | Traes_2BL_34819D129 | -0.994225494 | 4.99E-05 |
| tae-m2905-5p | Traes_6DS_33D6888AF | -0.994223426 | 5.00E-05 |
| tae-m3738-5p | Traes_4BS_C9070AEA4 | -0.994220964 | 5.00E-05 |
| cpa-miR8155_R4-18L19 | Traes_4BS_DE9B5B1AB | -0.994169968 | 5.09E-05 |
| tae-m2840-5p | Traes_2DL_41666605D | -0.994163813 | 5.10E-05 |
| ata-miR9772b-3p_R1-20L21 | Traes_6AS_6C7C4893E | -0.994145099 | 5.13E-05 |
| tae-m2748-5p | Traes_7AL_7A572FF1F | -0.994144608 | 5.13E-05 |
| tae-m2333-5p | Traes_2DS_89AB214A2 | -0.99413829 | 5.14E-05 |
| tae-m3808-5p | Traes_2DS_89AB214A2 | -0.99413829 | 5.14E-05 |
| tae-m3294-3p | Traes_2DL_12CC97DAF | -0.994134269 | 5.15E-05 |
| cpa-miR8155_R4-18L19 | Traes_5BL_BE75F32CC | -0.994130603 | 5.16E-05 |
| tae-miR9659-3p | Traes_5DL_ECC866A4B1 | -0.994114072 | 5.19E-05 |
| hsa-miR-6882-5p_R15-1L22 | Traes_2DL_11F6A8A27 | -0.994088525 | 5.23E-05 |
| pma-miR-4543_R8-22L22 | Traes_2DL_11F6A8A27 | -0.994088525 | 5.23E-05 |
| tae-m0966-5p | Traes_2DL_11F6A8A27 | -0.994088525 | 5.23E-05 |
| tae-m1217-5p | Traes_2DL_11F6A8A27 | -0.994088525 | 5.23E-05 |
| tae-m1682-5p | Traes_2DL_11F6A8A27 | -0.994088525 | 5.23E-05 |
| dpr-miR397_R17-1L21 | Traes_2DL_AB02E0580 | -0.994088167 | 5.23E-05 |
| tae-m1322-5p | Traes_2DL_AB02E0580 | -0.994088167 | 5.23E-05 |
| tae-m3641-5p | Traes_2DL_AB02E0580 | -0.994088167 | 5.23E-05 |
| xtr-miR-428a_R7-22L22 | Traes_2DL_AB02E0580 | -0.994088167 | 5.23E-05 |
| tae-miR9659-3p | Traes_2DS_9570A4143 | -0.994082132 | 5.24E-05 |
| tae-m2660-5p | Traes_2AL_C2979B0C3 | -0.994077834 | 5.25E-05 |
| gga-miR-7475-5p_R20-6L20 | Traes_6BL_5B70744B1 | -0.994073573 | 5.26E-05 |
| tae-miR396-5p_R1-21L21 | Traes_7AS_6F12B2064 | -0.994042716 | 5.31E-05 |
| tae-m3738-5p | TRAES3BF074500010CFD_g | -0.994026587 | 5.34E-05 |
| osa-miR5072_R6-20L22 | Traes_6AS_ADA72EB86 | -0.994026317 | 5.34E-05 |
| tae-m4103-5p | Traes_1AL_F4304F02B | -0.994015195 | 5.36E-05 |
| ata-miR167d-5p_R1-22L22 | Traes_4DS_79D16595F | -0.994006084 | 5.38E-05 |
| tae-m3294-3p | Traes_1BL_0302D9F63 | -0.993984604 | 5.42E-05 |
| gga-miR-7475-5p_R20-6L20 | Traes_6BL_F4597CA77 | -0.993968428 | 5.45E-05 |
| tch-miR-378a-3p | Traes_2BL_DA7C50D42 | -0.993965097 | 5.45E-05 |
| gga-miR-7475-5p_R20-6L20 | Traes_7AL_2EA20BAA3 | -0.9939603 | 5.46E-05 |
| ppt-miR894_R1-17L20 | Traes_4AS_B4AE9F889 | -0.993954947 | 5.47E-05 |
| gra-miR7505b_R6-20L21 | Traes_3DS_C0A4FDDDD | -0.993942451 | 5.49E-05 |
| mml-miR-892c-3p_R1-17L22 | Traes_3DS_C0A4FDDDD | -0.993942451 | 5.49E-05 |
| tae-m0403-5p | Traes_3DS_C0A4FDDDD | -0.993942451 | 5.49E-05 |
| tae-m2665-5p | Traes_3DS_C0A4FDDDD | -0.993942451 | 5.49E-05 |
| tae-m2876-5p | Traes_3DS_C0A4FDDDD | -0.993942451 | 5.49E-05 |
| tae-m4081-5p | Traes_3DS_C0A4FDDDD | -0.993942451 | 5.49E-05 |
| gga-miR-7475-5p_R20-6L20 | Traes_3AL_D2D34DED1 | -0.993909134 | 5.55E-05 |
| tae-m0025-5p | Traes_2BL_8ABEDF0A6 | -0.993872658 | 5.62E-05 |
| tae-m2737-5p | Traes_2BL_8ABEDF0A6 | -0.993872658 | 5.62E-05 |
| bdi-miR529-3p_R20-6L21 | Traes_1AL_F865B9A4A | -0.993859772 | 5.64E-05 |
| ata-miR9672-3p_R19-2L21 | Traes_4BL_AF74BC98E | -0.993855657 | 5.65E-05 |
| tae-m4222-3p | Traes_3DL_55B6B2012 | -0.99384442 | 5.67E-05 |
| tae-m1498-5p | Traes_2DL_B7ABC1CB9 | -0.993829654 | 5.70E-05 |
| tae-m4236-5p | Traes_2DL_B7ABC1CB9 | -0.993829654 | 5.70E-05 |
| gga-miR-7475-5p_R20-6L20 | Traes_5DL_F7C53022D | -0.993827583 | 5.70E-05 |
| mtr-miR7701-5p_R18-4L21 | Traes_2BS_0B2869248 | -0.993827258 | 5.70E-05 |
| tae-m0298-5p | Traes_6DL_F9589C62A | -0.993821711 | 5.71E-05 |
| bdi-miR529-3p_R20-6L21 | Traes_4BL_FE84D2A93 | -0.993769048 | 5.81E-05 |
| tae-miR9659-3p | Traes_4DS_4C6846850 | -0.993730022 | 5.88E-05 |
| tae-miR396-5p_R1-21L21 | Traes_6BS_257A12B34 | -0.993726926 | 5.89E-05 |
| gga-miR-7475-5p_R20-6L20 | Traes_4DS_EC9D5061D | -0.993721935 | 5.90E-05 |
| gra-miR7505b_R6-20L21 | Traes_5BL_1CF10603C | -0.993710986 | 5.92E-05 |
| mml-miR-892c-3p_R1-17L22 | Traes_5BL_1CF10603C | -0.993710986 | 5.92E-05 |
| tae-m0403-5p | Traes_5BL_1CF10603C | -0.993710986 | 5.92E-05 |
| tae-m2665-5p | Traes_5BL_1CF10603C | -0.993710986 | 5.92E-05 |
| tae-m2876-5p | Traes_5BL_1CF10603C | -0.993710986 | 5.92E-05 |
| tae-m4081-5p | Traes_5BL_1CF10603C | -0.993710986 | 5.92E-05 |
| gga-miR-7475-5p_R20-6L20 | Traes_2BL_DA7C50D42 | -0.99370751 | 5.93E-05 |
| tae-m3738-5p | Traes_6DS_50D1DE754 | -0.993678247 | 5.98E-05 |
| tae-m0169-5p | Traes_7AS_406885D49 | -0.99367572 | 5.99E-05 |
| tae-m0982-5p | TRAES3BF268900100CFD_g | -0.993661742 | 6.01E-05 |
| tae-m0143-5p | TRAES3BF266400050CFD_g | -0.993659061 | 6.02E-05 |
| tae-m3709-5p | Traes_7BL_4E1837613 | -0.993657982 | 6.02E-05 |
| gra-miR7505b_R6-20L21 | TRAES3BF038000030CFD_g | -0.993653871 | 6.03E-05 |
| mml-miR-892c-3p_R1-17L22 | TRAES3BF038000030CFD_g | -0.993653871 | 6.03E-05 |
| tae-m0403-5p | TRAES3BF038000030CFD_g | -0.993653871 | 6.03E-05 |
| tae-m2665-5p | TRAES3BF038000030CFD_g | -0.993653871 | 6.03E-05 |
| tae-m2876-5p | TRAES3BF038000030CFD_g | -0.993653871 | 6.03E-05 |
| tae-m4081-5p | TRAES3BF038000030CFD_g | -0.993653871 | 6.03E-05 |
| tae-m2724-5p | Traes_7DS_4301CA3BC | -0.993637215 | 6.06E-05 |
| mmu-miR-1258-3p_R4-18L22 | Traes_6DL_418BF9785 | -0.993626279 | 6.08E-05 |
| tae-m0298-5p | Traes_2BL_01FC4C993 | -0.9936152 | 6.10E-05 |
| tae-m3294-3p | Traes_4AL_92708BAA0 | -0.993611211 | 6.11E-05 |
| tae-m1602-5p | Traes_5BL_128918E7A | -0.99360211 | 6.13E-05 |
| oha-miR-99a-5p | Traes_6BL_5B613F9E5 | -0.993571223 | 6.19E-05 |
| gga-miR-7475-5p_R20-6L20 | TRAES3BF059200050CFD_g | -0.993569043 | 6.19E-05 |
| tae-m2905-5p | Traes_6BS_8020617A51 | -0.993568059 | 6.19E-05 |
| tae-m0431-5p | Traes_2BL_7100AB5AD | -0.993566047 | 6.20E-05 |
| tae-m3294-3p | Traes_6AL_E3E3F32E0 | -0.993530181 | 6.27E-05 |
| gra-miR7505b_R6-20L21 | Traes_3DS_BA1FCA793 | -0.993528006 | 6.27E-05 |
| mml-miR-892c-3p_R1-17L22 | Traes_3DS_BA1FCA793 | -0.993528006 | 6.27E-05 |
| tae-m0403-5p | Traes_3DS_BA1FCA793 | -0.993528006 | 6.27E-05 |
| tae-m2665-5p | Traes_3DS_BA1FCA793 | -0.993528006 | 6.27E-05 |
| tae-m2876-5p | Traes_3DS_BA1FCA793 | -0.993528006 | 6.27E-05 |
| tae-m4081-5p | Traes_3DS_BA1FCA793 | -0.993528006 | 6.27E-05 |
| tae-m0298-5p | Traes_6BL_A9EFABD8F | -0.993524906 | 6.28E-05 |
| gga-miR-7475-5p_R20-6L20 | Traes_4DS_993693839 | -0.993517923 | 6.29E-05 |
| tae-m3949-5p | Traes_2BL_E3D7C17C2 | -0.993509467 | 6.31E-05 |
| gga-miR-7475-5p_R20-6L20 | Traes_2AS_78A7576A9 | -0.993508405 | 6.31E-05 |
| ata-miR9672-3p_R19-2L21 | Traes_2BL_B64F61BB8 | -0.993442502 | 6.44E-05 |
| tae-m2507-5p | Traes_1DL_B2CA253F1 | -0.993405752 | 6.51E-05 |
| bdi-miR5181d_R7-21L21 | Traes_6DS_4EAAEFBB0 | -0.993391091 | 6.54E-05 |
| ppy-miR-4451_R1-16L18 | Traes_2DL_892F83E0B | -0.99338555 | 6.55E-05 |
| tae-m1498-5p | Traes_7AL_F37BE187C | -0.993385157 | 6.55E-05 |
| tae-m4236-5p | Traes_7AL_F37BE187C | -0.993385157 | 6.55E-05 |
| tae-m1521-5p | Traes_4AL_DA3A16A9A | -0.993345864 | 6.63E-05 |
| tae-m2489-5p | Traes_4AL_DA3A16A9A | -0.993345864 | 6.63E-05 |
| tae-m4416-5p | Traes_4AL_DA3A16A9A | -0.993345864 | 6.63E-05 |
| ppy-miR-4451_R1-16L18 | Traes_3AL_B3DB454DC | -0.993317353 | 6.68E-05 |
| hsa-miR-6882-5p_R15-1L22 | Traes_2AS_B264257CD | -0.993311999 | 6.69E-05 |
| pma-miR-4543_R8-22L22 | Traes_2AS_B264257CD | -0.993311999 | 6.69E-05 |
| tae-m0966-5p | Traes_2AS_B264257CD | -0.993311999 | 6.69E-05 |
| tae-m1217-5p | Traes_2AS_B264257CD | -0.993311999 | 6.69E-05 |
| tae-m1682-5p | Traes_2AS_B264257CD | -0.993311999 | 6.69E-05 |
| tae-m0298-5p | Traes_6BS_6D2A626DE | -0.993280197 | 6.76E-05 |
| gga-miR-7475-5p_R20-6L20 | Traes_4AS_49955C28B | -0.993271691 | 6.78E-05 |
| tae-m3294-3p | Traes_4AL_234D9FEC7 | -0.993256836 | 6.81E-05 |
| tae-m2748-5p | Traes_2DL_C46B73FA3 | -0.993251868 | 6.82E-05 |
| ppy-miR-4451_R1-16L18 | Traes_2AL_B50371043 | -0.993249012 | 6.82E-05 |
| tae-m3294-3p | Traes_6AL_0361DD7E9 | -0.993234721 | 6.85E-05 |
| tch-miR-378a-3p | Traes_6AL_116E0BA24 | -0.993232209 | 6.85E-05 |
| tae-m1602-5p | Traes_5DL_7BEC640FA | -0.993225497 | 6.87E-05 |
| bdi-miR5181d_R7-21L21 | Traes_5BL_1505DCD78 | -0.993222059 | 6.88E-05 |
| tch-miR-378a-3p | Traes_2DL_5436C046D | -0.99320856 | 6.90E-05 |
| gga-miR-7475-5p_R20-6L20 | Traes_1BL_F32B62E49 | -0.993204463 | 6.91E-05 |
| hvu-miR6177_R6-22L22 | Traes_4AS_56BE1072A | -0.993197903 | 6.92E-05 |
| dpr-miR397_R17-1L21 | Traes_2DS_D3BA8D38F1 | -0.993197856 | 6.92E-05 |
| tae-m1322-5p | Traes_2DS_D3BA8D38F1 | -0.993197856 | 6.92E-05 |
| tae-m3641-5p | Traes_2DS_D3BA8D38F1 | -0.993197856 | 6.92E-05 |
| xtr-miR-428a_R7-22L22 | Traes_2DS_D3BA8D38F1 | -0.993197856 | 6.92E-05 |
| gga-miR-7475-5p_R20-6L20 | Traes_5BL_67DAB39A6 | -0.993181421 | 6.96E-05 |
| cpa-miR8155_R4-18L19 | Traes_2BS_B36CAD1D5 | -0.993174707 | 6.97E-05 |
| tae-miR396-5p_R1-21L21 | TRAES3BF044000030CFD_g | -0.993148962 | 7.02E-05 |
| bta-miR-2429_R19-5L20 | TRAES3BF111700080CFD_g | -0.993141438 | 7.04E-05 |
| nta-miR156f | TRAES3BF111700080CFD_g | -0.993141438 | 7.04E-05 |
| tae-m4030-5p | TRAES3BF111700080CFD_g | -0.993141438 | 7.04E-05 |
| tae-m4349-5p | TRAES3BF111700080CFD_g | -0.993141438 | 7.04E-05 |
| tch-miR-378a-3p | Traes_1BL_68E4D6F70 | -0.993133305 | 7.06E-05 |
| tae-m0298-5p | Traes_5BS_99ED7E919 | -0.99311812 | 7.09E-05 |
| tae-m0298-5p | Traes_5BL_10BF821D1 | -0.993116664 | 7.09E-05 |
| gga-miR-7475-5p_R20-6L20 | Traes_1BL_71EF97B701 | -0.993109431 | 7.11E-05 |
| tae-m3294-3p | Traes_6AS_263764436 | -0.993093988 | 7.14E-05 |
| ppy-miR-608_R7-21L25 | Traes_2AS_A8BF6F543 | -0.993077595 | 7.17E-05 |
| gma-miR6300_R3-18L18 | Traes_4DL_4A0341E1A | -0.993063015 | 7.20E-05 |
| osa-miR5072_R6-20L22 | TRAES3BF270400010CFD_g | -0.993058614 | 7.21E-05 |
| bdi-miR159b-3p.1_R1-21L21 | Traes_6DL_C1C8B54BF | -0.99305051 | 7.23E-05 |
| tae-miR9659-3p | Traes_5BL_CDB206601 | -0.99304571 | 7.24E-05 |
| gra-miR7505b_R6-20L21 | Traes_6AL_01DB61EF6 | -0.993027552 | 7.28E-05 |
| mml-miR-892c-3p_R1-17L22 | Traes_6AL_01DB61EF6 | -0.993027552 | 7.28E-05 |
| tae-m0403-5p | Traes_6AL_01DB61EF6 | -0.993027552 | 7.28E-05 |
| tae-m2665-5p | Traes_6AL_01DB61EF6 | -0.993027552 | 7.28E-05 |
| tae-m2876-5p | Traes_6AL_01DB61EF6 | -0.993027552 | 7.28E-05 |
| tae-m4081-5p | Traes_6AL_01DB61EF6 | -0.993027552 | 7.28E-05 |
| gga-miR-7475-5p_R20-6L20 | Traes_5BL_010D71116 | -0.993026019 | 7.28E-05 |
| tae-miR396-5p_R1-21L21 | Traes_7AS_31BBD1E59 | -0.992996923 | 7.34E-05 |
| oha-miR-22a_R1-19L22 | Traes_6BL_5B613F9E5 | -0.992994207 | 7.34E-05 |
| tae-miR396-5p_R1-21L21 | Traes_4BL_C247E3780 | -0.992981492 | 7.37E-05 |
| tae-miR396-5p_R1-21L21 | Traes_2DL_28FF6250C | -0.99298009 | 7.37E-05 |
| gga-miR-7475-5p_R20-6L20 | Traes_2DS_C53EC62E8 | -0.992958115 | 7.42E-05 |
| gga-miR-7475-5p_R20-6L20 | Traes_7BL_C86E000C5 | -0.992936116 | 7.47E-05 |
| tae-miR9659-3p | Traes_6AL_E9A292FCD | -0.992928227 | 7.48E-05 |
| hsa-miR-6882-5p_R15-1L22 | Traes_4DL_A780BF320 | -0.992925248 | 7.49E-05 |
| pma-miR-4543_R8-22L22 | Traes_4DL_A780BF320 | -0.992925248 | 7.49E-05 |
| tae-m0966-5p | Traes_4DL_A780BF320 | -0.992925248 | 7.49E-05 |
| tae-m1217-5p | Traes_4DL_A780BF320 | -0.992925248 | 7.49E-05 |
| tae-m1682-5p | Traes_4DL_A780BF320 | -0.992925248 | 7.49E-05 |
| tae-m4432-5p | TRAES3BF026400040CFD_g | -0.992920344 | 7.50E-05 |
| tae-miR1121_R3-21L22 | Traes_2BL_CA7D819C8 | -0.992915177 | 7.51E-05 |
| tae-miR396-5p_R1-21L21 | Traes_5DL_6BF85DB4D | -0.992913372 | 7.52E-05 |
| gga-miR-7475-5p_R20-6L20 | Traes_6DL_F1E787360 | -0.992910165 | 7.52E-05 |
| bdi-miR390a-3p | Traes_1DL_E219BD119 | -0.992888906 | 7.57E-05 |
| tae-m3294-3p | Traes_5BL_67DAB39A6 | -0.992887773 | 7.57E-05 |
| tae-miR396-5p_R1-21L21 | Traes_3AS_074469002 | -0.992886297 | 7.57E-05 |
| tae-m3949-5p | Traes_2AL_721710827 | -0.992874381 | 7.60E-05 |
| tae-m0431-5p | Traes_4DS_39D51FA67 | -0.99286101 | 7.63E-05 |
| gga-miR-7475-5p_R20-6L20 | Traes_1AS_8A319EC97 | -0.992851076 | 7.65E-05 |
| bta-miR-2285e_R5-19L22 | Traes_1DS_5833EEE5F | -0.992850256 | 7.65E-05 |
| tae-m3294-3p | Traes_2BS_457A03DD9 | -0.992848097 | 7.65E-05 |
| gra-miR7505b_R6-20L21 | Traes_4AS_1938645BA | -0.992841035 | 7.67E-05 |
| mml-miR-892c-3p_R1-17L22 | Traes_4AS_1938645BA | -0.992841035 | 7.67E-05 |
| tae-m0403-5p | Traes_4AS_1938645BA | -0.992841035 | 7.67E-05 |
| tae-m2665-5p | Traes_4AS_1938645BA | -0.992841035 | 7.67E-05 |
| tae-m2876-5p | Traes_4AS_1938645BA | -0.992841035 | 7.67E-05 |
| tae-m4081-5p | Traes_4AS_1938645BA | -0.992841035 | 7.67E-05 |
| tae-m2905-5p | Traes_2BS_990895438 | -0.992829873 | 7.69E-05 |
| tae-m0298-5p | Traes_7DL_8E0F29CAE | -0.992822012 | 7.71E-05 |
| tae-m0298-5p | Traes_3AL_81435CD25 | -0.992778334 | 7.80E-05 |
| gga-miR-7475-5p_R20-6L20 | Traes_2DL_6CD064E13 | -0.992760941 | 7.84E-05 |
| tae-m4103-5p | Traes_1DL_F17BA285B | -0.992758143 | 7.85E-05 |
| tae-miR9659-3p | Traes_6BL_34B081BBE | -0.992755244 | 7.85E-05 |
| bdi-miR156f-3p | Traes_7AS_BAD262E31 | -0.992753933 | 7.86E-05 |
| tae-miR171a_R1-16L21 | Traes_2BS_A1D759B23 | -0.992732796 | 7.90E-05 |
| tae-m2905-5p | Traes_6BL_B1B21B021 | -0.992716571 | 7.94E-05 |
| tae-m2507-5p | Traes_7DS_FBCD836CA | -0.992716204 | 7.94E-05 |
| tch-miR-378a-3p | Traes_1AL_36750809D | -0.992713183 | 7.95E-05 |
| ata-miR167e-5p_R1-21L21 | Traes_6BS_6D2A626DE | -0.992712869 | 7.95E-05 |
| tae-m3157-5p | Traes_6BS_6D2A626DE | -0.992712869 | 7.95E-05 |
| dpr-miR397_R17-1L21 | Traes_3DL_2BC4CDDAA | -0.992712797 | 7.95E-05 |
| tae-m1322-5p | Traes_3DL_2BC4CDDAA | -0.992712797 | 7.95E-05 |
| tae-m3641-5p | Traes_3DL_2BC4CDDAA | -0.992712797 | 7.95E-05 |
| xtr-miR-428a_R7-22L22 | Traes_3DL_2BC4CDDAA | -0.992712797 | 7.95E-05 |
| tae-m3625-3p | Traes_5DS_CE69C025E | -0.992709248 | 7.95E-05 |
| tae-m3312-5p | Traes_6BL_ADE1875EF | -0.992698227 | 7.98E-05 |
| osa-miR5072_R6-20L22 | Traes_3DS_EC6FE9271 | -0.992680915 | 8.02E-05 |
| tae-m0195-5p | Traes_2DS_C4237A91B | -0.992662741 | 8.06E-05 |
| tae-m4103-5p | Traes_1AL_9907E1500 | -0.99265713 | 8.07E-05 |
| osa-miR5072_R6-20L22 | Traes_4BL_704315653 | -0.992627851 | 8.13E-05 |
| tae-m2905-5p | Traes_2DS_352C4C75C | -0.992618127 | 8.15E-05 |
| tch-miR-378a-3p | Traes_6DL_C26461F78 | -0.992615161 | 8.16E-05 |
| mtr-miR2655b_R4-18L21 | Traes_2AL_EE95E4780 | -0.992606745 | 8.18E-05 |
| mtr-miR7701-5p_R18-4L21 | Traes_5DL_19CF37E84 | -0.992591067 | 8.21E-05 |
| ata-miR9672-3p_R19-2L21 | Traes_5DL_99B0AF349 | -0.992586482 | 8.22E-05 |
| tae-m3294-3p | Traes_5AS_EDF185C68 | -0.992583038 | 8.23E-05 |
| tae-miR396-5p_R1-21L21 | Traes_3DL_0D2EA3B33 | -0.992570501 | 8.26E-05 |
| gra-miR7505b_R6-20L21 | Traes_6AL_2017727C4 | -0.992565323 | 8.27E-05 |
| mml-miR-892c-3p_R1-17L22 | Traes_6AL_2017727C4 | -0.992565323 | 8.27E-05 |
| tae-m0403-5p | Traes_6AL_2017727C4 | -0.992565323 | 8.27E-05 |
| tae-m2665-5p | Traes_6AL_2017727C4 | -0.992565323 | 8.27E-05 |
| tae-m2876-5p | Traes_6AL_2017727C4 | -0.992565323 | 8.27E-05 |
| tae-m4081-5p | Traes_6AL_2017727C4 | -0.992565323 | 8.27E-05 |
| tae-m0025-5p | Traes_6DL_5F7D8CF66 | -0.992500194 | 8.42E-05 |
| tae-m2737-5p | Traes_6DL_5F7D8CF66 | -0.992500194 | 8.42E-05 |
| ata-miR167e-5p_R1-21L21 | Traes_1BS_D4CB5F655 | -0.992482106 | 8.46E-05 |
| tae-m3157-5p | Traes_1BS_D4CB5F655 | -0.992482106 | 8.46E-05 |
| ata-miR171a-3p_R1-20L21 | Traes_7AL_D69B0DC0B | -0.99245995 | 8.51E-05 |
| tae-miR396-5p_R1-21L21 | Traes_1AS_858D1842B | -0.992459713 | 8.51E-05 |
| tae-miR9659-3p | Traes_2BL_A402261EC1 | -0.992436102 | 8.56E-05 |
| gma-miR5037a_R19-5L22 | Traes_5DL_268762B41 | -0.992422345 | 8.59E-05 |
| smo-miR159_R2-21L21 | TRAES3BF075200060CFD_g | -0.992421329 | 8.59E-05 |
| tae-m1832-5p | TRAES3BF075200060CFD_g | -0.992421329 | 8.59E-05 |
| tae-m2038-5p | TRAES3BF075200060CFD_g | -0.992421329 | 8.59E-05 |
| tae-m3208-5p | TRAES3BF075200060CFD_g | -0.992421329 | 8.59E-05 |
| ppt-miR894_R1-17L20 | Traes_7DL_F631D2D2E | -0.992416108 | 8.61E-05 |
| tae-miR396-5p_R1-21L21 | Traes_4DL_CDDD5E13E | -0.992414897 | 8.61E-05 |
| tae-m3625-3p | Traes_2BS_1B3E61DE0 | -0.992408111 | 8.62E-05 |
| osa-miR5072_R6-20L22 | Traes_3AL_294877B24 | -0.992407939 | 8.62E-05 |
| gra-miR7505b_R6-20L21 | Traes_6AL_920563984 | -0.992403489 | 8.63E-05 |
| mml-miR-892c-3p_R1-17L22 | Traes_6AL_920563984 | -0.992403489 | 8.63E-05 |
| tae-m0403-5p | Traes_6AL_920563984 | -0.992403489 | 8.63E-05 |
| tae-m2665-5p | Traes_6AL_920563984 | -0.992403489 | 8.63E-05 |
| tae-m2876-5p | Traes_6AL_920563984 | -0.992403489 | 8.63E-05 |
| tae-m4081-5p | Traes_6AL_920563984 | -0.992403489 | 8.63E-05 |
| gga-miR-7475-5p_R20-6L20 | Traes_6AL_0361DD7E9 | -0.99240004 | 8.64E-05 |
| tae-miR9659-3p | Traes_5AL_629EDDFC3 | -0.992386217 | 8.67E-05 |
| gga-miR-7475-5p_R20-6L20 | Traes_5AL_099F2FB96 | -0.992344968 | 8.77E-05 |
| tae-m0298-5p | Traes_3AS_F23A6A83C | -0.992343343 | 8.77E-05 |
| ata-miR167e-5p_R1-21L21 | Traes_2BL_01FC4C993 | -0.992325984 | 8.81E-05 |
| tae-m3157-5p | Traes_2BL_01FC4C993 | -0.992325984 | 8.81E-05 |
| tae-m0025-5p | Traes_5BL_634C3B43F | -0.992315619 | 8.83E-05 |
| tae-m2737-5p | Traes_5BL_634C3B43F | -0.992315619 | 8.83E-05 |
| tch-miR-378a-3p | Traes_3AS_FD018268B | -0.99231295 | 8.84E-05 |
| tch-miR-378a-3p | Traes_1AL_E25202CC2 | -0.99229648 | 8.88E-05 |
| tae-m4432-5p | Traes_4DS_8ED58C477 | -0.992295618 | 8.88E-05 |
| ptr-miR-3937_R1-15L24 | Traes_4DS_BABF96DAF | -0.992293008 | 8.89E-05 |
| tae-miR396-5p_R1-21L21 | Traes_1BL_4AB17FFF4 | -0.992271986 | 8.94E-05 |
| ata-miR9672-3p_R19-2L21 | Traes_1DL_115A0324A | -0.992258834 | 8.97E-05 |
| bdi-miR529-3p_R20-6L21 | Traes_1AL_7262657D3 | -0.992254209 | 8.98E-05 |
| sly-miR5300_R21-6L22 | Traes_1BL_0CB993ADF | -0.99225146 | 8.98E-05 |
| ata-miR9672-3p_R19-2L21 | Traes_4DL_B1C234DED | -0.992240016 | 9.01E-05 |
| oha-miR-99a-5p | Traes_5BL_1D79B1A9B | -0.992238036 | 9.01E-05 |
| ata-miR9674a-5p_R1-18L21 | Traes_7DS_E497B7A1F | -0.992237058 | 9.02E-05 |
| mmu-miR-3535_R7-21L26 | Traes_4DL_469179461 | -0.992224501 | 9.05E-05 |
| bdi-miR529-3p_R20-6L21 | Traes_3AS_4A806BAF2 | -0.992219292 | 9.06E-05 |
| ata-miR167f-5p_17A-G | Traes_4BS_B7AF6872F | -0.992216867 | 9.06E-05 |
| tae-miR1121_R3-21L22 | Traes_4AS_98267C6F6 | -0.992176384 | 9.16E-05 |
| ppy-miR-4451_R1-16L18 | Traes_4AL_7F88C0A92 | -0.992166245 | 9.18E-05 |
| tae-miR9659-3p | Traes_5BL_F0C94BA2D | -0.992154587 | 9.21E-05 |
| tae-m1014-5p | Traes_4DS_39D51FA67 | -0.992149843 | 9.22E-05 |
| gma-miR319q_R1-18L21 | Traes_7DS_4301CA3BC | -0.992136173 | 9.25E-05 |
| bdi-miR529-3p_R20-6L21 | Traes_6AL_055CBC57B | -0.992129807 | 9.27E-05 |
| ptr-miR-3937_R1-15L24 | Traes_1DL_7FD4EA081 | -0.992128894 | 9.27E-05 |
| tae-m1477-5p | Traes_2DS_2CB4C2389 | -0.992128498 | 9.27E-05 |
| ssp-miR444b.2_R15-1L21 | Traes_2AS_EEADD06D6 | -0.992122411 | 9.28E-05 |
| efu-miR-9189e_R24-4L24_8A-C | Traes_4AL_DAEB3EBA5 | -0.99211906 | 9.29E-05 |
| tch-miR-378a-3p | Traes_7AS_04B7C0B3F | -0.992097676 | 9.34E-05 |
| tch-miR-378a-3p | Traes_1DL_291C515F3 | -0.992096322 | 9.35E-05 |
| tae-miR396-5p_R1-21L21 | Traes_3DL_BE597D771 | -0.992094257 | 9.35E-05 |
| gga-miR-7475-5p_R20-6L20 | Traes_7DS_1D44614AE | -0.992091705 | 9.36E-05 |
| tae-m3294-3p | Traes_5BL_97BEC5636 | -0.992072676 | 9.40E-05 |
| tae-miR9659-3p | Traes_7AL_65F481DB9 | -0.992063252 | 9.42E-05 |
| gga-miR-7475-5p_R20-6L20 | Traes_2DL_5436C046D | -0.992045058 | 9.47E-05 |
| ssp-miR444b.2_R15-1L21 | Traes_1DL_A9FBE73D6 | -0.992037248 | 9.49E-05 |
| cbn-miR-7630_R6-21L21 | Traes_4DS_39D51FA67 | -0.992034804 | 9.49E-05 |
| cpa-miR167c | Traes_4DS_39D51FA67 | -0.992034804 | 9.49E-05 |
| ppy-miR-378a_R7-21L21 | Traes_4DS_39D51FA67 | -0.992034804 | 9.49E-05 |
| tae-m0253-5p | Traes_4DS_39D51FA67 | -0.992034804 | 9.49E-05 |
| tae-m0275-5p | Traes_4DS_39D51FA67 | -0.992034804 | 9.49E-05 |
| tae-m1233-5p | Traes_4DS_39D51FA67 | -0.992034804 | 9.49E-05 |
| tae-m2619-5p | Traes_4DS_39D51FA67 | -0.992034804 | 9.49E-05 |
| gga-miR-7475-5p_R20-6L20 | Traes_3DS_094739A97 | -0.992028765 | 9.51E-05 |
| gga-miR-7475-5p_R20-6L20 | Traes_1AL_2F2E5017B | -0.992022701 | 9.52E-05 |
| tae-m3294-3p | Traes_6DL_C26461F78 | -0.992017213 | 9.53E-05 |
| tae-miR9659-3p | Traes_6DS_75C2FBF08 | -0.992013532 | 9.54E-05 |
| tae-m2840-5p | TRAES3BF049800030CFD_g | -0.992010131 | 9.55E-05 |
| ata-miR167e-5p_R1-21L21 | TRAES3BF136200010CFD_g | -0.991996358 | 9.58E-05 |
| tae-m3157-5p | TRAES3BF136200010CFD_g | -0.991996358 | 9.58E-05 |
| tae-m0982-5p | Traes_5BL_1D79B1A9B | -0.991992943 | 9.59E-05 |
| bdi-miR5181d_R7-21L21 | Traes_1AL_4905637FC | -0.991985423 | 9.61E-05 |
| tae-m2748-5p | Traes_2BS_64BD37C41 | -0.991981387 | 9.62E-05 |
| tae-m3294-3p | Traes_4AL_5F552AAAD | -0.991978987 | 9.62E-05 |
| gga-miR-7475-5p_R20-6L20 | Traes_4AL_D25430175 | -0.99197134 | 9.64E-05 |
| tae-miR396-5p_R1-21L21 | Traes_6BS_9B886B116 | -0.991965033 | 9.66E-05 |
| gra-miR7505b_R6-20L21 | Traes_6AS_32423383C | -0.991949288 | 9.70E-05 |
| mml-miR-892c-3p_R1-17L22 | Traes_6AS_32423383C | -0.991949288 | 9.70E-05 |
| tae-m0403-5p | Traes_6AS_32423383C | -0.991949288 | 9.70E-05 |
| tae-m2665-5p | Traes_6AS_32423383C | -0.991949288 | 9.70E-05 |
| tae-m2876-5p | Traes_6AS_32423383C | -0.991949288 | 9.70E-05 |
| tae-m4081-5p | Traes_6AS_32423383C | -0.991949288 | 9.70E-05 |
| ata-miR9672-3p_R19-2L21 | Traes_5AL_6A3F590BC | -0.991946746 | 9.70E-05 |
| ata-miR167e-5p_R1-21L21 | Traes_5BS_F93273CED | -0.991946608 | 9.70E-05 |
| tae-m3157-5p | Traes_5BS_F93273CED | -0.991946608 | 9.70E-05 |
| gra-miR7505b_R6-20L21 | Traes_5AL_8B9C3A5E5 | -0.991939221 | 9.72E-05 |
| mml-miR-892c-3p_R1-17L22 | Traes_5AL_8B9C3A5E5 | -0.991939221 | 9.72E-05 |
| tae-m0403-5p | Traes_5AL_8B9C3A5E5 | -0.991939221 | 9.72E-05 |
| tae-m2665-5p | Traes_5AL_8B9C3A5E5 | -0.991939221 | 9.72E-05 |
| tae-m2876-5p | Traes_5AL_8B9C3A5E5 | -0.991939221 | 9.72E-05 |
| tae-m4081-5p | Traes_5AL_8B9C3A5E5 | -0.991939221 | 9.72E-05 |
| tae-miR396-5p_R1-21L21 | Traes_4AL_8911073AB | -0.991938156 | 9.72E-05 |
| ptr-miR-3937_R1-15L24 | Traes_2DS_5D09437DA | -0.99193803 | 9.72E-05 |
| gra-miR7505b_R6-20L21 | Traes_4BS_9940A14C1 | -0.99193435 | 9.73E-05 |
| mml-miR-892c-3p_R1-17L22 | Traes_4BS_9940A14C1 | -0.99193435 | 9.73E-05 |
| tae-m0403-5p | Traes_4BS_9940A14C1 | -0.99193435 | 9.73E-05 |
| tae-m2665-5p | Traes_4BS_9940A14C1 | -0.99193435 | 9.73E-05 |
| tae-m2876-5p | Traes_4BS_9940A14C1 | -0.99193435 | 9.73E-05 |
| tae-m4081-5p | Traes_4BS_9940A14C1 | -0.99193435 | 9.73E-05 |
| tae-m0298-5p | Traes_4BL_D9458F705 | -0.991930947 | 9.74E-05 |
| gga-miR-7475-5p_R20-6L20 | Traes_6AL_5940122BE | -0.99191714 | 9.77E-05 |
| tae-m3294-3p | Traes_6BL_E9173BD9B | -0.991894988 | 9.83E-05 |
| tae-m1651-5p | Traes_2BL_3A44C99D2 | -0.991888292 | 9.84E-05 |
| tch-miR-378a-3p | Traes_4AS_2CFCD8D62 | -0.991886991 | 9.85E-05 |
| tae-miR9659-3p | Traes_7DL_A321818FB | -0.991886026 | 9.85E-05 |
| tae-m3294-3p | Traes_1DL_CA3D844E5 | -0.991885486 | 9.85E-05 |
| tae-miR396-5p_R1-21L21 | Traes_2AL_04F903351 | -0.991882565 | 9.86E-05 |
| cpa-miR8155_R4-18L19 | Traes_2DS_0791E6C67 | -0.991871989 | 9.88E-05 |
| ptr-miR-3937_R1-15L24 | Traes_4BL_41633B2BB | -0.991858814 | 9.91E-05 |
| tae-miR1121_R3-21L22 | Traes_2DL_1815C4C40 | -0.991852121 | 9.93E-05 |
| tae-miR396-5p_R1-21L21 | Traes_4AL_A494372EB | -0.991843461 | 9.95E-05 |
| tae-m3294-3p | Traes_4DL_8D4180F54 | -0.991800642 | 0.000100569 |
| tae-miR9659-3p | Traes_4DL_3D374FF4C | -0.991797369 | 0.000100649 |
| tae-m2170-5p | Traes_4DL_4A0341E1A | -0.991785225 | 0.000100947 |
| mmu-miR-1187_R7-21L23 | Traes_4DL_4A0341E1A | -0.991785225 | 0.000100947 |
| tae-m2840-5p | Traes_4BS_02C5D7625 | -0.99176335 | 0.000101484 |
| gga-miR-7475-5p_R20-6L20 | Traes_6DL_D02E85399 | -0.991747648 | 0.000101871 |
| osa-miR5072_R6-20L22 | Traes_3DL_37B035E55 | -0.991735306 | 0.000102175 |
| tae-m2905-5p | Traes_5DL_3B0E69498 | -0.991734553 | 0.000102194 |
| tae-m1521-5p | TRAES3BF009500010CFD_g | -0.991701046 | 0.000103023 |
| tae-m2489-5p | TRAES3BF009500010CFD_g | -0.991701046 | 0.000103023 |
| tae-m4416-5p | TRAES3BF009500010CFD_g | -0.991701046 | 0.000103023 |
| ata-miR167e-5p_R1-21L21 | Traes_3DL_416E228F3 | -0.991700932 | 0.000103026 |
| tae-m3157-5p | Traes_3DL_416E228F3 | -0.991700932 | 0.000103026 |
| gga-miR-7475-5p_R20-6L20 | Traes_6AL_8B1EA1513 | -0.991689886 | 0.0001033 |
| tae-m3294-3p | Traes_5BL_62D9B877B | -0.99168022 | 0.00010354 |
| tch-miR-378a-3p | Traes_5AS_EDF185C68 | -0.99167764 | 0.000103604 |
| tae-m2840-5p | Traes_1BL_72EC293D2 | -0.991674397 | 0.000103685 |
| gga-miR-7475-5p_R20-6L20 | Traes_2BL_28A7E0BF8 | -0.991629809 | 0.000104797 |
| zma-miR395l-5p_R21-7L22 | Traes_2AS_F03516AF01 | -0.991625119 | 0.000104914 |
| tae-m3738-5p | Traes_6BS_FFD1AED0D | -0.991618063 | 0.000105091 |
| tae-m0195-5p | TRAES3BF063000030CFD_g | -0.99158783 | 0.000105849 |
| tae-m0982-5p | Traes_5BS_6BC7A44AD | -0.991571079 | 0.000106271 |
| ppt-miR894_R1-17L20 | Traes_2AS_D940B19D1 | -0.991552991 | 0.000106727 |
| tae-miR396-5p_R1-21L21 | Traes_7AL_1784D5698 | -0.991552348 | 0.000106743 |
| smo-miR159_R2-21L21 | Traes_3AL_B3DB454DC | -0.991516633 | 0.000107646 |
| tae-m1832-5p | Traes_3AL_B3DB454DC | -0.991516633 | 0.000107646 |
| tae-m2038-5p | Traes_3AL_B3DB454DC | -0.991516633 | 0.000107646 |
| tae-m3208-5p | Traes_3AL_B3DB454DC | -0.991516633 | 0.000107646 |
| tae-miR9659-3p | Traes_7DL_63118F92C | -0.991497723 | 0.000108126 |
| tae-m3949-5p | Traes_2AS_731C703DE | -0.991493536 | 0.000108232 |
| tch-miR-378a-3p | Traes_5AL_099F2FB96 | -0.991480264 | 0.00010857 |
| bmo-miR-3208_R4-19L22 | Traes_6AL_9F1F4A19A | -0.991477465 | 0.000108641 |
| tae-m1452-5p | Traes_6AL_9F1F4A19A | -0.991477465 | 0.000108641 |
| tae-miR396-5p_R1-21L21 | Traes_7DL_62F01AA40 | -0.991474554 | 0.000108715 |
| bdi-miR529-3p_R20-6L21 | Traes_2DL_B5CA07600 | -0.991459648 | 0.000109095 |
| cpa-miR8155_R4-18L19 | Traes_7DS_62C96AD95 | -0.991455971 | 0.000109189 |
| tae-m0982-5p | Traes_3AL_74A14ACC1 | -0.99142308 | 0.00011003 |
| tch-miR-378a-3p | Traes_2BL_5C9966D42 | -0.991407663 | 0.000110425 |
| tch-miR-378a-3p | Traes_6AL_074141030 | -0.991381206 | 0.000111105 |
| cme-miR156g_R1-18L20 | Traes_6DL_2807D89841 | -0.991373986 | 0.000111291 |
| tae-m2996-5p | Traes_6DL_2807D89841 | -0.991373986 | 0.000111291 |
| tae-miR396-5p_R1-21L21 | Traes_7BL_F0868B391 | -0.991365558 | 0.000111509 |
| tae-m1602-5p | Traes_2BL_7F7F6544F | -0.991364802 | 0.000111528 |
| gga-miR-7475-5p_R20-6L20 | Traes_7DL_175492122 | -0.991347555 | 0.000111973 |
| tch-miR-378a-3p | Traes_4DS_993693839 | -0.991344856 | 0.000112043 |
| ata-miR172b-5p_R1-20L21 | Traes_1AS_82CA6D2F3 | -0.991335435 | 0.000112287 |
| bmo-miR-3302_R19-5L23 | Traes_1AS_82CA6D2F3 | -0.991335435 | 0.000112287 |
| tae-m0631-5p | Traes_1AS_82CA6D2F3 | -0.991335435 | 0.000112287 |
| tae-miR9659-3p | Traes_1AL_1F3A0CD1F | -0.991321584 | 0.000112646 |
| tae-m1602-5p | TRAES3BF118300060CFD_g | -0.991316889 | 0.000112767 |
| tae-m3294-3p | Traes_4BS_62FE27332 | -0.99131573 | 0.000112797 |
| tae-m3294-3p | Traes_1AL_36750809D | -0.991313697 | 0.00011285 |
| stu-miR6027_R6-20L22 | Traes_6AS_A137671B1 | -0.991286287 | 0.000113562 |
| mtr-miR7701-5p_R18-4L21 | Traes_2DL_620E5D504 | -0.991284929 | 0.000113598 |
| gga-miR-7475-5p_R20-6L20 | Traes_1BL_5CD8FB94C | -0.991278871 | 0.000113755 |
| ata-miR167e-5p_R1-21L21 | Traes_7AS_799E72BE7 | -0.991264527 | 0.000114129 |
| tae-m3157-5p | Traes_7AS_799E72BE7 | -0.991264527 | 0.000114129 |
| tae-m3294-3p | Traes_4AS_EC04D9E8A | -0.991264341 | 0.000114134 |
| dpr-miR397_R17-1L21 | Traes_4BS_85666ADAF | -0.991258911 | 0.000114276 |
| tae-m1322-5p | Traes_4BS_85666ADAF | -0.991258911 | 0.000114276 |
| tae-m3641-5p | Traes_4BS_85666ADAF | -0.991258911 | 0.000114276 |
| xtr-miR-428a_R7-22L22 | Traes_4BS_85666ADAF | -0.991258911 | 0.000114276 |
| gga-miR-7475-5p_R20-6L20 | Traes_2DL_33F39DFAA | -0.991254817 | 0.000114383 |
| gra-miR7505b_R6-20L21 | Traes_6AL_9437F730F | -0.991252456 | 0.000114445 |
| mml-miR-892c-3p_R1-17L22 | Traes_6AL_9437F730F | -0.991252456 | 0.000114445 |
| tae-m0403-5p | Traes_6AL_9437F730F | -0.991252456 | 0.000114445 |
| tae-m2876-5p | Traes_6AL_9437F730F | -0.991252456 | 0.000114445 |
| tae-m2665-5p | Traes_6AL_9437F730F | -0.991252456 | 0.000114445 |
| tae-m4081-5p | Traes_6AL_9437F730F | -0.991252456 | 0.000114445 |
| gma-miR5037a_R19-5L22 | Traes_3AL_FAC642E2B | -0.991250232 | 0.000114503 |
| tch-miR-378a-3p | Traes_2AL_A7198751F | -0.991248016 | 0.000114561 |
| dpr-miR397_R17-1L21 | Traes_3DL_416E228F3 | -0.991243396 | 0.000114681 |
| tae-m1322-5p | Traes_3DL_416E228F3 | -0.991243396 | 0.000114681 |
| tae-m3641-5p | Traes_3DL_416E228F3 | -0.991243396 | 0.000114681 |
| xtr-miR-428a_R7-22L22 | Traes_3DL_416E228F3 | -0.991243396 | 0.000114681 |
| dpr-miR397_R17-1L21 | Traes_3AL_0D9B4F64B | -0.99123379 | 0.000114933 |
| tae-m1322-5p | Traes_3AL_0D9B4F64B | -0.99123379 | 0.000114933 |
| tae-m3641-5p | Traes_3AL_0D9B4F64B | -0.99123379 | 0.000114933 |
| xtr-miR-428a_R7-22L22 | Traes_3AL_0D9B4F64B | -0.99123379 | 0.000114933 |
| gga-miR-7475-5p_R20-6L20 | Traes_2DL_77FEB7329 | -0.991226366 | 0.000115127 |
| tae-m0982-5p | Traes_4DS_4D761086B | -0.991222029 | 0.000115241 |
| tae-m3294-3p | Traes_4DS_64893FC941 | -0.991217396 | 0.000115362 |
| tae-m1498-5p | Traes_2AL_7719A0369 | -0.991216457 | 0.000115387 |
| tae-m4236-5p | Traes_2AL_7719A0369 | -0.991216457 | 0.000115387 |
| ata-miR9776-5p_R5-20L21 | Traes_2DL_3816B2E1D | -0.99121209 | 0.000115502 |
| tae-m1651-5p | Traes_2BL_965768E2D | -0.991187293 | 0.000116153 |
| dpr-miR397_R17-1L21 | Traes_4BL_0A8199503 | -0.991183031 | 0.000116266 |
| tae-m1322-5p | Traes_4BL_0A8199503 | -0.991183031 | 0.000116266 |
| tae-m3641-5p | Traes_4BL_0A8199503 | -0.991183031 | 0.000116266 |
| xtr-miR-428a_R7-22L22 | Traes_4BL_0A8199503 | -0.991183031 | 0.000116266 |
| tch-miR-378a-3p | Traes_3AS_1DD0D7B7A | -0.991173051 | 0.000116529 |
| ata-miR167d-5p_R1-22L22 | Traes_7BL_D49CE4E57 | -0.991166923 | 0.00011669 |
| tae-m0298-5p | Traes_4DL_59311B671 | -0.991157278 | 0.000116945 |
| gra-miR7505b_R6-20L21 | Traes_3DL_92D59112E | -0.991155736 | 0.000116986 |
| mml-miR-892c-3p_R1-17L22 | Traes_3DL_92D59112E | -0.991155736 | 0.000116986 |
| tae-m0403-5p | Traes_3DL_92D59112E | -0.991155736 | 0.000116986 |
| tae-m2876-5p | Traes_3DL_92D59112E | -0.991155736 | 0.000116986 |
| tae-m2665-5p | Traes_3DL_92D59112E | -0.991155736 | 0.000116986 |
| tae-m4081-5p | Traes_3DL_92D59112E | -0.991155736 | 0.000116986 |
| cme-miR156g_R1-18L20 | Traes_6BL_95C7F7123 | -0.991155634 | 0.000116988 |
| tae-m2996-5p | Traes_6BL_95C7F7123 | -0.991155634 | 0.000116988 |
| tae-m0195-5p | Traes_7BL_670FFBA7D | -0.991135467 | 0.000117522 |
| ssp-miR444b.2_R15-1L21 | Traes_5DL_7E2053226 | -0.991124865 | 0.000117802 |
| ata-miR167e-5p_R1-21L21 | TRAES3BF105300050CFD_g | -0.991082957 | 0.000118916 |
| tae-m3157-5p | TRAES3BF105300050CFD_g | -0.991082957 | 0.000118916 |
| tae-miR1121_R3-21L22 | Traes_4AS_37F805FF1 | -0.991078286 | 0.00011904 |
| tae-m3294-3p | Traes_2BS_66D0C26F4 | -0.991072054 | 0.000119207 |
| gga-miR-7475-5p_R20-6L20 | Traes_5DS_42A1A9BAA | -0.991069417 | 0.000119277 |
| gga-miR-7475-5p_R20-6L20 | TRAES3BF046300070CFD_g | -0.991068717 | 0.000119296 |
| tae-m0452-5p | Traes_4DL_7276FF6C9 | -0.991068368 | 0.000119305 |
| tae-m1358-5p | Traes_4DL_7276FF6C9 | -0.991068368 | 0.000119305 |
| tch-miR-378a-3p | Traes_6DL_BEDFBC80F | -0.991060289 | 0.00011952 |
| tae-m2840-5p | Traes_1AL_E12BF1698 | -0.991052442 | 0.00011973 |
| tae-m3949-5p | Traes_4BL_985CBED5D | -0.991039913 | 0.000120065 |
| gga-miR-7475-5p_R20-6L20 | Traes_4AL_92F24A5B9 | -0.991033306 | 0.000120242 |
| ata-miR167e-5p_R1-21L21 | Traes_3DL_2BC4CDDAA | -0.991024081 | 0.000120489 |
| tae-m3157-5p | Traes_3DL_2BC4CDDAA | -0.991024081 | 0.000120489 |
| tch-miR-378a-3p | Traes_7BS_DE3B9C449 | -0.990994398 | 0.000121286 |
| tae-m3294-3p | Traes_7DL_175492122 | -0.990987571 | 0.00012147 |
| tae-m3949-5p | Traes_4BS_7CF1AE699 | -0.990978207 | 0.000121722 |
| ata-miR9672-3p_R19-2L21 | Traes_7DS_3F91E7A16 | -0.990957841 | 0.000122271 |
| lus-miR159c_R1-21L21 | Traes_5AL_A8C598914 | -0.990950844 | 0.00012246 |
| tch-miR-378a-3p | Traes_2DL_06741A74F | -0.990937304 | 0.000122827 |
| osa-miR5539b_R1-16L22 | Traes_2AL_305405ECA | -0.990934306 | 0.000122908 |
| tae-miR396-5p_R1-21L21 | Traes_2BL_301EE0DC7 | -0.990929072 | 0.000123049 |
| dpr-miR397_R17-1L21 | TRAES3BF105300050CFD_g | -0.990896313 | 0.000123938 |
| tae-m1322-5p | TRAES3BF105300050CFD_g | -0.990896313 | 0.000123938 |
| tae-m3641-5p | TRAES3BF105300050CFD_g | -0.990896313 | 0.000123938 |
| xtr-miR-428a_R7-22L22 | TRAES3BF105300050CFD_g | -0.990896313 | 0.000123938 |
| gga-miR-7475-5p_R20-6L20 | Traes_1AL_25576148E | -0.990886013 | 0.000124219 |
| tae-m1477-5p | TRAES3BF098200010CFD_g | -0.990875306 | 0.00012451 |
| tae-m0195-5p | Traes_4DL_B0C5E69F7 | -0.990865436 | 0.000124779 |
| tae-miR1120a_R24-4L24 | TRAES3BF115600050CFD_g | -0.990855068 | 0.000125062 |
| tae-m2905-5p | Traes_2AL_22EFC1256 | -0.990844176 | 0.00012536 |
| ata-miR167e-5p_R1-21L21 | TRAES3BF073700180CFD_g | -0.990842708 | 0.0001254 |
| tae-m3157-5p | TRAES3BF073700180CFD_g | -0.990842708 | 0.0001254 |
| tae-m0298-5p | Traes_1AS_12E5ECBE2 | -0.990838473 | 0.000125516 |
| tae-miR396-5p_R1-21L21 | Traes_2DS_47717E715 | -0.990819293 | 0.000126041 |
| tch-miR-378a-3p | Traes_2BL_BD8DBA189 | -0.990812433 | 0.000126229 |
| tae-miR396-5p_R1-21L21 | Traes_7DL_40E0AD332 | -0.990804191 | 0.000126456 |
| mmu-miR-3535_R7-21L26 | Traes_1BL_0CB993ADF | -0.990796655 | 0.000126663 |
| mmu-miR-3535_R7-21L26 | Traes_1AL_A69755144 | -0.990795697 | 0.000126689 |
| tae-m3294-3p | Traes_4BS_4BC62B7F7 | -0.990793685 | 0.000126744 |
| tae-miR9659-3p | Traes_7DL_F1F20A901 | -0.990778814 | 0.000127153 |
| tae-miR1121_R3-21L22 | Traes_6DS_BDFEA01CE | -0.990768764 | 0.00012743 |
| tch-miR-378a-3p | Traes_6BL_F4597CA77 | -0.990767185 | 0.000127474 |
| tae-m1918-5p | Traes_4DL_5F7C9AF64 | -0.990764457 | 0.000127549 |
| tae-m2015-5p | Traes_4DL_5F7C9AF64 | -0.990764457 | 0.000127549 |
| tae-m3263-5p | Traes_4DL_5F7C9AF64 | -0.990764457 | 0.000127549 |
| tae-m4217-3p | Traes_4DL_5F7C9AF64 | -0.990764457 | 0.000127549 |
| ata-miR9772b-3p_R1-20L21 | Traes_2AL_748546422 | -0.990754161 | 0.000127833 |
| osa-miR5072_R6-20L22 | Traes_2BL_BEEFE1910 | -0.990741844 | 0.000128173 |
| tae-miR9659-3p | TRAES3BF012700030CFD_g | -0.990740604 | 0.000128208 |
| tae-m1918-5p | Traes_7AL_364A6E96F | -0.990740079 | 0.000128222 |
| tae-m2015-5p | Traes_7AL_364A6E96F | -0.990740079 | 0.000128222 |
| tae-m3263-5p | Traes_7AL_364A6E96F | -0.990740079 | 0.000128222 |
| tae-m4217-3p | Traes_7AL_364A6E96F | -0.990740079 | 0.000128222 |
| gga-miR-7475-5p_R20-6L20 | Traes_5DL_F13C5B5C6 | -0.990732899 | 0.000128421 |
| bdi-miR529-3p_R20-6L21 | Traes_7DS_952325CE0 | -0.990732649 | 0.000128428 |
| ata-miR9672-3p_R19-2L21 | Traes_3DS_DB6B05D3C | -0.990732267 | 0.000128438 |
| tae-miR9659-3p | Traes_7BS_C0604DCF9 | -0.990720668 | 0.00012876 |
| tch-miR-378a-3p | Traes_6DL_909B865C0 | -0.990719847 | 0.000128782 |
| tch-miR-378a-3p | Traes_1DS_E55F00FDB | -0.990705254 | 0.000129187 |
| ata-miR167e-5p_R1-21L21 | Traes_3DL_5585491A2 | -0.990696409 | 0.000129433 |
| tae-m3157-5p | Traes_3DL_5585491A2 | -0.990696409 | 0.000129433 |
| tae-m3738-5p | Traes_5DL_ABB313AE8 | -0.990689013 | 0.000129638 |
| tae-miR396-5p_R1-21L21 | Traes_5AL_68844CE9B | -0.990685243 | 0.000129743 |
| ppt-miR894_R1-17L20 | Traes_4DS_79D16595F | -0.990669621 | 0.000130178 |
| mtr-miR7701-5p_R18-4L21 | Traes_6DL_74673564D | -0.990665297 | 0.000130298 |
| tae-m2905-5p | Traes_2DS_6973E2FF5 | -0.9906635 | 0.000130348 |
| lus-miR159c_R1-21L21 | Traes_7DS_E497B7A1F | -0.990663408 | 0.000130351 |
| gma-miR6300_R3-18L18 | Traes_5BL_ECB922F43 | -0.990652305 | 0.000130661 |
| gga-miR-7475-5p_R20-6L20 | Traes_5AL_55CFC4B56 | -0.990650714 | 0.000130705 |
| gra-miR7505b_R6-20L21 | Traes_4BS_A229FBF7E | -0.990646307 | 0.000130828 |
| mml-miR-892c-3p_R1-17L22 | Traes_4BS_A229FBF7E | -0.990646307 | 0.000130828 |
| tae-m0403-5p | Traes_4BS_A229FBF7E | -0.990646307 | 0.000130828 |
| tae-m2665-5p | Traes_4BS_A229FBF7E | -0.990646307 | 0.000130828 |
| tae-m2876-5p | Traes_4BS_A229FBF7E | -0.990646307 | 0.000130828 |
| tae-m4081-5p | Traes_4BS_A229FBF7E | -0.990646307 | 0.000130828 |
| tae-m4103-5p | Traes_6BS_59D86764B | -0.990644177 | 0.000130888 |
| bta-miR-2285e_R5-19L22 | Traes_3AL_01B03DD4F | -0.990639955 | 0.000131006 |
| stu-miR6027_R6-20L22 | Traes_3AL_4ACBEDEF9 | -0.990633089 | 0.000131198 |
| gga-miR-7475-5p_R20-6L20 | Traes_4AL_92708BAA0 | -0.990609637 | 0.000131854 |
| bdi-miR529-3p_R20-6L21 | TRAES3BF128500150CFD_g | -0.990589725 | 0.000132413 |
| tae-m3949-5p | Traes_1BL_C955569B8 | -0.990588315 | 0.000132453 |
| ssp-miR444b.2_R15-1L21 | Traes_1AL_5EEF86979 | -0.990576739 | 0.000132778 |
| gra-miR7505b_R6-20L21 | Traes_5AL_19DBD0B75 | -0.990567904 | 0.000133027 |
| mml-miR-892c-3p_R1-17L22 | Traes_5AL_19DBD0B75 | -0.990567904 | 0.000133027 |
| tae-m0403-5p | Traes_5AL_19DBD0B75 | -0.990567904 | 0.000133027 |
| tae-m2665-5p | Traes_5AL_19DBD0B75 | -0.990567904 | 0.000133027 |
| tae-m2876-5p | Traes_5AL_19DBD0B75 | -0.990567904 | 0.000133027 |
| tae-m4081-5p | Traes_5AL_19DBD0B75 | -0.990567904 | 0.000133027 |
| tae-m3294-3p | Traes_2DS_326E97FCE | -0.990535938 | 0.000133929 |
| gra-miR7505b_R6-20L21 | Traes_4AS_4C25DF6F4 | -0.990529985 | 0.000134097 |
| mml-miR-892c-3p_R1-17L22 | Traes_4AS_4C25DF6F4 | -0.990529985 | 0.000134097 |
| tae-m0403-5p | Traes_4AS_4C25DF6F4 | -0.990529985 | 0.000134097 |
| tae-m2665-5p | Traes_4AS_4C25DF6F4 | -0.990529985 | 0.000134097 |
| tae-m2876-5p | Traes_4AS_4C25DF6F4 | -0.990529985 | 0.000134097 |
| tae-m4081-5p | Traes_4AS_4C25DF6F4 | -0.990529985 | 0.000134097 |
| tae-miR9659-3p | Traes_3AS_0C477EE8D | -0.990529383 | 0.000134114 |
| tae-m2840-5p | Traes_6AS_A883BC2B1 | -0.99052562 | 0.000134221 |
| bdi-miR529-3p_R20-6L21 | Traes_5DL_55F9B086B | -0.99051948 | 0.000134394 |
| tae-m2905-5p | Traes_7DS_D0D8AA00F | -0.990509167 | 0.000134686 |
| dpr-miR397_R17-1L21 | Traes_5BL_AE6D54F4B | -0.990508391 | 0.000134708 |
| tae-m1322-5p | Traes_5BL_AE6D54F4B | -0.990508391 | 0.000134708 |
| tae-m3641-5p | Traes_5BL_AE6D54F4B | -0.990508391 | 0.000134708 |
| xtr-miR-428a_R7-22L22 | Traes_5BL_AE6D54F4B | -0.990508391 | 0.000134708 |
| tae-m2840-5p | Traes_7DS_CABE66833 | -0.990504579 | 0.000134816 |
| ppy-miR-608_R7-21L25 | Traes_6AL_1C2856D4F | -0.990502397 | 0.000134878 |
| tae-m2905-5p | Traes_1AL_57AE37835 | -0.99047583 | 0.000135633 |
| bta-miR-2429_R19-5L20 | Traes_7DS_2565207CB | -0.990442985 | 0.000136568 |
| nta-miR156f | Traes_7DS_2565207CB | -0.990442985 | 0.000136568 |
| tae-m4030-5p | Traes_7DS_2565207CB | -0.990442985 | 0.000136568 |
| tae-m4349-5p | Traes_7DS_2565207CB | -0.990442985 | 0.000136568 |
| tae-miR1121_R3-21L22 | Traes_4AL_2A6C6F593 | -0.99042985 | 0.000136943 |
| tae-m3709-5p | Traes_7BS_2EF473B22 | -0.990429846 | 0.000136944 |
| tae-m3625-3p | Traes_6BL_0A6DEA6FB | -0.990395029 | 0.00013794 |
| bdi-miR529-3p_R20-6L21 | Traes_3DS_2B09B3362 | -0.990393823 | 0.000137975 |
| tae-m0025-5p | Traes_2AL_748546422 | -0.990389488 | 0.000138099 |
| tae-m2737-5p | Traes_2AL_748546422 | -0.990389488 | 0.000138099 |
| cme-miR156g_R1-18L20 | Traes_5BL_F7E6E35B8 | -0.990388796 | 0.000138119 |
| tae-m2996-5p | Traes_5BL_F7E6E35B8 | -0.990388796 | 0.000138119 |
| tae-m2660-5p | Traes_2DS_9447ACFA4 | -0.990369344 | 0.000138678 |
| tch-miR-378a-3p | Traes_5BL_97BEC5636 | -0.990356442 | 0.000139049 |
| tae-miR9659-3p | Traes_1BS_1E67F5AE2 | -0.99035416 | 0.000139115 |
| tch-miR-378a-3p | Traes_7DL_090D0E08E | -0.990343952 | 0.000139409 |
| mmu-miR-3535_R7-21L26 | Traes_7BS_1E97A291D | -0.990343413 | 0.000139424 |
| tch-miR-378a-3p | Traes_4AL_A73AD2DAD | -0.990327538 | 0.000139882 |
| tae-m0169-5p | Traes_7BS_8B18BE79B | -0.990325762 | 0.000139934 |
| tae-m4429-5p | Traes_1AS_4A9E433A7 | -0.990325681 | 0.000139936 |
| tch-miR-378a-3p | Traes_2BL_34819D129 | -0.990316613 | 0.000140198 |
| tae-miR9659-3p | Traes_5BL_99AB709F2 | -0.990289442 | 0.000140985 |
| tae-miR9659-3p | Traes_5AL_ACB202E80 | -0.990289367 | 0.000140987 |
| tch-miR-378a-3p | Traes_7DL_AAD0A273B1 | -0.990286209 | 0.000141078 |
| tae-m3294-3p | Traes_2DL_6CD064E13 | -0.990282773 | 0.000141178 |
| gga-miR-7475-5p_R20-6L20 | Traes_6DL_EAF038D23 | -0.990265828 | 0.00014167 |
| ata-miR167e-5p_R1-21L21 | Traes_4BL_E2E2C4E1D | -0.990249381 | 0.000142148 |
| tae-m3157-5p | Traes_4BL_E2E2C4E1D | -0.990249381 | 0.000142148 |
| ata-miR167e-5p_R1-21L21 | Traes_4BL_E43C1BB11 | -0.99023026 | 0.000142705 |
| tae-m3157-5p | Traes_4BL_E43C1BB11 | -0.99023026 | 0.000142705 |
| tae-m0169-5p | Traes_4BS_0B76BC2BB | -0.990221722 | 0.000142955 |
| tae-m3294-3p | TRAES3BF051600120CFD_g | -0.990221047 | 0.000142974 |
| ata-miR167d-5p_R1-22L22 | Traes_1BL_682638D5F | -0.990220098 | 0.000143002 |
| ata-miR171a-3p_R1-20L21 | Traes_2BL_0ED5DCF4B | -0.990210858 | 0.000143272 |
| tae-m0431-5p | Traes_2BS_736EF207B1 | -0.990209406 | 0.000143314 |
| cpa-miR8155_R4-18L19 | Traes_5BS_673FCF599 | -0.990203873 | 0.000143476 |
| tae-m1651-5p | Traes_6BL_A9EFABD8F | -0.990199615 | 0.000143601 |
| tae-miR9659-3p | Traes_7AL_0B358E4B4 | -0.990195086 | 0.000143733 |
| tae-m0298-5p | Traes_7BS_81AD0B25D | -0.990193231 | 0.000143788 |
| gga-miR-7475-5p_R20-6L20 | Traes_1AL_95235FDBD | -0.990192209 | 0.000143817 |
| tch-miR-378a-3p | Traes_7DS_86E0BBC6D | -0.990189257 | 0.000143904 |
| tch-miR-378a-3p | TRAES3BF051600120CFD_g | -0.990189098 | 0.000143909 |
| tae-m0982-5p | Traes_2BL_63CB9A0E1 | -0.9901654 | 0.000144603 |
| tae-miR1120a_R24-4L24 | TRAES3BF179600010CFD_g | -0.990163368 | 0.000144663 |
| tae-m2840-5p | Traes_4AL_941C0E3EF | -0.990153381 | 0.000144957 |
| bdi-miR529-3p_R20-6L21 | Traes_3DL_10CCDF363 | -0.990150104 | 0.000145053 |
| tae-m2507-5p | Traes_1BL_156D855B2 | -0.990146819 | 0.000145149 |
| gga-miR-7475-5p_R20-6L20 | Traes_7AS_779E8A5D2 | -0.990143184 | 0.000145256 |
| ata-miR167e-5p_R1-21L21 | Traes_4BS_5222C50FC | -0.990141594 | 0.000145303 |
| tae-m3157-5p | Traes_4BS_5222C50FC | -0.990141594 | 0.000145303 |
| tae-m1651-5p | Traes_6DL_EB6020454 | -0.990119098 | 0.000145966 |
| tae-m1477-5p | Traes_5AL_6A3F590BC | -0.990118386 | 0.000145987 |
| tae-m3051-5p | Traes_1DL_C420A4A4E | -0.990108219 | 0.000146287 |
| cme-miR156g_R1-18L20 | Traes_7DL_0B4336C25 | -0.990104536 | 0.000146396 |
| tae-m2996-5p | Traes_7DL_0B4336C25 | -0.990104536 | 0.000146396 |
| tch-miR-378a-3p | Traes_1DL_88DD1E468 | -0.990089596 | 0.000146837 |
| tch-miR-378a-3p | Traes_5DL_F13C5B5C6 | -0.990086309 | 0.000146935 |
| tae-m3294-3p | Traes_1AL_34404D5D8 | -0.990081051 | 0.00014709 |
| tae-m3294-3p | Traes_6BL_F4597CA77 | -0.990071684 | 0.000147368 |
| ssp-miR444b.2_R15-1L21 | Traes_5DL_412D1CEF6 | -0.990068951 | 0.000147449 |
| gra-miR7505b_R6-20L21 | Traes_1DS_EEBA94F4D | -0.990022091 | 0.000148841 |
| mml-miR-892c-3p_R1-17L22 | Traes_1DS_EEBA94F4D | -0.990022091 | 0.000148841 |
| tae-m0403-5p | Traes_1DS_EEBA94F4D | -0.990022091 | 0.000148841 |
| tae-m2665-5p | Traes_1DS_EEBA94F4D | -0.990022091 | 0.000148841 |
| tae-m2876-5p | Traes_1DS_EEBA94F4D | -0.990022091 | 0.000148841 |
| tae-m4081-5p | Traes_1DS_EEBA94F4D | -0.990022091 | 0.000148841 |
| tae-m4103-5p | Traes_1AL_8B97DE3B4 | -0.990015286 | 0.000149044 |
| tae-m3051-5p | Traes_7BL_DF205C0B0 | -0.990011469 | 0.000149158 |
| ppt-miR894_R1-17L20 | Traes_4DL_42EA23191 | -0.990002801 | 0.000149416 |
| tae-m4432-5p | Traes_2DL_02A40DAE4 | 0.990016474 | 0.000149009 |
| tae-m3294-3p | Traes_4BL_15DBF043B | 0.99001973 | 0.000148912 |
| oha-miR-199c-3p_R2-22L22 | Traes_6BS_7F65A4F6C | 0.990027341 | 0.000148685 |
| gga-miR-7475-5p_R20-6L20 | Traes_5AS_51DBC1F49 | 0.990029529 | 0.00014862 |
| bta-miR-2429_R19-5L20 | Traes_2BL_0B80B3876 | 0.990032482 | 0.000148532 |
| nta-miR156f | Traes_2BL_0B80B3876 | 0.990032482 | 0.000148532 |
| tae-m4030-5p | Traes_2BL_0B80B3876 | 0.990032482 | 0.000148532 |
| tae-m4349-5p | Traes_2BL_0B80B3876 | 0.990032482 | 0.000148532 |
| ssp-miR444b.2_R15-1L21 | Traes_2AS_537EEA6B2 | 0.990034618 | 0.000148468 |
| mmu-miR-7665-3p_R8-22L22 | Traes_5DL_017B20CCA | 0.990038507 | 0.000148353 |
| tae-m0431-5p | Traes_7BL_360608806 | 0.990052371 | 0.000147941 |
| tae-m3821-3p | Traes_2BL_CDF259349 | 0.99005265 | 0.000147933 |
| tae-m4368-5p | Traes_2DL_53D5FD18C | 0.990064127 | 0.000147592 |
| gra-miR7505b_R6-20L21 | Traes_2BS_73911552F | 0.990073453 | 0.000147315 |
| mml-miR-892c-3p_R1-17L22 | Traes_2BS_73911552F | 0.990073453 | 0.000147315 |
| tae-m0403-5p | Traes_2BS_73911552F | 0.990073453 | 0.000147315 |
| tae-m2665-5p | Traes_2BS_73911552F | 0.990073453 | 0.000147315 |
| tae-m2876-5p | Traes_2BS_73911552F | 0.990073453 | 0.000147315 |
| tae-m4081-5p | Traes_2BS_73911552F | 0.990073453 | 0.000147315 |
| smo-miR159_R2-21L21 | Traes_7DL_A1D314F33 | 0.990074782 | 0.000147276 |
| tae-m1832-5p | Traes_7DL_A1D314F33 | 0.990074782 | 0.000147276 |
| tae-m2038-5p | Traes_7DL_A1D314F33 | 0.990074782 | 0.000147276 |
| tae-m3208-5p | Traes_7DL_A1D314F33 | 0.990074782 | 0.000147276 |
| oha-miR-199c-3p_R2-22L22 | Traes_3AL_D6C22440B | 0.990110493 | 0.00014622 |
| ata-miR167e-5p_R1-21L21 | Traes_4DL_92D72827C | 0.990121802 | 0.000145886 |
| tae-m3157-5p | Traes_4DL_92D72827C | 0.990121802 | 0.000145886 |
| bdi-miR390a-3p | Traes_1BL_D6EDAAEAC | 0.990123955 | 0.000145823 |
| bdi-miR1127_R18-4L21 | Traes_2AL_5D9E0A761 | 0.990124602 | 0.000145804 |
| tae-m4432-5p | Traes_6DS_536814F70 | 0.990125408 | 0.00014578 |
| hsa-miR-4684-3p_R8-22L22 | Traes_4BL_762F09E09 | 0.990128084 | 0.000145701 |
| tae-m0195-5p | Traes_6AL_FD7E876FF | 0.990132552 | 0.000145569 |
| bta-miR-2429_R19-5L20 | Traes_5AL_6284FBBD8 | 0.990134326 | 0.000145517 |
| nta-miR156f | Traes_5AL_6284FBBD8 | 0.990134326 | 0.000145517 |
| tae-m4030-5p | Traes_5AL_6284FBBD8 | 0.990134326 | 0.000145517 |
| tae-m4349-5p | Traes_5AL_6284FBBD8 | 0.990134326 | 0.000145517 |
| mmu-miR-5108_R1-16L19 | Traes_2DS_E35B3BFB4 | 0.990137829 | 0.000145414 |
| tae-m3239-5p | Traes_2DS_E35B3BFB4 | 0.990137829 | 0.000145414 |
| tae-m3294-3p | Traes_7DL_98EC3AE06 | 0.990139492 | 0.000145365 |
| mtr-miR7701-5p_R18-4L21 | Traes_3AS_E23913975 | 0.990142778 | 0.000145268 |
| mmu-miR-5627-5p_R18-4L22 | Traes_2BL_FCAE67A28 | 0.990164556 | 0.000144628 |
| tae-m2905-5p | Traes_2BL_E507901E4 | 0.990166129 | 0.000144582 |
| gma-miR5037a_R19-5L22 | Traes_3AL_A379BDDC5 | 0.990168683 | 0.000144507 |
| ata-miR172b-5p_R1-20L21 | Traes_2DS_6973E2FF5 | 0.990172171 | 0.000144405 |
| bmo-miR-3302_R19-5L23 | Traes_2DS_6973E2FF5 | 0.990172171 | 0.000144405 |
| tae-m0631-5p | Traes_2DS_6973E2FF5 | 0.990172171 | 0.000144405 |
| ata-miR172b-5p_R1-20L21 | Traes_3AS_8A727B48F | 0.990172399 | 0.000144398 |
| bmo-miR-3302_R19-5L23 | Traes_3AS_8A727B48F | 0.990172399 | 0.000144398 |
| tae-m0631-5p | Traes_3AS_8A727B48F | 0.990172399 | 0.000144398 |
| tae-m0488-5p | Traes_5AL_A37BD65AB | 0.99017779 | 0.00014424 |
| tae-m4293-5p | Traes_5AL_A37BD65AB | 0.99017779 | 0.00014424 |
| gma-miR5037a_R19-5L22 | Traes_5BL_0A654A16E | 0.990184426 | 0.000144045 |
| efu-miR-9189d_R3-17L23 | Traes_4BL_04956D80D | 0.990186682 | 0.000143979 |
| tae-m1456-5p | Traes_7AL_649E6547F | 0.990200309 | 0.00014358 |
| tae-m2333-5p | Traes_5DS_C53497323 | 0.990204541 | 0.000143457 |
| tae-m3808-5p | Traes_5DS_C53497323 | 0.990204541 | 0.000143457 |
| tch-miR-378a-3p | Traes_7BL_D1311836E | 0.990209419 | 0.000143314 |
| smo-miR159_R2-21L21 | Traes_5BL_8512C24F7 | 0.990215601 | 0.000143133 |
| tae-m1832-5p | Traes_5BL_8512C24F7 | 0.990215601 | 0.000143133 |
| tae-m2038-5p | Traes_5BL_8512C24F7 | 0.990215601 | 0.000143133 |
| tae-m3208-5p | Traes_5BL_8512C24F7 | 0.990215601 | 0.000143133 |
| tae-miR9659-3p | Traes_4BS_F395A220A | 0.990219938 | 0.000143007 |
| efu-miR-9189d_R3-17L23 | Traes_5DL_62CE05147 | 0.99022059 | 0.000142988 |
| tae-m0982-5p | Traes_6AS_621A7A571 | 0.990225268 | 0.000142851 |
| tch-miR-378a-3p | Traes_5AS_9689E345A | 0.990226923 | 0.000142803 |
| tae-m4432-5p | Traes_4BS_B2497F675 | 0.990228148 | 0.000142767 |
| ssp-miR444b.2_R15-1L21 | Traes_6BS_6D2A626DE | 0.990229933 | 0.000142715 |
| tae-m4429-5p | Traes_3AS_6BC049834 | 0.990260858 | 0.000141814 |
| smo-miR159_R2-21L21 | Traes_2AS_EEADD06D6 | 0.990274418 | 0.00014142 |
| tae-m1832-5p | Traes_2AS_EEADD06D6 | 0.990274418 | 0.00014142 |
| tae-m2038-5p | Traes_2AS_EEADD06D6 | 0.990274418 | 0.00014142 |
| tae-m3208-5p | Traes_2AS_EEADD06D6 | 0.990274418 | 0.00014142 |
| tae-m0982-5p | Traes_6BL_2B7EC4BEA | 0.990275588 | 0.000141386 |
| bbe-miR-4857-5p_R3-17L23 | Traes_4DL_DC6A55EEE | 0.990281735 | 0.000141208 |
| hsa-miR-3677-5p_R17-3L22 | Traes_4DL_DC6A55EEE | 0.990281735 | 0.000141208 |
| ata-miR167e-5p_R1-21L21 | Traes_4BS_2BDEF5A53 | 0.990282924 | 0.000141174 |
| tae-m3157-5p | Traes_4BS_2BDEF5A53 | 0.990282924 | 0.000141174 |
| bdi-miR529-3p_R20-6L21 | Traes_6AS_A86274C4F | 0.990286591 | 0.000141067 |
| tae-m0982-5p | Traes_2BL_84B12F4F8 | 0.990287022 | 0.000141055 |
| tae-m2665-5p | Traes_3AS_FD018268B | 0.990288787 | 0.000141004 |
| tae-m4081-5p | Traes_3AS_FD018268B | 0.990288787 | 0.000141004 |
| gra-miR7505b_R6-20L21 | Traes_3AS_FD018268B | 0.990288787 | 0.000141004 |
| mml-miR-892c-3p_R1-17L22 | Traes_3AS_FD018268B | 0.990288787 | 0.000141004 |
| tae-m0403-5p | Traes_3AS_FD018268B | 0.990288787 | 0.000141004 |
| tae-m2876-5p | Traes_3AS_FD018268B | 0.990288787 | 0.000141004 |
| oha-miR-99a-5p | Traes_2BS_24B01ABC4 | 0.990292721 | 0.00014089 |
| hvu-miR6209_R15-1L20 | TRAES3BF045900020CFD_g | 0.990293249 | 0.000140874 |
| tae-m0561-5p | Traes_6AL_E54708BB8 | 0.990298303 | 0.000140728 |
| gra-miR7505b_R6-20L21 | Traes_1DL_CA3D844E5 | 0.990304866 | 0.000140538 |
| mml-miR-892c-3p_R1-17L22 | Traes_1DL_CA3D844E5 | 0.990304866 | 0.000140538 |
| tae-m0403-5p | Traes_1DL_CA3D844E5 | 0.990304866 | 0.000140538 |
| tae-m2665-5p | Traes_1DL_CA3D844E5 | 0.990304866 | 0.000140538 |
| tae-m2876-5p | Traes_1DL_CA3D844E5 | 0.990304866 | 0.000140538 |
| tae-m4081-5p | Traes_1DL_CA3D844E5 | 0.990304866 | 0.000140538 |
| smo-miR159_R2-21L21 | Traes_1AL_D7BDD58BD | 0.990305095 | 0.000140531 |
| tae-m1832-5p | Traes_1AL_D7BDD58BD | 0.990305095 | 0.000140531 |
| tae-m2038-5p | Traes_1AL_D7BDD58BD | 0.990305095 | 0.000140531 |
| tae-m3208-5p | Traes_1AL_D7BDD58BD | 0.990305095 | 0.000140531 |
| gra-miR7505b_R6-20L21 | Traes_2DL_77FEB7329 | 0.99030738 | 0.000140465 |
| mml-miR-892c-3p_R1-17L22 | Traes_2DL_77FEB7329 | 0.99030738 | 0.000140465 |
| tae-m0403-5p | Traes_2DL_77FEB7329 | 0.99030738 | 0.000140465 |
| tae-m2665-5p | Traes_2DL_77FEB7329 | 0.99030738 | 0.000140465 |
| tae-m2876-5p | Traes_2DL_77FEB7329 | 0.99030738 | 0.000140465 |
| tae-m4081-5p | Traes_2DL_77FEB7329 | 0.99030738 | 0.000140465 |
| tae-m3821-3p | Traes_3AL_729C392FB | 0.990310057 | 0.000140388 |
| dpr-miR397_R17-1L21 | TRAES3BF031600050CFD_g | 0.990310234 | 0.000140382 |
| tae-m1322-5p | TRAES3BF031600050CFD_g | 0.990310234 | 0.000140382 |
| tae-m3641-5p | TRAES3BF031600050CFD_g | 0.990310234 | 0.000140382 |
| xtr-miR-428a_R7-22L22 | TRAES3BF031600050CFD_g | 0.990310234 | 0.000140382 |
| mmu-miR-1187_R7-21L23 | Traes_5AL_BCB2F438B | 0.990310339 | 0.000140379 |
| tae-m2170-5p | Traes_5AL_BCB2F438B | 0.990310339 | 0.000140379 |
| oha-miR-99a-5p | TRAES3BF272100030CFD_g | 0.990315912 | 0.000140218 |
| bbe-miR-4857-5p_R3-17L23 | Traes_1BL_BD721F2E3 | 0.990322489 | 0.000140028 |
| hsa-miR-3677-5p_R17-3L22 | Traes_1BL_BD721F2E3 | 0.990322489 | 0.000140028 |
| tae-m2840-5p | Traes_7AS_A94491411 | 0.990323406 | 0.000140002 |
| gra-miR7505b_R6-20L21 | Traes_6BL_A3E9C02F0 | 0.990324957 | 0.000139957 |
| mml-miR-892c-3p_R1-17L22 | Traes_6BL_A3E9C02F0 | 0.990324957 | 0.000139957 |
| tae-m0403-5p | Traes_6BL_A3E9C02F0 | 0.990324957 | 0.000139957 |
| tae-m2665-5p | Traes_6BL_A3E9C02F0 | 0.990324957 | 0.000139957 |
| tae-m2876-5p | Traes_6BL_A3E9C02F0 | 0.990324957 | 0.000139957 |
| tae-m4081-5p | Traes_6BL_A3E9C02F0 | 0.990324957 | 0.000139957 |
| ssp-miR444b.2_R15-1L21 | Traes_7BS_C0604DCF9 | 0.990332289 | 0.000139745 |
| smo-miR159_R2-21L21 | Traes_2BL_08FC042C0 | 0.990354366 | 0.000139109 |
| tae-m1832-5p | Traes_2BL_08FC042C0 | 0.990354366 | 0.000139109 |
| tae-m2038-5p | Traes_2BL_08FC042C0 | 0.990354366 | 0.000139109 |
| tae-m3208-5p | Traes_2BL_08FC042C0 | 0.990354366 | 0.000139109 |
| tae-m3294-3p | Traes_6AS_976D36CDD | 0.990355598 | 0.000139073 |
| bta-miR-760-5p_R17-3L22 | Traes_4DS_829B5AEE9 | 0.990355739 | 0.000139069 |
| cbr-miR-35a_R4-18L22 | Traes_4DS_829B5AEE9 | 0.990355739 | 0.000139069 |
| tae-m4294-5p | Traes_4DS_829B5AEE9 | 0.990355739 | 0.000139069 |
| mmu-miR-7657-5p_R5-19L21 | Traes_1DS_08DB46EF9 | 0.990357541 | 0.000139017 |
| tae-m0295-5p | Traes_5BL_CAA25FDD4 | 0.990364934 | 0.000138805 |
| gga-miR-7475-5p_R20-6L20 | Traes_5BL_1CF10603C | 0.990365622 | 0.000138785 |
| oha-miR-22a_R1-19L22 | Traes_1BS_36169E4A1 | 0.990367027 | 0.000138744 |
| gra-miR7505b_R6-20L21 | Traes_7AS_A94491411 | 0.990369045 | 0.000138686 |
| mml-miR-892c-3p_R1-17L22 | Traes_7AS_A94491411 | 0.990369045 | 0.000138686 |
| tae-m0403-5p | Traes_7AS_A94491411 | 0.990369045 | 0.000138686 |
| tae-m2665-5p | Traes_7AS_A94491411 | 0.990369045 | 0.000138686 |
| tae-m2876-5p | Traes_7AS_A94491411 | 0.990369045 | 0.000138686 |
| tae-m4081-5p | Traes_7AS_A94491411 | 0.990369045 | 0.000138686 |
| bdi-miR5198_R1-15L21 | Traes_2DS_99538A3AE | 0.990370499 | 0.000138644 |
| oha-miR-30e-5p_R1-24L25 | Traes_2DS_99538A3AE | 0.990370499 | 0.000138644 |
| ata-miR167e-5p_R1-21L21 | Traes_5DL_412D1CEF6 | 0.990377334 | 0.000138448 |
| tae-m3157-5p | Traes_5DL_412D1CEF6 | 0.990377334 | 0.000138448 |
| bta-miR-760-5p_R17-3L22 | Traes_2AS_3161D54F8 | 0.990377725 | 0.000138437 |
| cbr-miR-35a_R4-18L22 | Traes_2AS_3161D54F8 | 0.990377725 | 0.000138437 |
| tae-m4294-5p | Traes_2AS_3161D54F8 | 0.990377725 | 0.000138437 |
| cme-miR156g_R1-18L20 | TRAES3BF072400290CFD_g | 0.990379523 | 0.000138385 |
| tae-m2996-5p | TRAES3BF072400290CFD_g | 0.990379523 | 0.000138385 |
| tch-miR-378a-3p | Traes_5DL_834E4B05E | 0.990387048 | 0.000138169 |
| tae-m2333-5p | Traes_4BS_7700FC74D | 0.990404481 | 0.000137669 |
| tae-m3808-5p | Traes_4BS_7700FC74D | 0.990404481 | 0.000137669 |
| tae-m2333-5p | Traes_6DS_53B15C3C4 | 0.99041273 | 0.000137433 |
| tae-m3808-5p | Traes_6DS_53B15C3C4 | 0.99041273 | 0.000137433 |
| osa-miR5539b_R1-16L22 | Traes_4DL_8ACB43847 | 0.99041542 | 0.000137356 |
| tae-m0044-5p | Traes_2DL_B5CA07600 | 0.990419609 | 0.000137236 |
| tae-m1918-5p | Traes_6BS_85AA7A67F | 0.990438342 | 0.000136701 |
| tae-m2015-5p | Traes_6BS_85AA7A67F | 0.990438342 | 0.000136701 |
| tae-m3263-5p | Traes_6BS_85AA7A67F | 0.990438342 | 0.000136701 |
| tae-m4217-3p | Traes_6BS_85AA7A67F | 0.990438342 | 0.000136701 |
| bdi-miR5198_R1-15L21 | Traes_2DL_4CFD341D6 | 0.990438523 | 0.000136696 |
| oha-miR-30e-5p_R1-24L25 | Traes_2DL_4CFD341D6 | 0.990438523 | 0.000136696 |
| oha-miR-99a-5p | Traes_4AL_9E250501B | 0.990453798 | 0.00013626 |
| zma-miR395l-5p_R21-7L22 | Traes_1BS_DD55B7D8F | 0.99045614 | 0.000136193 |
| gga-miR-7475-5p_R20-6L20 | Traes_2AS_71B3134F6 | 0.990456833 | 0.000136173 |
| ppy-miR-4451_R1-16L18 | Traes_5DS_64C30A803 | 0.990458481 | 0.000136127 |
| tae-m0298-5p | Traes_4BS_1687E4DC6 | 0.990459269 | 0.000136104 |
| oha-miR-99a-5p | TRAES3BF116200290CFD_g | 0.990459796 | 0.000136089 |
| bbe-miR-4857-5p_R3-17L23 | Traes_4AL_1D4A5919A1 | 0.990460041 | 0.000136082 |
| hsa-miR-3677-5p_R17-3L22 | Traes_4AL_1D4A5919A1 | 0.990460041 | 0.000136082 |
| gma-miR5037a_R19-5L22 | Traes_7AS_6EA67C265 | 0.990463854 | 0.000135974 |
| tae-m1521-5p | Traes_7AS_B0AA62C31 | 0.990468319 | 0.000135846 |
| tae-m2489-5p | Traes_7AS_B0AA62C31 | 0.990468319 | 0.000135846 |
| tae-m4416-5p | Traes_7AS_B0AA62C31 | 0.990468319 | 0.000135846 |
| tae-m3312-5p | Traes_5AL_99BEC1C3B | 0.9904773 | 0.000135591 |
| tae-m1918-5p | Traes_1AL_0BDE975B6 | 0.99047756 | 0.000135584 |
| tae-m2015-5p | Traes_1AL_0BDE975B6 | 0.99047756 | 0.000135584 |
| tae-m3263-5p | Traes_1AL_0BDE975B6 | 0.99047756 | 0.000135584 |
| tae-m4217-3p | Traes_1AL_0BDE975B6 | 0.99047756 | 0.000135584 |
| tae-m0147-5p | Traes_4DL_DE24CB90D | 0.99047793 | 0.000135573 |
| tae-m3576-5p | Traes_4DL_DE24CB90D | 0.99047793 | 0.000135573 |
| tae-m3683-5p | Traes_4DL_DE24CB90D | 0.99047793 | 0.000135573 |
| ssp-miR444b.2_R15-1L21 | TRAES3BF175100090CFD_g | 0.990478439 | 0.000135559 |
| tch-miR-378a-3p | Traes_3DL_2BC4E4FEB | 0.990479475 | 0.000135529 |
| tch-miR-378a-3p | Traes_2DS_85E22FAC3 | 0.990480453 | 0.000135501 |
| tae-m0982-5p | Traes_1AL_1D02EEFB9 | 0.990497408 | 0.00013502 |
| dpr-miR397_R17-1L21 | Traes_5BS_D0C6E034C | 0.990498848 | 0.000134979 |
| tae-m1322-5p | Traes_5BS_D0C6E034C | 0.990498848 | 0.000134979 |
| tae-m3641-5p | Traes_5BS_D0C6E034C | 0.990498848 | 0.000134979 |
| xtr-miR-428a_R7-22L22 | Traes_5BS_D0C6E034C | 0.990498848 | 0.000134979 |
| tae-m0147-5p | Traes_7DS_1690B1EC2 | 0.990503187 | 0.000134856 |
| tae-m3576-5p | Traes_7DS_1690B1EC2 | 0.990503187 | 0.000134856 |
| tae-m3683-5p | Traes_7DS_1690B1EC2 | 0.990503187 | 0.000134856 |
| bbe-miR-4857-5p_R3-17L23 | Traes_5BL_9DCE48BD2 | 0.990519717 | 0.000134388 |
| hsa-miR-3677-5p_R17-3L22 | Traes_5BL_9DCE48BD2 | 0.990519717 | 0.000134388 |
| tae-m0982-5p | Traes_7AL_4EFFD9BDF | 0.990521402 | 0.00013434 |
| mmu-miR-5627-5p_R18-4L22 | Traes_5BL_294A23904 | 0.990523188 | 0.000134289 |
| gga-miR-7475-5p_R20-6L20 | Traes_7DL_88DE10A78 | 0.990523657 | 0.000134276 |
| mmu-miR-5106_R2-19L23 | Traes_5DS_3D0E0BC00 | 0.990533832 | 0.000133988 |
| tae-m4432-5p | Traes_7AL_B4329E36B | 0.990533892 | 0.000133987 |
| bta-miR-2429_R19-5L20 | Traes_6AS_7B7ECDCB3 | 0.990535971 | 0.000133928 |
| nta-miR156f | Traes_6AS_7B7ECDCB3 | 0.990535971 | 0.000133928 |
| tae-m4030-5p | Traes_6AS_7B7ECDCB3 | 0.990535971 | 0.000133928 |
| tae-m4349-5p | Traes_6AS_7B7ECDCB3 | 0.990535971 | 0.000133928 |
| oha-miR-199c-3p_R2-22L22 | Traes_4BL_8A8D56D7B | 0.990537134 | 0.000133895 |
| bta-miR-2429_R19-5L20 | Traes_5DS_581B4DB50 | 0.990540078 | 0.000133812 |
| nta-miR156f | Traes_5DS_581B4DB50 | 0.990540078 | 0.000133812 |
| tae-m4030-5p | Traes_5DS_581B4DB50 | 0.990540078 | 0.000133812 |
| tae-m4349-5p | Traes_5DS_581B4DB50 | 0.990540078 | 0.000133812 |
| tae-m3294-3p | Traes_3DL_2114C4621 | 0.990545332 | 0.000133664 |
| tae-m2333-5p | Traes_7AS_7C000CD93 | 0.990561679 | 0.000133202 |
| tae-m3808-5p | Traes_7AS_7C000CD93 | 0.990561679 | 0.000133202 |
| bta-miR-2429_R19-5L20 | Traes_6DL_3A44079B9 | 0.990574011 | 0.000132855 |
| nta-miR156f | Traes_6DL_3A44079B9 | 0.990574011 | 0.000132855 |
| tae-m4030-5p | Traes_6DL_3A44079B9 | 0.990574011 | 0.000132855 |
| tae-m4349-5p | Traes_6DL_3A44079B9 | 0.990574011 | 0.000132855 |
| tae-m4283-5p | Traes_1AL_3791A0A3A | 0.990575208 | 0.000132821 |
| tae-m3294-3p | Traes_1BL_28DB0E01C | 0.990576253 | 0.000132792 |
| gma-miR5037a_R19-5L22 | Traes_5BL_0B5C3EEC6 | 0.990578415 | 0.000132731 |
| ata-miR172b-5p_R1-20L21 | Traes_4AL_3BA1D51E2 | 0.990580151 | 0.000132682 |
| bmo-miR-3302_R19-5L23 | Traes_4AL_3BA1D51E2 | 0.990580151 | 0.000132682 |
| tae-m0631-5p | Traes_4AL_3BA1D51E2 | 0.990580151 | 0.000132682 |
| gra-miR7505b_R6-20L21 | Traes_1DS_3E8E0BE88 | 0.990585339 | 0.000132537 |
| mml-miR-892c-3p_R1-17L22 | Traes_1DS_3E8E0BE88 | 0.990585339 | 0.000132537 |
| tae-m0403-5p | Traes_1DS_3E8E0BE88 | 0.990585339 | 0.000132537 |
| tae-m2665-5p | Traes_1DS_3E8E0BE88 | 0.990585339 | 0.000132537 |
| tae-m2876-5p | Traes_1DS_3E8E0BE88 | 0.990585339 | 0.000132537 |
| tae-m4081-5p | Traes_1DS_3E8E0BE88 | 0.990585339 | 0.000132537 |
| tae-m0982-5p | Traes_7DL_D03D0D5DD | 0.990586598 | 0.000132501 |
| tae-m3294-3p | Traes_6AL_01DB61EF6 | 0.990587203 | 0.000132484 |
| gma-miR5037a_R19-5L22 | Traes_1BL_3A901257A | 0.990588188 | 0.000132456 |
| tch-miR-378a-3p | Traes_5AL_5A2A6E1FF | 0.990591268 | 0.00013237 |
| tae-m3821-3p | Traes_6DL_13B51D4DC | 0.990593531 | 0.000132306 |
| tae-m3821-3p | Traes_2BS_9A6251B43 | 0.990596024 | 0.000132236 |
| bta-miR-2429_R19-5L20 | Traes_7BS_46E1123CE | 0.990600045 | 0.000132123 |
| nta-miR156f | Traes_7BS_46E1123CE | 0.990600045 | 0.000132123 |
| tae-m4030-5p | Traes_7BS_46E1123CE | 0.990600045 | 0.000132123 |
| tae-m4349-5p | Traes_7BS_46E1123CE | 0.990600045 | 0.000132123 |
| smo-miR159_R2-21L21 | Traes_2BL_5DFE6B3311 | 0.99060633 | 0.000131947 |
| tae-m1832-5p | Traes_2BL_5DFE6B3311 | 0.99060633 | 0.000131947 |
| tae-m2038-5p | Traes_2BL_5DFE6B3311 | 0.99060633 | 0.000131947 |
| tae-m3208-5p | Traes_2BL_5DFE6B3311 | 0.99060633 | 0.000131947 |
| tch-miR-378a-3p | Traes_5DL_69DE08EB5 | 0.990612394 | 0.000131777 |
| oha-miR-199c-3p_R2-22L22 | Traes_7BL_2472DB370 | 0.990615361 | 0.000131694 |
| gga-miR-7475-5p_R20-6L20 | Traes_1DS_EEBA94F4D | 0.990620604 | 0.000131547 |
| mmu-miR-3535_R7-21L26 | Traes_1BS_E72B7E4BF | 0.990631761 | 0.000131235 |
| tae-m3821-3p | Traes_4DL_413A99ECF | 0.990639308 | 0.000131024 |
| gma-miR5677_R15-1L21 | Traes_6DL_B28B5501C | 0.990641565 | 0.000130961 |
| tae-m3603-5p | Traes_6DL_B28B5501C | 0.990641565 | 0.000130961 |
| tae-m3782-5p | Traes_6DL_B28B5501C | 0.990641565 | 0.000130961 |
| oha-miR-22a_R1-19L22 | Traes_7DL_B80093097 | 0.990642883 | 0.000130924 |
| bta-miR-2429_R19-5L20 | Traes_2BL_CDF259349 | 0.990646191 | 0.000130831 |
| nta-miR156f | Traes_2BL_CDF259349 | 0.990646191 | 0.000130831 |
| tae-m4030-5p | Traes_2BL_CDF259349 | 0.990646191 | 0.000130831 |
| tae-m4349-5p | Traes_2BL_CDF259349 | 0.990646191 | 0.000130831 |
| oha-miR-199c-3p_R2-22L22 | Traes_6DL_13B51D4DC | 0.990647229 | 0.000130802 |
| oha-miR-203-3p_R1-20L22 | Traes_1BL_49F8F7A44 | 0.990647239 | 0.000130802 |
| oha-miR-203-3p_R1-20L22 | Traes_1BL_24850CFA6 | 0.990677476 | 0.000129959 |
| ata-miR167e-5p_R1-21L21 | Traes_1DL_122F16FB2 | 0.990679893 | 0.000129892 |
| tae-m3157-5p | Traes_1DL_122F16FB2 | 0.990679893 | 0.000129892 |
| hvu-miR6209_R15-1L20 | Traes_1BL_BD721F2E3 | 0.990681543 | 0.000129846 |
| oha-miR-199c-3p_R2-22L22 | Traes_5DL_2D0D83A55 | 0.990682066 | 0.000129831 |
| hvu-miR6209_R15-1L20 | Traes_3AS_B09E8B029 | 0.990688357 | 0.000129656 |
| tae-m2161-3p | Traes_2BS_45CD5B99E | 0.99070241 | 0.000129266 |
| tae-m3294-3p | Traes_7DL_63118F92C | 0.990705684 | 0.000129175 |
| ata-miR167e-5p_R1-21L21 | Traes_2AS_BF5FA8832 | 0.990707123 | 0.000129135 |
| tae-m3157-5p | Traes_2AS_BF5FA8832 | 0.990707123 | 0.000129135 |
| tch-miR-378a-3p | Traes_6AS_D49C93E84 | 0.990720954 | 0.000128752 |
| tch-miR-378a-3p | Traes_1AS_08A0A8A0E | 0.99072652 | 0.000128597 |
| tae-m2161-3p | Traes_3DL_8D8B3AD6F | 0.990728575 | 0.000128541 |
| tae-m3312-5p | Traes_3AL_E6A0E30B9 | 0.990731374 | 0.000128463 |
| tae-m0982-5p | Traes_7AS_2EB9E44921 | 0.990735325 | 0.000128354 |
| ssp-miR444b.2_R15-1L21 | Traes_3DL_416E228F3 | 0.990736667 | 0.000128317 |
| tae-m3294-3p | Traes_3DS_C0A4FDDDD | 0.990742077 | 0.000128167 |
| tae-m0982-5p | Traes_6BS_8DA635027 | 0.990744141 | 0.00012811 |
| oha-miR-99a-5p | Traes_6DL_3F7FAE7F9 | 0.990752101 | 0.00012789 |
| osa-miR5539b_R1-16L22 | Traes_2BL_A6BE13EEF | 0.990769285 | 0.000127416 |
| tae-m1521-5p | Traes_1AL_13F863A51 | 0.990780006 | 0.000127121 |
| tae-m2489-5p | Traes_1AL_13F863A51 | 0.990780006 | 0.000127121 |
| tae-m4416-5p | Traes_1AL_13F863A51 | 0.990780006 | 0.000127121 |
| ssp-miR444b.2_R15-1L21 | Traes_5BL_733D552E81 | 0.99078086 | 0.000127097 |
| bta-miR-2429_R19-5L20 | Traes_5DL_46901C98E | 0.990782384 | 0.000127055 |
| nta-miR156f | Traes_5DL_46901C98E | 0.990782384 | 0.000127055 |
| tae-m4030-5p | Traes_5DL_46901C98E | 0.990782384 | 0.000127055 |
| tae-m4349-5p | Traes_5DL_46901C98E | 0.990782384 | 0.000127055 |
| tch-miR-378a-3p | Traes_4AS_714D540BA | 0.990782801 | 0.000127044 |
| gga-miR-7475-5p_R20-6L20 | Traes_4AS_A3EAF8C80 | 0.990783829 | 0.000127015 |
| tae-m3294-3p | TRAES3BF091200050CFD_g | 0.990784832 | 0.000126988 |
| tae-m3821-3p | Traes_5DL_C0A5AC8F4 | 0.990785178 | 0.000126978 |
| tae-m0044-5p | TRAES3BF031900080CFD_g | 0.990795324 | 0.000126699 |
| gga-miR-7475-5p_R20-6L20 | Traes_7DL_63118F92C | 0.990798959 | 0.000126599 |
| tae-m0982-5p | Traes_1BL_B7318A659 | 0.990799195 | 0.000126593 |
| tae-m0982-5p | Traes_4BL_4CC0E0AAA | 0.990802124 | 0.000126512 |
| tae-m2161-3p | Traes_5DS_FB851A705 | 0.990803336 | 0.000126479 |
| tae-m0982-5p | Traes_3DL_3BBA9BB35 | 0.99080425 | 0.000126454 |
| tae-m2748-5p | Traes_4BL_0C7FECAE9 | 0.990814136 | 0.000126183 |
| gga-miR-7475-5p_R20-6L20 | Traes_4AS_714D540BA | 0.99082276 | 0.000125946 |
| tae-m0982-5p | Traes_4DL_056EBD3F1 | 0.990822899 | 0.000125942 |
| tch-miR-378a-3p | Traes_3AL_6D1917532 | 0.990833493 | 0.000125652 |
| tae-m0982-5p | Traes_2DS_C80293002 | 0.990835932 | 0.000125585 |
| tch-miR-378a-3p | Traes_2AL_57C3C7FAC | 0.990847202 | 0.000125277 |
| tae-m0561-5p | Traes_2DS_82AF233B4 | 0.990853292 | 0.000125111 |
| tae-m3821-3p | Traes_1BL_8BDEB895D | 0.990855023 | 0.000125064 |
| tae-m1383-5p | Traes_5DL_F075AAD29 | 0.990862406 | 0.000124862 |
| tae-m2333-5p | Traes_6BS_9B886B116 | 0.990864512 | 0.000124804 |
| tae-m3808-5p | Traes_6BS_9B886B116 | 0.990864512 | 0.000124804 |
| tae-m0298-5p | Traes_1AL_5EEF86979 | 0.99086465 | 0.000124801 |
| ata-miR172b-5p_R1-20L21 | Traes_6DL_EFFCA5F6F | 0.990865486 | 0.000124778 |
| bmo-miR-3302_R19-5L23 | Traes_6DL_EFFCA5F6F | 0.990865486 | 0.000124778 |
| tae-m0631-5p | Traes_6DL_EFFCA5F6F | 0.990865486 | 0.000124778 |
| bmo-miR-3208_R4-19L22 | Traes_7AS_BE336DC2E | 0.990865596 | 0.000124775 |
| tae-m1452-5p | Traes_7AS_BE336DC2E | 0.990865596 | 0.000124775 |
| dme-miR-954-3p_R15-1L21 | Traes_4AS_094442636 | 0.990871686 | 0.000124609 |
| tae-m4432-5p | Traes_5BL_8FD1FB0E1 | 0.990876309 | 0.000124483 |
| oha-miR-99a-5p | Traes_6DL_39276DDB6 | 0.990889502 | 0.000124124 |
| ata-miR167e-5p_R1-21L21 | Traes_1BL_F32B62E49 | 0.990891677 | 0.000124064 |
| tae-m3157-5p | Traes_1BL_F32B62E49 | 0.990891677 | 0.000124064 |
| tae-m1014-5p | Traes_4DS_E6210A614 | 0.990895562 | 0.000123959 |
| ata-miR172b-5p_R1-20L21 | Traes_7DL_DB6471BF0 | 0.990901484 | 0.000123798 |
| bmo-miR-3302_R19-5L23 | Traes_7DL_DB6471BF0 | 0.990901484 | 0.000123798 |
| tae-m0631-5p | Traes_7DL_DB6471BF0 | 0.990901484 | 0.000123798 |
| tae-m3294-3p | Traes_2DS_85E22FAC3 | 0.990910972 | 0.00012354 |
| ata-miR167e-5p_R1-21L21 | Traes_2DL_28DFAC79D | 0.990928125 | 0.000123075 |
| tae-m3157-5p | Traes_2DL_28DFAC79D | 0.990928125 | 0.000123075 |
| tae-m2333-5p | Traes_5DS_68BC2269E | 0.990929672 | 0.000123033 |
| tae-m3808-5p | Traes_5DS_68BC2269E | 0.990929672 | 0.000123033 |
| gma-miR5037a_R19-5L22 | Traes_5DS_C53497323 | 0.990930044 | 0.000123023 |
| tch-miR-378a-3p | Traes_6BS_B38F89538 | 0.990934411 | 0.000122905 |
| oha-miR-99a-5p | Traes_5BL_91F474465 | 0.990942049 | 0.000122698 |
| tae-miR9659-3p | Traes_2DS_6F88878F7 | 0.990944109 | 0.000122642 |
| bbe-miR-4857-5p_R3-17L23 | Traes_1DS_4F51F6DFA | 0.990949571 | 0.000122495 |
| hsa-miR-3677-5p_R17-3L22 | Traes_1DS_4F51F6DFA | 0.990949571 | 0.000122495 |
| bta-miR-2429_R19-5L20 | Traes_4DL_99168B17C | 0.990952454 | 0.000122417 |
| nta-miR156f | Traes_4DL_99168B17C | 0.990952454 | 0.000122417 |
| tae-m4030-5p | Traes_4DL_99168B17C | 0.990952454 | 0.000122417 |
| tae-m4349-5p | Traes_4DL_99168B17C | 0.990952454 | 0.000122417 |
| ata-miR172b-5p_R1-20L21 | Traes_2AL_0A1F83B9E | 0.990961109 | 0.000122183 |
| bmo-miR-3302_R19-5L23 | Traes_2AL_0A1F83B9E | 0.990961109 | 0.000122183 |
| tae-m0631-5p | Traes_2AL_0A1F83B9E | 0.990961109 | 0.000122183 |
| oha-miR-99a-5p | Traes_7DS_77FC13D7B1 | 0.990963294 | 0.000122124 |
| tae-m0044-5p | Traes_6DL_9FAE3F08F | 0.990964791 | 0.000122084 |
| tae-m0298-5p | TRAES3BF042900030CFD_g | 0.990971376 | 0.000121906 |
| osa-miR5539b_R1-16L22 | Traes_5BL_FAABCC38A | 0.990973632 | 0.000121845 |
| tae-m0982-5p | Traes_7BL_69588C86C | 0.990981733 | 0.000121627 |
| ppy-miR-4451_R1-16L18 | Traes_1BL_51774DCD7 | 0.990982862 | 0.000121597 |
| gma-miR5677_R15-1L21 | Traes_2DL_53D5FD18C | 0.990994948 | 0.000121271 |
| tae-m3603-5p | Traes_2DL_53D5FD18C | 0.990994948 | 0.000121271 |
| tae-m3782-5p | Traes_2DL_53D5FD18C | 0.990994948 | 0.000121271 |
| gga-miR-7475-5p_R20-6L20 | Traes_2BL_2825A3D0F | 0.990995497 | 0.000121257 |
| gga-miR-7475-5p_R20-6L20 | Traes_4DL_CBD89C78D | 0.990997805 | 0.000121194 |
| tae-m3821-3p | Traes_7AS_7E46CDBE9 | 0.991002706 | 0.000121063 |
| oha-miR-22a_R1-19L22 | Traes_5DL_C05F32EE4 | 0.991015655 | 0.000120715 |
| tae-m3821-3p | Traes_1BL_5ED9A20B3 | 0.9910206 | 0.000120582 |
| ata-miR9674a-5p_R1-18L21 | Traes_3AS_697F2FC13 | 0.991021268 | 0.000120565 |
| tae-m4429-5p | Traes_5DS_EA2F39971 | 0.99102566 | 0.000120447 |
| bbe-miR-4857-5p_R3-17L23 | Traes_1BL_C955569B8 | 0.991027976 | 0.000120385 |
| hsa-miR-3677-5p_R17-3L22 | Traes_1BL_C955569B8 | 0.991027976 | 0.000120385 |
| ata-miR167e-5p_R1-21L21 | Traes_2BS_CAA102CFC | 0.991042683 | 0.000119991 |
| tae-m3157-5p | Traes_2BS_CAA102CFC | 0.991042683 | 0.000119991 |
| tch-miR-378a-3p | Traes_3DS_C0A4FDDDD | 0.991044289 | 0.000119948 |
| oha-miR-22a_R1-19L22 | Traes_6DL_3F7FAE7F9 | 0.991046181 | 0.000119897 |
| ppy-miR-608_R7-21L25 | Traes_7DL_2351C7EEB | 0.991047957 | 0.00011985 |
| tae-m0195-5p | Traes_7BL_ABEAC99C8 | 0.99105225 | 0.000119735 |
| cpa-miR8155_R4-18L19 | Traes_3AL_F623F588A | 0.991053482 | 0.000119702 |
| tae-m2333-5p | Traes_5BL_A8B4F5EC6 | 0.991062475 | 0.000119462 |
| tae-m3808-5p | Traes_5BL_A8B4F5EC6 | 0.991062475 | 0.000119462 |
| tae-m0298-5p | Traes_7DL_2EDF5CB11 | 0.991065604 | 0.000119379 |
| smo-miR159_R2-21L21 | Traes_5BL_EDB3E6AD6 | 0.991113358 | 0.000118108 |
| tae-m1832-5p | Traes_5BL_EDB3E6AD6 | 0.991113358 | 0.000118108 |
| tae-m2038-5p | Traes_5BL_EDB3E6AD6 | 0.991113358 | 0.000118108 |
| tae-m3208-5p | Traes_5BL_EDB3E6AD6 | 0.991113358 | 0.000118108 |
| smo-miR159_R2-21L21 | Traes_4DS_6D718ECA2 | 0.991120821 | 0.00011791 |
| tae-m1832-5p | Traes_4DS_6D718ECA2 | 0.991120821 | 0.00011791 |
| tae-m2038-5p | Traes_4DS_6D718ECA2 | 0.991120821 | 0.00011791 |
| tae-m3208-5p | Traes_4DS_6D718ECA2 | 0.991120821 | 0.00011791 |
| tae-m4330-5p | Traes_2BL_633BE9A98 | 0.9911212 | 0.0001179 |
| oha-miR-199c-3p_R2-22L22 | Traes_6BL_30CAC7928 | 0.991124622 | 0.000117809 |
| tch-miR-378a-3p | Traes_4AL_A3091D983 | 0.991125436 | 0.000117787 |
| ata-miR167e-5p_R1-21L21 | Traes_7DS_1D44614AE | 0.991126686 | 0.000117754 |
| tae-m3157-5p | Traes_7DS_1D44614AE | 0.991126686 | 0.000117754 |
| tae-m2333-5p | Traes_7AL_7C3AF19DC | 0.991139758 | 0.000117408 |
| tae-m3808-5p | Traes_7AL_7C3AF19DC | 0.991139758 | 0.000117408 |
| bta-miR-2429_R19-5L20 | Traes_1AL_1D02EEFB9 | 0.991144263 | 0.000117289 |
| nta-miR156f | Traes_1AL_1D02EEFB9 | 0.991144263 | 0.000117289 |
| tae-m4030-5p | Traes_1AL_1D02EEFB9 | 0.991144263 | 0.000117289 |
| tae-m4349-5p | Traes_1AL_1D02EEFB9 | 0.991144263 | 0.000117289 |
| hvu-miR6209_R15-1L20 | Traes_5AL_F30A5E7F8 | 0.991153141 | 0.000117054 |
| oha-miR-199c-3p_R2-22L22 | Traes_7BS_C222B0121 | 0.991154077 | 0.000117029 |
| tae-m3294-3p | Traes_4DL_54A769EAC | 0.991155225 | 0.000116999 |
| oha-miR-199c-3p_R2-22L22 | Traes_1AL_1F70A2C8B | 0.991167063 | 0.000116687 |
| gga-miR-7475-5p_R20-6L20 | Traes_7BL_04D6F3E9E | 0.991169685 | 0.000116617 |
| ssp-miR444b.2_R15-1L21 | Traes_5DL_2DDE3DD76 | 0.991170184 | 0.000116604 |
| tae-miR396-5p_R1-21L21 | Traes_3DL_A631E1F0C | 0.991174173 | 0.000116499 |
| tae-miR9659-3p | Traes_1DL_88DD1E468 | 0.991174268 | 0.000116497 |
| sly-miR5300_R21-6L22 | Traes_7BS_2EF473B22 | 0.991174393 | 0.000116493 |
| chi-miR-133a-3p_R2-23L23 | Traes_7DL_24CBFF460 | 0.991177574 | 0.000116409 |
| tae-m2333-5p | Traes_6DL_3ED4C3974 | 0.991180582 | 0.00011633 |
| tae-m3808-5p | Traes_6DL_3ED4C3974 | 0.991180582 | 0.00011633 |
| sly-miR5300_R21-6L22 | Traes_4BL_9558D9CDF1 | 0.991181722 | 0.0001163 |
| oha-miR-199c-3p_R2-22L22 | Traes_5AL_6284FBBD8 | 0.99118185 | 0.000116297 |
| gga-miR-7475-5p_R20-6L20 | Traes_7DS_44D3A516A | 0.991184911 | 0.000116216 |
| tae-m4283-5p | Traes_6BS_85E5E342C | 0.991191445 | 0.000116044 |
| tch-miR-378a-3p | Traes_7AS_8D8503F70 | 0.991192608 | 0.000116014 |
| oha-miR-99a-5p | Traes_2DL_CCAE7B431 | 0.991192984 | 0.000116004 |
| nta-miR156f | Traes_3AS_D6CBEB7BA | 0.991199647 | 0.000115829 |
| tae-m4030-5p | Traes_3AS_D6CBEB7BA | 0.991199647 | 0.000115829 |
| tae-m4349-5p | Traes_3AS_D6CBEB7BA | 0.991199647 | 0.000115829 |
| bta-miR-2429_R19-5L20 | Traes_3AS_D6CBEB7BA | 0.991199647 | 0.000115829 |
| bmo-miR-3208_R4-19L22 | Traes_5DL_7E2053226 | 0.991204177 | 0.000115709 |
| tae-m1452-5p | Traes_5DL_7E2053226 | 0.991204177 | 0.000115709 |
| ppy-miR-608_R7-21L25 | Traes_2AL_D2FF0ECB4 | 0.991204306 | 0.000115706 |
| tae-m1918-5p | Traes_6DL_B28B5501C | 0.991210219 | 0.000115551 |
| tae-m2015-5p | Traes_6DL_B28B5501C | 0.991210219 | 0.000115551 |
| tae-m3263-5p | Traes_6DL_B28B5501C | 0.991210219 | 0.000115551 |
| tae-m4217-3p | Traes_6DL_B28B5501C | 0.991210219 | 0.000115551 |
| cme-miR156g_R1-18L20 | Traes_2DS_15F511161 | 0.991221496 | 0.000115255 |
| tae-m2996-5p | Traes_2DS_15F511161 | 0.991221496 | 0.000115255 |
| hvu-miR6209_R15-1L20 | Traes_6AS_A88CB01FD | 0.991227495 | 0.000115098 |
| tae-miR9659-3p | TRAES3BF031600050CFD_g | 0.991230151 | 0.000115028 |
| zma-miR395l-5p_R21-7L22 | Traes_5AL_534F40911 | 0.991245821 | 0.000114618 |
| tae-m4283-5p | Traes_5BL_404F6E798 | 0.991246872 | 0.000114591 |
| tae-m0982-5p | Traes_7BL_0D3422654 | 0.991249177 | 0.00011453 |
| tae-m0982-5p | Traes_1DL_F0FE02681 | 0.991261296 | 0.000114214 |
| tae-m0044-5p | Traes_4AL_6A515079C | 0.99126489 | 0.00011412 |
| gra-miR7505b_R6-20L21 | Traes_1BS_672D28589 | 0.991265233 | 0.000114111 |
| mml-miR-892c-3p_R1-17L22 | Traes_1BS_672D28589 | 0.991265233 | 0.000114111 |
| tae-m0403-5p | Traes_1BS_672D28589 | 0.991265233 | 0.000114111 |
| tae-m2665-5p | Traes_1BS_672D28589 | 0.991265233 | 0.000114111 |
| tae-m2876-5p | Traes_1BS_672D28589 | 0.991265233 | 0.000114111 |
| tae-m4081-5p | Traes_1BS_672D28589 | 0.991265233 | 0.000114111 |
| tae-m2069-5p | TRAES3BF017100050CFD_g | 0.991266976 | 0.000114066 |
| tch-miR-378a-3p | Traes_4BS_4EB2C19CF | 0.991268611 | 0.000114023 |
| tae-m4432-5p | Traes_7BS_FF9F03B12 | 0.991285779 | 0.000113576 |
| tae-m3821-3p | Traes_3DL_2C2D73C68 | 0.991286538 | 0.000113556 |
| bta-miR-760-5p_R17-3L22 | Traes_6DL_9FAE3F08F | 0.991290896 | 0.000113442 |
| cbr-miR-35a_R4-18L22 | Traes_6DL_9FAE3F08F | 0.991290896 | 0.000113442 |
| tae-m4294-5p | Traes_6DL_9FAE3F08F | 0.991290896 | 0.000113442 |
| gma-miR5677_R15-1L21 | Traes_5AS_423283446 | 0.991297382 | 0.000113274 |
| tae-m3603-5p | Traes_5AS_423283446 | 0.991297382 | 0.000113274 |
| tae-m3782-5p | Traes_5AS_423283446 | 0.991297382 | 0.000113274 |
| oha-miR-99a-5p | Traes_4BS_FFA687FA8 | 0.991311653 | 0.000112903 |
| tae-m4283-5p | Traes_7AS_3692334E1 | 0.99132564 | 0.00011254 |
| tae-m3865-3p | Traes_2BL_7F7F6544F | 0.991332051 | 0.000112374 |
| ssp-miR444b.2_R15-1L21 | Traes_2BL_6552196A1 | 0.991333707 | 0.000112332 |
| bta-miR-760-5p_R17-3L22 | Traes_7BL_2472DB370 | 0.991345337 | 0.000112031 |
| cbr-miR-35a_R4-18L22 | Traes_7BL_2472DB370 | 0.991345337 | 0.000112031 |
| tae-m4294-5p | Traes_7BL_2472DB370 | 0.991345337 | 0.000112031 |
| tae-m2333-5p | Traes_5BL_0A654A16E | 0.991357917 | 0.000111706 |
| tae-m3808-5p | Traes_5BL_0A654A16E | 0.991357917 | 0.000111706 |
| oha-miR-199c-3p_R2-22L22 | Traes_1BL_22B15A022 | 0.991357951 | 0.000111705 |
| oha-miR-199c-3p_R2-22L22 | Traes_1BL_05865F944 | 0.991359595 | 0.000111662 |
| oha-miR-99a-5p | Traes_6BS_5CF265676 | 0.991365357 | 0.000111514 |
| tae-m1383-5p | Traes_1BL_9CFA90539 | 0.991367157 | 0.000111467 |
| tae-m0195-5p | Traes_1BL_F4D20AE54 | 0.991371052 | 0.000111367 |
| smo-miR159_R2-21L21 | Traes_1DL_0D7B55EF1 | 0.991378581 | 0.000111173 |
| tae-m1832-5p | Traes_1DL_0D7B55EF1 | 0.991378581 | 0.000111173 |
| tae-m2038-5p | Traes_1DL_0D7B55EF1 | 0.991378581 | 0.000111173 |
| tae-m3208-5p | Traes_1DL_0D7B55EF1 | 0.991378581 | 0.000111173 |
| tch-miR-378a-3p | Traes_1AL_36B201285 | 0.991381117 | 0.000111108 |
| tae-m3294-3p | Traes_7DL_2A079DBC4 | 0.991383407 | 0.000111049 |
| oha-miR-199c-3p_R2-22L22 | Traes_4BL_36170B1A6 | 0.991389764 | 0.000110885 |
| tae-m2333-5p | Traes_7DL_7282B7CBD | 0.991392011 | 0.000110827 |
| tae-m3808-5p | Traes_7DL_7282B7CBD | 0.991392011 | 0.000110827 |
| hvu-miR6209_R15-1L20 | Traes_2DL_682D84924 | 0.991393042 | 0.000110801 |
| mmu-miR-3535_R7-21L26 | Traes_2BS_9A6251B43 | 0.991393784 | 0.000110782 |
| gra-miR7505b_R6-20L21 | Traes_2AL_DE86B9FD4 | 0.991398252 | 0.000110667 |
| mml-miR-892c-3p_R1-17L22 | Traes_2AL_DE86B9FD4 | 0.991398252 | 0.000110667 |
| tae-m0403-5p | Traes_2AL_DE86B9FD4 | 0.991398252 | 0.000110667 |
| tae-m2665-5p | Traes_2AL_DE86B9FD4 | 0.991398252 | 0.000110667 |
| tae-m2876-5p | Traes_2AL_DE86B9FD4 | 0.991398252 | 0.000110667 |
| tae-m4081-5p | Traes_2AL_DE86B9FD4 | 0.991398252 | 0.000110667 |
| mmu-miR-5106_R2-19L23 | Traes_2BS_2717F7DA4 | 0.991409572 | 0.000110376 |
| ata-miR9776-5p_R5-20L21 | Traes_5BL_3D3F5564B | 0.991417873 | 0.000110163 |
| tae-m2840-5p | Traes_5DL_A2A5A19B4 | 0.991423145 | 0.000110028 |
| tae-m0044-5p | Traes_2DL_6EAD0DD1C | 0.991427049 | 0.000109928 |
| ata-miR172b-5p_R1-20L21 | Traes_6AS_2A59D8EDC | 0.991430523 | 0.000109839 |
| bmo-miR-3302_R19-5L23 | Traes_6AS_2A59D8EDC | 0.991430523 | 0.000109839 |
| tae-m0631-5p | Traes_6AS_2A59D8EDC | 0.991430523 | 0.000109839 |
| tae-miR9659-3p | Traes_6BL_22FB1D3F4 | 0.991432101 | 0.000109799 |
| tae-m0982-5p | Traes_6DL_28AA9AE0E | 0.991436309 | 0.000109691 |
| ata-miR167e-5p_R1-21L21 | Traes_2BL_34819D129 | 0.991440575 | 0.000109582 |
| tae-m3157-5p | Traes_2BL_34819D129 | 0.991440575 | 0.000109582 |
| bta-miR-2429_R19-5L20 | Traes_4AS_4B47AEE0D | 0.991458906 | 0.000109114 |
| nta-miR156f | Traes_4AS_4B47AEE0D | 0.991458906 | 0.000109114 |
| tae-m4030-5p | Traes_4AS_4B47AEE0D | 0.991458906 | 0.000109114 |
| tae-m4349-5p | Traes_4AS_4B47AEE0D | 0.991458906 | 0.000109114 |
| tae-m4432-5p | Traes_7BL_89235BE1B | 0.991462308 | 0.000109027 |
| ata-miR167e-5p_R1-21L21 | Traes_4AL_C2A08A56A | 0.991465646 | 0.000108942 |
| tae-m3157-5p | Traes_4AL_C2A08A56A | 0.991465646 | 0.000108942 |
| bta-miR-2429_R19-5L20 | Traes_3AS_58EAB01FE | 0.99147063 | 0.000108815 |
| nta-miR156f | Traes_3AS_58EAB01FE | 0.99147063 | 0.000108815 |
| tae-m4030-5p | Traes_3AS_58EAB01FE | 0.99147063 | 0.000108815 |
| tae-m4349-5p | Traes_3AS_58EAB01FE | 0.99147063 | 0.000108815 |
| dme-miR-954-3p_R15-1L21 | Traes_2DL_5A2381464 | 0.9914812 | 0.000108546 |
| tae-miR9659-3p | Traes_1AL_C1EF04361 | 0.991491767 | 0.000108277 |
| ppy-miR-4451_R1-16L18 | Traes_2DS_EB5F25337 | 0.991500658 | 0.000108051 |
| efu-miR-9189d_R3-17L23 | Traes_6BL_57FC6AD11 | 0.99150205 | 0.000108016 |
| tae-miR9659-3p | Traes_1AL_C73885878 | 0.991507697 | 0.000107873 |
| bbe-miR-4857-5p_R3-17L23 | Traes_6AS_0520AAF4F | 0.991508548 | 0.000107851 |
| hsa-miR-3677-5p_R17-3L22 | Traes_6AS_0520AAF4F | 0.991508548 | 0.000107851 |
| mmu-miR-5627-5p_R18-4L22 | Traes_7DS_EE252D2DC | 0.991511137 | 0.000107785 |
| tae-m3294-3p | Traes_5BL_D190954CB | 0.991512453 | 0.000107752 |
| tae-m0982-5p | Traes_6AL_9AC15F055 | 0.991513199 | 0.000107733 |
| gga-miR-7475-5p_R20-6L20 | Traes_6DS_6BD4CA5B2 | 0.991524464 | 0.000107448 |
| ptr-miR-3937_R1-15L24 | Traes_2BL_7D2114731 | 0.991526693 | 0.000107391 |
| bdi-miR529-3p_R20-6L21 | Traes_2AS_D56DFCD76 | 0.991526889 | 0.000107386 |
| zma-miR395l-5p_R21-7L22 | Traes_5BL_404F6E798 | 0.991528478 | 0.000107346 |
| ata-miR167e-5p_R1-21L21 | Traes_5AL_8990624B8 | 0.991528812 | 0.000107338 |
| tae-m3157-5p | Traes_5AL_8990624B8 | 0.991528812 | 0.000107338 |
| tae-m3821-3p | Traes_2DL_EC8C5E66B | 0.99153386 | 0.00010721 |
| gra-miR7505b_R6-20L21 | Traes_5AL_FE34AC127 | 0.991535375 | 0.000107172 |
| mml-miR-892c-3p_R1-17L22 | Traes_5AL_FE34AC127 | 0.991535375 | 0.000107172 |
| tae-m0403-5p | Traes_5AL_FE34AC127 | 0.991535375 | 0.000107172 |
| tae-m2665-5p | Traes_5AL_FE34AC127 | 0.991535375 | 0.000107172 |
| tae-m2876-5p | Traes_5AL_FE34AC127 | 0.991535375 | 0.000107172 |
| tae-m4081-5p | Traes_5AL_FE34AC127 | 0.991535375 | 0.000107172 |
| mmu-miR-5627-5p_R18-4L22 | Traes_6DS_053061050 | 0.991536129 | 0.000107153 |
| smo-miR159_R2-21L21 | Traes_1DS_69740CF6C | 0.991538068 | 0.000107103 |
| tae-m1832-5p | Traes_1DS_69740CF6C | 0.991538068 | 0.000107103 |
| tae-m2038-5p | Traes_1DS_69740CF6C | 0.991538068 | 0.000107103 |
| tae-m3208-5p | Traes_1DS_69740CF6C | 0.991538068 | 0.000107103 |
| tae-m3294-3p | Traes_4AS_1938645BA | 0.99154422 | 0.000106948 |
| bdi-miR156f-3p | Traes_4AL_C4C082F05 | 0.99156259 | 0.000106484 |
| tae-m2748-5p | Traes_5DL_F292F9EA4 | 0.991563984 | 0.000106449 |
| tae-m0982-5p | Traes_1AS_EB6754AA5 | 0.991568099 | 0.000106346 |
| hvu-miR6177_R6-22L22 | TRAES3BF276100090CFD_g | 0.991576704 | 0.000106129 |
| tch-miR-378a-3p | Traes_4BL_0F72E76F1 | 0.991590454 | 0.000105783 |
| tae-m0982-5p | Traes_2BL_38F4078EC | 0.991591428 | 0.000105759 |
| tae-m4432-5p | Traes_3DS_B886F28ED | 0.991595271 | 0.000105662 |
| mmu-miR-5106_R2-19L23 | Traes_6AL_904D2D9AB | 0.991598034 | 0.000105593 |
| oha-miR-22a_R1-19L22 | Traes_2DL_CCAE7B431 | 0.991609003 | 0.000105318 |
| dpr-miR397_R17-1L21 | Traes_2DL_AEE3FBCED | 0.991622099 | 0.00010499 |
| tae-m1322-5p | Traes_2DL_AEE3FBCED | 0.991622099 | 0.00010499 |
| tae-m3641-5p | Traes_2DL_AEE3FBCED | 0.991622099 | 0.00010499 |
| xtr-miR-428a_R7-22L22 | Traes_2DL_AEE3FBCED | 0.991622099 | 0.00010499 |
| oha-miR-99a-5p | Traes_2DS_E8B3ED40A1 | 0.991623585 | 0.000104953 |
| gga-miR-7475-5p_R20-6L20 | Traes_1AS_06988AF2C | 0.991623755 | 0.000104948 |
| smo-miR159_R2-21L21 | Traes_2BL_E5A7188DB | 0.991630759 | 0.000104773 |
| tae-m1832-5p | Traes_2BL_E5A7188DB | 0.991630759 | 0.000104773 |
| tae-m2038-5p | Traes_2BL_E5A7188DB | 0.991630759 | 0.000104773 |
| tae-m3208-5p | Traes_2BL_E5A7188DB | 0.991630759 | 0.000104773 |
| tae-m2597-5p | Traes_7DL_702672926 | 0.99163147 | 0.000104755 |
| ata-miR167e-5p_R1-21L21 | Traes_4DS_6A7D1C662 | 0.99164132 | 0.000104509 |
| tae-m3157-5p | Traes_4DS_6A7D1C662 | 0.99164132 | 0.000104509 |
| tae-m4222-3p | Traes_1AS_34DBDBA7D | 0.991644167 | 0.000104438 |
| tae-m2665-5p | Traes_4AL_92708BAA0 | 0.991648195 | 0.000104338 |
| tae-m4081-5p | Traes_4AL_92708BAA0 | 0.991648195 | 0.000104338 |
| gra-miR7505b_R6-20L21 | Traes_4AL_92708BAA0 | 0.991648195 | 0.000104338 |
| mml-miR-892c-3p_R1-17L22 | Traes_4AL_92708BAA0 | 0.991648195 | 0.000104338 |
| tae-m0403-5p | Traes_4AL_92708BAA0 | 0.991648195 | 0.000104338 |
| tae-m2876-5p | Traes_4AL_92708BAA0 | 0.991648195 | 0.000104338 |
| tae-m3294-3p | Traes_2AS_235EEA0AE | 0.991653 | 0.000104218 |
| oha-miR-99a-5p | Traes_6BS_7F65A4F6C | 0.991666248 | 0.000103888 |
| bta-miR-2429_R19-5L20 | Traes_6BS_5CF265676 | 0.991672578 | 0.00010373 |
| nta-miR156f | Traes_6BS_5CF265676 | 0.991672578 | 0.00010373 |
| tae-m4030-5p | Traes_6BS_5CF265676 | 0.991672578 | 0.00010373 |
| tae-m4349-5p | Traes_6BS_5CF265676 | 0.991672578 | 0.00010373 |
| tch-miR-378a-3p | Traes_2DL_FA7A15F63 | 0.991683019 | 0.000103471 |
| oha-miR-99a-5p | Traes_6BL_30CAC7928 | 0.991688759 | 0.000103328 |
| ata-miR172b-5p_R1-20L21 | Traes_4DL_478BB6FEF | 0.991699548 | 0.00010306 |
| bmo-miR-3302_R19-5L23 | Traes_4DL_478BB6FEF | 0.991699548 | 0.00010306 |
| tae-m0631-5p | Traes_4DL_478BB6FEF | 0.991699548 | 0.00010306 |
| gma-miR5677_R15-1L21 | Traes_1DL_5DC4E9455 | 0.991701995 | 0.000103 |
| tae-m3603-5p | Traes_1DL_5DC4E9455 | 0.991701995 | 0.000103 |
| tae-m3782-5p | Traes_1DL_5DC4E9455 | 0.991701995 | 0.000103 |
| tae-m1521-5p | Traes_4BS_AE8BCD580 | 0.991708119 | 0.000102848 |
| tae-m2489-5p | Traes_4BS_AE8BCD580 | 0.991708119 | 0.000102848 |
| tae-m4416-5p | Traes_4BS_AE8BCD580 | 0.991708119 | 0.000102848 |
| tae-m4432-5p | Traes_1DL_53DDD6D20 | 0.991712057 | 0.00010275 |
| tae-m0147-5p | Traes_5DL_20A09D99E | 0.991721124 | 0.000102526 |
| tae-m3576-5p | Traes_5DL_20A09D99E | 0.991721124 | 0.000102526 |
| tae-m3683-5p | Traes_5DL_20A09D99E | 0.991721124 | 0.000102526 |
| mmu-miR-5627-5p_R18-4L22 | Traes_7DL_4B936BD0A | 0.991721914 | 0.000102506 |
| ata-miR167e-5p_R1-21L21 | Traes_4BS_C1AB2DDA8 | 0.991728367 | 0.000102347 |
| tae-m3157-5p | Traes_4BS_C1AB2DDA8 | 0.991728367 | 0.000102347 |
| bta-miR-2429_R19-5L20 | Traes_6AL_FE462C6F8 | 0.991736144 | 0.000102155 |
| nta-miR156f | Traes_6AL_FE462C6F8 | 0.991736144 | 0.000102155 |
| tae-m4030-5p | Traes_6AL_FE462C6F8 | 0.991736144 | 0.000102155 |
| tae-m4349-5p | Traes_6AL_FE462C6F8 | 0.991736144 | 0.000102155 |
| oha-miR-199c-3p_R2-22L22 | Traes_7DL_57CEECAF6 | 0.99173768 | 0.000102117 |
| efu-miR-9189d_R3-17L23 | Traes_6AL_24A5FC5D9 | 0.991749386 | 0.000101828 |
| gma-miR5037a_R19-5L22 | Traes_1AL_341E2A7A8 | 0.99175518 | 0.000101685 |
| tch-miR-378a-3p | Traes_4BS_F2B98DC82 | 0.991760037 | 0.000101566 |
| gga-miR-7475-5p_R20-6L20 | Traes_5AL_81745FA28 | 0.991761501 | 0.00010153 |
| gra-miR7505b_R6-20L21 | Traes_2BL_BD8DBA189 | 0.991763501 | 0.00010148 |
| mml-miR-892c-3p_R1-17L22 | Traes_2BL_BD8DBA189 | 0.991763501 | 0.00010148 |
| tae-m0403-5p | Traes_2BL_BD8DBA189 | 0.991763501 | 0.00010148 |
| tae-m2665-5p | Traes_2BL_BD8DBA189 | 0.991763501 | 0.00010148 |
| tae-m2876-5p | Traes_2BL_BD8DBA189 | 0.991763501 | 0.00010148 |
| tae-m4081-5p | Traes_2BL_BD8DBA189 | 0.991763501 | 0.00010148 |
| efu-miR-9189e_R24-4L24_8A-C | Traes_4BL_1C9103753 | 0.991769382 | 0.000101336 |
| mtr-miR2655b_R4-18L21 | Traes_1BL_5E6CEFBC5 | 0.99178273 | 0.000101008 |
| mmu-miR-3535_R7-21L26 | Traes_4BL_9558D9CDF1 | 0.991783128 | 0.000100998 |
| tch-miR-378a-3p | Traes_7DL_A321818FB | 0.991785515 | 0.000100939 |
| tae-m3312-5p | Traes_6AS_8C0D1110B | 0.991787869 | 0.000100882 |
| tae-m0982-5p | Traes_2DL_49AA3F1D1 | 0.99179218 | 0.000100776 |
| tae-m0982-5p | Traes_1DL_274235AB5 | 0.991793751 | 0.000100737 |
| gma-miR5677_R15-1L21 | Traes_3AS_7BF3615EC | 0.991799572 | 0.000100595 |
| tae-m3603-5p | Traes_3AS_7BF3615EC | 0.991799572 | 0.000100595 |
| tae-m3782-5p | Traes_3AS_7BF3615EC | 0.991799572 | 0.000100595 |
| tch-miR-378a-3p | Traes_1BL_AE6A21360 | 0.991803614 | 0.000100496 |
| tae-m0044-5p | TRAES3BF035300100CFD_g | 0.991803645 | 0.000100495 |
| tae-m0561-5p | Traes_5DL_134F29727 | 0.991809301 | 0.000100357 |
| bta-miR-2429_R19-5L20 | Traes_6DS_204661A07 | 0.991812012 | 0.00010029 |
| nta-miR156f | Traes_6DS_204661A07 | 0.991812012 | 0.00010029 |
| tae-m4030-5p | Traes_6DS_204661A07 | 0.991812012 | 0.00010029 |
| tae-m4349-5p | Traes_6DS_204661A07 | 0.991812012 | 0.00010029 |
| ssp-miR444b.2_R15-1L21 | Traes_4AL_E8EEC0D6E | 0.991828509 | 9.99E-05 |
| bta-miR-2429_R19-5L20 | Traes_6AL_9AC15F055 | 0.991828575 | 9.99E-05 |
| nta-miR156f | Traes_6AL_9AC15F055 | 0.991828575 | 9.99E-05 |
| tae-m4030-5p | Traes_6AL_9AC15F055 | 0.991828575 | 9.99E-05 |
| tae-m4349-5p | Traes_6AL_9AC15F055 | 0.991828575 | 9.99E-05 |
| tae-m0195-5p | Traes_4DL_A780BF320 | 0.991846284 | 9.95E-05 |
| gra-miR7505b_R6-20L21 | Traes_2DL_6CD064E13 | 0.991852804 | 9.93E-05 |
| mml-miR-892c-3p_R1-17L22 | Traes_2DL_6CD064E13 | 0.991852804 | 9.93E-05 |
| tae-m0403-5p | Traes_2DL_6CD064E13 | 0.991852804 | 9.93E-05 |
| tae-m2665-5p | Traes_2DL_6CD064E13 | 0.991852804 | 9.93E-05 |
| tae-m2876-5p | Traes_2DL_6CD064E13 | 0.991852804 | 9.93E-05 |
| tae-m4081-5p | Traes_2DL_6CD064E13 | 0.991852804 | 9.93E-05 |
| bbe-miR-4857-5p_R3-17L23 | Traes_6BL_EDE707220 | 0.991854804 | 9.92E-05 |
| hsa-miR-3677-5p_R17-3L22 | Traes_6BL_EDE707220 | 0.991854804 | 9.92E-05 |
| tae-m3294-3p | TRAES3BF007900030CFD_g | 0.991856687 | 9.92E-05 |
| tae-m0147-5p | Traes_7AS_FC4D18A1D | 0.99186269 | 9.91E-05 |
| tae-m3576-5p | Traes_7AS_FC4D18A1D | 0.99186269 | 9.91E-05 |
| tae-m3683-5p | Traes_7AS_FC4D18A1D | 0.99186269 | 9.91E-05 |
| tae-m3294-3p | Traes_2BL_A402261EC1 | 0.99187635 | 9.87E-05 |
| tae-m0044-5p | Traes_6BS_7800EA2AE | 0.991894144 | 9.83E-05 |
| tae-m2333-5p | Traes_5AL_3EF409B1D | 0.991896248 | 9.82E-05 |
| tae-m3808-5p | Traes_5AL_3EF409B1D | 0.991896248 | 9.82E-05 |
| oha-miR-199c-3p_R2-22L22 | Traes_7BL_83400E481 | 0.991904424 | 9.80E-05 |
| oha-miR-22a_R1-19L22 | Traes_1DL_1D9958B1E | 0.991906176 | 9.80E-05 |
| bbe-miR-4857-5p_R3-17L23 | Traes_1BL_08309EDEA | 0.991910804 | 9.79E-05 |
| hsa-miR-3677-5p_R17-3L22 | Traes_1BL_08309EDEA | 0.991910804 | 9.79E-05 |
| hvu-miR6209_R15-1L20 | Traes_4DL_83C41A77C1 | 0.991915284 | 9.78E-05 |
| oha-miR-99a-5p | Traes_1DS_75AF80583 | 0.99192084 | 9.76E-05 |
| tae-m3294-3p | Traes_5DL_E4168273F | 0.991923979 | 9.76E-05 |
| tae-m0295-5p | Traes_5BL_C57898165 | 0.991927027 | 9.75E-05 |
| tae-m4283-5p | Traes_1BS_DD55B7D8F | 0.991936714 | 9.73E-05 |
| hvu-miR6209_R15-1L20 | Traes_4BL_4E4466B13 | 0.991942191 | 9.71E-05 |
| smo-miR159_R2-21L21 | TRAES3BF081300060CFD_g | 0.99195016 | 9.69E-05 |
| tae-m1832-5p | TRAES3BF081300060CFD_g | 0.99195016 | 9.69E-05 |
| tae-m2038-5p | TRAES3BF081300060CFD_g | 0.99195016 | 9.69E-05 |
| tae-m3208-5p | TRAES3BF081300060CFD_g | 0.99195016 | 9.69E-05 |
| tae-m1383-5p | Traes_4AS_D9959802D | 0.991954587 | 9.68E-05 |
| ata-miR172b-5p_R1-20L21 | Traes_1DL_CAA9298F0 | 0.991955733 | 9.68E-05 |
| bmo-miR-3302_R19-5L23 | Traes_1DL_CAA9298F0 | 0.991955733 | 9.68E-05 |
| tae-m0631-5p | Traes_1DL_CAA9298F0 | 0.991955733 | 9.68E-05 |
| tch-miR-378a-3p | TRAES3BF038000030CFD_g | 0.991957747 | 9.68E-05 |
| tch-miR-378a-3p | Traes_1AS_06988AF2C | 0.991960137 | 9.67E-05 |
| tae-m1383-5p | Traes_5BS_D0C6E034C | 0.991965518 | 9.66E-05 |
| smo-miR159_R2-21L21 | Traes_7DS_F770E4011 | 0.991983887 | 9.61E-05 |
| tae-m1832-5p | Traes_7DS_F770E4011 | 0.991983887 | 9.61E-05 |
| tae-m2038-5p | Traes_7DS_F770E4011 | 0.991983887 | 9.61E-05 |
| tae-m3208-5p | Traes_7DS_F770E4011 | 0.991983887 | 9.61E-05 |
| ata-miR172b-5p_R1-20L21 | Traes_2AS_6AB3D73F7 | 0.991987482 | 9.60E-05 |
| bmo-miR-3302_R19-5L23 | Traes_2AS_6AB3D73F7 | 0.991987482 | 9.60E-05 |
| tae-m0631-5p | Traes_2AS_6AB3D73F7 | 0.991987482 | 9.60E-05 |
| gga-miR-7475-5p_R20-6L20 | Traes_7BL_27009DE7F | 0.991994953 | 9.59E-05 |
| tch-miR-378a-3p | Traes_1AL_CF6C67E1B | 0.991998379 | 9.58E-05 |
| gga-miR-7475-5p_R20-6L20 | Traes_2DS_A270E9A2B | 0.991998986 | 9.58E-05 |
| ata-miR172b-5p_R1-20L21 | Traes_4DL_056EBD3F1 | 0.992005564 | 9.56E-05 |
| bmo-miR-3302_R19-5L23 | Traes_4DL_056EBD3F1 | 0.992005564 | 9.56E-05 |
| tae-m0631-5p | Traes_4DL_056EBD3F1 | 0.992005564 | 9.56E-05 |
| ata-miR167e-5p_R1-21L21 | Traes_2BL_4BCDE1D48 | 0.992008993 | 9.55E-05 |
| tae-m3157-5p | Traes_2BL_4BCDE1D48 | 0.992008993 | 9.55E-05 |
| gra-miR7505b_R6-20L21 | Traes_4AL_234D9FEC7 | 0.992009305 | 9.55E-05 |
| mml-miR-892c-3p_R1-17L22 | Traes_4AL_234D9FEC7 | 0.992009305 | 9.55E-05 |
| tae-m0403-5p | Traes_4AL_234D9FEC7 | 0.992009305 | 9.55E-05 |
| tae-m2665-5p | Traes_4AL_234D9FEC7 | 0.992009305 | 9.55E-05 |
| tae-m2876-5p | Traes_4AL_234D9FEC7 | 0.992009305 | 9.55E-05 |
| tae-m4081-5p | Traes_4AL_234D9FEC7 | 0.992009305 | 9.55E-05 |
| tae-m4368-5p | Traes_5AL_E4E5B111A | 0.992012516 | 9.54E-05 |
| tae-m3294-3p | Traes_5AS_51DBC1F49 | 0.992029368 | 9.50E-05 |
| tae-m3294-3p | Traes_7DS_51E4B75F7 | 0.992037328 | 9.49E-05 |
| gma-miR5037a_R19-5L22 | Traes_1AS_E2E5E14DC | 0.992058916 | 9.43E-05 |
| oha-miR-22a_R1-19L22 | Traes_6AL_F856D4C7A | 0.992062864 | 9.42E-05 |
| tae-m2333-5p | Traes_4BL_04B01EA0C | 0.992088455 | 9.36E-05 |
| tae-m3808-5p | Traes_4BL_04B01EA0C | 0.992088455 | 9.36E-05 |
| gma-miR5677_R15-1L21 | Traes_2BS_CADAA49C9 | 0.992090465 | 9.36E-05 |
| tae-m3603-5p | Traes_2BS_CADAA49C9 | 0.992090465 | 9.36E-05 |
| tae-m3782-5p | Traes_2BS_CADAA49C9 | 0.992090465 | 9.36E-05 |
| oha-miR-22a_R1-19L22 | Traes_1DS_75AF80583 | 0.992092231 | 9.36E-05 |
| tae-m1918-5p | Traes_5DL_134F29727 | 0.99209385 | 9.35E-05 |
| tae-m2015-5p | Traes_5DL_134F29727 | 0.99209385 | 9.35E-05 |
| tae-m3263-5p | Traes_5DL_134F29727 | 0.99209385 | 9.35E-05 |
| tae-m4217-3p | Traes_5DL_134F29727 | 0.99209385 | 9.35E-05 |
| smo-miR159_R2-21L21 | Traes_5AL_BCA187DFF | 0.99210072 | 9.34E-05 |
| tae-m1832-5p | Traes_5AL_BCA187DFF | 0.99210072 | 9.34E-05 |
| tae-m2038-5p | Traes_5AL_BCA187DFF | 0.99210072 | 9.34E-05 |
| tae-m3208-5p | Traes_5AL_BCA187DFF | 0.99210072 | 9.34E-05 |
| nta-miR156f | Traes_1BL_05865F944 | 0.992102843 | 9.33E-05 |
| tae-m4030-5p | Traes_1BL_05865F944 | 0.992102843 | 9.33E-05 |
| tae-m4349-5p | Traes_1BL_05865F944 | 0.992102843 | 9.33E-05 |
| bta-miR-2429_R19-5L20 | Traes_1BL_05865F944 | 0.992102843 | 9.33E-05 |
| tae-m2333-5p | TRAES3BF033900100CFD_g | 0.992103054 | 9.33E-05 |
| tae-m3808-5p | TRAES3BF033900100CFD_g | 0.992103054 | 9.33E-05 |
| bta-miR-760-5p_R17-3L22 | TRAES3BF117500060CFD_g | 0.992125606 | 9.28E-05 |
| cbr-miR-35a_R4-18L22 | TRAES3BF117500060CFD_g | 0.992125606 | 9.28E-05 |
| tae-m4294-5p | TRAES3BF117500060CFD_g | 0.992125606 | 9.28E-05 |
| tae-m4283-5p | Traes_7BL_30974FA2F1 | 0.992127088 | 9.27E-05 |
| tae-miR9659-3p | Traes_2DL_06741A74F | 0.992138777 | 9.25E-05 |
| gra-miR7505b_R6-20L21 | TRAES3BF082600160CFD_g | 0.992146755 | 9.23E-05 |
| mml-miR-892c-3p_R1-17L22 | TRAES3BF082600160CFD_g | 0.992146755 | 9.23E-05 |
| tae-m0403-5p | TRAES3BF082600160CFD_g | 0.992146755 | 9.23E-05 |
| tae-m2665-5p | TRAES3BF082600160CFD_g | 0.992146755 | 9.23E-05 |
| tae-m2876-5p | TRAES3BF082600160CFD_g | 0.992146755 | 9.23E-05 |
| tae-m4081-5p | TRAES3BF082600160CFD_g | 0.992146755 | 9.23E-05 |
| oha-miR-199c-3p_R2-22L22 | Traes_3DL_C5D9E24D5 | 0.99214717 | 9.23E-05 |
| tae-m0982-5p | Traes_7AS_E0F56CCBF1 | 0.992147252 | 9.23E-05 |
| tch-miR-378a-3p | Traes_1AL_91E56EC8C | 0.992154835 | 9.21E-05 |
| hvu-miR6209_R15-1L20 | Traes_4DS_7394D3FBE | 0.992164222 | 9.19E-05 |
| oha-miR-99a-5p | Traes_3DS_C74F56379 | 0.992165788 | 9.18E-05 |
| dpr-miR397_R17-1L21 | Traes_1AL_D1AF9EF20 | 0.992172104 | 9.17E-05 |
| tae-m1322-5p | Traes_1AL_D1AF9EF20 | 0.992172104 | 9.17E-05 |
| tae-m3641-5p | Traes_1AL_D1AF9EF20 | 0.992172104 | 9.17E-05 |
| xtr-miR-428a_R7-22L22 | Traes_1AL_D1AF9EF20 | 0.992172104 | 9.17E-05 |
| bbe-miR-4857-5p_R3-17L23 | Traes_1BS_2E766829E | 0.99217347 | 9.16E-05 |
| hsa-miR-3677-5p_R17-3L22 | Traes_1BS_2E766829E | 0.99217347 | 9.16E-05 |
| tae-m3312-5p | Traes_7DL_C4F9E6AC0 | 0.992173679 | 9.16E-05 |
| tae-m0599-5p | Traes_2BL_F7A3C3775 | 0.992197687 | 9.11E-05 |
| tae-m4330-5p | Traes_5BS_4C0A12D38 | 0.992198849 | 9.10E-05 |
| ssp-miR444b.2_R15-1L21 | TRAES3BF105300050CFD_g | 0.99219988 | 9.10E-05 |
| sbi-miR396d | TRAES3BF034700070CFD_g | 0.992202485 | 9.10E-05 |
| bta-miR-760-5p_R17-3L22 | Traes_6AL_60E7A9254 | 0.992203697 | 9.09E-05 |
| cbr-miR-35a_R4-18L22 | Traes_6AL_60E7A9254 | 0.992203697 | 9.09E-05 |
| tae-m4294-5p | Traes_6AL_60E7A9254 | 0.992203697 | 9.09E-05 |
| gga-miR-7475-5p_R20-6L20 | Traes_2AL_D344EC2AF | 0.992209691 | 9.08E-05 |
| tae-m0147-5p | Traes_5BL_0BFAE33B9 | 0.992209887 | 9.08E-05 |
| tae-m3576-5p | Traes_5BL_0BFAE33B9 | 0.992209887 | 9.08E-05 |
| tae-m3683-5p | Traes_5BL_0BFAE33B9 | 0.992209887 | 9.08E-05 |
| tae-miR9659-3p | Traes_1DS_07AD88BDA | 0.992216628 | 9.06E-05 |
| tae-m1918-5p | Traes_1AL_5184A1376 | 0.992219495 | 9.06E-05 |
| tae-m2015-5p | Traes_1AL_5184A1376 | 0.992219495 | 9.06E-05 |
| tae-m3263-5p | Traes_1AL_5184A1376 | 0.992219495 | 9.06E-05 |
| tae-m4217-3p | Traes_1AL_5184A1376 | 0.992219495 | 9.06E-05 |
| tae-m2840-5p | Traes_6BS_9752E1BBC | 0.992228183 | 9.04E-05 |
| gma-miR5037a_R19-5L22 | Traes_7DL_40E0AD332 | 0.992228216 | 9.04E-05 |
| tae-miR156_R2-20L23 | Traes_3AS_B7522D4FE | 0.992228913 | 9.04E-05 |
| tae-m0488-5p | Traes_7DS_003BEB003 | 0.992230701 | 9.03E-05 |
| tae-m4293-5p | Traes_7DS_003BEB003 | 0.992230701 | 9.03E-05 |
| gra-miR7505b_R6-20L21 | Traes_6AL_116E0BA24 | 0.992233961 | 9.02E-05 |
| mml-miR-892c-3p_R1-17L22 | Traes_6AL_116E0BA24 | 0.992233961 | 9.02E-05 |
| tae-m0403-5p | Traes_6AL_116E0BA24 | 0.992233961 | 9.02E-05 |
| tae-m2665-5p | Traes_6AL_116E0BA24 | 0.992233961 | 9.02E-05 |
| tae-m2876-5p | Traes_6AL_116E0BA24 | 0.992233961 | 9.02E-05 |
| tae-m4081-5p | Traes_6AL_116E0BA24 | 0.992233961 | 9.02E-05 |
| tae-m1521-5p | Traes_4BL_25A23A7EC | 0.992240772 | 9.01E-05 |
| tae-m2489-5p | Traes_4BL_25A23A7EC | 0.992240772 | 9.01E-05 |
| tae-m4416-5p | Traes_4BL_25A23A7EC | 0.992240772 | 9.01E-05 |
| tae-m0982-5p | Traes_2DS_B44C394FB | 0.992242555 | 9.00E-05 |
| zma-miR395l-5p_R21-7L22 | Traes_2BS_6AECC4811 | 0.992258594 | 8.97E-05 |
| oha-miR-199c-3p_R2-22L22 | TRAES3BF116200270CFD_g | 0.992280379 | 8.92E-05 |
| tae-m4368-5p | Traes_1DL_5DC4E9455 | 0.992287421 | 8.90E-05 |
| tae-m4429-5p | Traes_1BL_EF484D43E | 0.992288412 | 8.90E-05 |
| bta-miR-760-5p_R17-3L22 | Traes_4BL_44AB1802E | 0.992289229 | 8.90E-05 |
| cbr-miR-35a_R4-18L22 | Traes_4BL_44AB1802E | 0.992289229 | 8.90E-05 |
| tae-m4294-5p | Traes_4BL_44AB1802E | 0.992289229 | 8.90E-05 |
| oha-miR-199c-3p_R2-22L22 | Traes_1BL_34181088A | 0.992289814 | 8.89E-05 |
| bta-miR-2429_R19-5L20 | Traes_7BL_75AC97945 | 0.992296629 | 8.88E-05 |
| nta-miR156f | Traes_7BL_75AC97945 | 0.992296629 | 8.88E-05 |
| tae-m4030-5p | Traes_7BL_75AC97945 | 0.992296629 | 8.88E-05 |
| tae-m4349-5p | Traes_7BL_75AC97945 | 0.992296629 | 8.88E-05 |
| efu-miR-9189d_R3-17L23 | Traes_1AS_DEAC58FC4 | 0.992307074 | 8.85E-05 |
| bta-miR-760-5p_R17-3L22 | Traes_7BS_E6B3A196E | 0.992327533 | 8.81E-05 |
| cbr-miR-35a_R4-18L22 | Traes_7BS_E6B3A196E | 0.992327533 | 8.81E-05 |
| tae-m4294-5p | Traes_7BS_E6B3A196E | 0.992327533 | 8.81E-05 |
| tae-m4218-5p | Traes_2BL_3D459A937 | 0.992337652 | 8.78E-05 |
| gga-miR-7475-5p_R20-6L20 | Traes_5DL_0ED2854E2 | 0.992354312 | 8.75E-05 |
| gga-miR-7475-5p_R20-6L20 | Traes_6DL_24A8AB125 | 0.992365734 | 8.72E-05 |
| ppy-miR-4451_R1-16L18 | Traes_2AL_0D81DFB10 | 0.992397347 | 8.65E-05 |
| oha-miR-22a_R1-19L22 | Traes_7BL_30974FA2F1 | 0.992399274 | 8.64E-05 |
| tae-m3821-3p | Traes_6AL_751C1A39D | 0.992402109 | 8.64E-05 |
| bbe-miR-4857-5p_R3-17L23 | TRAES3BF052300070CFD_g | 0.992404445 | 8.63E-05 |
| hsa-miR-3677-5p_R17-3L22 | TRAES3BF052300070CFD_g | 0.992404445 | 8.63E-05 |
| ppy-miR-608_R7-21L25 | Traes_4BL_9DCECEEEE | 0.992409251 | 8.62E-05 |
| tch-miR-378a-3p | Traes_1BL_F143786F5 | 0.992425022 | 8.59E-05 |
| dme-miR-954-3p_R15-1L21 | Traes_5BL_8FDBF53BC | 0.992425783 | 8.58E-05 |
| bmo-miR-3208_R4-19L22 | Traes_4BL_396369E0B | 0.992425889 | 8.58E-05 |
| tae-m1452-5p | Traes_4BL_396369E0B | 0.992425889 | 8.58E-05 |
| tae-miR1121_R3-21L22 | Traes_3DL_A9A607A62 | 0.992431427 | 8.57E-05 |
| tae-miR396-5p_R1-21L21 | Traes_7DL_E82D94FA8 | 0.992435094 | 8.56E-05 |
| smo-miR159_R2-21L21 | Traes_2DS_EB5F25337 | 0.992442709 | 8.55E-05 |
| tae-m1832-5p | Traes_2DS_EB5F25337 | 0.992442709 | 8.55E-05 |
| tae-m2038-5p | Traes_2DS_EB5F25337 | 0.992442709 | 8.55E-05 |
| tae-m3208-5p | Traes_2DS_EB5F25337 | 0.992442709 | 8.55E-05 |
| ata-miR167e-5p_R1-21L21 | Traes_4AL_DF47C07FD | 0.992445091 | 8.54E-05 |
| tae-m3157-5p | Traes_4AL_DF47C07FD | 0.992445091 | 8.54E-05 |
| tae-miR9659-3p | Traes_3AL_716569701 | 0.992460796 | 8.50E-05 |
| gma-miR5037a_R19-5L22 | Traes_4AS_5D6957D15 | 0.992473496 | 8.48E-05 |
| dpr-miR397_R17-1L21 | Traes_2BL_4BCDE1D48 | 0.992474722 | 8.47E-05 |
| tae-m1322-5p | Traes_2BL_4BCDE1D48 | 0.992474722 | 8.47E-05 |
| tae-m3641-5p | Traes_2BL_4BCDE1D48 | 0.992474722 | 8.47E-05 |
| xtr-miR-428a_R7-22L22 | Traes_2BL_4BCDE1D48 | 0.992474722 | 8.47E-05 |
| gga-miR-7475-5p_R20-6L20 | Traes_4BL_E2E2C4E1D | 0.992475562 | 8.47E-05 |
| bta-miR-2429_R19-5L20 | Traes_3AS_369371CF7 | 0.992486563 | 8.45E-05 |
| nta-miR156f | Traes_3AS_369371CF7 | 0.992486563 | 8.45E-05 |
| tae-m4030-5p | Traes_3AS_369371CF7 | 0.992486563 | 8.45E-05 |
| tae-m4349-5p | Traes_3AS_369371CF7 | 0.992486563 | 8.45E-05 |
| tae-m4218-5p | Traes_1DS_2B4F536B1 | 0.992500568 | 8.42E-05 |
| tae-m1456-5p | Traes_5DS_E289017BD | 0.99250311 | 8.41E-05 |
| tae-m0488-5p | Traes_4AL_234E1CDF6 | 0.992506769 | 8.40E-05 |
| tae-m4293-5p | Traes_4AL_234E1CDF6 | 0.992506769 | 8.40E-05 |
| tch-miR-378a-3p | TRAES3BF110000010CFD_g | 0.992527706 | 8.35E-05 |
| tae-m0982-5p | Traes_2BS_9B34A7A43 | 0.992532478 | 8.34E-05 |
| bta-miR-2429_R19-5L20 | TRAES3BF027700220CFD_g | 0.992534083 | 8.34E-05 |
| nta-miR156f | TRAES3BF027700220CFD_g | 0.992534083 | 8.34E-05 |
| tae-m4030-5p | TRAES3BF027700220CFD_g | 0.992534083 | 8.34E-05 |
| tae-m4349-5p | TRAES3BF027700220CFD_g | 0.992534083 | 8.34E-05 |
| oha-miR-22a_R1-19L22 | Traes_2DS_3E640A897 | 0.992536678 | 8.33E-05 |
| tae-m1521-5p | Traes_6DS_AB82EC92A | 0.992551323 | 8.30E-05 |
| tae-m2489-5p | Traes_6DS_AB82EC92A | 0.992551323 | 8.30E-05 |
| tae-m4416-5p | Traes_6DS_AB82EC92A | 0.992551323 | 8.30E-05 |
| tch-miR-378a-3p | Traes_2DS_B77283745 | 0.99255202 | 8.30E-05 |
| tae-m1456-5p | TRAES3BF003200130CFD_g | 0.992552919 | 8.30E-05 |
| tae-m2333-5p | Traes_1BL_4FE03D7B0 | 0.992556514 | 8.29E-05 |
| tae-m3808-5p | Traes_1BL_4FE03D7B0 | 0.992556514 | 8.29E-05 |
| efu-miR-9189d_R3-17L23 | Traes_3AS_B7522D4FE | 0.99256152 | 8.28E-05 |
| osa-miR5539b_R1-16L22 | Traes_2BL_1B2A85367 | 0.992565064 | 8.27E-05 |
| tae-m2333-5p | Traes_1BL_3A901257A | 0.992565139 | 8.27E-05 |
| tae-m3808-5p | Traes_1BL_3A901257A | 0.992565139 | 8.27E-05 |
| tae-m0431-5p | Traes_4DS_805590E76 | 0.992566866 | 8.27E-05 |
| zma-miR395l-5p_R21-7L22 | Traes_7AS_E98EFC7A8 | 0.992574483 | 8.25E-05 |
| tae-m4432-5p | Traes_2AS_6CCC35B60 | 0.992577019 | 8.24E-05 |
| bta-miR-2429_R19-5L20 | Traes_5DL_C0A5AC8F4 | 0.99257813 | 8.24E-05 |
| nta-miR156f | Traes_5DL_C0A5AC8F4 | 0.99257813 | 8.24E-05 |
| tae-m4030-5p | Traes_5DL_C0A5AC8F4 | 0.99257813 | 8.24E-05 |
| tae-m4349-5p | Traes_5DL_C0A5AC8F4 | 0.99257813 | 8.24E-05 |
| ppy-miR-4451_R1-16L18 | Traes_5DS_2F6D9BD24 | 0.992598114 | 8.20E-05 |
| cme-miR156g_R1-18L20 | Traes_2DL_4C8968BAF | 0.992598527 | 8.20E-05 |
| tae-m2996-5p | Traes_2DL_4C8968BAF | 0.992598527 | 8.20E-05 |
| gga-miR-7475-5p_R20-6L20 | Traes_4BL_9BCD28A4E | 0.99261957 | 8.15E-05 |
| ata-miR167f-5p_17A-G | Traes_4BL_998689F24 | 0.992623015 | 8.14E-05 |
| bta-miR-760-5p_R17-3L22 | Traes_2AL_1598A938B | 0.992637089 | 8.11E-05 |
| cbr-miR-35a_R4-18L22 | Traes_2AL_1598A938B | 0.992637089 | 8.11E-05 |
| tae-m4294-5p | Traes_2AL_1598A938B | 0.992637089 | 8.11E-05 |
| efu-miR-9189d_R3-17L23 | Traes_1AL_BF0FB9CCC | 0.992640621 | 8.10E-05 |
| tae-m0143-5p | Traes_2AL_AC822B5BC | 0.992641294 | 8.10E-05 |
| ptr-miR-3937_R1-15L24 | Traes_1BL_EC3D3628A | 0.992645044 | 8.09E-05 |
| gma-miR5037a_R19-5L22 | Traes_3AS_9B31D1D81 | 0.992659183 | 8.06E-05 |
| efu-miR-9189d_R3-17L23 | Traes_1AL_88D49649D | 0.99266187 | 8.06E-05 |
| gma-miR5677_R15-1L21 | Traes_5DS_C87840EBA | 0.992663794 | 8.05E-05 |
| tae-m3603-5p | Traes_5DS_C87840EBA | 0.992663794 | 8.05E-05 |
| tae-m3782-5p | Traes_5DS_C87840EBA | 0.992663794 | 8.05E-05 |
| tae-m2333-5p | Traes_4AL_F24A42899 | 0.992674677 | 8.03E-05 |
| tae-m3808-5p | Traes_4AL_F24A42899 | 0.992674677 | 8.03E-05 |
| bdi-miR5198_R1-15L21 | Traes_6AS_D99AF3950 | 0.992676298 | 8.03E-05 |
| oha-miR-30e-5p_R1-24L25 | Traes_6AS_D99AF3950 | 0.992676298 | 8.03E-05 |
| ssp-miR444b.2_R15-1L21 | TRAES3BF073700180CFD_g | 0.992678776 | 8.02E-05 |
| gga-miR-7475-5p_R20-6L20 | Traes_7AL_65F481DB9 | 0.99269078 | 7.99E-05 |
| smo-miR159_R2-21L21 | Traes_1DL_F3EBE6998 | 0.99270353 | 7.97E-05 |
| tae-m1832-5p | Traes_1DL_F3EBE6998 | 0.99270353 | 7.97E-05 |
| tae-m2038-5p | Traes_1DL_F3EBE6998 | 0.99270353 | 7.97E-05 |
| tae-m3208-5p | Traes_1DL_F3EBE6998 | 0.99270353 | 7.97E-05 |
| oha-miR-22a_R1-19L22 | Traes_7AS_ED5778D91 | 0.992704879 | 7.96E-05 |
| oha-miR-199c-3p_R2-22L22 | Traes_3AL_729C392FB | 0.992707559 | 7.96E-05 |
| tae-m0599-5p | Traes_6AL_2EEA40050 | 0.992709456 | 7.95E-05 |
| tae-m1456-5p | Traes_5BL_68FCD3492 | 0.992710544 | 7.95E-05 |
| oha-miR-22a_R1-19L22 | Traes_6BS_881DA479E | 0.99271745 | 7.94E-05 |
| tae-m2748-5p | Traes_7BL_D5B527888 | 0.992721926 | 7.93E-05 |
| smo-miR159_R2-21L21 | TRAES3BF066400010CFD_g | 0.992722235 | 7.93E-05 |
| tae-m1832-5p | TRAES3BF066400010CFD_g | 0.992722235 | 7.93E-05 |
| tae-m2038-5p | TRAES3BF066400010CFD_g | 0.992722235 | 7.93E-05 |
| tae-m3208-5p | TRAES3BF066400010CFD_g | 0.992722235 | 7.93E-05 |
| oha-miR-199c-3p_R2-22L22 | Traes_2DS_3AC11B9D8 | 0.992731448 | 7.91E-05 |
| ata-miR172b-5p_R1-20L21 | Traes_6DL_28AA9AE0E | 0.992735928 | 7.90E-05 |
| bmo-miR-3302_R19-5L23 | Traes_6DL_28AA9AE0E | 0.992735928 | 7.90E-05 |
| tae-m0631-5p | Traes_6DL_28AA9AE0E | 0.992735928 | 7.90E-05 |
| tch-miR-378a-3p | Traes_7DL_2A079DBC4 | 0.992737284 | 7.89E-05 |
| mmu-miR-5627-5p_R18-4L22 | Traes_6AS_A3A4E4DFC | 0.992750406 | 7.86E-05 |
| gga-miR-7475-5p_R20-6L20 | Traes_2BL_A402261EC1 | 0.992779238 | 7.80E-05 |
| tae-m3294-3p | Traes_1AL_36B201285 | 0.992783282 | 7.79E-05 |
| bta-miR-2429_R19-5L20 | Traes_6BL_8D21A769D | 0.992784213 | 7.79E-05 |
| nta-miR156f | Traes_6BL_8D21A769D | 0.992784213 | 7.79E-05 |
| tae-m4030-5p | Traes_6BL_8D21A769D | 0.992784213 | 7.79E-05 |
| tae-m4349-5p | Traes_6BL_8D21A769D | 0.992784213 | 7.79E-05 |
| tae-m3294-3p | Traes_2AL_57C3C7FAC | 0.992784736 | 7.79E-05 |
| tae-m0982-5p | Traes_7AL_AA1C50065 | 0.992786002 | 7.79E-05 |
| oha-miR-22a_R1-19L22 | Traes_7AL_5BB4F681E | 0.992788531 | 7.78E-05 |
| ata-miR172b-5p_R1-20L21 | Traes_5BL_1315614DC | 0.992794211 | 7.77E-05 |
| bmo-miR-3302_R19-5L23 | Traes_5BL_1315614DC | 0.992794211 | 7.77E-05 |
| tae-m0631-5p | Traes_5BL_1315614DC | 0.992794211 | 7.77E-05 |
| tae-m3294-3p | Traes_6DL_24A8AB125 | 0.992796169 | 7.77E-05 |
| tae-m2597-5p | Traes_4BL_E667A799C | 0.992809717 | 7.74E-05 |
| tae-m0298-5p | Traes_2AL_C69D52D2E | 0.992834923 | 7.68E-05 |
| ata-miR167e-5p_R1-21L21 | Traes_2AL_905D1E0E7 | 0.992839106 | 7.67E-05 |
| tae-m3157-5p | Traes_2AL_905D1E0E7 | 0.992839106 | 7.67E-05 |
| bmo-miR-3208_R4-19L22 | Traes_2BL_8101DFF11 | 0.992843979 | 7.66E-05 |
| tae-m1452-5p | Traes_2BL_8101DFF11 | 0.992843979 | 7.66E-05 |
| tae-m4432-5p | Traes_5AL_E5FB83F56 | 0.992854084 | 7.64E-05 |
| tae-m0298-5p | Traes_4DS_4C1265002 | 0.992860963 | 7.63E-05 |
| tch-miR-378a-3p | Traes_1DS_3C40BAEFD | 0.99287266 | 7.60E-05 |
| bta-miR-2429_R19-5L20 | Traes_7BL_311DDF828 | 0.992876477 | 7.59E-05 |
| nta-miR156f | Traes_7BL_311DDF828 | 0.992876477 | 7.59E-05 |
| tae-m4030-5p | Traes_7BL_311DDF828 | 0.992876477 | 7.59E-05 |
| tae-m4349-5p | Traes_7BL_311DDF828 | 0.992876477 | 7.59E-05 |
| gma-miR5037a_R19-5L22 | Traes_2DL_E37ECDD6E | 0.992876574 | 7.59E-05 |
| tae-m0044-5p | Traes_2AL_1A5B6CF28 | 0.992878394 | 7.59E-05 |
| tae-miR1137a_R15-1L20 | Traes_2AS_7941B9D9C | 0.992883377 | 7.58E-05 |
| tch-miR-378a-3p | Traes_5BL_A0350B6AB | 0.992884493 | 7.58E-05 |
| tae-m0147-5p | Traes_2DL_FBABE17F1 | 0.992897735 | 7.55E-05 |
| tae-m3576-5p | Traes_2DL_FBABE17F1 | 0.992897735 | 7.55E-05 |
| tae-m3683-5p | Traes_2DL_FBABE17F1 | 0.992897735 | 7.55E-05 |
| ata-miR172b-5p_R1-20L21 | Traes_6DS_AA77F7548 | 0.992901653 | 7.54E-05 |
| bmo-miR-3302_R19-5L23 | Traes_6DS_AA77F7548 | 0.992901653 | 7.54E-05 |
| tae-m0631-5p | Traes_6DS_AA77F7548 | 0.992901653 | 7.54E-05 |
| tae-m2069-5p | Traes_7DS_35615C7D4 | 0.992904693 | 7.53E-05 |
| bmo-miR-3208_R4-19L22 | Traes_5BL_668C8B975 | 0.992908618 | 7.53E-05 |
| tae-m1452-5p | Traes_5BL_668C8B975 | 0.992908618 | 7.53E-05 |
| hvu-miR6209_R15-1L20 | Traes_4BL_2A47728EB | 0.992909337 | 7.52E-05 |
| bmo-miR-3208_R4-19L22 | Traes_5DL_B1E6692B6 | 0.992917915 | 7.51E-05 |
| tae-m1452-5p | Traes_5DL_B1E6692B6 | 0.992917915 | 7.51E-05 |
| tae-miR9659-3p | Traes_2DL_5436C046D | 0.992921969 | 7.50E-05 |
| gga-miR-7475-5p_R20-6L20 | Traes_2BL_D94A13D35 | 0.992924515 | 7.49E-05 |
| tae-miR1133_R19-5L22 | Traes_4BS_DA73ED408 | 0.992927768 | 7.48E-05 |
| tae-m1456-5p | Traes_5AS_32C705A83 | 0.992928194 | 7.48E-05 |
| gga-miR-7475-5p_R20-6L20 | Traes_4AS_A65DDCD05 | 0.992931994 | 7.48E-05 |
| gma-miR5037a_R19-5L22 | Traes_1DL_674796210 | 0.992936334 | 7.47E-05 |
| gga-miR-7475-5p_R20-6L20 | Traes_2DL_D02E6E48B | 0.992937381 | 7.46E-05 |
| bbe-miR-4857-5p_R3-17L23 | Traes_2BS_74DFF2FD5 | 0.992937658 | 7.46E-05 |
| hsa-miR-3677-5p_R17-3L22 | Traes_2BS_74DFF2FD5 | 0.992937658 | 7.46E-05 |
| bmo-miR-3208_R4-19L22 | Traes_2AS_BF5FA8832 | 0.992942447 | 7.45E-05 |
| tae-m1452-5p | Traes_2AS_BF5FA8832 | 0.992942447 | 7.45E-05 |
| tae-m4429-5p | Traes_2DL_1C63A1042 | 0.992945966 | 7.45E-05 |
| oha-miR-99a-5p | Traes_2BL_004BCF477 | 0.992955348 | 7.43E-05 |
| tch-miR-378a-3p | Traes_4DL_A76BE77E4 | 0.992967144 | 7.40E-05 |
| tae-m0982-5p | Traes_4BL_19891B81D1 | 0.992973615 | 7.39E-05 |
| tae-m1521-5p | Traes_4DL_C3044FB58 | 0.992992793 | 7.35E-05 |
| tae-m2489-5p | Traes_4DL_C3044FB58 | 0.992992793 | 7.35E-05 |
| tae-m4416-5p | Traes_4DL_C3044FB58 | 0.992992793 | 7.35E-05 |
| smo-miR159_R2-21L21 | Traes_1AL_1EBD4E9F5 | 0.993002577 | 7.33E-05 |
| tae-m1832-5p | Traes_1AL_1EBD4E9F5 | 0.993002577 | 7.33E-05 |
| tae-m2038-5p | Traes_1AL_1EBD4E9F5 | 0.993002577 | 7.33E-05 |
| tae-m3208-5p | Traes_1AL_1EBD4E9F5 | 0.993002577 | 7.33E-05 |
| oha-miR-199c-3p_R2-22L22 | Traes_4BL_C09256DE9 | 0.993008611 | 7.31E-05 |
| tae-m3294-3p | Traes_6DL_3AAE464EA | 0.993017439 | 7.30E-05 |
| bdi-miR390a-3p | Traes_5BS_1D2221052 | 0.993024509 | 7.28E-05 |
| tae-m3504-5p | Traes_7BL_342CAC6CE | 0.993038041 | 7.25E-05 |
| bta-miR-2429_R19-5L20 | Traes_7AS_F1E2FE61B | 0.993045859 | 7.24E-05 |
| nta-miR156f | Traes_7AS_F1E2FE61B | 0.993045859 | 7.24E-05 |
| tae-m4030-5p | Traes_7AS_F1E2FE61B | 0.993045859 | 7.24E-05 |
| tae-m4349-5p | Traes_7AS_F1E2FE61B | 0.993045859 | 7.24E-05 |
| oha-miR-22a_R1-19L22 | Traes_7DS_77FC13D7B1 | 0.993047576 | 7.23E-05 |
| tae-m3312-5p | Traes_2AS_BDB406ADE | 0.993054143 | 7.22E-05 |
| ata-miR172b-5p_R1-20L21 | Traes_7DS_3CC764005 | 0.993058326 | 7.21E-05 |
| bmo-miR-3302_R19-5L23 | Traes_7DS_3CC764005 | 0.993058326 | 7.21E-05 |
| tae-m0631-5p | Traes_7DS_3CC764005 | 0.993058326 | 7.21E-05 |
| tae-m1521-5p | Traes_2DS_A380503DA | 0.993070127 | 7.19E-05 |
| tae-m2489-5p | Traes_2DS_A380503DA | 0.993070127 | 7.19E-05 |
| tae-m4416-5p | Traes_2DS_A380503DA | 0.993070127 | 7.19E-05 |
| tch-miR-378a-3p | Traes_4DS_4C6846850 | 0.993077176 | 7.17E-05 |
| tae-m2748-5p | Traes_2DL_A8E6D38B1 | 0.993089146 | 7.15E-05 |
| tae-m3821-3p | Traes_3AL_61E79425F | 0.993095249 | 7.13E-05 |
| tae-miR9659-3p | Traes_2DL_04535D371 | 0.993119771 | 7.08E-05 |
| mmu-miR-5627-5p_R18-4L22 | Traes_4AS_8A64DBE8E | 0.993124089 | 7.08E-05 |
| tae-m0025-5p | Traes_5DS_B9BFD5BEC | 0.993134264 | 7.05E-05 |
| tae-m2737-5p | Traes_5DS_B9BFD5BEC | 0.993134264 | 7.05E-05 |
| ata-miR167e-5p_R1-21L21 | Traes_2DS_4646885A5 | 0.99314612 | 7.03E-05 |
| tae-m3157-5p | Traes_2DS_4646885A5 | 0.99314612 | 7.03E-05 |
| ata-miR167e-5p_R1-21L21 | TRAES3BF159100010CFD_g | 0.993154427 | 7.01E-05 |
| tae-m3157-5p | TRAES3BF159100010CFD_g | 0.993154427 | 7.01E-05 |
| mmu-miR-3535_R7-21L26 | Traes_7DL_6AB639E60 | 0.993160453 | 7.00E-05 |
| tae-m0044-5p | Traes_4BS_0742E3F1D | 0.993164504 | 6.99E-05 |
| ata-miR167e-5p_R1-21L21 | Traes_1BL_71EF97B701 | 0.993168241 | 6.98E-05 |
| tae-m3157-5p | Traes_1BL_71EF97B701 | 0.993168241 | 6.98E-05 |
| smo-miR159_R2-21L21 | Traes_1DL_03D1DC620 | 0.993168278 | 6.98E-05 |
| tae-m1832-5p | Traes_1DL_03D1DC620 | 0.993168278 | 6.98E-05 |
| tae-m2038-5p | Traes_1DL_03D1DC620 | 0.993168278 | 6.98E-05 |
| tae-m3208-5p | Traes_1DL_03D1DC620 | 0.993168278 | 6.98E-05 |
| mtr-miR7701-5p_R18-4L21 | Traes_5AL_E03FF5EC3 | 0.993186956 | 6.95E-05 |
| bbe-miR-4857-5p_R3-17L23 | Traes_5AL_F30A5E7F8 | 0.993188973 | 6.94E-05 |
| hsa-miR-3677-5p_R17-3L22 | Traes_5AL_F30A5E7F8 | 0.993188973 | 6.94E-05 |
| tae-m0147-5p | Traes_5BL_8512C24F7 | 0.993206961 | 6.91E-05 |
| tae-m3576-5p | Traes_5BL_8512C24F7 | 0.993206961 | 6.91E-05 |
| tae-m3683-5p | Traes_5BL_8512C24F7 | 0.993206961 | 6.91E-05 |
| dme-miR-954-3p_R15-1L21 | TRAES3BF020200050CFD_g | 0.993213409 | 6.89E-05 |
| tae-m4429-5p | Traes_7AL_649E6547F | 0.993223401 | 6.87E-05 |
| bdi-miR390a-3p | Traes_2DS_5D09437DA | 0.993226552 | 6.87E-05 |
| tae-m0982-5p | Traes_1BS_64E9CC6E0 | 0.993249533 | 6.82E-05 |
| tae-m1521-5p | Traes_6DL_CD5689351 | 0.993251332 | 6.82E-05 |
| tae-m2489-5p | Traes_6DL_CD5689351 | 0.993251332 | 6.82E-05 |
| tae-m4416-5p | Traes_6DL_CD5689351 | 0.993251332 | 6.82E-05 |
| mmu-miR-5627-5p_R18-4L22 | Traes_5DL_59AAC9844 | 0.993265302 | 6.79E-05 |
| tae-m2665-5p | Traes_1BL_0302D9F63 | 0.993273971 | 6.77E-05 |
| tae-m4081-5p | Traes_1BL_0302D9F63 | 0.993273971 | 6.77E-05 |
| gra-miR7505b_R6-20L21 | Traes_1BL_0302D9F63 | 0.993273971 | 6.77E-05 |
| mml-miR-892c-3p_R1-17L22 | Traes_1BL_0302D9F63 | 0.993273971 | 6.77E-05 |
| tae-m0403-5p | Traes_1BL_0302D9F63 | 0.993273971 | 6.77E-05 |
| tae-m2876-5p | Traes_1BL_0302D9F63 | 0.993273971 | 6.77E-05 |
| gma-miR5037a_R19-5L22 | Traes_1AL_59800A13E | 0.993274177 | 6.77E-05 |
| zma-miR395l-5p_R21-7L22 | Traes_1DL_D5F3DA85C | 0.993275655 | 6.77E-05 |
| bta-miR-2429_R19-5L20 | Traes_6BL_30CAC7928 | 0.993287551 | 6.74E-05 |
| nta-miR156f | Traes_6BL_30CAC7928 | 0.993287551 | 6.74E-05 |
| tae-m4030-5p | Traes_6BL_30CAC7928 | 0.993287551 | 6.74E-05 |
| tae-m4349-5p | Traes_6BL_30CAC7928 | 0.993287551 | 6.74E-05 |
| tae-m0561-5p | Traes_4AL_EE0AD342B | 0.993308468 | 6.70E-05 |
| oha-miR-22a_R1-19L22 | Traes_2AS_64E41196E | 0.993328261 | 6.66E-05 |
| tae-miR1121_R3-21L22 | Traes_1DS_C6BE97C0E | 0.993348791 | 6.62E-05 |
| tch-miR-378a-3p | Traes_1DL_274235AB5 | 0.993349904 | 6.62E-05 |
| gga-miR-7475-5p_R20-6L20 | Traes_5BS_F93273CED | 0.993351079 | 6.62E-05 |
| tae-m4283-5p | Traes_7DS_425ED0B6D | 0.993374675 | 6.57E-05 |
| tch-miR-378a-3p | Traes_7AL_0B358E4B4 | 0.993391829 | 6.54E-05 |
| osa-miR166j-5p_R4-19L21 | Traes_1AL_492E5A536 | 0.993395863 | 6.53E-05 |
| gga-miR-7475-5p_R20-6L20 | Traes_7DS_5D913121E | 0.993409162 | 6.50E-05 |
| tae-miR156_R2-20L23 | Traes_5BS_8CD712BC5 | 0.993413796 | 6.49E-05 |
| bta-miR-2429_R19-5L20 | Traes_1AL_3B82F56A2 | 0.99342747 | 6.47E-05 |
| nta-miR156f | Traes_1AL_3B82F56A2 | 0.99342747 | 6.47E-05 |
| tae-m4030-5p | Traes_1AL_3B82F56A2 | 0.99342747 | 6.47E-05 |
| tae-m4349-5p | Traes_1AL_3B82F56A2 | 0.99342747 | 6.47E-05 |
| bta-miR-760-5p_R17-3L22 | Traes_5DL_A6B74DFE0 | 0.993452283 | 6.42E-05 |
| cbr-miR-35a_R4-18L22 | Traes_5DL_A6B74DFE0 | 0.993452283 | 6.42E-05 |
| tae-m4294-5p | Traes_5DL_A6B74DFE0 | 0.993452283 | 6.42E-05 |
| tae-m4429-5p | Traes_2BS_F71538C9E | 0.993455352 | 6.41E-05 |
| gma-miR5037a_R19-5L22 | Traes_2DL_1815C4C40 | 0.993456228 | 6.41E-05 |
| tch-miR-378a-3p | Traes_1BL_9C0934A77 | 0.993458434 | 6.40E-05 |
| tch-miR-378a-3p | Traes_2DS_CB9F42CCD | 0.993462963 | 6.40E-05 |
| bbe-miR-4857-5p_R3-17L23 | Traes_4DL_83C41A77C1 | 0.993472271 | 6.38E-05 |
| hsa-miR-3677-5p_R17-3L22 | Traes_4DL_83C41A77C1 | 0.993472271 | 6.38E-05 |
| tae-m3821-3p | Traes_6AL_8E3293C90 | 0.993482659 | 6.36E-05 |
| tae-m3294-3p | Traes_5BS_F93273CED | 0.993490887 | 6.34E-05 |
| gma-miR5037a_R19-5L22 | Traes_4AL_F24A42899 | 0.993504288 | 6.32E-05 |
| tae-m0982-5p | Traes_7AL_5C9D36038 | 0.993509996 | 6.30E-05 |
| tae-m0298-5p | Traes_2DS_093D3F605 | 0.993510613 | 6.30E-05 |
| ata-miR167e-5p_R1-21L21 | Traes_1AL_5EEF86979 | 0.993514071 | 6.30E-05 |
| tae-m3157-5p | Traes_1AL_5EEF86979 | 0.993514071 | 6.30E-05 |
| oha-miR-199c-3p_R2-22L22 | Traes_1DS_80F18285E | 0.993520273 | 6.28E-05 |
| tae-m2333-5p | Traes_1AL_59800A13E | 0.993522342 | 6.28E-05 |
| tae-m3808-5p | Traes_1AL_59800A13E | 0.993522342 | 6.28E-05 |
| tae-m0599-5p | Traes_3AS_E0768C642 | 0.993530719 | 6.26E-05 |
| bta-miR-2429_R19-5L20 | Traes_1BL_5ED9A20B31 | 0.993531602 | 6.26E-05 |
| nta-miR156f | Traes_1BL_5ED9A20B31 | 0.993531602 | 6.26E-05 |
| tae-m4030-5p | Traes_1BL_5ED9A20B31 | 0.993531602 | 6.26E-05 |
| tae-m4349-5p | Traes_1BL_5ED9A20B31 | 0.993531602 | 6.26E-05 |
| tae-m2161-3p | Traes_6DL_D83F439E1 | 0.993546116 | 6.23E-05 |
| tae-m4283-5p | Traes_6BL_090822FBB | 0.993549981 | 6.23E-05 |
| tae-m2333-5p | TRAES3BF044000030CFD_g | 0.993553111 | 6.22E-05 |
| tae-m3808-5p | TRAES3BF044000030CFD_g | 0.993553111 | 6.22E-05 |
| bta-miR-2429_R19-5L20 | Traes_4AL_71305BD00 | 0.993553903 | 6.22E-05 |
| nta-miR156f | Traes_4AL_71305BD00 | 0.993553903 | 6.22E-05 |
| tae-m4030-5p | Traes_4AL_71305BD00 | 0.993553903 | 6.22E-05 |
| tae-m4349-5p | Traes_4AL_71305BD00 | 0.993553903 | 6.22E-05 |
| ata-miR172b-5p_R1-20L21 | Traes_6DL_3E5249DE3 | 0.9935781 | 6.17E-05 |
| bmo-miR-3302_R19-5L23 | Traes_6DL_3E5249DE3 | 0.9935781 | 6.17E-05 |
| tae-m0631-5p | Traes_6DL_3E5249DE3 | 0.9935781 | 6.17E-05 |
| ata-miR167e-5p_R1-21L21 | Traes_3AS_AE28A51DB | 0.993602776 | 6.13E-05 |
| tae-m3157-5p | Traes_3AS_AE28A51DB | 0.993602776 | 6.13E-05 |
| ata-miR171a-3p_R1-20L21 | Traes_2AL_F83267140 | 0.993604233 | 6.12E-05 |
| gra-miR7505b_R6-20L21 | Traes_2BS_BDB1BA66F | 0.993604822 | 6.12E-05 |
| mml-miR-892c-3p_R1-17L22 | Traes_2BS_BDB1BA66F | 0.993604822 | 6.12E-05 |
| tae-m0403-5p | Traes_2BS_BDB1BA66F | 0.993604822 | 6.12E-05 |
| tae-m2665-5p | Traes_2BS_BDB1BA66F | 0.993604822 | 6.12E-05 |
| tae-m2876-5p | Traes_2BS_BDB1BA66F | 0.993604822 | 6.12E-05 |
| tae-m4081-5p | Traes_2BS_BDB1BA66F | 0.993604822 | 6.12E-05 |
| oha-miR-22a_R1-19L22 | Traes_5DL_448D838B8 | 0.993623592 | 6.09E-05 |
| mmu-miR-5627-5p_R18-4L22 | Traes_7AL_4297B209C | 0.993627783 | 6.08E-05 |
| tae-miR9659-3p | Traes_1DL_291C515F3 | 0.99363969 | 6.06E-05 |
| bbe-miR-4857-5p_R3-17L23 | Traes_2AL_34C9F0C76 | 0.993678556 | 5.98E-05 |
| hsa-miR-3677-5p_R17-3L22 | Traes_2AL_34C9F0C76 | 0.993678556 | 5.98E-05 |
| bta-miR-2429_R19-5L20 | Traes_2AS_DE74C38CD | 0.993684173 | 5.97E-05 |
| nta-miR156f | Traes_2AS_DE74C38CD | 0.993684173 | 5.97E-05 |
| tae-m4030-5p | Traes_2AS_DE74C38CD | 0.993684173 | 5.97E-05 |
| tae-m4349-5p | Traes_2AS_DE74C38CD | 0.993684173 | 5.97E-05 |
| tae-m2333-5p | Traes_4AL_8911073AB | 0.993695692 | 5.95E-05 |
| tae-m3808-5p | Traes_4AL_8911073AB | 0.993695692 | 5.95E-05 |
| tch-miR-378a-3p | Traes_2BL_3041037F1 | 0.993704288 | 5.93E-05 |
| tae-m1918-5p | Traes_1AL_6D7D639F3 | 0.993705648 | 5.93E-05 |
| tae-m2015-5p | Traes_1AL_6D7D639F3 | 0.993705648 | 5.93E-05 |
| tae-m3263-5p | Traes_1AL_6D7D639F3 | 0.993705648 | 5.93E-05 |
| tae-m4217-3p | Traes_1AL_6D7D639F3 | 0.993705648 | 5.93E-05 |
| tae-m1918-5p | Traes_4DL_BAFFF6E71 | 0.993717536 | 5.91E-05 |
| tae-m2015-5p | Traes_4DL_BAFFF6E71 | 0.993717536 | 5.91E-05 |
| tae-m3263-5p | Traes_4DL_BAFFF6E71 | 0.993717536 | 5.91E-05 |
| tae-m4217-3p | Traes_4DL_BAFFF6E71 | 0.993717536 | 5.91E-05 |
| tae-m2161-3p | Traes_1BL_325A2C8C0 | 0.993718092 | 5.91E-05 |
| gga-miR-7475-5p_R20-6L20 | Traes_1BL_930E75040 | 0.993721269 | 5.90E-05 |
| tae-miR9659-3p | Traes_6DL_D02E85399 | 0.993728286 | 5.89E-05 |
| tae-m1521-5p | Traes_5BL_F33327C47 | 0.993728466 | 5.89E-05 |
| tae-m2489-5p | Traes_5BL_F33327C47 | 0.993728466 | 5.89E-05 |
| tae-m4416-5p | Traes_5BL_F33327C47 | 0.993728466 | 5.89E-05 |
| oha-miR-99a-5p | Traes_5BL_EBD050E21 | 0.99372994 | 5.88E-05 |
| tae-miR9659-3p | Traes_1AL_34404D5D8 | 0.993752369 | 5.84E-05 |
| tae-m0982-5p | Traes_5DL_39A62425E | 0.993765695 | 5.82E-05 |
| tae-m3821-3p | Traes_1BL_05865F944 | 0.993768702 | 5.81E-05 |
| tae-m4283-5p | TRAES3BF066400020CFD_g | 0.993776613 | 5.80E-05 |
| tae-m1521-5p | Traes_7DL_7124BF4F0 | 0.99378472 | 5.78E-05 |
| tae-m2489-5p | Traes_7DL_7124BF4F0 | 0.99378472 | 5.78E-05 |
| tae-m4416-5p | Traes_7DL_7124BF4F0 | 0.99378472 | 5.78E-05 |
| oha-miR-22a_R1-19L22 | Traes_2BS_6AECC4811 | 0.993786832 | 5.78E-05 |
| dme-miR-954-3p_R15-1L21 | Traes_7BS_E90F35103 | 0.99379488 | 5.76E-05 |
| tae-m4330-5p | Traes_2BL_F49D48248 | 0.993798737 | 5.76E-05 |
| tae-m3294-3p | Traes_2BL_D506EE7D2 | 0.993801164 | 5.75E-05 |
| tae-m0982-5p | Traes_7DS_748C4CFCA | 0.993810578 | 5.73E-05 |
| tae-miR9659-3p | Traes_2DS_326E97FCE | 0.993822816 | 5.71E-05 |
| hvu-miR6209_R15-1L20 | TRAES3BF093600150CFD_g | 0.993828084 | 5.70E-05 |
| ssp-miR444b.2_R15-1L21 | Traes_2BL_433D3147C | 0.993833745 | 5.69E-05 |
| nta-miR156f | Traes_7DS_D42371FA3 | 0.993855374 | 5.65E-05 |
| tae-m4030-5p | Traes_7DS_D42371FA3 | 0.993855374 | 5.65E-05 |
| tae-m4349-5p | Traes_7DS_D42371FA3 | 0.993855374 | 5.65E-05 |
| bta-miR-2429_R19-5L20 | Traes_7DS_D42371FA3 | 0.993855374 | 5.65E-05 |
| hvu-miR6209_R15-1L20 | Traes_4AL_E57587E16 | 0.993856387 | 5.65E-05 |
| gma-miR5037a_R19-5L22 | Traes_6BL_168034B0D | 0.993858982 | 5.65E-05 |
| tae-m1521-5p | TRAES3BF042800080CFD_g | 0.993859714 | 5.64E-05 |
| tae-m2489-5p | TRAES3BF042800080CFD_g | 0.993859714 | 5.64E-05 |
| tae-m4416-5p | TRAES3BF042800080CFD_g | 0.993859714 | 5.64E-05 |
| tae-m2748-5p | Traes_2BS_4E5639007 | 0.993864683 | 5.63E-05 |
| mmu-miR-5627-5p_R18-4L22 | Traes_4AL_279526586 | 0.993870454 | 5.62E-05 |
| tae-m0195-5p | Traes_2BS_C8FB3060D | 0.993872848 | 5.62E-05 |
| bta-miR-2429_R19-5L20 | Traes_2AL_47B1A5BF2 | 0.993875253 | 5.62E-05 |
| nta-miR156f | Traes_2AL_47B1A5BF2 | 0.993875253 | 5.62E-05 |
| tae-m4030-5p | Traes_2AL_47B1A5BF2 | 0.993875253 | 5.62E-05 |
| tae-m4349-5p | Traes_2AL_47B1A5BF2 | 0.993875253 | 5.62E-05 |
| mmu-miR-7665-3p_R8-22L22 | Traes_5AS_C383279B1 | 0.993877794 | 5.61E-05 |
| tae-m4283-5p | Traes_6AL_00EDAC7DB | 0.993881763 | 5.60E-05 |
| tae-miR9659-3p | Traes_7DL_71ED1C153 | 0.993900345 | 5.57E-05 |
| oha-miR-199c-3p_R2-22L22 | Traes_1BL_B16C84B50 | 0.993917485 | 5.54E-05 |
| tae-m2333-5p | Traes_7AS_6F12B2064 | 0.993920053 | 5.53E-05 |
| tae-m3808-5p | Traes_7AS_6F12B2064 | 0.993920053 | 5.53E-05 |
| tae-miR9659-3p | Traes_2BL_F93E1C539 | 0.993923443 | 5.53E-05 |
| tae-m4283-5p | Traes_6BL_EE65A6BFD | 0.993928913 | 5.52E-05 |
| gga-miR-7475-5p_R20-6L20 | Traes_4DS_4C6846850 | 0.993931829 | 5.51E-05 |
| bta-miR-760-5p_R17-3L22 | Traes_4BL_36170B1A6 | 0.993946254 | 5.49E-05 |
| cbr-miR-35a_R4-18L22 | Traes_4BL_36170B1A6 | 0.993946254 | 5.49E-05 |
| tae-m4294-5p | Traes_4BL_36170B1A6 | 0.993946254 | 5.49E-05 |
| gra-miR7505b_R6-20L21 | Traes_1AL_6CDAB2400 | 0.993953483 | 5.47E-05 |
| mml-miR-892c-3p_R1-17L22 | Traes_1AL_6CDAB2400 | 0.993953483 | 5.47E-05 |
| tae-m0403-5p | Traes_1AL_6CDAB2400 | 0.993953483 | 5.47E-05 |
| tae-m2665-5p | Traes_1AL_6CDAB2400 | 0.993953483 | 5.47E-05 |
| tae-m2876-5p | Traes_1AL_6CDAB2400 | 0.993953483 | 5.47E-05 |
| tae-m4081-5p | Traes_1AL_6CDAB2400 | 0.993953483 | 5.47E-05 |
| mmu-miR-5106_R2-19L23 | Traes_6DL_3BCB83718 | 0.993957311 | 5.47E-05 |
| bdi-miR5181d_R7-21L21 | Traes_3DL_4A818FD98 | 0.994005878 | 5.38E-05 |
| mmu-miR-5106_R2-19L23 | Traes_4BL_536016EDD | 0.994020483 | 5.35E-05 |
| gga-miR-1665_R21-7L22 | Traes_1BL_6C39CF535 | 0.994047504 | 5.30E-05 |
| tae-m0754-5p | Traes_1BL_6C39CF535 | 0.994047504 | 5.30E-05 |
| tae-m1521-5p | Traes_2BS_1702DBE8E | 0.994048241 | 5.30E-05 |
| tae-m2489-5p | Traes_2BS_1702DBE8E | 0.994048241 | 5.30E-05 |
| tae-m4416-5p | Traes_2BS_1702DBE8E | 0.994048241 | 5.30E-05 |
| ssp-miR444b.2_R15-1L21 | Traes_6DS_CDB16CE3F | 0.994079849 | 5.25E-05 |
| tae-m0044-5p | Traes_5DL_AC0C3A9BD | 0.994081022 | 5.24E-05 |
| tch-miR-378a-3p | Traes_1BL_D4A0D0191 | 0.994085781 | 5.24E-05 |
| tae-m1521-5p | Traes_2BS_407669DEF | 0.994099545 | 5.21E-05 |
| tae-m2489-5p | Traes_2BS_407669DEF | 0.994099545 | 5.21E-05 |
| tae-m4416-5p | Traes_2BS_407669DEF | 0.994099545 | 5.21E-05 |
| tch-miR-378a-3p | Traes_2BS_91FF9EB1C | 0.994100054 | 5.21E-05 |
| tae-miR9659-3p | Traes_3AS_1DD0D7B7A | 0.994101538 | 5.21E-05 |
| oha-miR-199c-3p_R2-22L22 | Traes_1BL_751E80A9A | 0.994101881 | 5.21E-05 |
| dpr-miR397_R17-1L21 | Traes_1BL_5D23F5133 | 0.994107292 | 5.20E-05 |
| tae-m1322-5p | Traes_1BL_5D23F5133 | 0.994107292 | 5.20E-05 |
| tae-m3641-5p | Traes_1BL_5D23F5133 | 0.994107292 | 5.20E-05 |
| xtr-miR-428a_R7-22L22 | Traes_1BL_5D23F5133 | 0.994107292 | 5.20E-05 |
| tae-m4283-5p | Traes_7AL_282AB2B65 | 0.994130598 | 5.16E-05 |
| tae-m4222-3p | Traes_2DL_5DAAA0DF7 | 0.994138778 | 5.14E-05 |
| bta-miR-2429_R19-5L20 | Traes_4DL_2C636B5DB | 0.994150322 | 5.12E-05 |
| nta-miR156f | Traes_4DL_2C636B5DB | 0.994150322 | 5.12E-05 |
| tae-m4030-5p | Traes_4DL_2C636B5DB | 0.994150322 | 5.12E-05 |
| tae-m4349-5p | Traes_4DL_2C636B5DB | 0.994150322 | 5.12E-05 |
| gga-miR-7475-5p_R20-6L20 | Traes_1DL_5F13FD194 | 0.994153774 | 5.12E-05 |
| tae-m3709-5p | Traes_1BL_22EDF8650 | 0.994177753 | 5.07E-05 |
| sly-miR5300_R21-6L22 | Traes_3AL_A306585EF | 0.994179036 | 5.07E-05 |
| tch-miR-378a-3p | Traes_5DL_0ED2854E2 | 0.994186574 | 5.06E-05 |
| tae-m1918-5p | Traes_3AL_A5A6F8761 | 0.994203491 | 5.03E-05 |
| tae-m2015-5p | Traes_3AL_A5A6F8761 | 0.994203491 | 5.03E-05 |
| tae-m3263-5p | Traes_3AL_A5A6F8761 | 0.994203491 | 5.03E-05 |
[truncated: 47,938 more chars]
