# Supplementary material for: Genome-Wide Identification and Characterization of Long Non-Coding RNA in Wheat Roots in Response to Ca2+ Channel Blocker
Source: Front Plant Sci. 2018 Mar 6;9:244. doi: 10.3389/fpls.2018.00244 (PMC5845709; doi:10.3389/fpls.2018.00244)
Supplement: Supplementary file 4 [file Image1.pdf]

SUPPLEMENTARY INFORMATION 1: FIGURES

Genome-wide identification and characterization of long non-coding RNA in wheat roots in response to Ca^{2+} channel blocker

Keshi Ma[1, 2], Wenshuo Shi[1], Mengyue Xu[1], Jiaxi Liu[1] & Feixiong Zhang[1]

[1]College of Life Sciences, Capital Normal University, Beijing 100048, China.

[2]College of Life Science and Agronomy, Zhoukou Normal University, Zhoukou 466001, China. Correspondence and requests for materials should be addressed to F.Z. (email: fxzhang@cnu.edu.cn) or J.L. (email: liu-jiaxi@263.net)

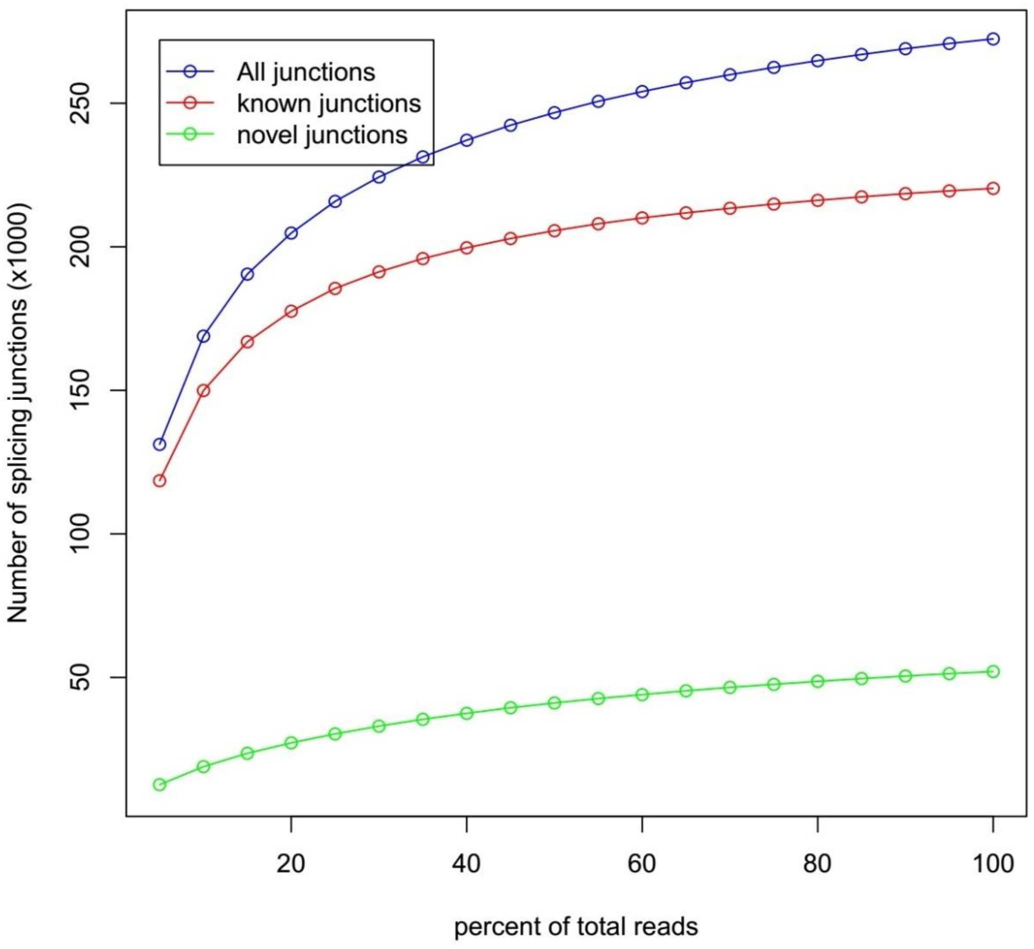

Figure S1. The saturation of lncRAs from RNA-seq measured by RSeQC.

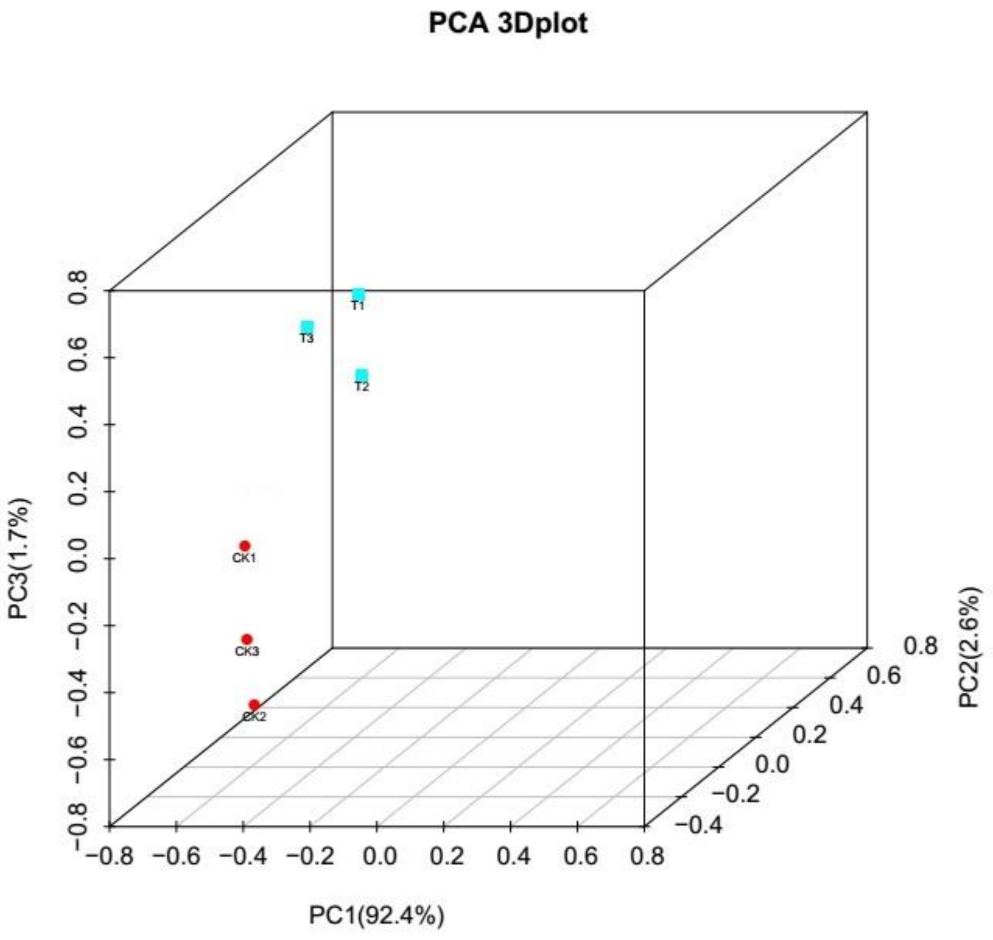

Figure S2. The cluster or PCA results of the six samples. CK (CK1, CK2, CK3): control (0 mM $LaCl_3$). T (T1, T2, T3): treatment (1.5 mM $LaCl_3$).

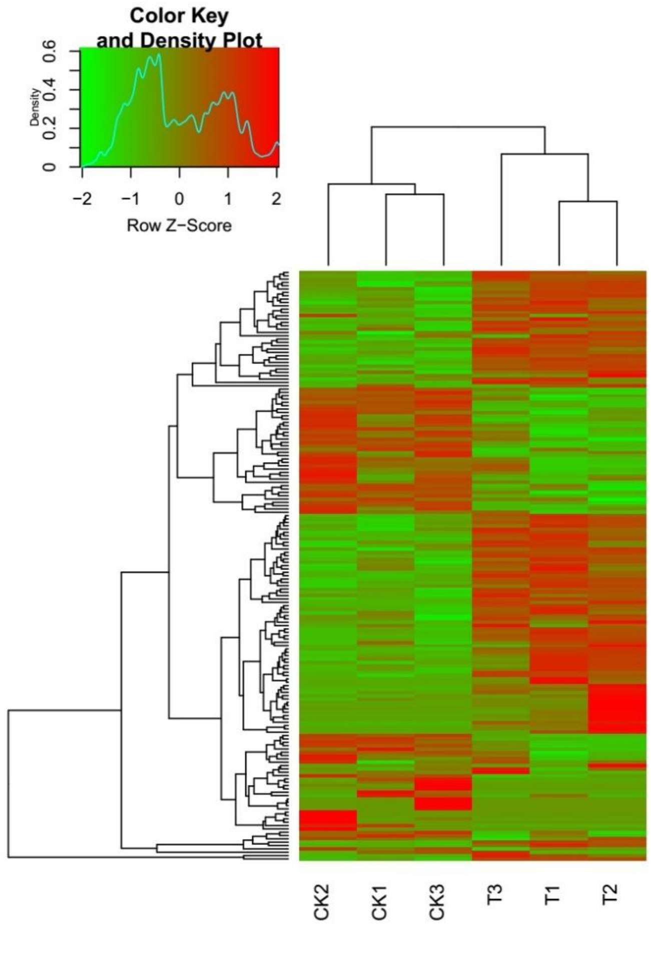

Figure S3. The heatmap of differently expressed lncRAs in wheat root responsive to Ca^{2+}-channel blocker. CK (CK1, CK2, CK3): control (0 mM $LaCl_3$). T (T1, T2, T3): treatment (1.5 mM $LaCl_3$).

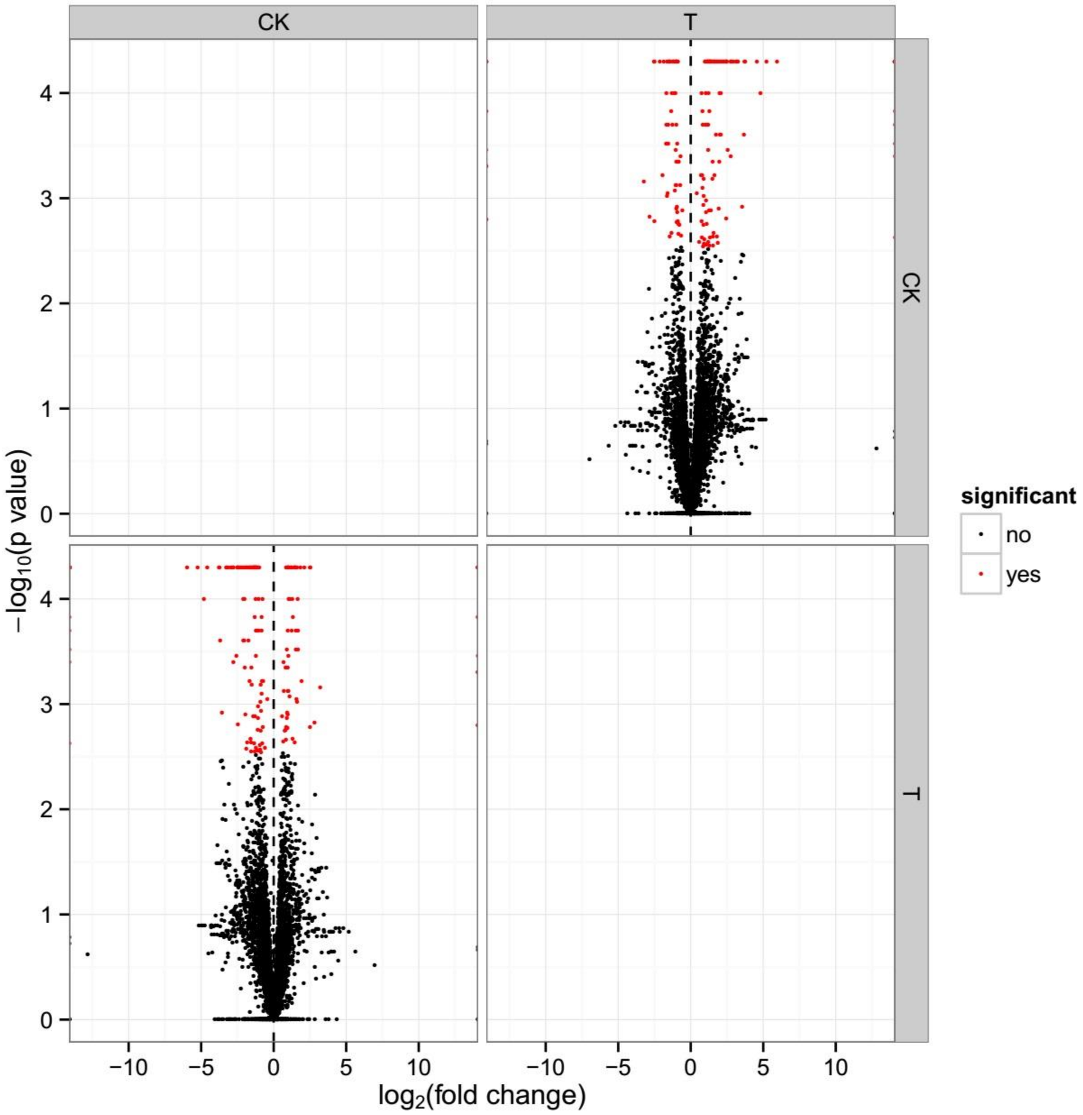

Figure S4. The Volcano matrix of lncRAs differently expressed in wheat root responsive to Ca^{2+}-channel blocker. CK: control (0 mM $LaCl_3$). T: treatment (1.5 mM $LaCl_3$).

a

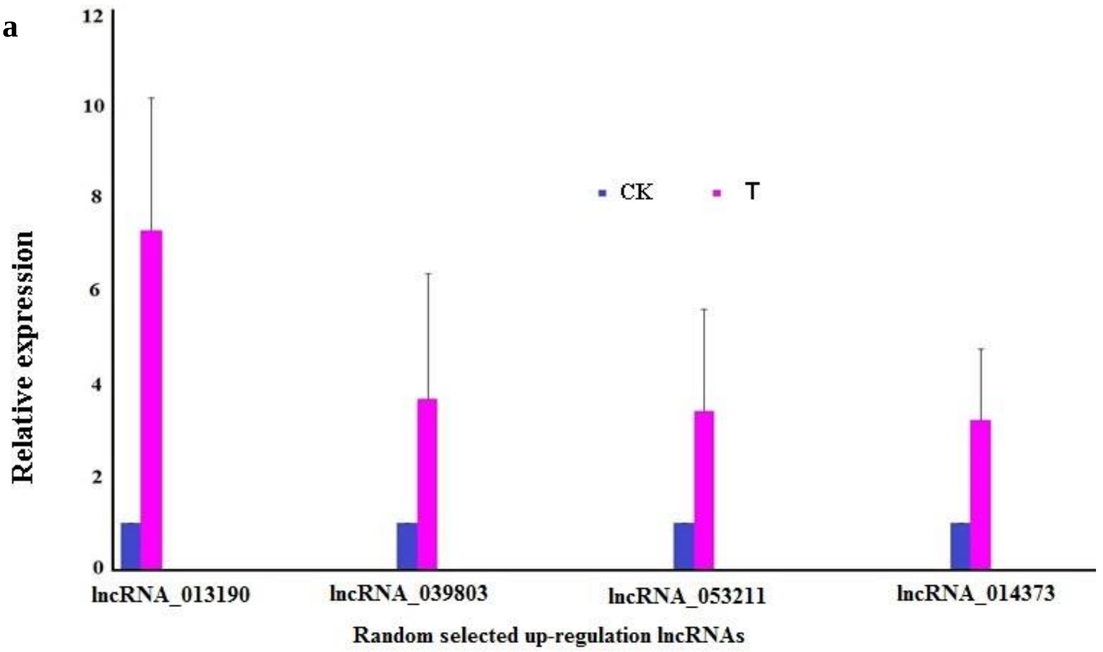

b

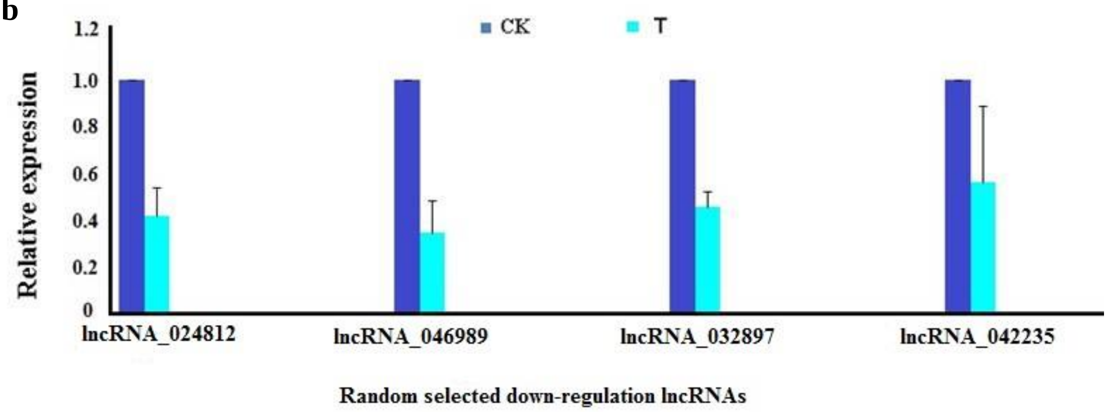

Figure S5. The availability of lncRNAs identified from RNA-seq verified by real-time qRT-PCR. (a) The relative expression of randomly selected down-regulated lncRNAs. (b) The relative expression of randomly selected up-regulated lncRNAs. All reactions were performed in triplicate.

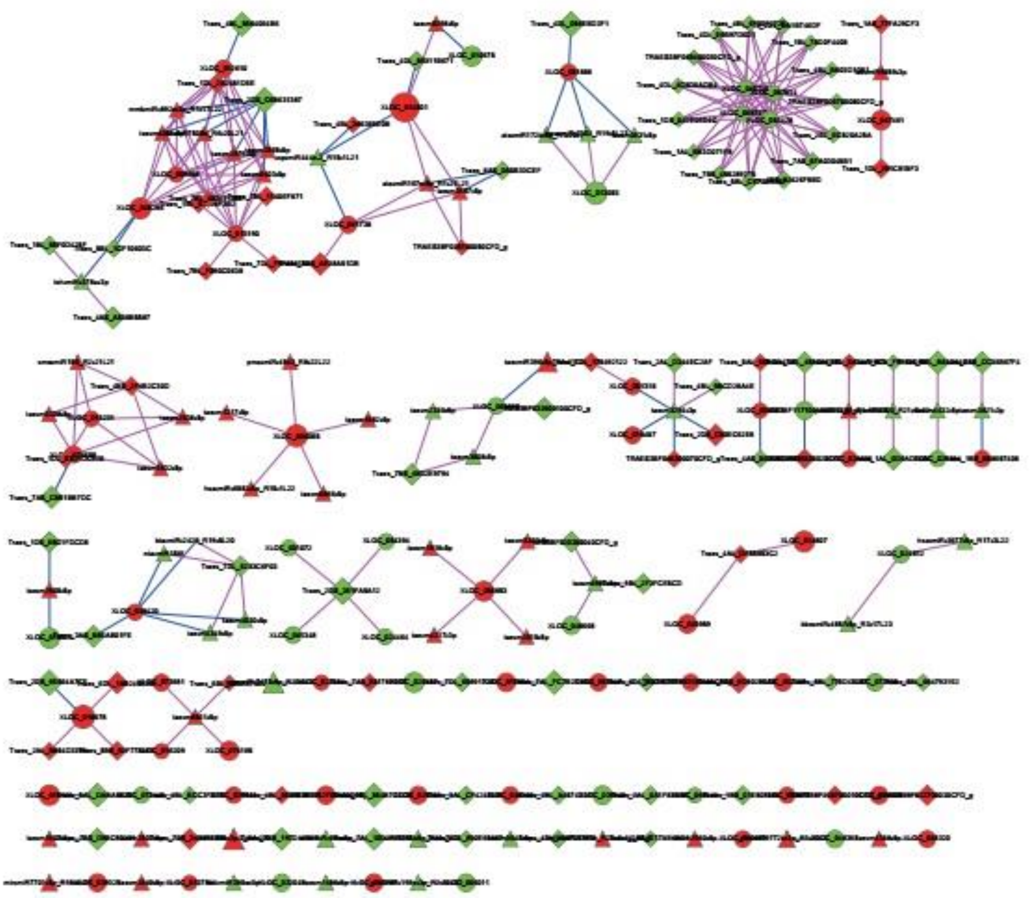

Figure S6. Cytoscape whole Network of miRNA-lncRNA-mRNA responsive lncRNA-regulated networks. Circle nodes: lncRNA. Square nodes: mRNA. Triangular nodes: miRNA. Red: up-regulation. Green: down-regulation. Node size positive relate to expression of gene. Pink edge: positive relation. Blue edge: negative relation.

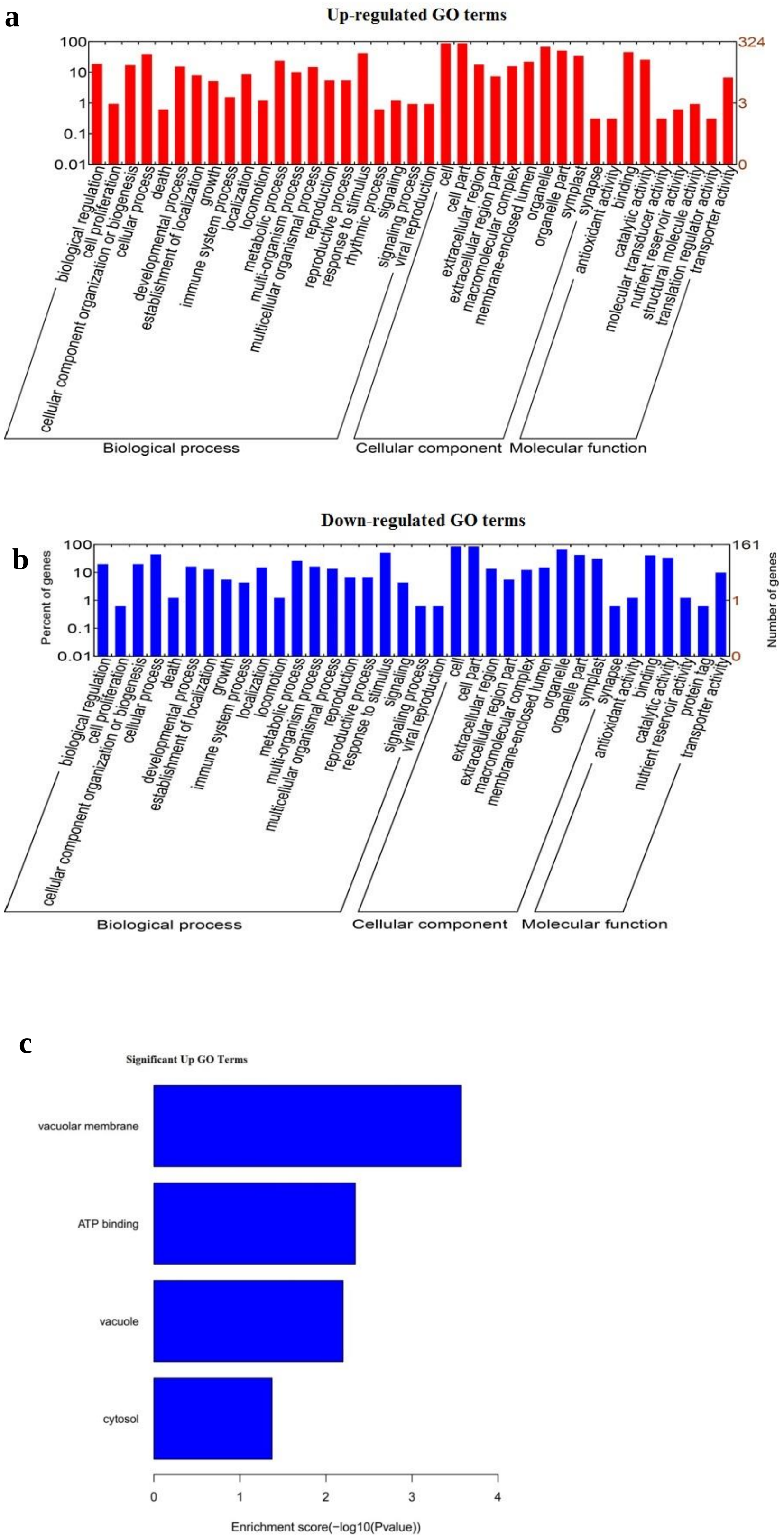
a
Up-regulated GO terms
100
10
1
0.1
0.01
324
3
0
biological regulation
cell proliferation
cellular component organization or biogenesis
cellular process
death
developmental process
establishment of localization
growth
immune system process
localization
locomotion
metabolic process
multi-organism process
multicellular organismal process
reproduction
reproductive process
response to stimulus
rhythmic process
signaling
signaling process
viral reproduction
cell
cell part
extracellular region
extracellular region part
macromolecular complex
membrane-enclosed lumen
organelle
organelle part
symplast
synapse
antioxidant activity
binding
catalytic activity
molecular transducer activity
nutrient reservoir activity
structural molecule activity
translation regulator activity
transporter activity
Biological process
Cellular component
Molecular function
b
Down-regulated GO terms
Percent of genes
Number of genes
100
10
1
0.1
0.01
161
1
0
biological regulation
cell proliferation
cellular component organization or biogenesis
cellular process
death
developmental process
establishment of localization
growth
immune system process
localization
locomotion
metabolic process
multi-organism process
multicellular organismal process
reproduction
reproductive process
response to stimulus
signaling
signaling process
viral reproduction
cell
cell part
extracellular region
extracellular region part
macromolecular complex
membrane-enclosed lumen
organelle
organelle part
symplast
synapse
antioxidant activity
binding
catalytic activity
nutrient reservoir activity
protein tag
transporter activity
Biological process
Cellular component
Molecular function
c
Significant Up GO Terms
vacuolar membrane
ATP binding
vacuole
cytosol
0
1
2
3
4
Enrichment score(−log10(Pvalue))

d

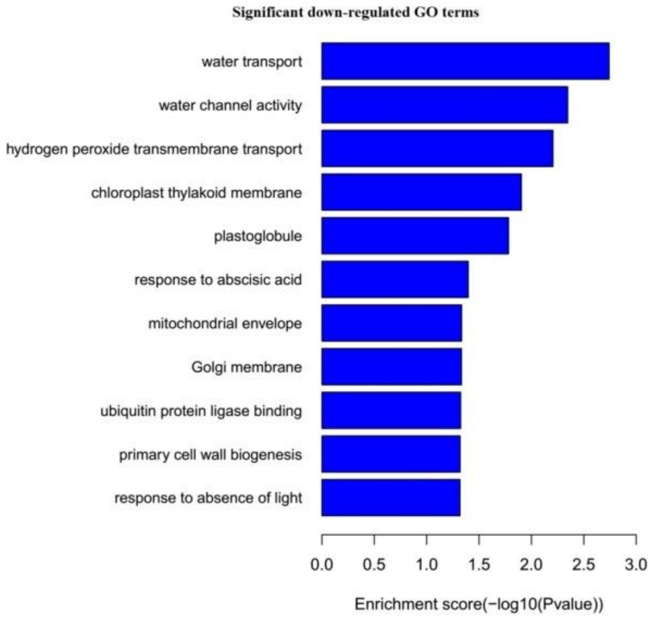
Significant down-regulated GO terms
water transport
water channel activity
hydrogen peroxide transmembrane transport
chloroplast thylakoid membrane
plastoglobule
response to abscisic acid
mitochondrial envelope
Golgi membrane
ubiquitin protein ligase binding
primary cell wall biogenesis
response to absence of light
0.0
0.5
1.0
1.5
2.0
2.5
3.0
Enrichment score(−log10(Pvalue))

e

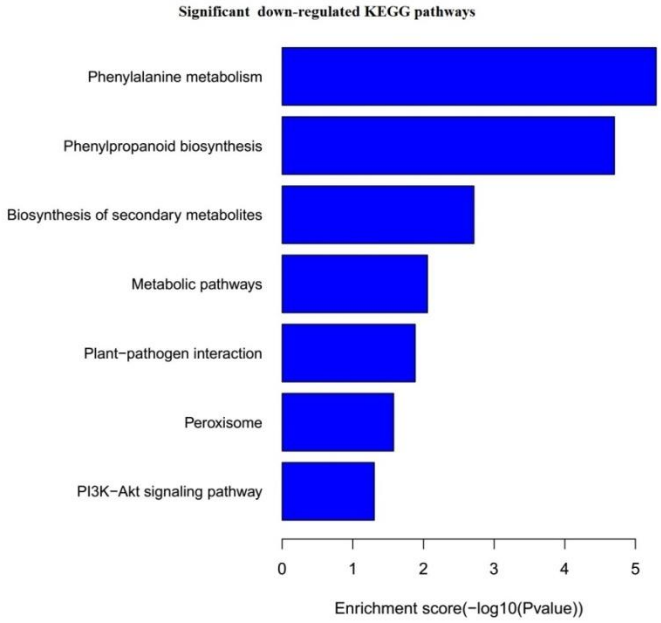
Significant down-regulated KEGG pathways
Phenylalanine metabolism
Phenylpropanoid biosynthesis
Biosynthesis of secondary metabolites
Metabolic pathways
Plant−pathogen interaction
Peroxisome
PI3K−Akt signaling pathway
0
1
2
3
4
5
Enrichment score(−log10(Pvalue))

f

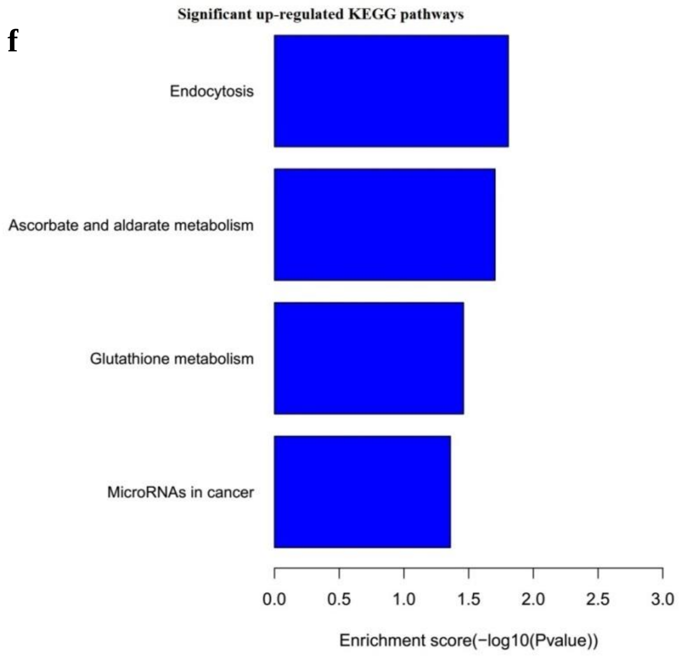

Figure S7. GO terms and KEGG pathways of differently expressed lncRNAs in wheat roots responsive to Ca^{2+}-blockage. In the bar chart, the GO annotation is presented as the X axis legend and percent of genes as the Y axis legend. (a) Up-regulated GO terms. (b) Down-regulated GO terms. (c) Significant up-regulated GO terms. (d) Significant down-regulated GO terms. (e) Significant up -regulated KEGG pathways. (f) Significant down-regulated KEGG pathways.

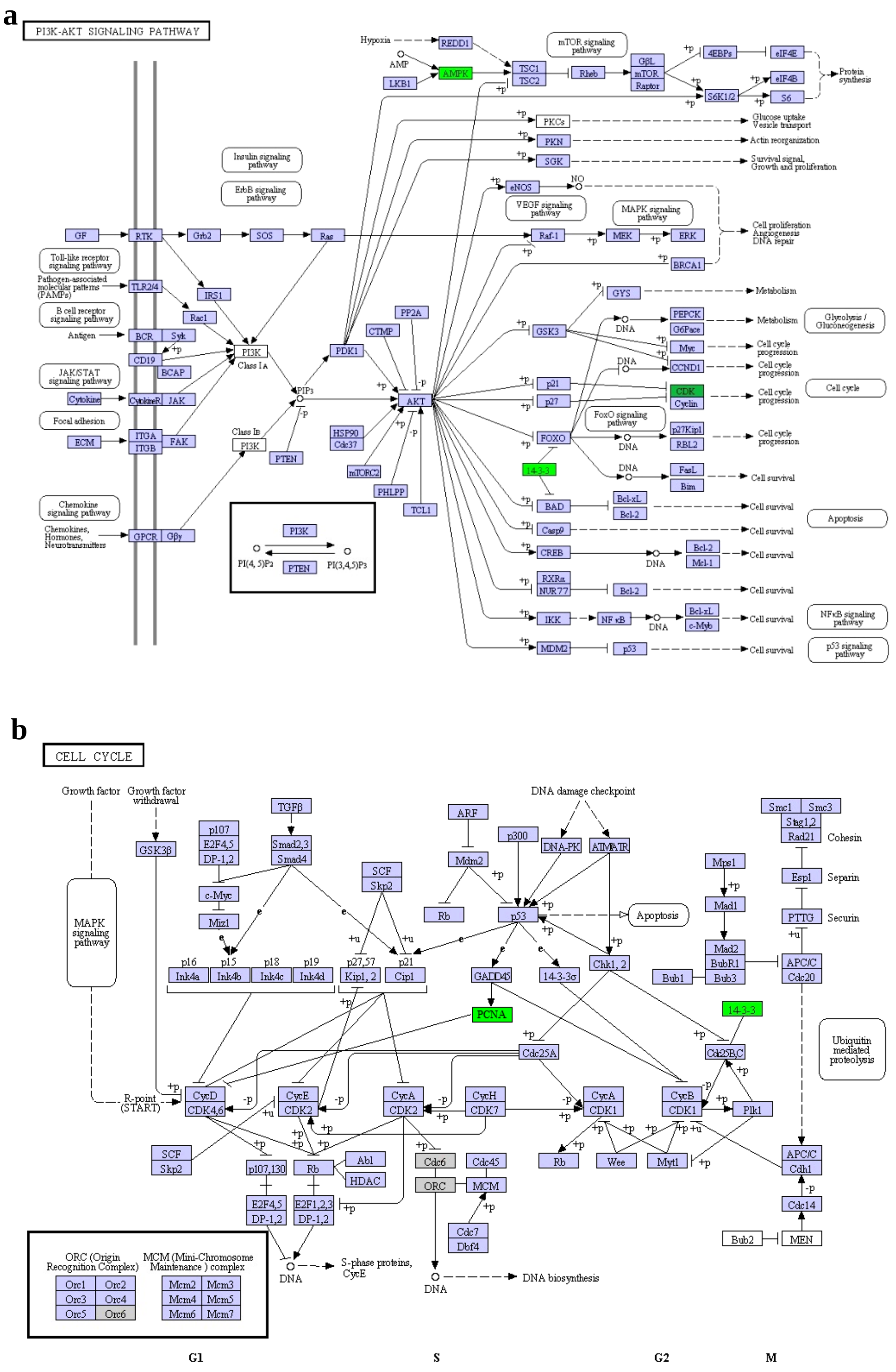

Figure S8. The expression of the 14-3-3 protein in the PI3K-AKT pathway and cell cycle pathway. Green: down-regulation. (a) The image indicates 14-3-3 down-regulation in the “PI3K-AKT signaling pathway”. (b) The image indicates 14-3-3 down-regulation in the “Cell cycle pathway”.
